# Assembling Hexahedral Supramolecular Nano‐Aggregates on Rice Wax Layer Matrices to Promote the Leaf Deposition and Bioavailability of Bactericides for Plant Protections

**DOI:** 10.1002/advs.202504225

**Published:** 2025-05-28

**Authors:** Run Yang, Jinghan Yang, Min Liu, Juan Liu, Peiyi Wang

**Affiliations:** ^1^ State Key Laboratory of Green Pesticide Key Laboratory of Green Pesticide and Agricultural Bioengineering Ministry of Education Center for Research and Development of Fine Chemicals of Guizhou University Guiyang 550025 China

**Keywords:** enhanced bioavailability, leaf deposition, preventing bacterial diseases, rice microcrystalline matrix, self‐assembly strategy

## Abstract

Naturally hydrophobic wax layer microstructures in plants seriously hinder the leaf adhesion and deposition of pesticide droplets, thereby causing low pesticide bioavailability and inevitable environmental pollution. Inspired by the supramolecular self‐assembly strategy, two anisotropic supramolecular building units (BiTA18@*β*‐CD and BiTA18@*γ*‐CD) are invented based on the host–guest complexation between a benzimidazole‐modified bactericidal molecule (BiTA18) and *β*‐/*γ*‐cyclodextrin (*β*‐CD/*γ*‐CD), which self‐assemble into nano‐sized hexagonal cuboids on the rice microcrystalline matrix. This consequence markedly enhances the retention of bactericidal ingredients on target plants. More intriguingly, these oligosaccharide‐coated supramolecular materials, with superior biocompatibility, can break through the bacterial biofilm barrier, limit bacterial motility and extracellular enzyme secretion, and induce electrolyte leakage and ROS accumulation in bacteria, ultimately annihilating the stubborn pathogenic bacterium. Combining these excellent advantages, the optimal supramolecular material (BiTA18@*β*‐CD) displays broad‐spectrum and efficient control efficacies of 54.4% and 71.7% against rice bacterial blight and citrus bacterial canker, respectively, surpassing those of kasugamycin (34.3%/34.1%), thiodiazole‐copper‐20%SC (39.9%/42.7%), and BiTA18 (42.7%/46.9%) at 200 *µg* mL^−1^. Besides, the current supramolecular systems are safe for non‐target organisms like earthworms and zebrafishes. This study provides a key inspiration for the construction of supramolecular building units assembled on rice microcrystalline substrates to improve the utilization of pesticides.

## Graphical abstract

To address the problem of off‐target movement and low bioavailability of pesticides, two anisotropic supramolecular building units (BiTA18@*β*‐CD and BiTA18@*γ*‐CD) have been invented that can self‐assemble into nano‐sized hexagonal cuboids on the rice microcrystalline matrix. This manipulation realizes the enhanced utilization of bactericides for rice and citrus protections.

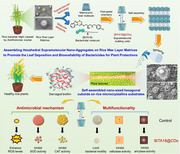

## 1 Introduction

Plant pathogenic bacteria, exemplified by notorious *Xanthomonas oryzae* pv. *oryzae* (*Xoo*) and *Xanthomonas axonopodis* pv. *citri* (*Xac*), are one of the main causes of plant diseases, leading to negative symptoms such as leaf blight, ulcers, yellowing, stunted growth, and even decay in various crops, which in turn reduce crop yields and cause significant economic losses.^[^ ^1^ ^]^ Although various prevention and control methods based on agricultural chemicals have been used, their effectiveness has been limited.^[^ ^2^ ^]^ Through years of research and investigation, scientists have identified a key reason for the substantial reduction in the effectiveness of bactericides: pathogenic bacteria have evolved defense mechanisms over time, leading to the formation of a 3D matrix known as a “biofilm”.^[^ ^3^ ^]^ This defensive biofilm is primarily composed of large amounts of extracellular polymers, including polysaccharides and proteins, which not only provide a solid physical barrier for the bacteria but also protect them from external antimicrobial agents and attacks from the immune system of plant.^[^ ^4^ ^]^ Furthermore, bacteria within the biofilm can exchange nutrients and signaling molecules through matrix channels, significantly enhancing their resistance and survival, which complicates disease prevention and control efforts.^[^ ^5^ ^]^ In response to this challenge, although existing commercial agrochemicals, such as agricultural antibiotics (kasugamycin, zhongshengmycin, etc.) and copper‐based bactericides (copper hydroxide, thiodiazole‐copper, etc.), have been somewhat effective in controlling plant pathogenic bacteria by inhibiting their spread, they exhibit low‐level functions in breaking through the stubborn biofilm barriers. Moreover, these chemicals often struggle to penetrate the biofilm barrier, rendering them ineffective in eliminating the bacterial populations within biofilm.^[^ ^6^ ^]^ This disappointed circumstance makes biofilm‐associated plant diseases particularly persistent and difficult to treat in agriculture, exacerbating the spread of bacterial infections and increasing crop losses. Consequently, there is an urgent need to develop new bactericidal strategies specifically designed to target and destroy biofilms, thereby more effectively addressing biofilm‐associated infections.

From another aspect, during pesticide spraying, many droplets are easily carried away by wind speed and direction, causing them to drift off‐target and significantly reducing pesticide efficiency.^[^ ^7^ ^]^ Even when some droplets reach the plant leaf surface, the hydrophobic waxy cuticle covering the leaves still poses a challenge. This waxy layer matrix reduces the affinity between the droplets and the leaf surface, preventing the droplets from spreading, and causing them to maintain a near‐spherical shape, thereby reducing contact area with the leaf. As a consequence, droplets tend to bounce or slide off the surface, further reducing effective pesticide deposition.^[^ ^8^ ^]^ This phenomenon is particularly noticeable in certain plants. For example, the surface of rice leaves is covered with wax crystals forming micro‐scale rough structures, which enhance the hydrophobicity of the leaves.^[^ ^9^ ^]^ When droplets come into contact with this leaf surface, they struggle to spread or adhere. Instead, they often roll or bounce off, making it difficult for them to remain on the leaf. These surface characteristics not only reduce the effective deposition of pesticides on the leaves, but also significantly decrease the transmission efficiency of the pesticide, making disease control in these crops more challenging.^[^ ^10^ ^]^ Facing this additional dilemma, the invention of advanced bactericidal materials that can self‐assemble on the waxy layer microcrystalline matrix to promote the retention and deposition of pesticides is an innovative response strategy. To the best of our knowledge, the development of self‐assembled materials on hydrophobic leaf substrates have rarely been reported and proposed.

Currently, wetting and dispersing agents play a crucial role in the field of pesticide adjuvants, helping to improve the adhesion, spreading, and deposition of pesticides on plant leaves. Several common and widely used wetting agents include organosilicone (OS) and nonylphenol ethoxylates (NPE), both of which offer substantial advantages in enhancing droplet spreading and reducing rebound on hydrophobic leaf surfaces due to their ultra‐low surface tension and excellent wetting properties.^[^ ^11^ ^]^ However, the high cost of these agents limits their widespread use, and they may leave persistent residues in soil and water, posing potential environmental risks.^[^ ^12^ ^]^ Moreover, with increasingly stringent environmental regulations, these auxiliary agents are being gradually restricted, and many regions and countries have begun phasing them out.^[^ ^13^ ^]^ As an eco‐friendly wetting agent, alkyl polyglucoside (APG) has gained increasing attention in recent years. Its good biodegradability and certain wetting properties make it an ideal choice in many pesticide formulations. While APG excels in terms of environmental sustainability, its wetting performance on superhydrophobic crop leaves is less effective compared to more powerful wetting agents, such as OS‐based surfactants.^[^ ^14^ ^]^ Based on this, developing a multifunctional bactericide capable of breaking down bacterial biofilms, achieving effective deposition on hydrophobic leaves, and being environmentally friendly is crucial for advancing the next generation of agricultural bactericide.

With the booming development of advanced materials science, supramolecular self‐assembly strategies based on host–guest chemistry display great potential in solving the above challenges.^[^ ^15^ ^]^ Through the manipulation of host–guest interaction, diverse anisotropic supramolecular building units can be reasonably created and have self‐assembly properties on differential substrates.^[^ ^16^ ^]^ This adjustable self‐assembly characteristic not only effectively improves the solubility, permeability and biocompatibility of active molecules, but also potentially reduces the splash and bounce phenomenon by adjusting the surface tension of the solution, and increases the retention of active ingredients on the hydrophobic surface.^[^ ^17^ ^]^ These intriguing consequences will ultimately enhance the deposition efficiency and overall bioavailability of the pesticide on target plants.^[^ ^18^ ^]^ In general, host–guest recognition is achieved through the complementarity of molecular shape and size, so that active small molecules can be embedded in the hydrophobic cavity of the host molecule to form a stable inclusion complex as the new building block.^[^ ^19^ ^]^ This precise assembly strategy can not only improve the stability of the active molecule, but also optimize the surface tension and contact angle by adjusting the molecular surface properties.^[^ ^20^ ^]^ Among the commonly used macrocyclic host compounds, exemplified by cyclodextrins (CDs), cucurbiturils, crown ethers and calixarenes, CDs are favored and widely applied for their low cost, unique external hydrophobic and internal hydrophilic structure, high selectivity, excellent biocompatibility and chemical stability.^[^ ^21^ ^]^ Besides this, CDs are composed of 6–8 glucose residues bound by *β*‐1,4‐glycosidic bonds and naturally have good ecological safety and biodegradability.^[^ ^22^ ^]^ More importantly, CDs can match various hydrophobic active molecules to form host–guest inclusions, which will further self‐assemble into hierarchical aggregates with different sizes and morphologies.^[^ ^23^ ^]^ Inspired by these outstanding superiorities, the development of a kind of multifunctional CDs‐involved host–guest building units that can self‐assemble into sequenced aggregates on wax layer microcrystallite matrices will promote the leaf deposition and bioavailability of bactericides for plant protections. On the other hand, the choice of guest molecules directly determines the functionality of constructed supramolecular materials. Benzimidazole and its derivatives, possessing significant antimicrobial activities, can be suitably embedded in the hydrophobic cavity of CDs due to their good size matching and effective hydrophobic driving force.^[^ ^24^ ^]^ Additionally, benzimidazole derivatives have been reported to possess anti‐biofilm properties against medical bacteria, such as *Staphylococcus aureus* and *Pseudomonas aeruginosa*.^[^ ^25^ ^]^ Therefore, the invention of biocompatible CDs/benzimidazole co‐assembled supramolecular materials is expected to solve low pesticide bioavailability in plant bacterial disease control.

**Scheme 1:**
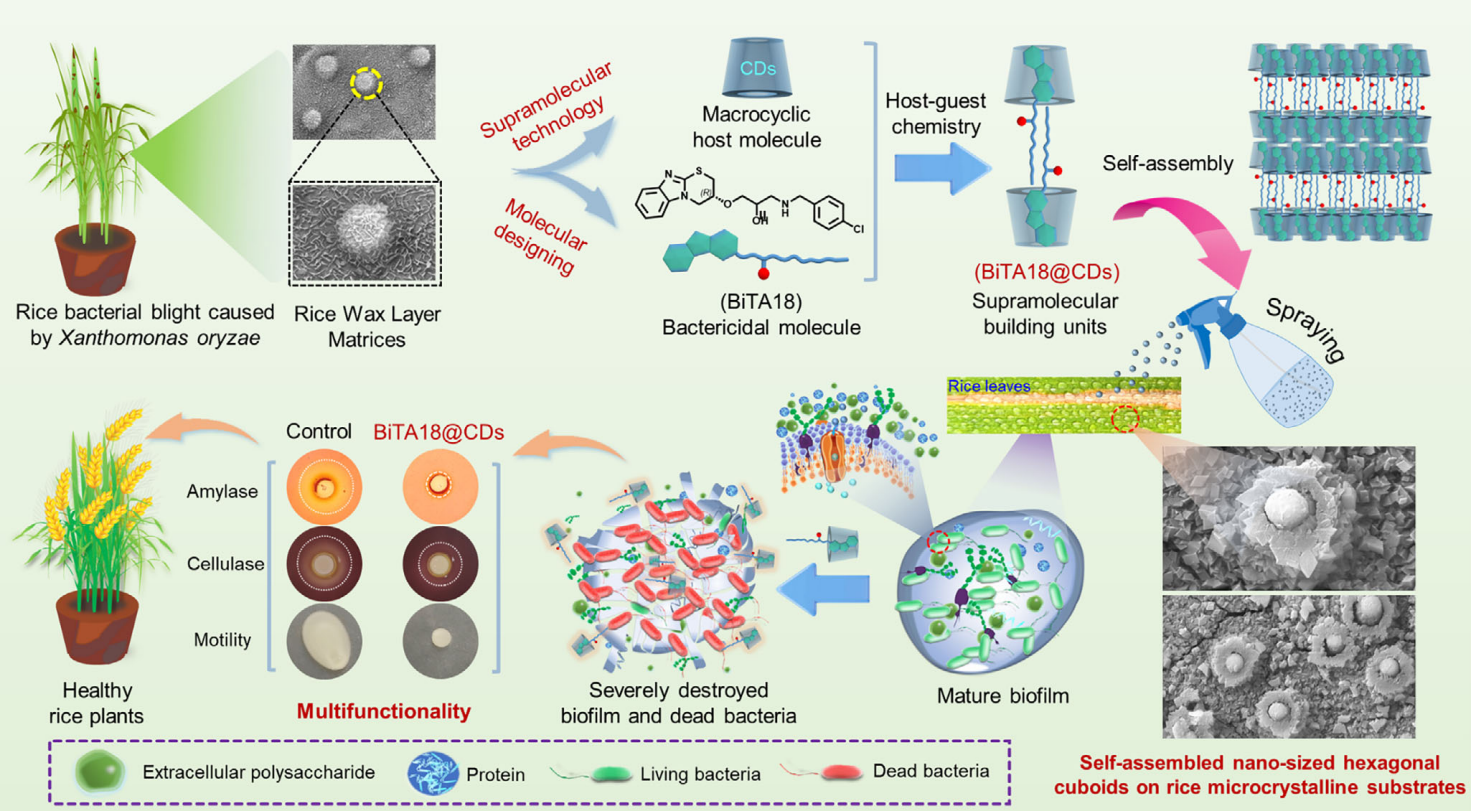
Schematic diagram for the development of advanced supramolecular nano‐aggregates assembled on rice wax layer matrices to promote the leaf deposition and bioavailability of bactericides for preventing plant bacterial infections.

According to the above‐mentioned design concept, a type of structurally novel isopropanolamine‐modified benzimidazole compounds was synthesized and biologically screened. As a result, a highly active bactericidal molecule (BiTA18) was selected as the guest molecule, subsequently creating two anisotropic supramolecular building units (BiTA18@*β*‐CD and BiTA18@*γ*‐CD) based on the host–guest complexation between BiTA18 and *β*‐/*γ*‐cyclodextrin (*β*‐CD/*γ*‐CD). More attractively, these building blocks can self‐assemble into nano‐sized hexagonal cuboids on the rice microcrystalline matrix, markedly enhancing the adsorption and retention of bactericidal ingredients on target plants. In addition, the constructed oligosaccharide‐coated supramolecular materials, with excellent biocompatibility, can radically destroy the bacterial biofilm barrier, limit bacterial motility, and induce electrolyte leakage and ROS (reactive oxygen species) accumulation in bacteria, ultimately eradicating the intractable pathogenic bacteria (**Scheme** **1**). In vivo study discloses that the conceived supramolecular materials are safe for target plants and non‐target organisms and have superior control efficacies than commercial agricultural bactericides. This study provides a crucial theoretical basis and new ideas for the development of environmentally friendly multifunctional supramolecular bactericides.

## 2 Results and Discussion

### 2.1 Rational Conception of Benzimidazole Derivatives (BiTA1‐BiTA26) and Their In Vitro Bactericidal Assays

**Scheme 2:**
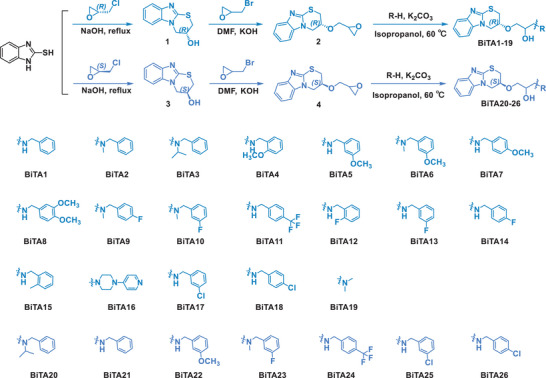
Synthetic procedure for target molecules of BiTA1‐BiTA26.

To potentially acquire a type of bioactive benzimidazole derivatives, a versatile isopropanolamine substructure, often appearing in the molecular composition of commercial microbicides, such as morinidazole and fluconazole,^[^ ^26^ ^]^ was merged into target compounds. A facile three‐step synthetic approach was raised and illustrated in **Scheme** **2**. First, starting from 2‐mercaptobenzimidazole (2‐MBI) and (*R*/*S*)‐(‐/+) epichlorohydrin, a substitution and cyclization reaction produced the intermediate 4‐hydroxybenzimidazole‐thiazine. Next, epibromohydrin substituted the hydrogen on the hydroxyl group, yielding a key intermediate owning an epoxide side chain. Finally, the target molecules (BiTA1‐BiTA26) were obtained via ring‐opening reactions with various primary or secondary amines. These structurally novel compounds were characterized by ^1^H‐NMR, ^13^C‐NMR, ^19^F‐NMR and HRMS (Figures S21–S114, Supporting Information), and then were screened for the bactericidal potency against *Xoo* by the frequently‐used turbidimetric method.^[^ ^27^ ^]^ For comparison, the existing agricultural bactericides thiodiazole‐copper (TC) and kasugamycin (KSM) were used as positive controls. The preliminary biological activity at 100 and 50 *µg* mL^−1^ are presented in Table S1 (Supporting Information). Notably, some of the designed target compounds displayed excellent bactericidal effects against pathogen *Xoo* at these two concentrations, exemplified by BiTA5, BiTA10, BiTA11, BiTA17‐18, and BiTA22‐26, achieving 100% inhibition, which were quite better than those of positive controls (TC, 27.0%; KSM, 72.9%; at 50 *µg* mL^−1^), 2‐MBI (starting reagent, 20.9% at 50 *µg* mL^−1^), and intermediates 1–4 (12.1–17.7% at 50 *µg* mL^−1^). This outcome preliminarily indicates that the introduction of isopropanolamine fragment by ring‐opening reaction in the target structure improves the bactericidal activity. Further screening of their EC_50_ values revealed that BiTA18 had the best activity (EC_50_ = 2.25 *µg* mL^−1^, **Table** **1**), far superior to TC (EC_50_ = 82.8 *µg* mL^−1^) and KSM (EC_50_ = 27.0 *µg* mL^−1^), suggesting the discovery of a highly effective benzimidazole‐based bactericidal agent.

**Table 1:** In vitro antibacterial activities of several target molecules against *Xoo*.

Compds	Regression equation	*R* ^2^	EC_50_ ^a)^ [µg mL^−1^]
BiTA5	*y* = 7.404x − 3.206	0.999	12.8 ± 0.4
BiTA10	*y* = 12.927x − 11.486	0.992	18.8 ± 0.5
BiTA11	*y* = 6.541x + 2.037	0.996	2.84 ± 0.08
BiTA17	*y* = 5.998x − 4.038	0.999	32.1 ± 0.1
BiTA18	*y* = 4.793x + 3.310	0.999	2.25 ± 0.05
BiTA22	*y* = 7.987x − 8.251	0.992	45.6 ± 1.0
BiTA23	*y* = 6.602x − 4.526	0.999	27.8 ± 0.2
BiTA24	*y* = 11.119x − 10.193	0.999	23.3 ± 0.1
BiTA25	*y* = 9.239x − 8.054	0.999	25.9 ± 0.8
BiTA26	*y* = 8.149x − 5.272	0.981	18.2 ± 0.2
TC^b)^	*y* = 2.797x − 0.365	0.958	82.8 ± 1.0
KSM^b)^	*y* = 2.567x + 1.326	0.930	27.0 ± 0.5

^a)^ The EC_50_ values for bactericidal activity are presented as the mean ± standard deviation (SD);

^b)^ The abbreviations TC and KSM represent thiodiazole‐copper and kasugamycin, respectively.

Based on the in vitro activity analysis of the target compounds against *Xoo* strains, we propose the following structure‐activity relationships. 1) The position of the halogen atom on the benzene ring significantly affects the biological activity. For example, the activity of the chlorine atom on the para‐position (4‐Cl, BiTA18, EC_50_ = 2.25 *µg* mL^−1^) markedly exceeds its meta‐position (3‐Cl, BiTA17, EC_50_ = 32.1 *µg* mL^−1^). 2) Compounds with non‐aromatic substituents or without substituents on the phenyl ring exhibit poor *anti‐Xoo* activity, such as BiTA1‐3 and BiTA19‐21 (showing activity <50% at 50 *µg* mL^−1^). 3) When a single electron‐donating group (─OCH_3_) is located at the meta‐position of the benzene ring, the anti*‐Xoo* activity increases, as observed with BiTA5 (100% activity at 50 *µg* mL^−1^, EC_50_ = 12.8 *µg* mL^−1^). However, when this substituent is located in other positions, lower activity is observed, as seen with BiTA4 and BiTA6‐8 (below 30% at 50 µg mL^−1^). 4) For the same substituent, the isopropyl ether group with *R*‐configuration shows better anti‐*Xoo* activity than the *S*‐configuration, exemplified by the following comparisons: BiTA5 (*R*‐type, 3‐OCH_3_, EC_50_ = 12.8 µg mL^−1^) vs. BiTA22 (*S*‐type, 3‐OCH_3_, EC_50_ = 45.6 µg mL^−1^), BiTA11 (*R*‐type, 4‐CF_3_, EC_50_ = 2.84 µg mL^−1^) vs. BiTA24 (*S*‐type, 4‐CF_3_, EC_50_ = 23.3 µg mL^−1^), and BiTA18 (*R*‐type, 4‐Cl, EC_50_ = 2.25 µg mL^−1^) vs. BiTA26 (*S*‐type, 4‐Cl, EC_50_ = 18.2 µg mL^−1^).

As a consequence, the optimal bactericidal molecule (BiTA18) with a 4‐chlorobenzylamine group was selected as the guest molecule. Considering its intrinsic hydrophobicity and poor water solubility, we will employ the eco‐friendly macrocyclic oligosaccharides (*β*‐CD/*γ*‐CD) to optimize its physicochemical property, water solubility, biocompatibility and biological functions by host–guest encapsulating strategy.

### 2.2 Preparation of Host–Guest Building Blocks (BiTA18@*β*‐CD and BiTA18@*γ*‐CD) and Their Self‐assembly Process

A straightforward and user‐friendly methodology was employed to prepare two host–guest building units (BiTA18@*β*‐CD and BiTA18@*γ*‐CD). Briefly, a tetrahydrofuran (THF) solution of the guest molecule BiTA18 (8.0 *µL*, 0.124 m) was gradually added dropwise into a 2.0 mL deionized water solution containing the host macromolecule *β*‐CD/*γ*‐CD (0.496 mm, molar ratio for BiTA18: *β*‐CD/*γ*‐CD was 1:1). After fully stirring and natural evaporation of THF, these two building blocks spontaneously self‐assembled into supramolecular aggregates. The correlative self‐assembly process, driving forces and topographic characteristics were investigated and discussed by ultraviolet titration, high‐resolution mass spectrometry (HRMS), Zeta potential, dynamic light scattering (DLS), ^1^H‐NMR titration, and scanning electron microscopy (SEM).

**Figure 1:**
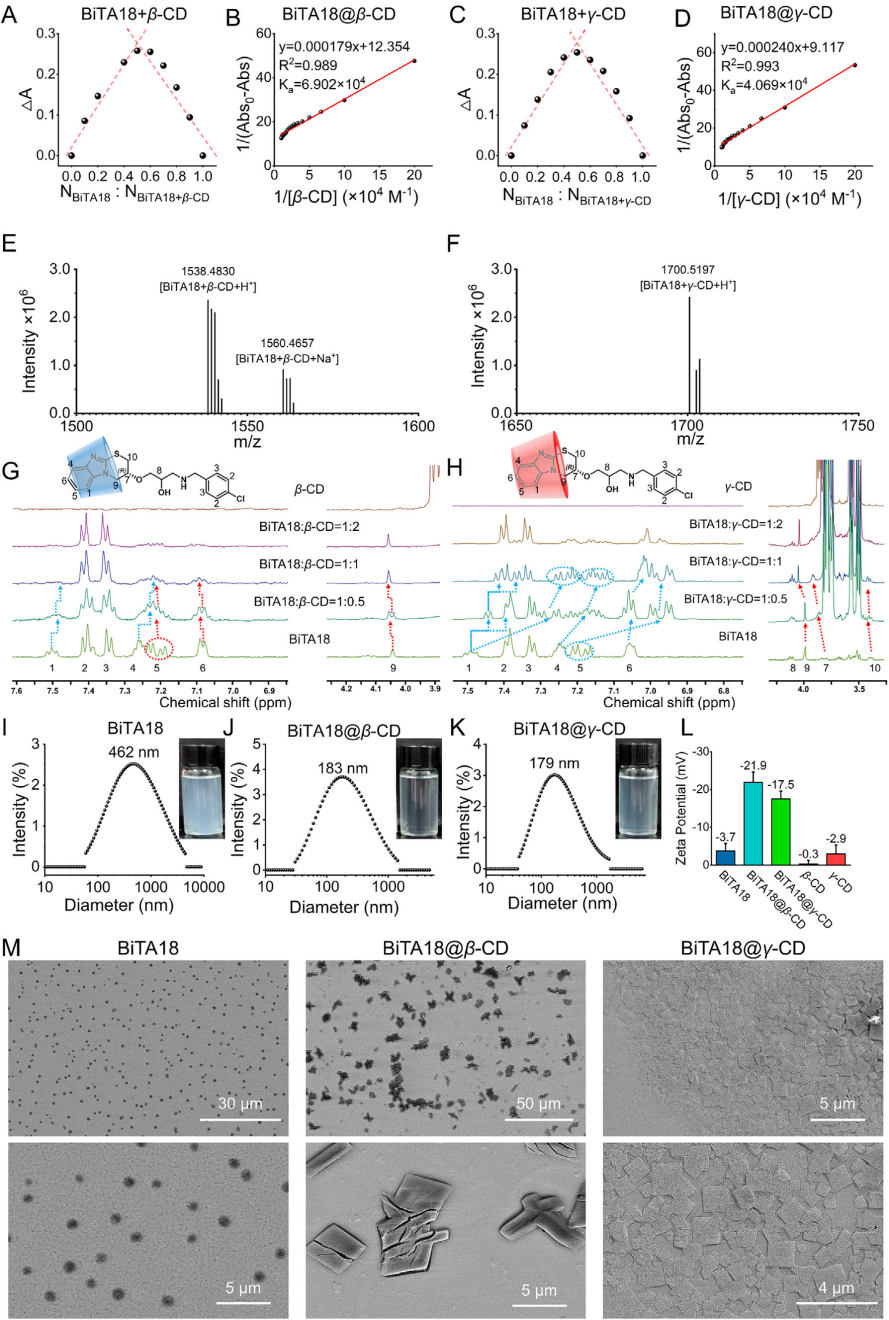
Preparation of host–guest supramolecular aggregates. A) Job's plots for the change in absorbance (ΔA) at 292 nm, generated from a 0.1 mm total concentration of BiTA8 and *β*‐CD in aqueous solution. B) Benesi‐Hildebrand equation and plots of colorimetric [*β*‐CD]^−1^ with (Abs_0_‐Abs)^−1^. C) Job's plots for the change in absorbance (ΔA) at 292 nm, based on a total concentration of 0.1 mm for BiTA18 and *γ*‐CD in aqueous solution. D) Benesi‐Hildebrand equation and plots of colorimetric [*γ*‐CD]^−1^ with (Abs_0_‐Abs)^−1^. E,F) HRMS analysis of BiTA18@*β*‐CD and BiTA18@*γ*‐CD. G‐H) ^1^H‐NMR titration spectra showing the interaction of BiTA18 with *β*‐CD or *γ*‐CD at molar ratios of 1:0.5, 1:1, and 1:2 in D_2_O. I–K) DLS measurements and corresponding images of BiTA18, BiTA18@*β*‐CD, and BiTA18@*γ*‐CD at a concentration of 200 µg mL^−1^ in aqueous solution with 0.4% DMSO. L) Zeta potential values for BiTA18, BiTA18@*β*‐CD, BiTA18@*γ*‐CD, *β*‐CD, and *γ*‐CD in water with 0.4% DMSO at a concentration of 200 µg mL^−1^. M) SEM images illustrating the morphology of BiTA18, BiTA18@*β*‐CD, and BiTA18@*γ*‐CD at a concentration of 200 µg mL^−1^.

First, the possible binding ratio was studied by their respective Job's plots curve. According to the achieved UV absorption spectra of *β*‐CD (or *γ*‐CD) and BiTA18 at different stoichiometric ratios (**Figure** **1**A,C), the UV absorbance intensity changes (ΔA) reached its maximum when N_BiTA18_: N_BiTA18+_ *_β_*_‐CD/_ *_γ_*_‐CD_ = 0.5, indicating that *β*‐CD/*γ*‐CD and BiTA18 probably combined together in a 1:1 stoichiometric ratio. Subsequent UV‐vis titration tests also confirmed this conclusion. When different equivalents of host molecules *β*‐CD or *γ*‐CD were added to a 0.1 mm aqueous solution of BiTA18, the absorbance of the system at 292 nm decreased, and this decrease continued until the host–guest binding ratio reached 1:1, at which point the trend leveled off (Figure S1, Supporting Information). Moreover, based on the fitting results using the Benesi‐Hildebrand equation, the binding constants for BiTA18@*β*‐CD and BiTA18@*γ*‐CD were calculated to be 6.902 × 10^4^ and 4.069 × 10^4^ m ^−1^, respectively, indicating that the host–guest binding in these constructed supramolecular building blocks is relatively stable (Figure 1B,D). Beyond that, HRMS analysis revealed that the relative molecular masses of [BiTA18+*β*‐CD+H^+^] and [BiTA18+*γ*‐CD+H^+^] were 1538.4830 (Figure 1E) and 1700.5197 (Figure 1F), respectively, confirming the successful formation of binary supramolecular complexes with a 1:1 binding ratio.

The host–guest recognition sites between BiTA18 and *β*‐CD/*γ*‐CD were probed by ^1^H NMR titration experiments. As illustrated in Figure 1G and Figure S2 (Supporting Information), upon the addition of 1.0 equivalent *β*‐CD, the protons from the benzimidazole ring become blunt peaks and give varying chemical shifts, probably indicating the location of encapsulation. For instance, protons H_1_ and H_4_ exhibited upfield shifts, showing Δ*δ* values of −0.02 and −0.03 ppm, respectively (Table S2, Supporting Information). By contrast, protons H_5_ and H_6_ moved to the low field direction with the Δ*δ* value of +0.02 ppm. Meanwhile, proton H_9_ adjacent to the benzimidazole ring also gave a downfield shift (Δ*δ* = +0.02 ppm). This outcome suggests that *β*‐CD mainly loads the middle portion (e.g. H_1_, H_4_ and imidazole ring) of benzimidazole ring and causes the shielding effect. At the same time, the protons H_5_, H_6_ and H_9_ are likely situated at the opening of the *β*‐CD, which could explain the observed deshielding effect. Careful observation found that protons H_2_ and H_3_ from the 4‐chlorobenzyl group had no chemical shift change, revealing that they were not the recognition and encapsulation sites. After loading BiTA18 with the larger macrocyclic oligosaccharide—*γ*‐CD at a molar ratio of 1:1, all the protons (H_1_, H_4_, H_5_ and H_6_) on the benzimidazole ring shifted to the high field with Δ*δ* values of ‐(0.09∼0.26), −0.10, −0.21, and −0.03 ppm, respectively (Figure 1H; Figure S3 and Table S3, Supporting Information), representing the probable recognition site. Meanwhile, protons H_7_, H_9_ and H_10_ exhibited downfield shifts, with Δ*δ* values of +0.10, +0.06, and +0.03 ppm, indicating their likely location at the openings of *γ*‐CD. Similarly, protons H_2_ and H_3_ from the 4‐chlorobenzyl group also did not give an obvious chemical shift, showing that this part was not the recognition site. Given the above inspections, the formation of host–guest binary supramolecular complexes (BiTA18@*β*‐CD and BiTA18@*γ*‐CD) were mainly through the selective partial or complete encapsulation of the benzimidazole fragment by *β*‐CD/*γ*‐CD.

DLS measurements demonstrated that the particle sizes of BiTA18, BiTA18@*β*‐CD, and BiTA18@*γ*‐CD at an effective concentration of 200 µg mL^−1^ were 462, 183, and 179 nm, respectively (Figure 1I–K). Concurrently, the turbidity of the solution was reduced after loading BiTA18 within the externally hydrophilic macrocyclic oligosaccharides (*β*‐CD or *γ*‐CD, inserted photographs), further illustrating that the current supramolecular optimization strategy can adjust the physicochemical properties, water solubility and biocompatibility of active ingredients. Next, the stability of these nanoparticles in aqueous solution was reflected by their Zeta potential measurements, providing the corresponding value of −3.7, −21.9, and −17.5 mV for BiTA18, BiTA18@*β*‐CD, and BiTA18@*γ*‐CD (Figure 1L). The increased absolute Zeta potential indicates that electrostatic repulsion is increased between the nanoparticles, which potentially promotes the stability of the system.^[^ ^28^ ^]^ To evaluate the stability of the supramolecular material BiTA18@*β*‐CD, we prepared an aqueous solution at 200 µg mL⁻¹ and monitored its particle size distribution and Zeta potential over time (0, 3, and 7 days). As shown in Figure S4 (Supporting Information), measurements taken at different storage intervals revealed no significant changes in either the primary particle size distribution or Zeta potential of the aggregates in solution. These results indicate that BiTA18@*β*‐CD exhibits good stability in aqueous solution. Finally, SEM imaging was used to reveal the morphological changes of BiTA18 during its transformation into supramolecular assemblies (Figure 1M). Notably, BiTA18 itself forms cotton cluster‐like aggregates in aqueous solution at 200 µg mL^−1^. Differently, BiTA18@*β*‐CD and BiTA18@*γ*‐CD afforded inhomogeneous flaky aggregates, reflecting the successful preparation of new supramolecular materials. The possible assembly mechanism is speculated as follows. For the BiTA18 itself, it contains a hydrophilic isopropanolamine part and hydrophobic 4‐chlorobenzyl and 3,4‐dihydro‐2*H*‐benzo[4,5]imidazo[2,1‐b][1,3]thiazine groups. When it aggregates in an aqueous solution, the hydrophilic part is more inclined to form hydrogen bond interaction with water molecules to protect the bulky hydrophobic part inside, thereby supplying spherical and cotton cluster‐like microstructures (Figure S5, Supporting Information). After coating the hydrophilic benzimidazole position of BiTA18 with externally hydrophilic CDs (*β*‐CD or *γ*‐CD), the hydrophilic‐hydrophobic balance of the whole molecule is remodeled and optimized, giving two host–guest building blocks BiTA18@*β*‐CD and BiTA18@*γ*‐CD. Given their anisotropic characteristics, the bulked hydrophilic cyclodextrin portion prefers to be exposed to the water environment and forms hydrogen bonds with water molecules, while the hydrophobic benzyl part will hide inside the assembly. Given this arrangement, the new supramolecular building units will align in a staggered configuration, optimizing the balance between hydrophilic and hydrophobic interactions, ultimately resulting in the formation of flaky aggregates (Scheme 1).

Given that the supramolecular host–guest complex combines the biocompatibility of cyclodextrin with an optimal hydrophilic‐hydrophobic balance, we propose that these flexible building blocks can be systematically organized on the hydrophobic rice wax layer, enhancing the effective deposition and bioavailability of BiTA18.

### 2.3 BiTA18@*β*‐CD/*γ*‐CD can Self‐Assemble into Nano‐Sized Hexagonal Cuboids on the Rice Microcrystalline Matrix, Realizing the Effective Leaf Retention and Deposition of Their Droplets

**Figure 2:**
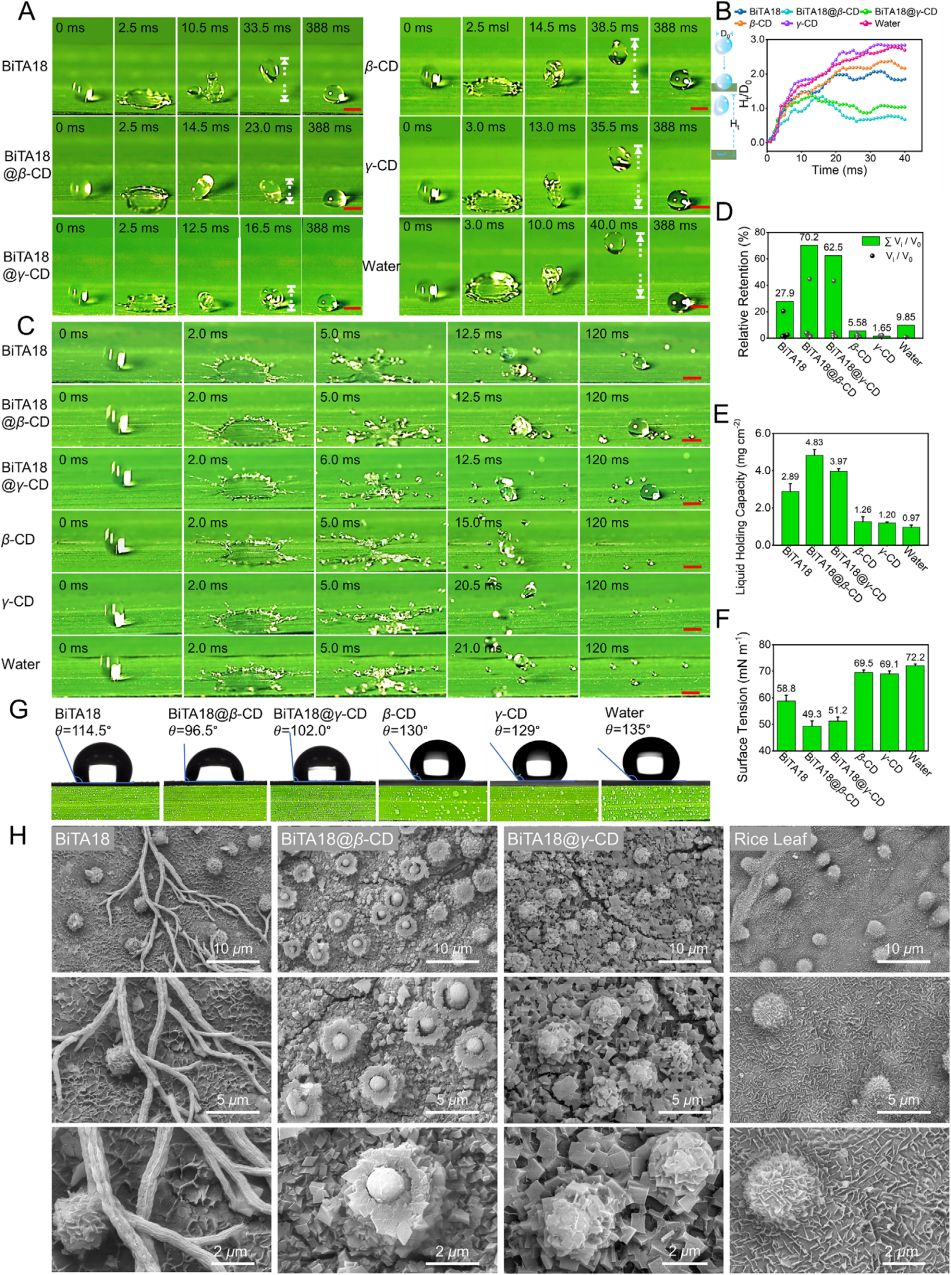
Droplet bounce/splash behaviors and self‐assembly of nano‐sized hexagonal cuboids on the rice microcrystalline matrix. A) The bouncing behavior of droplets containing BiTA18, BiTA18@*β*‐CD, BiTA18@*γ*‐CD, *β*‐CD, *γ*‐CD, and H_2_O on rice leaves from a height of 10 cm, with scale bars of 2 mm. B) Time‐resolved rebound height (H_t_/D_0_) as derived from Video S1 (Supporting Information), where D_0_ and H_t_ represent the initial droplet diameter and the height difference between the droplet tip and rice surface during shrinkage, respectively. C) The splashing behavior of BiTA18, BiTA18@*β*‐CD, BiTA18@*γ*‐CD, *β*‐CD, *γ*‐CD and H_2_O (30 cm height), scale bars: 2 mm. D) The volume of the normalized droplet finally staying on the rice leaves calculated according to Video S2 (Supporting Information), V_0_ and V_i_ represent the initial droplet volume and the volume of each droplet ultimately retained, respectively. E) Liquid holding capacity from the following experiment: immerse rice leaves with a diameter of 1.0 cm in various liquids for 30 s, then remove and weigh the amount of every group liquid retained on the leaves. F) The surface tension of BiTA18, BiTA18@*β*‐CD, BiTA18@*γ*‐CD, *β*‐CD, *γ*‐CD, and H_2_O. G) Contact angles of BiTA18, BiTA18@*β*‐CD, BiTA18@*γ*‐CD, *β*‐CD, *γ*‐CD, and H_2_O, and photographs for the sprayed droplets on rice leaves. H) SEM images of BiTA18, BiTA18@*β*‐CD, and BiTA18@*γ*‐CD deposition on rice leaves.

The key prerequisite for a good bactericide to achieve its efficacy is that it can remain on the target plants during foliar spraying. Given the optimized biocompatibility of the constructed supramolecular system, we carried out a series of experiments to examine its wettability, adhesion, and deposition on hydrophobic leaf surfaces. First, the droplet dynamic behavior recorded by a high‐speed camera was studied to evaluate the spreading and rebound performance of droplets on rice leaves. As illustrated in **Figure** **2**A and Video S1 (Supporting Information), the control droplets (H_2_O, *β*‐CD or *γ*‐CD) have significant rebound on rice leaves, while the supramolecular groups of BiTA18@*β*‐CD and BiTA18@*γ*‐CD can efficiently reduce droplet rebound, overtly better than the single BiTA18. Then, the bounce height of the above droplets was subsequently normalized (H_t_/D_0_, H_t_, and D_0_ represent the bounce height and the initial droplet diameter, respectively, Figure 2B). When the droplet reaches its maximum bounce height, the normalized heights of each component were 2.08 for BiTA18, 1.34 for BiTA18@*β*‐CD, 1.38 for BiTA18@*γ*‐CD, 2.37 for *β*‐CD, 2.86 for *γ*‐CD, and 2.78 for H_2_O, respectively. This finding discloses that the biocompatible BiTA18@*β*‐CD and BiTA18@*γ*‐CD droplets can markedly enhance the affinity to the hydrophobic rice wax layer matrices. Next, the droplet's splashing and splitting behavior as an important influencing factor of pesticide utilization was also investigated. As displayed in Figure 2C and Video S2 (Supporting Information), only a small amount of liquid was separated from the BiTA18@*β*‐CD and BiTA18@*γ*‐CD treated fractions during the high‐speed impact, and a large number of droplets eventually stayed on the leaf surface. The final retention rates of BiTA18@*β*‐CD and BiTA18@*γ*‐CD were 70.2% and 62.5%, respectively, which were markedly higher than those of BiTA18 and other control groups (Figure 2D). This outcome revealed that the current supramolecular system could effectively reduce the splash of droplets, make the majority deposited on the target crops, and ultimately increase the utilization rate of pesticides.

Liquid holding capacity (LHC) serves as another key parameter for assessing droplet retention.^[^ ^29^ ^]^ From Figure 2E, upon the treatment of BiTA18@*β*‐CD or BiTA18@*γ*‐CD on rice leaves, the corresponding LHC values were 4.83 and 3.97 mg cm^−3^, notably surpassing the single BiTA18 (2.89 mg cm^−3^) and other controls (H_2_O, *β*‐CD and *γ*‐CD, 0.97∼1.26 mg cm^−3^), indicating that the biocompatible supramolecular complexes prefer to attach to the rice wax layer matrices, demonstrating a clear advantage in droplet retention. To investigate the possible reasons for this case, their surface tension and contact angle measurements on hydrophobic rice leaves were performed and presented in Figure 2F,G. Clearly, the BiTA18@*β‐*CD droplet had the lowest surface tension (49.3 mN m^−1^) and the smallest contact angle (96.5°), indicating that this supramolecular system has the remarkable wetting and spreading properties on target plants, showing an enhanced droplet/leaf (liquid/solid) interfacial interaction. This effect is quite better than those of BiTA18 alone (58.8 mN m^−1^, 114.5°) and other controls (H_2_O, *β*‐CD and *γ*‐CD, 69.1∼72.2 mN m^−1^, 129°–135°). Moreover, the sprayed supramolecular droplets can be uniformly deposited on the surface of rice leaves compared with other components (Figure 2G, bottom). More importantly, to visualize the distribution of active ingredients on the leaf surface, the deposition morphology was observed using SEM in Figure 2H. Intriguingly, BiTA18@*β*‐CD and BiTA18@*γ*‐CD could self‐assemble into highly ordered nano‐sized hexagonal cuboids on the rice microcrystalline matrix. In particular, those supramolecular systems could also attach to the protuberance of rice leaves to form dense and regular architectures. This unique spectacle demonstrates that the reasonable construction of biocompatible supramolecular basic building blocks can promote their self‐assembly functions on hydrophobic substrates, which will be beneficial to fundamentally improve the deposition and bioavailability of pesticides. By contrast, BiTA18 itself with poor biocompatibility presented root‐like aggregates suspended on the surface of the leaves.

Based on the above investigations, the fabricated oligosaccharide‐coated supramolecular materials (BiTA18@*β*‐CD and BiTA18@*γ*‐CD) with good biocompatibility exhibit a variety of advantages in improving pesticide deposition, especially their self‐assembly properties on rice waxy layer substrates.

### 2.4 BiTA18@*β*‐CD and BiTA18@*γ*‐CD Efficiently Disrupt Biofilm Formation

**Figure 3:**
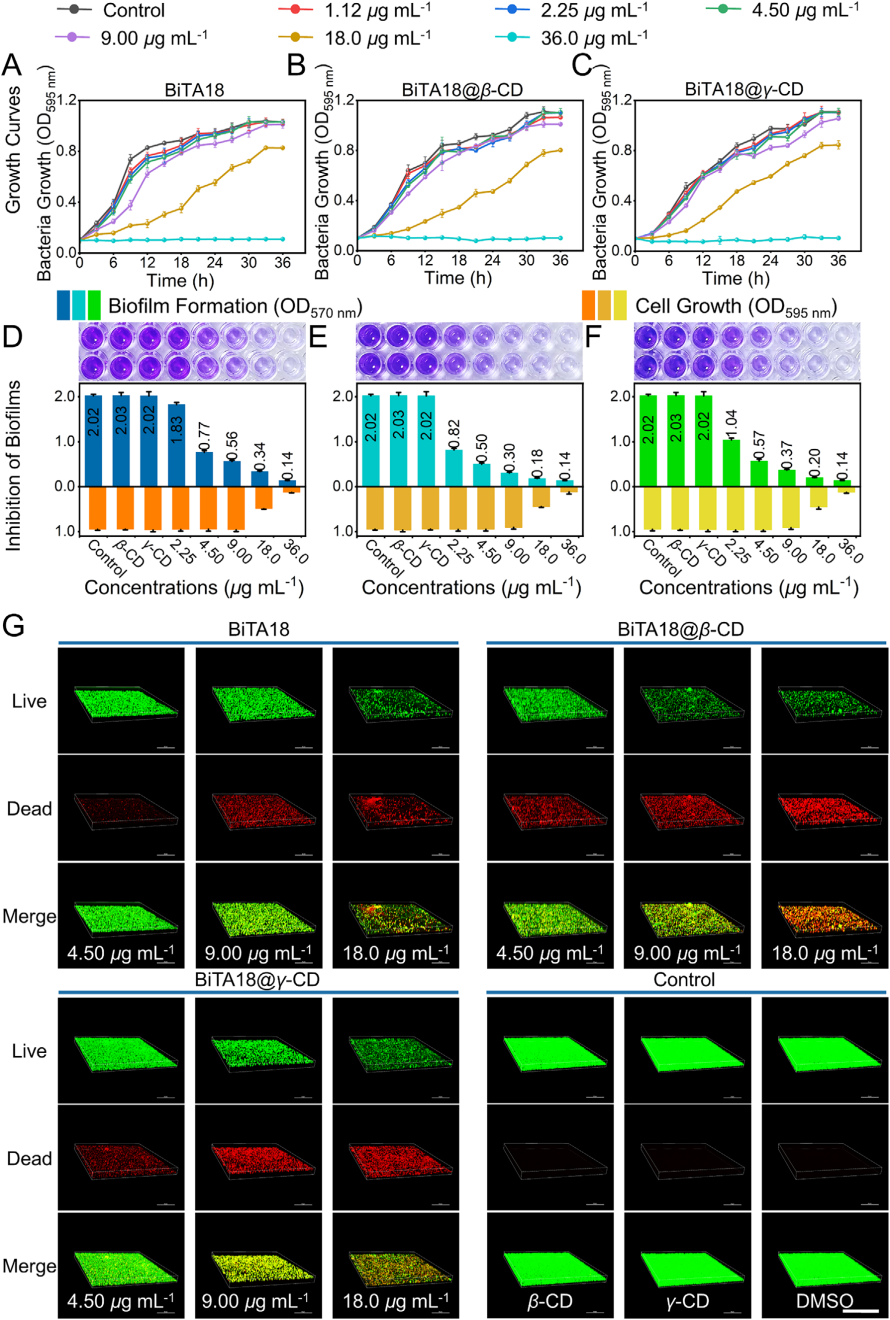
Supramolecular bactericides exhibit a stronger ability to inhibit biofilm formation. A–C) The growth curves of *Xoo* triggered by A) BiTA18, B) BiTA18@*β*‐CD, and C) BiTA18@*γ*‐CD at different dosages ranging from 1.12 to 36.0 µg mL^−1^ at an initial OD_595 nm_ value of 0.1. D–F) Quantification of *Xoo* biofilm formation through crystal violet staining at OD_570 nm_ and turbidimetric analysis of bacterial growth at OD_595 nm_ after co‐incubation with varying concentrations of D) BiTA18, E) BiTA18@*β*‐CD, and F) BiTA18@*γ*‐CD for 48 h. G) CLSM 3D images of *Xoo* cells within the biofilms formed after 48 h of treatment with BiTA18, BiTA18@*β*‐CD, and BiTA18@*γ*‐CD, stained with acridine orange (AO) and propidium iodide (PI), respectively. Scale bars: 100 *µm*.

Another key prerequisite for an excellent bactericide to achieve its function is to break through biofilm barriers. Therefore, inhibiting the growth/formation of bacterial biofilms in agriculture presents a significant challenge for the advancement of agrochemicals. In this section, the potential anti‐biofilm effects of BiTA18@*β*‐CD and BiTA18@*γ*‐CD were assessed using the widely employed crystal violet staining method.^[^ ^30^ ^]^ First, the growth curve of *Xoo* strain was determined when the initial OD_595 nm_ value was 0.1. As shown in **Figure** **3**A–C, under the condition of relatively sufficient bacteria, when adding different doses of components (BiTA18, BiTA18@*β*‐CD and BiTA18@*γ*‐CD), the growth of *Xoo* cells is not affected when the effective concentration is less than 9.0 µg mL^−1^. However, when the concentration exceeded 18.0 µg mL^−1^, the related bacterial growth began to be markedly inhibited. Based on this, *Xoo* cells (OD_595 nm_ = 0.1) were co‐cultured with varying concentrations of BiTA18, BiTA18@*β*‐CD, and BiTA18@*γ*‐CD for 48 h in 96‐well plates to quantify biofilm production using crystal violet staining. For comparison, *β*‐CD, *γ*‐CD, and 0.07% DMSO were used as control samples. As illustrated in Figure 3D–F, the color intensity of crystal violet solutions for *β*‐CD and *γ*‐CD was comparable to that of the solvent control (0.07% DMSO), indicating that they had no influence on biofilm formation. Attractively, at an extremely lower concentration of 2.25 µg mL^−1^, the constructed supramolecular materials (BiTA18@*β*‐CD and BiTA18@*γ*‐CD) presented noticeable inhibitory effects on the formation of biofilms, and the inhibition rates were 59.65% and 48.66%, respectively, which were quite better than that of BiTA18 (9.73%, Figure S6A—C, Supporting Information). This finding reveals that these oligosaccharide‐coated supramolecular inclusions may have higher biocompatibility and permeability to accomplish their anti‐biofilm functions. As the concentration of BiTA18@*β*‐CD was gradually increased to 4.5 and 9.0 µg mL^−1^, the formation of bacterial biofilms decreased by 75.27% and 84.99%, respectively, which were also superior to BiTA18 (62.20%/72.13%) and comparable to BiTA18@*γ*‐CD (71.89%/81.84%), confirming that loading BiTA18 with biocompatible *β*‐CD/*γ*‐CD can improve the biological properties of active ingredients, especially in terms of anti‐biofilm capability.

Considering its excellent anti‐biofilm and bactericidal abilities, we speculate that the bacteria encapsulated within the biofilm will be significantly reduced. After the supernatant was discarded, the bacteria within the biofilm left on the 96‐well plate substrate were cultured on agar solid medium to count the number of clones (Figure S6D, Supporting Information). Notably, with the increase of sample concentration, the number of bacteria within the biofilm presented a downward trend (Figure S6E—G, Supporting Information). For instance, at 2.25, 4.5, 9.0, 18, and 36 µg mL^−1^, the number of *Xoo* clones incubated with BiTA18@*β*‐CD were reduced by 38.67%, 58.01%, 77.17%, 87.82%, and 99.64%, respectively, which were higher than the individual BiTA18 (20.68%, 46.11%, 68.67%, 79.05%, and 90.69%), and similar to BiTA18@*γ*‐CD (35.72%, 50.49%, 75.29%, 85.14%, and 93.20%) at the corresponding same dose. This gratifying result discloses that BiTA18@*β*‐CD and BiTA18@*γ*‐CD are potent biofilm disruptors that can efficiently facilitate the bactericide/pathogen interactions, thereby leading to a substantial decrease in the number of bacteria hidden in the biofilm.

Beyond that, confocal laser scanning microscopy (CLSM) was employed to visually illustrate the density of biofilm formation and the survival of bacteria. In this experiment, acridine orange (AO) and propidium iodide (PI) were used to label living and dead bacteria, respectively.^[^ ^4c^ ^]^ As displayed in Figure 3G, regarding to the control groups (*β*‐CD, *γ*‐CD, and 0.04% DMSO), the green fluorescence channel gave a dense green coating, while no red spots appeared in the red fluorescence channel, indicating that the *Xoo* clusters within the biofilm barrier were growing well, with no death or inhibition observed. However, upon the treatment of BiTA18@*β*‐CD and BiTA18@*γ*‐CD, the density of biofilms gradually becomes thinner via a concentration‐dependent manner. Moreover, an obvious phenomenon was observed: as the sample concentration increased, the green spots gradually decreased, while the red spots increased (Figure S6H,I, Supporting Information). These outcomes reveal that BiTA18@*β*‐CD and BiTA18@*γ*‐CD have dual function in disrupting the formation of biofilms and annihilating the *Xoo* cells within the biofilms, which is consistent with the results of the above biofilm crystal violet staining and monoclonal culture experiments. Combined with the merged images, especially at 18 µg mL^−1^, we conclude that the supramolecular bactericide—BiTA18@*β*‐CD exhibited the optimal biological function, markedly surpassing the single BiTA18, which substantiates the practicability of the supramolecular optimization strategy.

### 2.5 BiTA18@*β*‐CD and BiTA18@*γ*‐CD can Eradicate Pre‐Established Biofilms

**Figure 4:**
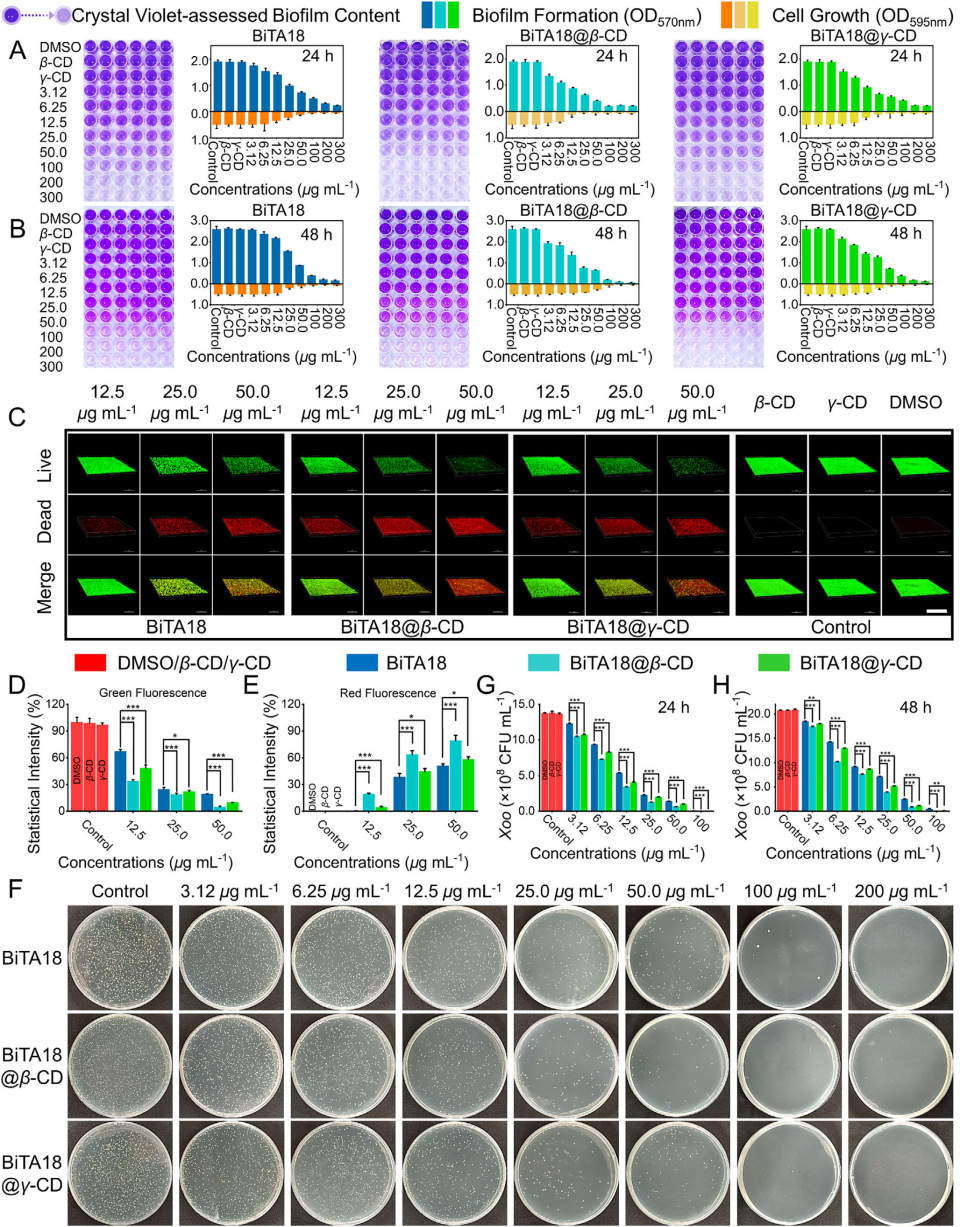
Supramolecular bactericides enhance the eradicating ability on pre‐established biofilms. The eradication efficacies of BiTA18, BiAT18@*β*‐CD, and BiTA18@*γ*‐CD on pre‐established biofilms aged for A) 24 h and B) 48 h. The biofilm contents were quantified using crystal violet staining at OD_570 nm_, while bacterial growth was assessed through turbidimetric analysis at OD_595 nm_. C) CLSM 3D images of *Xoo* biofilms aged for 24 h and subsequently treated with varying concentrations (12.5, 25.0, and 50.0 µg mL^−1^) of BiTA18, BiTA18@*β*‐CD, and BiTA18@*γ*‐CD. After treatment, *Xoo* cells within the biofilms were stained with AO and PI for imaging, with scale bars set at 100 *µm*. D‐E) Fluorescence statistics of live bacteria (D) and dead (E) bacteria from the CLSM image above. F) The colony‐culturing results for *Xoo* cells within the biofilm after treatment with different concentrations (0, 3.12, 6.25, 12.5, 25.0, 50.0, 100, and 200 µg mL^−1^) of BiTA18, BiAT18@*β*‐CD, and BiTA18@*γ*‐CD (Condition: *Xoo*‐biofilms were pre‐established for 24 h, and then treatment with bactericidal agents for 48 h). G,H) The number of bacterial clones within the biofilm counted from the agar plates (Condition: *Xoo*‐biofilms were pre‐established for 24 h (G) and 48 h (H), and treatment with bactericidal agents for 48 h). For (D,E, G,H), use one‐way analysis of variance (ANOVA) by least significant difference (LSD) multiple comparison test (For all studies, *n* ≥ 3, ns: no significant, *p < 0.05, **p < 0.01, ***p < 0.001) to determine significant differences.

In the middle and late stages of pathogen infection, mature biofilms are often established, so eradicating them is more challenging for bactericides. To test the scavenging ability of constructed supramolecular materials to mature biofilms, we pre‐established mature biofilms by ageing *Xoo* cells for 24 and 48 h, and then treated them with different doses of active ingredients for another 48 h before doing the crystal violet staining. From **Figure** **4**A,B, compared to the control samples (*β*‐CD, *γ*‐CD and 0.6% DMSO), the dark color intensity of crystal violet solutions gradually faded with increasing concentrations in the treatment groups, indicating that the pre‐established biofilms could be destroyed. Upon closer examination, it was found that BiTA18@*β*‐CD exhibited stronger disruptive activity than both BiTA18 and BiTA18@*γ*‐CD treatments. For example, regarding to biofilms aged for 24 h, upon the addition of a series of concentrations of BiTA18@*β*‐CD, the eradication rates were 41.61%, 53.43%, 66.45%, 78.37%, and 88.89% at 6.25, 12.5, 25.0, 50.0, and 100 µg mL^−1^, respectively, notably outperforming those of BiTA18 (19.10%, 26.29%, 48.18%, 62.13%, and 74.03%) and BiTA18@*γ*‐CD (32.23%, 51.67%, 65.28%, 70.16% and 77.79%) at the corresponding same dose (Figure S7A—C, Supporting Information). Similarly, for the biofilms aged for 48 h, BiTA18@*β*‐CD also parades the best scavenging ability on mature biofilms, comparing to the individual BiTA18 and BiTA18@*γ*‐CD. An example is as follows: at 25 µg mL^−1^, BiTA18@*β*‐CD, BiTA18@*γ*‐CD and BiTA18 provides the eradication rates of 71.07%, 51.16%, and 40.22%, respectively (Figure S7D—F, Supporting Information). These results suggest that the designed supramolecular bactericides, especially BiTA18@*β*‐CD, can serve as a potent biofilm‐disrupting agent.

Further, CLSM 3D imaging was carried out to visualize the eradicating effect on established biofilms. After the biofilm was pre‐incubated for 24 h, subsequent addition of different doses of supramolecular complexes resulted in different degrees of biofilm destruction. As presented in Figure 4C, from the effective concentration of 12.5 to 50 µg mL^−1^, compared with the control group, the density of biofilm in the treatment group was substantially reduced. Meanwhile, a phenomenon was observed that the green fluorescence intensity staining the living *Xoo* cells was gradually weakened (Figure 4D), while the red fluorescence intensity staining the deceased *Xoo* cells was strengthened (Figure 4E). This outcome reveals that our designed bactericides have dual function in destroying the mature biofilm barrier and annihilating the biofilm‐enclosed bacteria. It is worth noting that BiTA18@*β*‐CD has the strongest anti‐biofilm and bactericidal abilities by comparing the merged images. To concretely illustrate the bactericidal performance, the corresponding *Xoo* colonies within the biofilm in each treatment group were cultured on agar plates (Figure 4F). Clearly, the number of bacterial colonies decreased sharply with the increase of treatment concentration (Figure 4G). For instance, at 25 µg mL^−1^, the clone number for BiTA18, BiTA18@*β*‐CD and BiTA18@*γ*‐CD treatments was 2.27 × 10^8^, 1.28 × 10^8^, and 2.01 × 10^8^ CFU mL^−1^, respectively. Obviously, BiTA18@*β*‐CD paraded the optimal bactericidal effect with the fewest colony number at the same condition. More intriguingly, at 50 µg mL^−1^, BiTA18@*β*‐CD could result in a 95% reduction in the number of clones, demonstrating a potent bactericidal activity. Similar results were also observed in the pre‐formed biofilm for 48 h, subsequent BiTA18, BiTA18@*β*‐CD and BiTA18@*γ*‐CD treatments led to the significant disruption of both mature biofilms (Figure S8, Supporting Information) and bacterial reproduction (Figure 4H; Figure S9, Supporting Information). The order of their efficacies was as follows: BiTA18@*β*‐CD > BiTA18@*γ*‐CD > BiTA18, confirming the practicability of the supramolecular optimization strategy.

To evaluate the biofilm penetration capability of BiTA18@*β*‐CD and BiTA18@*γ*‐CD. First, the mature *Xoo* biofilms were pre‐formed in 6‐well plates, then 8.0 mL BiTA18@*β*‐CD and BiTA18@*γ*‐CD aqueous solution with a concentration of 9.0 *µ*g mL^−1^ were added into the above pre‐established biofilms to assess their entrance and absorption through HPLC determination. As shown in Figures S10–S12 (Supporting Information), compared to the biofilm‐free control, the concentrations of BiTA18@*β*‐CD and BiTA18@*γ*‐CD in the supernatant gradually decreased over time in the presence of *Xoo* biofilms. Notably, after 120 min, the peak area of BiTA18@*β*‐CD decreased by 89.4 (56.02%), while that of BiTA18@*γ*‐CD decreased by 71.9 (46.71%). These results demonstrate that both BiTA18@*β*‐CD and BiTA18@*γ*‐CD can penetrate bacterial biofilms, with BiTA18@*β*‐CD exhibiting significantly higher biofilm penetration efficiency than BiTA18@*γ*‐CD. This enhanced permeability directly improves bactericide‐pathogen interactions during biofilm removal. SEM‐assisted imaging was performed to further evaluate the biofilm disruption capability. As illustrated in Figure S13 (Supporting Information), the control group exhibited intact *Xoo* biofilms, with tightly packed bacterial cells. In contrast, treatment with BiTA18@*β*‐CD led to significant biofilm disruption in a concentration‐dependent manner, along with a gradual reduction in bacterial cells. Under the same concentrations, BiTA18@*γ*‐CD showed inferior efficacy. These results demonstrate that BiTA18@*β*‐CD is more effective than BiTA18@*γ*‐CD in eradicating mature biofilms.

The above experiments reveal that, compared to inhibiting biofilm formation, the effective concentration required to disrupt already‐formed biofilms is ≈5 times higher or more. This is because once the biofilm matures, its structure becomes more complex, and the extracellular polysaccharide (EPS) matrix becomes denser, which greatly reduces the permeability of antimicrobial agents. This highlights that in the early stages of pathogen infection, preventing biofilm formation is a more effective strategy, which will significantly alleviate the difficulty of disease control. Encouragingly, perhaps choosing the superior BiTA18@*β*‐CD is an alternative strategic decision.

### 2.6 Exploration of Bactericidal Mechanism of BiTA18@*β*‐CD and BiTA18@*γ*‐CD

**Figure 5:**
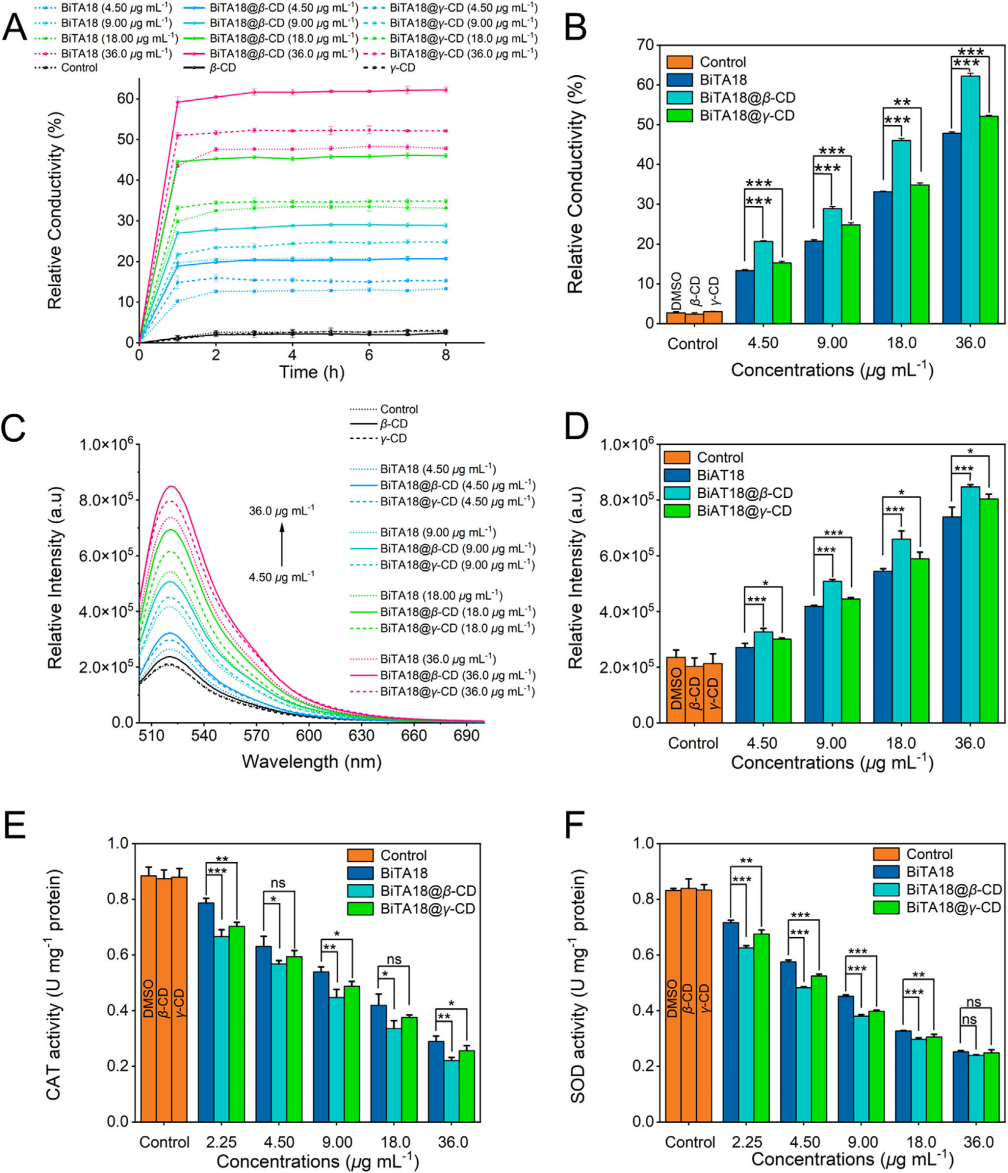
The possible bactericidal mechanism of supramolecular bactericides. A) The relative conductivity changes of *Xoo* cells upon the treatment with BiTA18, BiTA18@*β*‐CD, and BiTA18@*γ*‐CD for 0–8 h. B) The relative conductivity value of *Xoo* cells after being treated with BiTA18, BiTA18@*β*‐CD and BiTA18@*γ*‐CD for 8 h. C) The ROS contents in *Xoo* cells expressed by fluorescence emission after treatment with BiTA18, BiTA18@*β*‐CD and BiTA18@*γ*‐CD, Ex = 488 nm. D) Statistical diagram of fluorescence intensity at 520 nm for the indicative ROS contents. E‐F) CAT and SOD enzyme activities in *Xoo* cells after treatment with BiTA18, BiTA18@*β*‐CD and BiTA18@*γ*‐CD. For (B,D,E,F), use one‐way analysis of variance (ANOVA) by least significant difference (LSD) multiple comparison test (For all studies, *n* ≥ 3, ns: no significant, *p < 0.05, **p < 0.01, ***p < 0.001) to determine significant differences.

Changes in membrane permeability often affect a series of physiological response disorders.^[^ ^31^ ^]^ To clarify the characteristic of constructed supramolecular complexes, we employed indirect conductivity measurements to evaluate their influences on the membrane permeability. As illustrated in **Figure** **5**A,B, the relative conductivity had experienced significant changes after triggering by different concentrations of our designed bactericides, indicating that the membrane permeability is enhanced, which leads to the leakage of intracellular electrolytes. For example, after co‐incubation with *Xoo* cells at a concentration of 36 µg mL^−1^, the relative conductivity of BiTA18@*β*‐CD and BiTA18@*γ*‐CD reached 62.24% and 52.14%, respectively, exceeding that of BiTA18 (47.87%) and control samples (2.39–3.02% for *β*‐CD, *γ*‐CD and DMSO treatments). Clearly, the import of BiTA18@*β*‐CD can cause the most dramatic changes in membrane permeability, thereby potentially causing intracellular electrolyte leakage and physiological imbalances. Apart from that, oxidative stress, especially from excess ROS (reactive oxygen species), can lead to redox imbalance, DNA damage, and physiological dysfunction. Thus, we examined the ROS levels within bacteria across different treatment groups via commercial ROS kits (detection principle: a dye (6‐chloromethyl‐20,70‐dichlorodihydrofluorescein diacetate (CMH_2_DCFDA) will be activated by excessive ROS and emit green fluorescence). As presented in Figure 5C,D, the fluorescence intensity at 520 nm was increased in a dose‐dependent manner. At an effective concentration of 36 µg mL^−1^, the emission intensities of BiTA18@*β*‐CD and BiTA18@*γ*‐CD treatments were 8.47 × 10^5^ and 8.03 × 10^5^ a. u., respectively, markedly surpassing that of single BiTA18 (7.39 × 10^5^ a. u.). This intriguing finding reveals that BiTA18@*β*‐CD and BiTA18@*γ*‐CD can effectively provoke the accumulation of ROS contents within bacteria, intensifying oxidative stress and damage. On the other hand, some representative enzymes in microorganisms can automatically balance and remove excess peroxides, such as catalase (CAT) and superoxide dismutase (SOD). To delve into the possible reasons, the activity of these two enzymes in *Xoo* cells were tested by using corresponding commercial kits. As shown in Figure 5E,F, increasing concentrations of BiTA18@*β*‐CD, BiTA18@*γ*‐CD and BiTA18 led to a dose‐dependent decrease in CAT and SOD enzyme activities. Specifically, at 36 µg mL^−1^, the CAT activities of BiTA18@*β*‐CD and BiTA18@*γ*‐CD treatment groups were 0.22 and 0.26 U mg^−1^, respectively, which were lower than the 0.29 U mg^−1^ recorded for BiTA18; at 18 µg mL^−1^, the SOD activity (0.30 U mg^−1^) of BiTA18@*β*‐CD treatment was also lower than that of BiTA18 (0.33 U mg^−1^). These results demonstrate that BiTA18@*β*‐CD and BiTA18@*γ*‐CD can effectively weaken the bacterial antioxidant defense system. A possible bactericidal mechanism was concluded. After loading BiTA18 with cyclic macrocyclic oligosaccharides—*β*‐CD/*γ*‐CD, these carbohydrate‐coated supramolecular bactericides with good biocompatibility enhance the membrane permeability, thereby leading to electrolyte leakage. More seriously, upon the treatment of these designed bactericides, the bacterial redox system is disrupted and leads to the accumulation of excess ROS, which induces irreversible oxidative damage and eventually results in bacterial death.

### 2.7 The Potential Anti‐Biofilm Mechanism and Other Superior Biological Functions of BiAT18@*β*‐CD and BiTA18@*γ*‐CD in Reducing Bacterial Virulence

**Figure 6:**
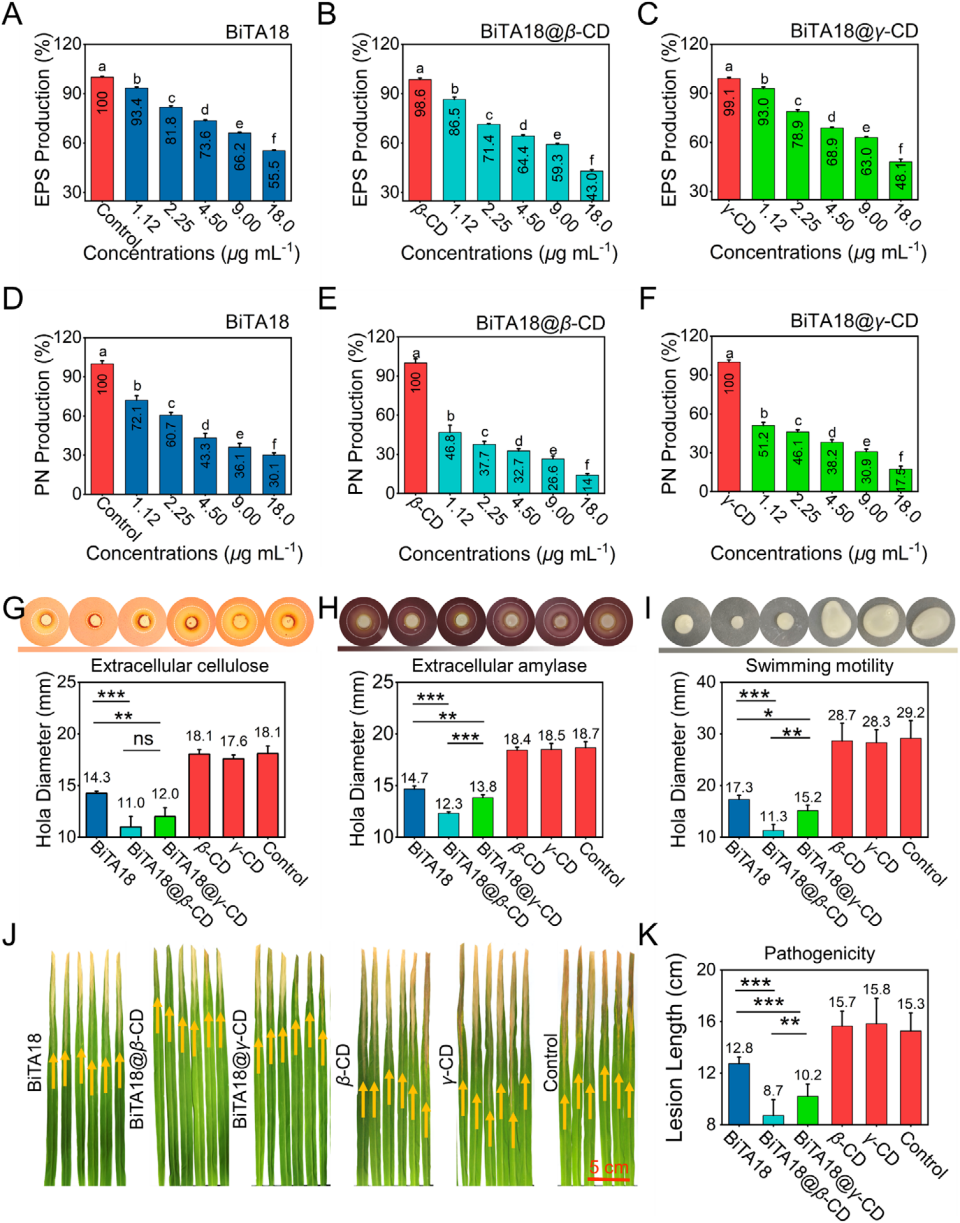
The anti‐biofilm mechanism and other superior biological functions of supramolecular bactericides. A–C) EPS production by *Xoo* cells following treatment with varying concentrations of BiTA18 (A), BiTA18@*β*‐CD (B), and BiTA18@*γ*‐CD (C). D–F) The effects of different doses of BiTA18 (D), BiAT18@*β*‐CD (E), and BiTA18@*γ*‐CD (F) on the production rate of extracellular *Xoo*‐proteins. G,H) The inhibitory effects of designed bactericides on the (G) cellulose and (H) amylase activity secreted by *Xoo* cells, the used concentration was 9.0 µg mL^−1^. I) The image of swimming motility assays and circle diameters statistical diagram at 9.0 µg mL^−1^. J,K) Pathogenicity assay of *Xoo*‐bacteria on rice leaves, the used concentration was 9.0 µg mL^−1^. For (A–F), different lowercase letters indicate statistically significant differences (For all studies, *n* ≥ 3, *p* < 0.05), as determined by one‐way ANOVA followed by Waller‐Duncan's post hoc test. For (G–I,K), use one‐way analysis of variance (ANOVA) by least significant difference (LSD) multiple comparison test (For all studies, *n* ≥ 3, ns: no significant, *p < 0.05, **p < 0.01, ***p < 0.001) to determine significant differences.

To preliminarily explore the anti‐biofilm mechanism, the influence of designed bactericides on the main components of biofilm was investigated. As presented in **Figure** **6**A–C, upon the addition of BiTA18, BiTA18@*β*‐CD, and BiTA18@*γ*‐CD, the extracellular polysaccharides (EPS) production decreased in a concentration‐dependent manner. At effective concentrations of 4.5, 9.0, and 18 µg mL^−1^, the EPS productions triggered by BiTA18@*β*‐CD were 64.4%, 59.3%, and 43.0%, respectively, which were lower than those of BiTA18 (73.6%, 66.2%, and 55.5%, respectively) and comparable to BiTA18@*γ*‐CD (68.9%, 63.0% and 48.1%, respectively). To investigate the underlying mechanism, we analyzed the transcriptional levels of the *gum* gene cluster, which regulates EPS synthesis and transport in *Xoo*, using qRT‐PCR. As shown in Figure S14 and Table S4 (Supporting Information), treatment with BiTA18, BiTA18@*β*‐CD, or BiTA18@*γ*‐CD (at 9.00 µg mL⁻¹) markedly downregulated the expression of *gumB*, *gumC*, *gumG*, *gumH*, *gumK*, and *gumM* compared to the control. Notably, BiTA18@*β*‐CD induced a more pronounced downregulation of *gum* genes than BiTA18 or BiTA18@*γ*‐CD, suggesting that its biofilm‐disrupting effect is likely due to its stronger suppression of EPS expression. A similar trend was observed in the impact on extracellular protein production. As the concentration of designed bactericides increased, the color of the protein solution gradually changed from blue to gray, indicating a significant reduction in protein contents (Figure S15, Supporting Information). At concentrations of 4.5 and 9.0 µg mL^−1^, comparing with negative controls (*β*‐CD, *γ*‐CD, and DMSO), the protein productions in BiTA18@*β*‐CD groups were 32.7% and 26.6% (Figure 6D–F), respectively, markedly less than those of BiTA18 (43.3% and 36.1%, respectively) and BiTA18@*γ*‐CD (38.2% and 30.9%, respectively), revealing the optimal biological activity of BiTA18@*β*‐CD. Based on the above investigations, the anti‐biofilm mechanism of designed bactericides may be attributed to their ability to efficiently disrupt the production of key components of biofilms.

When bacteria infect the host, they not only secrete a class of key extracellular enzymes that destroy plant tissue, such as cellulase and amylase, but also perform swimming to achieve large‐scale colonization and spread. The impact of designed bactericides on these virulence factors was evaluated. As illustrated in Figure 6G–I, BiTA18, BiTA18@*β*‐CD, and BiTA18@*γ*‐CD demonstrated excellent inhibitory effects on bacterial cellulose and amylase activities. Compared to the control group, which exhibited a degradation zone of 18.1 mm, the diameters for the three treatment groups were 14.3, 11.0, and 12.0 mm, respectively. Regarding the amylase activity, the degradation zones for control groups, BiTA18, BiTA18@*β*‐CD, and BiTA18@*γ*‐CD were 18.4∼18.7, 14.7, 12.3, and 13.8 mm, respectively. Clearly, BiTA18@*β*‐CD has the best inhibitory effect on extracellular enzyme activity, verifying the feasibility of supramolecular optimization technology in improving biological functions. Additionally, BiTA18@*β*‐CD could strongly hinder bacterial motility and provided a swimming diameter of 11.3 mm (Figure 6I), which was quite smaller than control groups (28.3–29.2 mm), BiTA18 (17.3 mm), and BiTA18@*γ*‐CD (15.2 mm). We speculate that these accessional superior properties in supramolecular bactericides will substantially reduce the pathogenicity of pathogens and alleviate the progression of bacterial disease. Therefore, the associated pathogenicity test was carried out in vivo. As presented in Figure 6J,K, upon the co‐incubation *Xoo* with designed bactericides, the corresponding disease symptoms were markedly relieved after inoculation of rice leaves, providing the average lesion lengths of 8.7 cm for BiTA18@*β*‐CD, 10.2 cm for BiTA18@*γ*‐CD, 12.8 cm for BiTA18, and 15.3–15.8 cm for control groups. Notably, the supramolecular system can effectively reduce the pathogenicity of *Xoo* to target crops by hindering various virulence factors, including the secretion of extracellular enzymes and bacterial motility, thereby hindering the spread of bacterial disease. Compared to BiTA18 alone, both BiTA18@*β*‐CD and BiTA18@*γ*‐CD showed enhanced biological functions, with BiTA18@*β*‐CD being particularly effective, highlighting the potential for plant disease control.

### 2.8 BiTA18@*β*‐CD and BiTA18@*γ*‐CD Have Good in vivo Control Efficacies Against Rice Bacterial Leaf Blight and Citrus Canker

**Figure 7:**
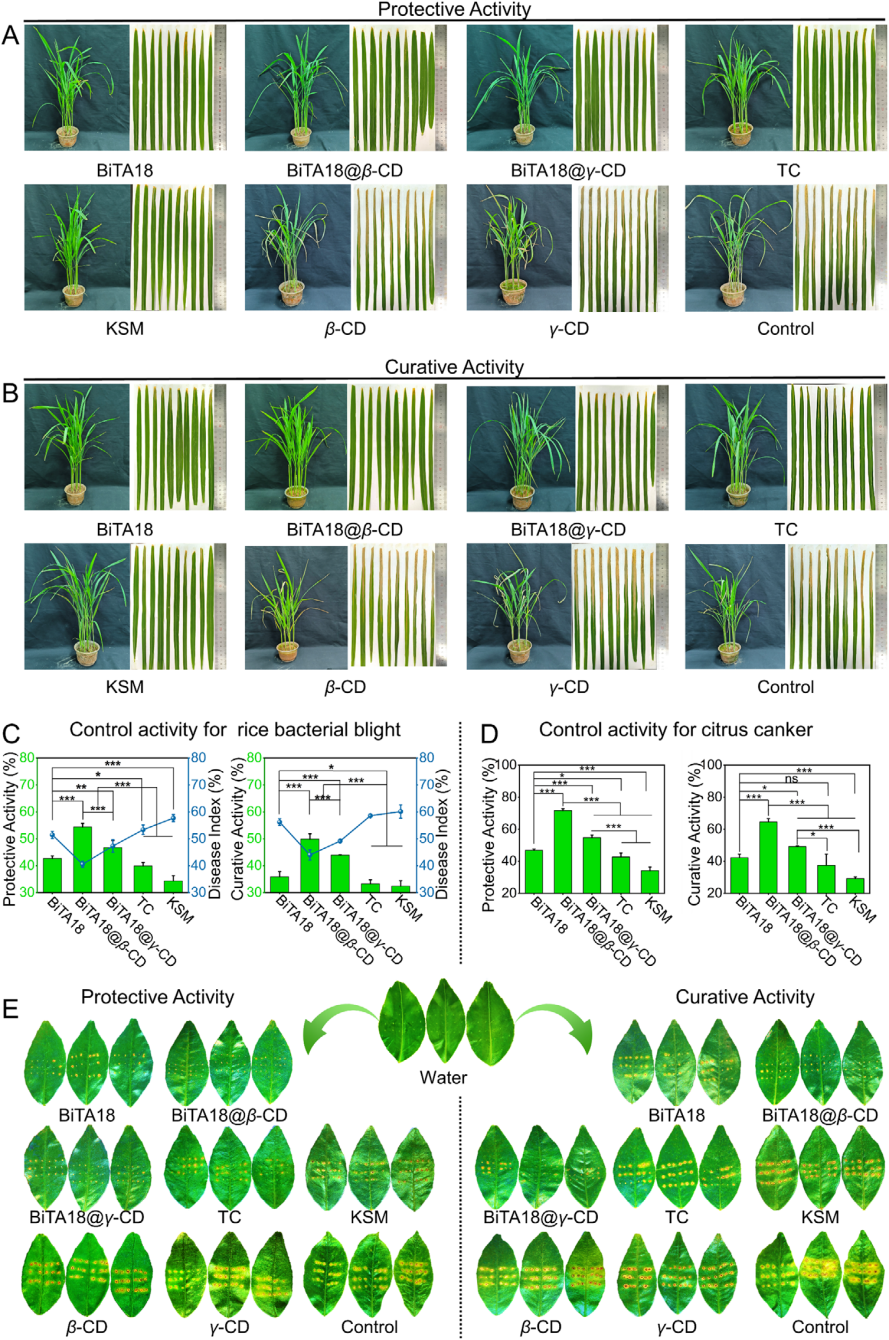
A,B) Photographs and disease symptoms showing the in vivo protective (A) and curative (B) effects of BiTA18, BiTA18@*β*‐CD, BiTA18@*γ*‐CD, and commercial bactericides (TC‐20%SC and KSM) against rice bacterial blight, administered at an effective dose of 200 µg mL^−1^, with rice plants continuously cultured for 14 days post‐spraying. C) The statistical charts for protective and curative activities as well as disease index against rice bacterial blight. D) The statistical charts for protective and curative activities against citrus canker at 200 µg mL^−1^. E) Photographs and disease symptoms for the in vivo protective and curative efficiencies of BiTA18, BiTA18@*β*‐CD, BiTA18@*γ*‐CD, TC‐20%SC and KSM against citrus canker at an effective dose of 200 µg mL^−1^ (continuously culturing citrus trees for 14 days after spraying). For (C‐D), use one‐way analysis of variance (ANOVA) by least significant difference (LSD) multiple comparison test (For all studies, *n* ≥ 3, ns: no significant, *p < 0.05, **p < 0.01, ***p < 0.001) to determine significant differences.

Given the excellent foliar deposition, anti‐biofilm, and other vibrant performance, these constructed bactericides were tested in vivo against *Xoo*‐induced rice bacterial blight by a standard leaf‐cutting method.^[^ ^32^ ^]^ For comparison, we set up positive controls (TC‐20%SC and KSM) and negative controls (0.4% DMSO, *β*‐CD, and *γ*‐CD). As illustrated in **Figure** **7**A–C, compared with negative controls, the application of designed bactericides remarkably alleviated the symptoms of bacterial disease. For the protective activity at 200 µg/mL, BiTA18@*β*‐CD provided a superior effectiveness of 54.4%, notably higher than those of BiTA18 (42.7%), commercial bactericides (TC‐20%SC, 39.9%; KSM, 34.3%) and BiTA18@*γ*‐CD (46.7%). Regarding the curative activity, BiTA18@*β*‐CD afforded the control efficacy of 49.9%, still outperforming the other treatment groups (BiTA18, 35.9%; TC‐20%SC, 33.3%; KSM, 32.4%; BiTA18@*γ*‐CD, 43.9%). These gratifying results reveal that the designed oligosaccharide—coated supramolecular bactericide (BiTA18@*β*‐CD) can serve as a promising new agrochemical for managing bacterial diseases.

To verify whether the designed bactericides have broad‐spectrum applicability against more bacterial diseases, we first evaluated the in vitro bioactivity against *Xanthomonas axonopodis* pv. *citri* (*Xac*), which could cause canker disease in the citrus industry.^[^ ^33^ ^]^ Intriguingly, BiTA18 also showed excellent in vitro anti‐*Xac* activity with EC_50_ value of 4.74 µg mL^−1^ (Tables S5 and S6, Supporting Information). Following this, the in vivo control efficacy of these designed bactericides against *Xac*‐induced citrus canker was evaluated on citrus plants. Notably, BiTA18@*β*‐CD exhibited excellent protective activity, reaching 71.7% at 200 µg mL^−1^, while BiTA18@*γ*‐CD, BiTA18, TC‐20%SC, and KSM were less effective to some extent, showing 54.7%, 46.9%, 42.7%, and 34.1%, respectively (Figure 7D,E). In terms of curative activity, BiTA18@*β*‐CD also demonstrated the optimal efficacy of 64.6%, surpassing those of BiTA18@*γ*‐CD (49.2%), BiTA18 (42.3%), TC‐20%SC (37.5%) and KSM (29.3%). Besides, we found that BiTA18@*β*‐CD gave a lower contact angle of 66° on citrus leaves, displaying good leaf wettability. This effect was better than those of H_2_O (91.5°), *β*‐CD (92°), *γ*‐CD (91.5°), BiTA18 (75.5°), and BiTA18@*γ*‐CD (69°) at the same condition (Figure S16A, Supporting Information). The following droplet sliding experiments demonstrated that BiTA18@*β*‐CD afforded the lowest sliding distance than other groups, indicating that its droplets are more willing to stay on the leaf surface (Figure S16B,C and Video S3, Supporting Information). Considering these excellent qualities, we believe that BiTA18@*β*‐CD can be used as a new generation of alternatives in the prevention of agricultural diseases.

### 2.9 The Designed Supramolecular Bactericides Had Good Biosafety

**Figure 8:**
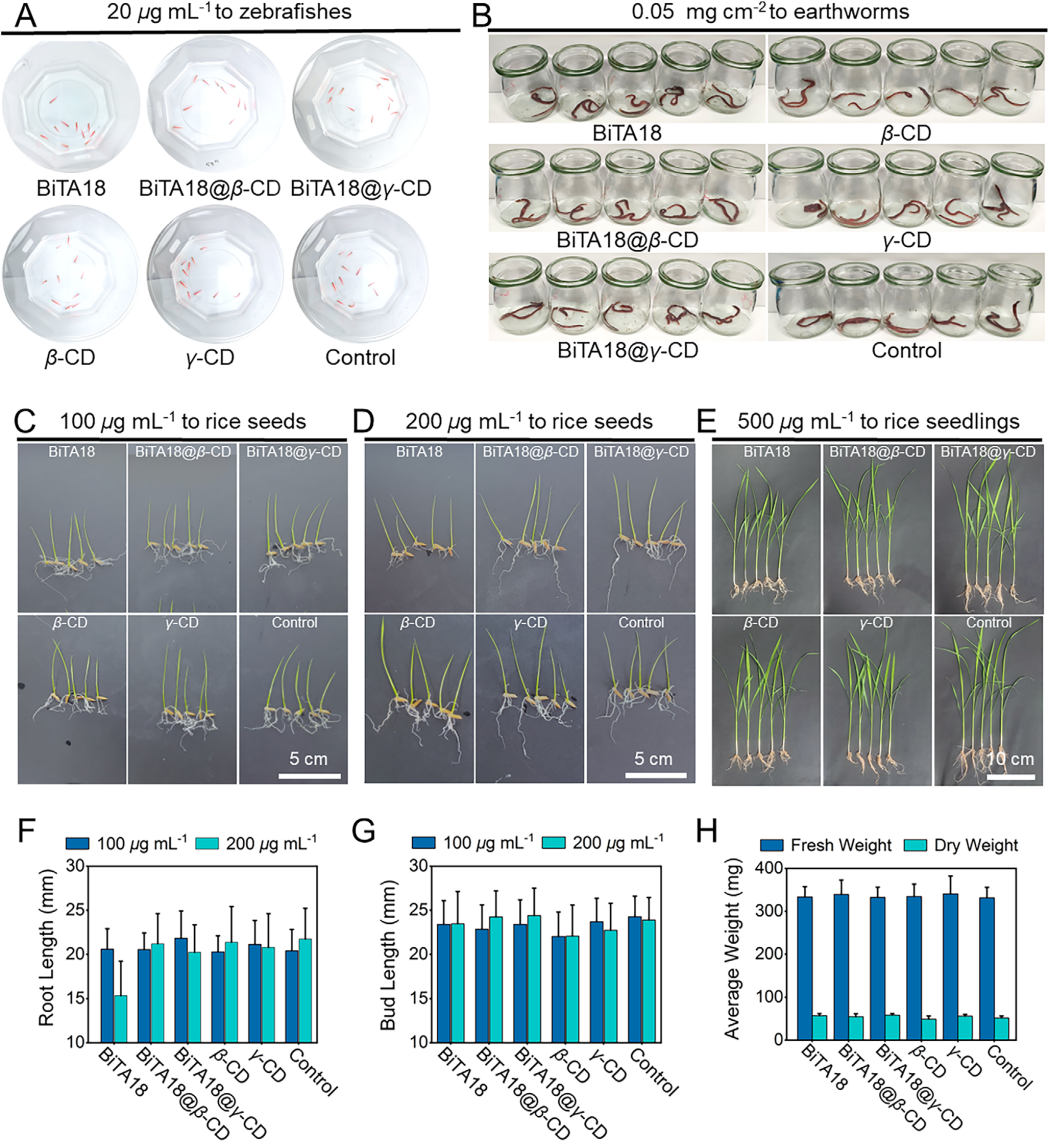
A,B) The acute toxicity test of BiTA18, BiTA18@*β*‐CD, and BiTA18@*γ*‐CD to zebrafishes at 20 µg mL^−1^ and earthworms at 0.05 mg cm^−2^. C,D) Photographs for the effect of BiTA18, BiTA18@*β*‐CD, and BiTA18@*γ*‐CD on the seed germination at the doses of 100 and 200 µg mL^−1^ after 7 days of cultivation. E) The effect of BiTA18, BiTA18@*β*‐CD, and BiTA18@*γ*‐CD on the growth of rice seedlings (initial stem length was 2–3 cm) at 500 µg mL^−1^ for an additional 14 days. F) Root and G) bud length statistics of rice seedlings treated with BiTA18, BiTA18@*β*‐CD, and BiTA18@*γ*‐CD at 100 and 200 µg mL^−1^ for 7 days. H) The fresh and dry weights of rice seedlings following 14 days of treatment with BiTA18, BiTA18@*β*‐CD, and BiTA18@*γ*‐CD at a concentration of 500 µg mL^−1^.

In line with the principles of low toxicity, sustainability, and environmental safety, this study further assessed the biotoxicity of BiTA18, BiTA18@*β*‐CD, and BiTA18@*γ*‐CD to non‐target organisms, such as zebrafish and earthworms, as well as target plants. In the zebrafish experiment, upon the treatment of designed bactericides at 20 µg mL^−1^ for a 96‐h period, all the groups remained active and exhibited no signs of toxicity over with a 100% survival rate (**Figure** **8**A; Figure S17A, Supporting Information). Similarly, a filter paper test at an effective concentration of 0.05 mg cm^−2^ demonstrated that all the treated earthworms were survival with no signs of toxicity over 72 h (Figure 8B; Figure S17B, Supporting Information). These outcomes indicate that the designed bactericides have low acute toxicity toward zebrafishes and earthworms. Next, the safety of these antimicrobial agents on rice seeds and their growth was evaluated. As shown in Figure S18 (Supporting Information), when the rice seeds were soaked at a concentration of 100 or 200 µg mL^−1^, the germination rate was not affected and could reach more than 95%. After 7 days of cultivation, the root and bud lengths of rice seedlings were measured (Figure 8C,D,F,G). At 100 µg mL^−1^, these indicators were not affected. At 200 µg mL^−1^, except for the monomer BiTA18 affecting root growth, other constituents had no effect on this index. To further assess long‐term effects, 20 rice seedlings treated with 500 µg mL^−1^ of the active components were cultivated for an additional 14 days. Then, the total length, fresh weight, and dry weight of the seedlings were measured, and no significant differences were observed (Figure 8E,H; Figure S19, Supporting Information). Additionally, no phytotoxic symptoms were detected on adult rice plants (Figure S20, Supporting Information).

Based on these comprehensive experiments, we conclude that BiTA18@*β*‐CD and BiTA18@*γ*‐CD are environmentally friendly, non‐toxic, and safe biocidal materials, posing no harm to either target or non‐target organisms. These properties highlight their potential for further commercialization and industrial‐scale production.

## 3 Conclusion

Inspired by the naturally hydrophobic wax layer microstructures in plants, we have developed two biocompatible host–guest building blocks—BiTA18@*β*‐CD and BiTA18@*γ*‐CD), which can self‐assemble into hierarchical nano‐sized hexagonal cuboids on the rice microcrystalline matrix. This outcome significantly enhances the retention and deposition of bactericidal ingredients on target plants, thereby solving the problem of pesticide off‐target. Interestingly, the formed oligosaccharide‐coated bactericides with good biocompatibility amplify the inhibition and eradication ability on bacterial biofilms. Meanwhile, the optimal supramolecular bactericide—BiTA18@*β*‐CD can not only efficiently hinder the bacterial motility and extracellular enzyme secretion, but also induce electrolyte leakage and ROS accumulation in bacteria, which eventually damages the bacteria and leads to bacterial death. In vivo experiments, BiTA18@*β*‐CD shows broad‐spectrum and efficient control efficiencies of 54.4% and 71.7% against rice bacterial blight and citrus bacterial canker, respectively, which are better than those of thiodiazole‐copper‐20%SC (39.9%/42.7%), kasugamycin (34.3%/34.1%) and BiTA18 (42.7%/46.9%) at 200 µg mL^−1^. More importantly, these carbohydrate‐coated bactericides are eco‐friendly and biosafe to target plants and non‐target organisms. This study offers a solid foundation for building multifunctional supramolecular bactericides that can self‐assemble on hydrophobic leaf microcrystalline substrates to enhance the bioavailability of pesticides.

## Conflict of Interest

The authors declare no conflict of interest.

## Supporting information



Supporting Information

Supplemental Video 1

Supplemental Video 2

Supplemental Video 3

## Data Availability

The data that support the findings of this study are available in the supplementary material of this article.
