# Supplementary material for: Assembling Hexahedral Supramolecular Nano‐Aggregates on Rice Wax Layer Matrices to Promote the Leaf Deposition and Bioavailability of Bactericides for Plant Protections
Source: Adv Sci (Weinh). 2025 May 28;12(30):e04225. doi: 10.1002/advs.202504225 (PMC12376559; doi:10.1002/advs.202504225)
Supplement: Supplementary file 1 — Supporting Information [file ADVS-12-e04225-s001.docx]

Supporting Information

**Assembling Hexahedral Supramolecular Nano-Aggregates on Rice Wax Layer Matrices to Promote the Leaf Deposition and Bioavailability of Bactericides for Plant Protections**

*Run Yang**^†^, Jinghan Yang^†^, Min Liu, Juan Liu, Peiyi Wang**

R. Yang, J.-H. Yang, M. Liu, J. Liu, Prof. P.-Y. Wang

State Key Laboratory of Green Pesticide, Key Laboratory of Green Pesticide and Agricultural Bioengineering, Ministry of Education, Center for Research and Development of Fine Chemicals of Guizhou University, Guiyang, 550025, China.

* Corresponding author E-mail: pywang@gzu.edu.cn; pywang888@126.com (P.-Y. Wang).

^†^ The two authors contribute equally to this work.

**Contents**

- 1. Experimental Section 4

1.1 Chemicals and Instruments 4

1.2 Bacterial Strains and Plant Varieties 5

1.3 Synthesis of Target Compounds 5

1.4 Antibacterial Bioactivity *In Vitro* Research 7

1.5 Job's Plot Experiment 8

1.6 UV-vis Titration Experiment 8

1.7 HRMS of BiTA18@*β*-CD and BiTA18@*γ*-CD 9

1.8 ^1^H NMR Titration Experiment 9

1.9 DLS and Zeta Potential Determination 9

1.10 SEM View Morphology 9

1.11 Bounce and Splash Behavior of Droplets on Rice Leaves 10

1.12 Liquid Holding Capacity Assay 10

1.13 Determination of Surface Tension and Contact Angles 10

1.14 Bacterial Growth Curve Determination 11

1.15 Crystal Violet Staining Experiment 11

1.16 Confocal Laser Scanning Microscopy (CLSM) 3D Imaging 12

1.17 Bacterial Plate Monoclonal Experiment 13

1.18 Measurement of Conductivity 13

1.19 Measurement of ROS Levels 14

1.20 Activity Assays of CAT and SOD Enzymes 14

1.21 Measurement of Extracellular Polysaccharide 15

1.22 Measurement of Protein Content 15

1.23 Measurement of Cellulase Inhibition 16

1.24 Measurement of Amylase Inhibition 16

1.25 Swimming Motility Assay 17

1.26 Pathogenicity Analysis 17

1.27 *In Vivo* Activity Test against Rice Bacterial Blight 18

1.28 *In Vivo* Activity Test against Citrus Canker 18

1.29 Sliding Behavior of Droplets on Citrus Leaves 20

1.30 Toxicity of Compounds on Non-target Organisms 20

1.31 Toxicity of Compounds to Rice Organisms 21

1.32 Statistical Analysis 22

- 2. Supplementary Figures and Tables 22

2.1 *In Vitro* anti-*Xoo* Activity of Title Compounds 22

2.2 UV-vis Titration Curves 23

2.3 ^1^H NMR Titration Analysis of Supramolecular Complexes 24

2.4 Particle Size Distributions and Zeta Potential Values at Different Storage Periods 25

2.5 UV-vis Absorption Spectra and Self-assembly Diagram of BiTA18 25

2.6 Biofilm Inhibition Assays 26

2.7 Crystal Violet Staining for Biofilm Eradication 27

2.8 CLSM Images Show the Eradication of Biofilms 28

2.9 The Plate Monoclonal Experiment from Biofilm Eradication 29

2.10 The Biofilm Penetrability of BiTA18@CDs 29

2.11 SEM Images of *Xoo* Biofilms Triggered by BiTA18@CDs 31

2.12 The Transcriptional Level of the Interrelated *Gum* Gene Cluster in *Xoo* 32

2.13 Measurement of Extracellular *Xoo*-Proteins Inhibition 33

2.14 *In vitro* anti-*Xac* Activity of Title Compounds 34

2.15 Interaction of Compounds with the Leaf Surface of Citrus 35

2.16 Acute Toxicity Experiment on Zebrafish and Earthworms 36

2.17 Rice Toxicity Experiment 36

- 3. ^1^H NMR, ^13^C NMR, ^19^F NMR and HRMS Analysis 37
- 4. ^1^H NMR, ^13^C NMR, and HRMS Spectra 51
- References 99

# 1. Experimental Section

## 1.1 Chemicals and Instruments

Chemicals. *β*-cyclodextrin (99%), *γ*-cyclodextrin (97%), 2-mercaptobenzimidazole (2-MBI, 98%), (R)-(-)-Epichlorohydrin (98%), (S)-(+)-Epichlorohydrin (98%), Epibromohydrin (97%), Sodium hydroxide (NaOH), Potassium carbonate (K_2_CO_3_), 2-Propanol (IPA), various primary or secondary amine (98%), NaCl, Iodine (I_2_), Potassium iodide (KI) was purchased from China. The DMSO-*d*_6_, CDCl_3_ and D_2_O used in ^1^H NMR, ^13^C NMR and ^19^F NMR were purchased from China. The methanol and acetonitrile of mass spectrometry grade used in MS were purchased from China. The DMSO used in the UV-vis spectrum was derived from China. The crystal violet, 3,6-bis (dimethyl amino) acridine (AO), Propidium iodide (PI), carboxymethyl cellulose (CMC), soluble starch and Congo red were purchased from China. The NB culture medium contains 1.0 g/L yeast powder (China), 3.0 g/L beef extract (China), 5.0 g/L peptone (China), and 10 g/L glucose (China) and NB solid culture medium was added 15 g/L agar (China) on the basis of NB culture medium.

Instruments. ^1^H-NMR, ^13^C-NMR and ^19^F-NMR spectra were achieved from the JEOL-ECX500 instrument or a Bruker Biospin-AG-400 apparatus. The mass spectra were obtained by a high-resolution mass spectrometer (UitiMate 3000, Thermo Scientific). Ultraviolet−visible (UV−vis) spectra were acquired using a UV–2335 spectrophotometer (Uniko (Shanghai, China) Instrument Co., LTD). Contact angles were tested by a JC-2-2000D1 apparatus (Shanghai Zhongchen Digital Technic Apparatus Co., Ltd., Shanghai, China). SEM (scanning electron microscope) images were visualized using FEI Nova. The molecular Zeta potential was tested by the analyzer (DelsaNanoC, Beckman. Coulter, Inc., USA). The OD value of *in vitro* antibacterial activity was monitored by using Cytation™ 5 multimode readers (BioTek Instruments, Inc. USA). Impact experiments were obtained from the i-SPEED 220 high-speed camera. Impact experiments were obtained from the C FOR Nikon Cyclona-2-2000-C high-speed camera. Nikon A1R Confocal Microscope System (Nikon Instruments Inc., Melville, New York, USA) was used for 3D imaging.

## 1.2 Bacterial Strains and Plant Varieties

Bacterial strains. The bacterial strains used in this study included *Xanthomonas oryzae* pv. *oryzae* (*Xoo*), and *Xanthomonas axonopodis* pv. *citri* (*Xac*). The two strains were obtained from Center for Research and Development of Fine Chemicals of Guizhou University and maintained in 80% sterile glycerol at -80 ℃.

Plant varieties. The rice plant (Fengyou Xiangzhan) and citrus plant (Fertile orange) were used as test plants. The two test plants were purchased from China commercial sources.

## 1.3 Synthesis of Target Compounds

1.3.1 Synthesis of Intermediates 1 and 3

2-MBI (1000 mg, 6.52 mmol) and NaOH (417.6 mg, 10.44 mmol) were placed in a round-bottom flask containing 30 mL anhydrous ethanol, stirring and dissolving, (R)-(−)-Epichlorohydrin or (S)-(+)-Epichlorohydrin (510.29 *μ*L, 6.52 mmol) was added and refluxed at 79 °C for 5.0 h with stirring. After that, the purified intermediate 1 or 3 were obtained by filtration and washing with anhydrous ethanol and water. For intermediate 1 (yield, 94.37%, Character, white powder). ^1^H NMR (400 MHz, DMSO-*d*_6_) *δ* 7.50 – 7.38 (m, 2H, Phenyl-H), 7.18 – 7.10 (m, 2H, Phenyl-H), 5.78 (s, 1H, -OH), 4.47 (dq, *J =* 6.1, 3.1 Hz, 1H, CH-OH), 4.23 (dd, *J =* 12.7, 3.6 Hz, 1H, N-CH_2_), 4.08 (dd, *J =* 12.6, 5.6 Hz, 1H, N-CH_2_), 3.40 (dd, *J =* 12.7, 2.5 Hz, 1H, S-CH_2_), 3.21 (dd, *J =* 12.5, 6.9 Hz, 1H, S-CH_2_). ^13^C NMR (101 MHz, DMSO-*d*_6_) *δ* 146.3, 142.8, 136.0, 121.8, 120.8, 117.0, 108.74, 60.6, 48.2, 31.4.

1.3.2 Synthesis of Intermediates 2 and 4

The intermediate 1 or 3 (1.0 g, 4.84 mmol), KOH (544.02 mg, 9.7 mmol), and DMF (15 mL) were mixed and stirred for 15 min, then epibromohydrin (625 *μ*L, 7.76 mmol) was added and reacted at room temperature for 5.0 h. The reaction system was extracted with saturated NH_4_Cl and ethyl acetate, the organic phase was retained and the preliminary purified intermediate 3 was obtained by vacuum distillation. Column chromatography (The eluent is V_dichloromethane_ : V_methanol_ = 30 : 1.0) to further obtain the final purified intermediate 2 or 4. For intermediate 2 (yield, 78.3%, properties, white powder). ^1^H NMR (500 MHz, Chloroform-*d*) *δ* 7.61 (dt, *J =* 6.7, 1.1 Hz, 1H, Phenyl-H), 7.25 – 7.17 (m, 3H, Phenyl-H), 4.41 – 4.32 (m, 1H, CH_2_-CH**-** O (CH_2_)), 4.32 – 4.25 (m, 1H, OCH-CH_2_–N), 4.19 – 4.11 (m, 1H, OCH-CH_2_–N), 4.05 (ddd, *J =* 38.6, 11.8, 2.3 Hz, 1H, O-CH_2_), 3.53 (ddd, *J =* 61.4, 11.9, 6.2 Hz, 1H, O-CH_2_), 3.39 – 3.27 (m, 2H, OCH-CH_2_**-**S), 3.20 (dtd, *J =* 10.8, 6.7, 2.5 Hz, 1H, OCH_2_-CH-O), 2.86 – 2.83 (m, 1H, OCH-CH_2_-O), 2.67 – 2.64 (m, 1H, OCH-CH_2_-O). ^13^C NMR (126 MHz, Chloroform-*d*) *δ* 143.6, 135.9, 122.7, 121.7, 118.4, 118.4, 108.0, 107.9, 103.1, 70.7, 70.4, 70.2, 51.0, 50.9, 47.1, 46.6, 44.3, 44.2, 29.4, 28.9.

1.3.3 Synthesis of Title Compounds BiTA1-BiTA26

The intermediate 3 or 4 (300 mg, 1.14 mmol) was dissolved in 10 mL isopropanol solution, followed by the addition of amine compound (1.72 mmol), and the reaction was carried out at 60 °C for 4.0 h. The reaction system was extracted with water and dichloromethane, and the organic layer was retained and dried by vacuum distillation. Further, the purified target compound was obtained by column chromatography (The eluent was V_dichloromethane_:V_methanol_ = 10 : 1.0). All these structures were detected by NMR and HRMS spectra.

## 1.4 Antibacterial Bioactivity *In Vitro* Research

The turbidimetric method was used to evaluate the antimicrobial action of compounds BiTA1-BiTA26 against *Xanthomonas* (*Xoo* and *Xac*)^[1]^. In brief, BiTA1-BiTA26 were dissolved in DMSO to gain a final concentration of 50 mg mL^-1^, then 40 *μ*L of the prepared solution was dissolved in 4.0 mL of 1.0 ‰ Tween 20 aqueous solution, shaken well, and 1.0 mL of the configured aqueous solution was added to a test tube containing 4.0 mL of NB liquid medium (3.0 g beef extract, 5.0 g peptone, 1.0 g yeast powder, 10.0 g glucose, and 1.0 L redistilled water; PH = 7.2) to get these compounds concentration was 100 *μ*g mL^-1^. The compounds medium was prepared downward according to the half-fold dilution method, then 40 *μ*L *Xoo* or *Xac* bacterial suspension (OD_595 nm_ = 0.6) was added. The inoculated test tubes were placed in a shaker and cultured for 24 - 48 h (220 rpm, 28 ± 1.0 ℃), the OD_595 nm_ value of 200 *μ*L bacterial suspension which was transferred to 96-well plates was measured with an enzyme calibration to evaluate the in vitro antibacterial activity of the compounds. DMSO was used as a negative control and TC-20%SC and KSM were used as positive control. The experiments were conducted with the establishment of three parallel groups for each trial.

All test samples were monitored at OD _(turbidity-corrected values)_ = OD_bacterial wilt_ – OD_no bacterial wilt_, and the inhibition rate I was calculated by

$$\text{I = }\frac{\left( \text{C - T} \right)}{\text{C}}\text{×100\%}$$

C is the corrected turbidity values of bacterial growth on untreated NB (negative control), and T is the corrected turbidity values of bacterial growth on treated NB. Compounds concentration was converted to log (x), and inhibition rate data was converted to odds (y). The toxicity regression equation (y = ax + b) and correlation coefficient (R^2^) were obtained using Excel calculations to determine the inhibitory semi-inhibitory concentration value (EC_50_).

## 1.5 Job's Plot Experiment

The mixed solution of host and guest with a total concentration of 0.1 mM (the ratio of host: guest = 0:10, 1:9, 2:8, 3:7, 4:6, 5:5, 6:4, 7:3, 8:2, 9:1, 10:0) was prepared. The absorbance difference of each sample was measured. Job's curve was generated by plotting ΔA against N_BiTA18_ : N_BiTA18 +_ *_β_*_-CD_ (molar ratio) or ΔA against N_BiTA18_ : N_BiTA18 +_ *_γ_*_-CD_ scatter plots at 292 nm.

$$\text{∆A=}\text{(Abs}_{\text{0}} \text{-}\text{ Abs}_{\text{i}}\text{)}*\text{a}$$

Abs_0_ is the absorbance when the molar ratio of the guest molecule to the total molecule is 100%, Abs_i_ is the absorbance of guest molecules at different molar ratios, a is the molar ratio of the guest molecule to the total.

## 1.6 UV-vis Titration Experiment

The concentration titration curves of compound BiTA18 at different concentrations (dissolved in 3.0 mL water) were determined in a 3.0 mL cuvette. Based on this, 0.1 mM guest molecule was selected as the test concentration. A 10 mM aqueous solution of *β*-CD and *γ*-CD was prepared and added proportionally to the colorimetric dish for the complex titration curve test. The binding constant (*K*_a_) between the host (*β*-CD or *γ*-CD) and guest molecules was determined by the Benesi-Hildebrand (B-H) formula:

$$\frac{\text{1}}{\text{Abs}_{\text{i}} \text{- }\text{Abs}_{\text{0}}}\text{ = }\frac{\text{1}}{\text{Abs}_{\text{∞}} \text{- }\text{Abs}_{\text{0}}}\text{ + }\frac{\text{1}}{\left( \text{Abs}_{\text{∞}} \text{- }\text{Abs}_{\text{0}} \right)\left[ \text{L} \right]\text{K}_{\text{a}}}$$

Abs_i_ is the absorbance measured experimentally, Abs_0_ is the absorbance of the guest molecule, Abs_∞_ is the maximum absorbance, [L] is the real-time concentration of the host molecule, K_a_ is the binding constant of host-guest molecules. To obtain *K*_a_, plot 1/(Abs - Abs_0_) against 1/[L] and calculate the slope (1/(Abs_∞_ - Abs)[L]) and intercept (1/(Abs_∞_ - Abs)) of the resulting straight line.

## 1.7 HRMS of BiTA18@*β*-CD and BiTA18@*γ*-CD

BiTA18 of 5.0 mg was accurately weighed, and *β*-CD or *γ*-CD with a molar ratio of 1:1 were added, respectively. The mixed solution was passed through the filter membrane (0.22 *μ*m), and the high-resolution mass spectrum of the BiTA18@*β*-CD and BiTA18@*γ*-CD were obtained by a high-resolution mass spectrometer (UitiMate 3000, Thermo Scientific).

## 1.8 ^1^H NMR Titration Experiment

Accurately calculate and weigh 4.0 mmol of BiTA18, add 490 *μ*L of deuterated water and 25 *μ*L of deuterated DMSO to dissolve it, following, 2.0, 4.0, 8.0 mmol of *β*-CD or *γ*-CD were added, respectively, and perform BiTA18@*β*-CD and BiTA18@*γ*-CD nuclear magnetic titration characterization by JEOL-ECX600 instrument.

## 1.9 DLS and Zeta Potential Determination

*β*-CD, *γ*-CD, BiTA18, BiTA18@*β*-CD and BiTA18@*γ*-CD were dissolved in water, respectively, to achieve the final concentration was 200 *μ*g mL^-1^. Zeta potential and average hydrodynamic diameters were determined by dynamic light scattering instrument.

## 1.10 SEM View Morphology

200 *µ*g mL^-1^ of BiTA18, BiTA18@*β*-CD and BiTA18@*γ*-CD were dropped onto the electroconductive glass. After natural drying, a layer of gold was sprayed on it, and the morphology of samples was photographed by SEM. For observing the deposition effect of the compounds on rice leaf, 30 *μ*L of pre-configured BiTA18, BiTA18@*β*-CD, and BiTA18@*γ*-CD at 200 *μ*g mL^-1^, which were carefully dropped on rice leaf or citrus leaf, dried naturally and sprayed with gold. Their deposition effects and morphology on the leaf finally were captured by SEM.

## 1.11 Bounce and Splash Behavior of Droplets on Rice Leaves

According to the reported methods.^[2]^ The i-SPEED 220 high-speed cameras (iX Cameras) has been used to record the micro-process of water droplets impacting rice leaves at a speed of 3000 fps, BiTA18, BiTA18@*β*-CD, BiTA18@*γ*-CD, *β*-CD, *γ*-CD aqueous solutions at 200 *μ*g mL^-1^ and H_2_O were punched out through 0.33 mm × 1.3 mm needles, respectively. The parameters are obtained by analyzing the video using i-SPEED Suite software. The droplet heights in the splash and bounce experiments are 30 cm and 10 cm, respectively.

## 1.12 Liquid Holding Capacity Assay

According to the reported methods,^[3]^ rice leaves were cut into circular slices equal to 1.0 cm in diameter and then immersed in a compound-containing aqueous solution at a concentration of 200 *μ*g mL^-1^. Take it out after 30 s to allow the water droplets on the leaves to slide off naturally, and the mass of leaves was weighed using an analytical balance. The liquid holding capacity (LHC) was calculated using the following equation:

$$\text{L}\text{HC}\text{=}\frac{\text{M}_{\text{1}}\text{-}\text{M}_{\text{0}}}{\text{S}}$$

where M_0_ and M_1_ represent the weights of the leaves before and after soaking, and S is the surface area of the leaves.

## 1.13 Determination of Surface Tension and Contact Angles

Surface tension and contact angles of six components (BiTA18, BiTA18@*β*-CD, BiTA18@*γ*-CD, *β*-CD, and *γ*-CD, 0.4% DMSO) were measured on a JC-2-2000D1 apparatus (Shanghai Zhongchen Digital Technic Apparatus Co., Ltd., Shanghai, China). The surface tension and contact angle were measured by the angle method and the hanging drop method, respectively.

## 1.14 Bacterial Growth Curve Determination

*Xoo*-bacteria were cultured to the logarithmic growth phase (OD_595 nm_ = 0.6), and the turbidity (OD_595 nm_) was adjusted back to 0.1 with NB liquid medium. Following, divided it into test tubes (1.0 mL bacterial suspension per test tube), and different doses of BiTA18, BiTA18@*β*-CD and BiTA18@*γ*-CD were added to make the final concentrations of 1.12 *μ*g mL^-1^ (0.5×EC_50_), 2.25 *μ*g mL^-1^ (1.0×EC_50_), 4.5 *μ*g mL^-1^ (2.0×EC_50_), 9.0 *μ*g mL^-1^ (4.0×EC_50_), 18 *μ*g mL^-1^ (8.0×EC_50_), 36 *μ*g mL^-1^ (16×EC_50_), respectively. 0.07% DMSO was used as a negative control, and the experiment included three parallel groups. The tubes were then incubated at 28 ± 1.0 °C and 220 rpm in a shaker. Next, the turbidity (OD_595 nm_) of the bacterial suspension was tested by the microplate reader per 3 hours, the experiment was carried out for 36 h. At this time, the turbidity (OD_595 nm_) value of the negative control group tends to be gentle.

## 1.15 Crystal Violet Staining Experiment

For biofilm inhibition experiment,^[4]^ *Xoo* suspension (OD_595 nm_ = 0.1) of 200 *μ*L was added to the 96-cell culture plate, and different doses of BiTA18, BiTA18@*β*-CD, and BiTA18@*γ*-CD were added by half dilution method to make the final concentration of 2.25, 4.5, 9.0, 18, 36 *μ*g mL^-1^, respectively. For comparison, *β*-CD, *γ*-CD and 0.07% DMSO were used as control samples. After 48 h of incubation in a constant temperature incubator (28 °C), the upper bacterial suspension was carefully sucked away, and the sterile PBS buffer solution (PH = 7.4, 10 mM, 200 *μ*L) was carefully washed 2 - 3 times to remove the residual planktonic bacteria. After natural drying, 0.1% crystal violet dye solution (200 *μ*L) was added to each hole to stain for 30 min. Next, the crystal violet staining solution on the upper layer was slightly sucked away, the crystal violet staining solution that was not combined with the biofilm was carefully washed 2 ~ 3 times with PBS buffer solution again. The plate was then dried in an oven at 40 ℃ for 1.0 ~ 2.0 h, and the crystal violet was completely dissolved with 200 mL 95% ethanol solution. Finally, the relative content of biofilm was measured by measuring OD_570 nm_ using an enzyme labeling instrument.

For biofilm eradication experiment,^[5]^ a total of 200 *μ*L of bacterial suspension (OD_595 nm_ = 0.1) was added to a 96-cell culture plate and cultured for 24 h or 48 h. The upper bacterial suspension was then carefully sucked away, and the biofilm attached to the 96-cell culture plate was washed with 200 *μ*L PBS once to remove potentially attached planktonic bacteria. 200 *μ*L of NB liquid medium containing BiTA18, BiTA18@*β*-CD, BiTA18@*γ*-CD were added to each well (the final concentrations were 300, 200, 100, 50, 25, 12.5, 6.25, 3.12 *μ*g mL^-1^, respectively, *β*-CD, *γ*-CD and 0.6% DMSO were used as control samples. Continued to incubate for 48 h, and then crystal violet staining was performed (the crystal violet staining method is the same as described above).

## 1.16 Confocal Laser Scanning Microscopy (CLSM) 3D Imaging

In advance, the conductive glass (10×10×1.1 mm, 10 Ω) was sterilized with high temperature and placed in a sterile 6-well bacterial culture plate.

For biofilm inhibition, NB medium containing *Xoo*-bacteria (OD_595 nm_ = 0.1) and BiTA18, BiTA18@*β*-CD, BiTA18@*γ*-CD (the final concentrations were 4.5 *μ*g mL^-1^ (2.0×EC_50_), 9.0 *μ*g mL^-1^ (4.0 ×EC_50_), 18 *μ*g mL^-1^ (8.0×EC_50_), respectively) were added to the above-mentioned pre-prepared 6-well cell culture plate. After aging for 48 h, the upper medium and planktonic bacteria were discarded, and the formed biofilm was washed twice with PBS. The living and dead bacteria were stained with acridine orange (AO) and propidium iodide (PI), and fixed by baking staining. Finally, CLSM 3D imaging was performed.

For biofilm eradication,^[6]^ we pre-established mature biofilms by ageing *Xoo* cells for 24 and 48 hours, and then treated them with different doses of active ingredients (BiTA18, BiTA18@*β*-CD, and BiTA18@*γ*-CD at 50, 25, and 12.5 *μ*g mL^-1^, respectively) for another 48 hours in 6 cell culture plates. Then, the upper planktonic bacteria and culture medium were discarded, the formed biofilm was washed twice with PBS. The living and dead bacteria were stained with acridine orange (AO) and propidium iodide (PI), and fixed by baking staining. Finally, CLSM 3D imaging was performed.

## 1.17 Bacterial Plate Monoclonal Experiment

The bacterial suspension was cultured according to section 1.15 above. After diluting the bacterial suspension by 10,000 times, 10 *μ*L was absorbed and spread evenly over NA solid medium (100 mL NB liquid medium with 1.5 g agarose powder). After 5-6 days of incubation, the number of bacteria was measured by Image J software. The experiment was set up with three parallel controls.

## 1.18 Measurement of Conductivity

Incubate the plant pathogen *Xoo* bacteria to the logarithmic growth phase (OD_595_ = 0.6~0.8). Centrifuge the culture (8,000 g, 4.0 °C, 4.0 min) and collect the *Xoo* cells from the NB solution. Wash the cells with 5.0% glucose solution until the conductivity matches that of the 5.0% glucose solution, creating an isotonic bacterial solution. As a baseline, boil the isotonic bacterial solution for 5.0 minutes and measure the conductivity, designating this value as L_0_. Prepare a 5.0% glucose solution with various concentrations (0, 4.5, 9.0, 18, and 36 *μ*g mL^-1^) of BiTA18, BiTA18@*β*-CD, and BiTA18@*γ*-CD, and record the conductivity as L_1_. Add the same concentrations (0, 4.5, 9.0, 18, and 36 *μ*g mL^-1^) of BiTA18, BiTA18@*β*-CD, and BiTA18@γ-CD to the isotonic bacterial solution, then incubate on a shaker (220 rpm, 28 ± 1.0 °C) for 8.0 hours. Measure the conductivity (L_2_) at 1-hour intervals, and calculate the relative conductivity using the formula: (L_2_ − L_1_) / L_0_.

## 1.19 Measurement of ROS Levels

Culturing the plant pathogen *Xoo* cells to the logarithmic growth phase, dilute the bacteria to an OD_595 nm_ of 0.1 using sterile NB medium. Next, incubate *Xoo* with different concentrations (0, 4.5, 9.0, 18, and 36 *μ*g mL^-1^) of BiTA18, BiTA18@*β*-CD, and BiTA18@*γ*-CD at 28 ± 1.0 °C and 220 rpm for 12 hours. After incubation, collect the *Xoo* cells by centrifugation at 8,000 g and 4.0 °C for 4.0 minutes. Wash the bacterial cells twice with sterile water and resuspend them in sterile water. Finally, add 1.0 *μ*L of DCFH-DA dye (10 mM) to 100 *μ*L of bacterial suspension, incubate in the dark for 5.0 minutes, and measure the fluorescence intensity using a Fluoromax-4cp spectrofluorometer at an excitation wavelength of 488 nm.

## 1.20 Activity Assays of CAT and SOD Enzymes

First, culture the plant pathogenic bacterium *Xoo* to the logarithmic growth phase and dilute it to an OD_595nm_ of 0.1 using sterile NB medium. Incubate the diluted *Xoo* with different concentrations (0, 2.25, 4.5, 9.0, 18, and 36 *μ*g mL^-1^) of BiTA18, BiTA18@*β*-CD, and BiTA18@*γ*-CD at 28 ± 1.0 °C and 220 rpm for 12 hours. After incubation, collect the *Xoo* cells by centrifugation at 8,000 g and 4.0 °C for 4.0 minutes and wash the cells with pre-chilled PBS buffer. Add the extraction buffer provided by the assay kit to the bacterial pellet and disrupt the cells by sonication (30% power, 3.0 seconds on, 10 seconds off, at 0 °C, for 60 cycles). Centrifuge the lysate at 8,000 g and 4.0 °C for 10 minutes and collect the supernatant. Measure the activities of catalase (CAT) and superoxide dismutase (SOD) according to the kit instructions. The protein concentration is determined by the Bradford method. Both the CAT and SOD assay kits are provided by Solarbio Life Sciences.

## 1.21 Measurement of Extracellular Polysaccharide

The *Xoo* cells suspension were adjusted to OD_595 nm_ = 0.1, and then, a total of 25 mL of the above-mentioned bacterial suspension was taken in a 50 mL sterilized conical flask, and different doses of BiTA18, BiTA18@*β*-CD, BiTA18@*γ*-CD (final concentrations were expressed as 1.12 *μ*g mL^-1^ (0.5×EC_50_), 2.25 *μ*g mL^-1^ (1.0×EC_50_), 4.5 *μ*g mL^-1^ (2.0×EC_50_), 9.0 *μ*g mL^-1^ (4.0×EC_50_), 18 *μ*g mL^-1^ (8.0×EC_50_) were added. *β*-CD, *γ*-CD and 0.036% DMSO were used as control samples. After 5 - 6 days of shaking culture (28 °C, 220 rpm), centrifugation for 10 min (10,000 rpm, 4.0 ℃), 10 mL of the supernatant was added into of ethanol (95%) of 30 mL, which was cooled to 4.0 °C in advance, and stood for 12 h (4.0 °C). After that, centrifugation (10,000 rpm, 10 min, 4.0 ℃) was performed again to collect the lower precipitate. Drying and weighing to determine the yield of EPS produced by the bacteria.

## 1.22 Measurement of Protein Content

The interaction between the compounds and bacteria was conducted in the manner described in section 1.21 for 5-6 days, the concentrations were 1.12 *μ*g mL^-1^ (0.5×EC_50_), 2.25 *μ*g mL^-1^ (1.0×EC_50_), 4.5 *μ*g mL^-1^ (2.0×EC_50_), 9.0 (4.0×EC_50_), 18 *μ*g mL^-1^ (8.0×EC_50_), respectively. Then centrifuge (10,000 rpm, 20 min, 4.0 ℃), discard the supernatant to obtain the lower precipitate, add secondary distilled water of 5.0 mL, ultrasonic 10 min (stop every three seconds), centrifuge again (10,000 rpm, 20 min, 4.0 ℃), then discard the lower precipitate and take 0.5 mL supernatant, and add 2.0 mL Coomassie Brilliant Blue G250.^[7]^ The reaction was carried out under dark conditions for 5.0 min. Pure Coomassie brilliant blue dye solution was used as blank control and the turbidity (OD_595 nm_) was tested by a microplate reader to obtain the relative content of PN.

## 1.23 Measurement of Cellulase Inhibition

BiTA18, BiTA18@*β*-CD, and BiTA18@*γ*-CD at an effective concentration of 9.0 *μ*g mL^-1^ (4.0 EC_50_) were added to the medium containing *Xoo*-bacteria (OD_595 nm_ = 0.1) to create a NB liquid medium in which bacteria and compounds coexist. Pour 10 mL of 1.5 % water agar into a sterile culture dish of 90 mm. After natural cooling, place four pre-sterilized Oxford cups (7.8×6×10 mm). Next, add 10 mL of NB solid medium containing sodium carboxymethyl cellulose of 0.5 % and agar powder of 1.5%. Cool and remove the Oxford cup. Subsequently, 2.0 *μ*L of the above-mentioned compound-containing medium was added to the pores formed by the Oxford cup, *β*-CD, *γ*-CD and 0.02% DMSO were used as control samples. Static incubation for 3 days at 28 ℃. Immediately after that, the *Xoo*-bacteria in the pores formed by the Oxford cup were rinsed with secondary distilled water, 10 mL of 0.1 % Congo red staining solution was added for staining for 3-4 h, then the plate was washed twice again with secondary distilled water, 10 mL of 1.0 mol L^-1^ NaCl solution was added for decolorization for 3-4 h. Finally, the diameter of the water ring was measured to obtain the final inhibitory effect of the compounds on cellulase.

## 1.24 Measurement of Amylase Inhibition

BiTA18, BiTA18@*β*-CD, and BiTA18@*γ*-CD at an effective concentration of 9.0 *μ*g mL^-1^ (4.0×EC_50_) were added to the medium containing *Xoo*-bacteria (OD_595 nm_ = 0.1) to create a NB liquid medium in which bacteria and compounds coexist, in advance. Pour 10 mL of 1.5 % water agar into a sterile culture dish of 90 mm. After natural cooling, place four pre-sterilized Oxford cups. Pour 10 mL of 1.5 % water agar into a sterile culture dish of 90 mm. After natural cooling, place four pre-sterilized Oxford cups. Next, add 10 mL of NB solid medium containing 0.1 % soluble starch and 1.5 % agar powder. Cool and remove the Oxford cup. Subsequently, 2.0 *μ*L of the above-mentioned compound-containing medium was added to the pores formed by the Oxford cup, *β*-CD, *γ*-CD and 0.02% DMSO. 28 ± 1.0 ℃ static incubation for 4 ~ 5 days. After washing the colonies in the plate with secondary distilled water, 10 mL 0.5 % I/KI dye solution was added to the dye for 10 min, then 70 % ethanol was used for decolorization treatment. Finally, the diameter of the water ring was measured to evaluate the compounds pair.

## 1.25 Swimming Motility Assay

*Xoo*-bacteria were pre-incubated to a turbidity (OD_595 nm_) of 1.0. A total of 10 mL NB semi-solid medium containing compounds was added to a 90 mm sterile culture dish, in which the compounds concentrations were 9.0 (4.0×EC_50_) *μ*g mL^-1^. After cooling and solidification, a compound-containing plate was formed, and 2.0 *μ*L of the above *Xoo*-bacterial suspension was added to the center of the plate, sealed and cultured at 28 ± 1.0 °C for 3-4 days. The diameter of bacterial swimming was measured to evaluate the inhibitory effect of compounds on bacterial swimming.

## 1.26 Pathogenicity Analysis

The pre-mixed bacteria cultured for 12 h were invaded into rice leaves by leaf cutting method, where the initial turbidity (OD_595 nm_) of *Xoo-*bacteria is 0.1, and the compounds dose was 9.0 *μ*g mL^-1^ (4.0×EC_50_). After 14 days of infection, the length of rice leaves infected was measured to evaluate the inhibitory effect of compounds on bacterial pathogenicity.

## 1.27 *In Vivo* Activity Test against Rice Bacterial Blight

*Xoo*-bacteria were pre-incubated to the logarithmic growth phase (OD_595 nm_ = 0.6 ~ 0.8), infected it into normally cultured mature rice plants by leaf cutting method. For protective activity: before 24 hours of infecting bacteria, 20 mL of each compound (200 *μ*g mL^-1^) was sprayed on rice plants. For curative activity: After 24 hours of infection with bacteria, 20 mL of each component of the compound (200 *μ*g mL^-1^) was sprayed on the rice plant. Among them, BiTA18, BiTA18@*β*-CD, and BiTA18@*γ*-CD were test groups, the commercial TC-20%SC and KSM were selected as positive controls, *β*-CD, *γ*-CD, and 0.4% DMSO were used as control samples. After 14 days, the total length and susceptible length of rice leaves were measured. The disease index (C or T) of the compounds was calculated following this formula:

$$\text{Disease index }\left( \text{C or T} \right)\text{ =}\frac{\text{∑ (the number of leaves at each Grade × the corresponding Grade)}}{\text{(the total number of leaves × the superlative Grade)}\text{ }}$$

The control efficiencies I for the *anti-Xoo* activity were calculated following equation:

$$\text{Control efficiency I =}\left( \text{C – T} \right)/\text{C}\text{ ×100\%}$$

In the equation, C is the disease index of the DMSO control and T is the treatment group, including test groups, positive controls, negative controls.

## 1.28 *In Vivo* Activity Test against Citrus Canker

*Xac*-bacteria were pre-cultivated to the logarithmic growth phase (OD_595 nm_ = 0.6 - 0.8), then the bacterial suspension was centrifuged and the upper medium was discarded, the lower *Xac*- bacteria were added with an appropriate amount of secondary distilled water to adjust its turbidity to OD_595 nm_ = 0.01, and the medium-speed filter paper of the same size as the citrus leaves was soaked. For protective activity against citrus canker: 18 small holes were formed by evenly penetrating citrus leaves with a 1.0 mL sterile syringe after disinfection treatment, then the medium-speed filter paper similar to the size of citrus leaves was soaked in each component solution at 200 *μ*g mL^-1^ and fitted to citrus leaves, discarded it after 24 h. Then the above-mentioned filter paper soaked in *Xac*-bacteria was attached to citrus leaves for 24 h. For curative activity against citrus canker: the filter paper containing *Xac*- bacteria was first adhered for 24 h, and the filter paper containing compounds was adhered for 24 h. Among them, BiTA18, BiTA18@*β*-CD, and BiTA18@*γ*-CD were test groups, the commercial TC-20%SC and KSM were selected as positive controls, *β*-CD, *γ*-CD, and 0.4% DMSO were used as control samples. In addition, the clean water treatment group (not infected with *Xac* bacteria) serves as the sterile control group. After 14 days of culture in a constant temperature incubator (28 ℃, 90% RH), the diseased portion of the leaf was uniformly sheared into 100 mg leaves, and then leaf chlorophyll was extracted with 10 mL of 85% acetone and 85% ethanol (1 : 1). The absorbance values of OD_663 nm_ and OD_645 nm_ were measured, respectively, and calculate the chlorophyll concentration (mg L^-1^) according to the following formula:

$$\text{C}_{\text{Chlorophyll A}}\text{ = 9.784 ×}{\text{ }\text{O}\text{D}}_{\text{663 nm}} \text{-}\text{0.99 × }{\text{O}\text{D}}_{\text{6}\text{45 nm}}\text{×0}\text{.1}$$

$$\text{C}_{\text{Chlorophyll B}}\text{ = 21.426 ×}{\text{ }\text{O}\text{D}}_{\text{645 nm}} \text{- }\text{4.65 ×}{\text{ }\text{O}\text{D}}_{\text{663 nm}}\text{×0.1}$$

$$\text{Total }\text{C}_{\text{Chlorophyll}}\text{ =}\text{ C}_{\text{Chlorophyll A}}\text{ + }\text{C}_{\text{Chlorophyll B}}$$

The relative chlorophyll content destroyed (C_x_) by bacteria comes from the following formula:

$$\text{C}_{\text{x}}\text{=Total }\text{C}_{\text{water}}\text{ - Total }\text{C}_{\text{i}}$$

In the equation: total C_water_ indicates the chlorophyll content of the clean water treatment group (not infected with *Xac*-bacteria), total C_i_ indicates chlorophyll content of each susceptible component.

The inhibition rate (I%) from the following formula:

$$\text{I }\left( \text{\%} \right)\text{=}\left( \text{C}_{\text{DMSO}}\text{ – }\text{C}_{\text{X}} \right)/{\text{C}_{\text{DMSO}}}\text{ × 100}$$

In the equation: C_DMSO_ indicates the relative chlorophyll content of the control group destroyed by *Xac*- bacteria.

## 1.29 Sliding Behavior of Droplets on Citrus Leaves

The i-SPEED 220 high-speed cameras (iX Cameras) has been used to record the micro-process of water droplets impacting citrus leaves (60° tilt with the ground) at a speed of 3000 fps, BiTA18, BiTA18@*β*-CD, BiTA18@*γ*-CD, *β*-CD, *γ*-CD aqueous solutions at 200 *μ*g mL^-1^ and water were punched out through 0.33 mm × 1.3 mm needles, respectively. The parameters are obtained by analyzing the video using i-SPEED Suite software. The droplet height is 15 cm.

## 1.30 Toxicity of Compounds on Non-target Organisms

For zebrafish toxicity test, ten red zebrafish with 1.5 - 2.5 cm were selected and cultured for 1 ~ 2 days, then, transfer them to pre-prepared aqueous solutions containing BiTA18, BiTA18@*β*-CD, BiTA18@*γ*-CD, *β*-CD and *γ*-CD with a concentration of 20 *μ*g mL^-1^. Another ten red zebrafish were cultured in water under the same environment as a blank control group. The survival status of zebrafish at 96 hours were observed and recorded to preliminarily evaluate the toxicity of compounds to zebrafish. The entire experiment maintained a dissolved oxygen content greater than 60 % of air-saturated oxygen, with a pH of 6.0 - 8.5 and a temperature of 21 - 25 ℃.

For the earthworm toxicity test, Eisenia foetida was selected as the experimental object. First, earthworms selected from the soil were placed in a disposable bowl containing filter paper to spit sand for 24 h. During this period, a glass bottle with a diameter of 4.8 cm at the bottom was selected, a layer of filter paper was put in, aqueous solutions of BiTA18, BiTA18@*β*-CD, BiTA18@*γ*-CD, *β*-CD and *γ*-CD (3.0 mL, 300 *μ*g mL^-1^) were added, and dried at 80 °C, in order to give a final concentration was 0.05 mg cm^-2^. 3.0 mL water was added to the glass bottle and two earthworms were put into each glass bottle respectively. Each compounds component was set up in five parallels, a total of ten earthworms, of which earthworms under pure water conditions were used as blank controls. Finally, the survival rate at 72 h was finally calculated to evaluate the toxicity of compounds. The test temperature was set to 25 ℃.

## 1.31 Toxicity of Compounds to Rice Organisms

For the experiment on the germination rate of rice seeds, a layer of medium-speed filter paper with a diameter of 90 mm was placed in a disposable petri dish with a diameter of 90 mm, and 40 sterilized complete rice seeds were added to each petri dish. Each dish was added with 10 mL of each component to be tested, including BiTA18, BiTA18@*β*-CD, BiTA18@*γ*-CD, *β*-CD and *γ*-CD, at 100, 200 *μ*g mL^-1^ (*β*-CD and *γ*-CD were added to the petri dish according to the molar ratio of host/guest is 1/1, respectively). DMSO aqueous solution (0.2% and 0.4%) were used as control samples. The compounds were changed once a day, and the germination of each day was recorded. After 7 days, 20 germinated rice seedlings were randomly selected from each dish to measure root length and bud length. The test temperature was set at 28 °C and the humidity was 75%.

For the rice seedling growth experiment, in simple terms, the appropriate amount of soil was added to the white plastic box of 25 × 19 × 6.5 cm, and 20 normal cultured rice seedlings were transplanted into different plastic boxes. When the rice seedlings grew to a leaf length of about 5.0 – 7.0 cm, BiTA18, BiTA18@*β*-CD, BiTA18@*γ*-CD with 10 mL at 500 *μ*g mL^-1^ were sprayed on top of them, *β*-CD, *γ*-CD, and 1% DMSO as control samples, the compounds were sprayed every 3 days for a total of five times. After 14 days, the total length, dry weight, and fresh weight were measured to evaluate the effect of the compounds on the growth of rice seedlings.

For the rice leaf toxicity experiment, normally cultivate rice plants for about two months, and then spray 20 mL of compounds (500 *μ*g mL^-1^), including BiTA18, BiTA18@*β*-CD, BiTA18@*γ*-CD, on their leaves, and equal molar ratio of *β*-CD and *γ*-CD as a negative control, the same amount of DMSO as a negative control. After 7 days, observe whether there are toxic spots on the leaves and take photos.

## 1.32 Statistical Analysis

Statistical analyses were performed using IBM SPSS Statistics 27 and Origin 2021 for Windows. Unless otherwise specified, all experiments were conducted independently with a minimum of three replicates with similar results. Data were presented as mean ± standard deviation (SD), and the sample size corresponding to each datapoint was specified within the respective figure legend. Use one-way analysis of variance (ANOVA) by least significant difference (LSD) multiple comparison test (ns = no significant, **p*<0.05, ***p*<0.01, ****p*<0.001) and one-way analysis of variance (ANOVA) by Waller-Dunca’s method (groups labeled with different lowercase letters show statistically significant differences (*p* < 0.05) between components) to determine significant differences. It should be noted that all statistical tests conducted in this study were two-sided.

# 2. Supplementary Figures and Tables

## 2.1 *In Vitro* anti-*Xoo* Activity of Title Compounds

**Table S1.** *In vitro* preliminary antibacterial activities of compounds BiTA1-BiTA26 against *Xoo*.

| Compounds | Inhibition ratio (%) | | Compounds | Inhibition ratio (%) | | |
| --- | --- | --- | --- | --- | --- | --- |
|  | 100 *μ*g mL^-1^ | 50 *μ*g mL^-1^ |  | 100 *μ*g mL^-1^ | | 50 *μ*g mL^-1^ |
| 2-MBI | 34.82±0.34 | 20.88±3.37 | BiTA13 | 97.96±1.48 | 26.11±1.74 | |
| 1 | 27.15±2.8 | 17.51±1.16 | BiTA14 | 87.46±1.73 | 0 | |
| 2 | 36.90±3.28 | 17.70±1.04 | BiTA15 | 22.87±2.99 | 0 | |
| 3 | 21.45±3.14 | 12.11±3.81 | BiTA16 | 16.91±1.67 | 0 | |
| 4 | 34.29±1.15 | 14.78±0.94 | BiTA17 | 100 | 100 | |
| BiTA1 | 100 | 35.15±3.14 | BiTA18 | 100 | 100 | |
| BiTA2 | 6.36±1.64 | 0 | BiTA19 | 13.30±1.34 | 0 | |
| BiTA3 | 100 | 42.62±7.12 | BiTA20 | 0 | 0 | |
| BiTA4 | 37.60±5.68 | 22.57±1.20 | BiTA21 | 8.75±2.86 | 0 | |
| BiTA5 | 100 | 100 | BiTA22 | 100 | 100 | |
| BiTA6 | 57.13±1.57 | 29.83±1.11 | BiTA23 | 100 | 100 | |
| BiTA7 | 28.04±1.76 | 17.84±2.95 | BiTA24 | 100 | 100 | |
| BiTA8 | 12.27±0.50 | 0 | BiTA25 | 100 | 100 | |
| BiTA9 | 9.20±2.21 | 0 | BiTA26 | 100 | 100 | |
| BiTA10 | 100 | 100 | TC | 50.79±0.80 | 26.99±0.80 | |
| BiTA11 | 100 | 100 | KSM | 94.77±0.83 | 72.86±1.26 | |
| BiTA12 | 15.22±1.03 | 5.32±0.19 |  |  |  | |

## 2.2 UV-vis Titration Curves


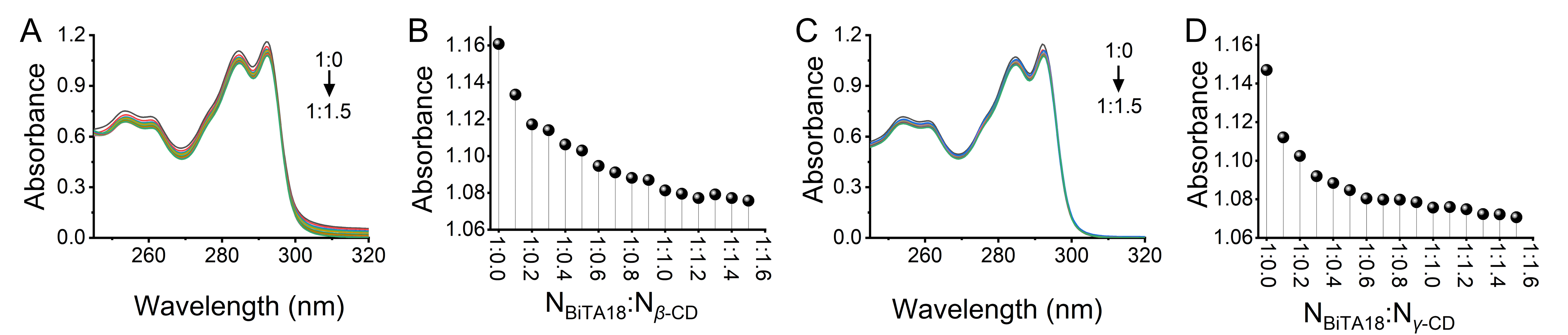


**Figure S1.** A) UV-vis absorption curves of BiTA18 (0.1 mM) after addition of different equivalents of *β*-CD (BiTA18: *β*-CD=1:0 to 1:1.5 equivalent). B) The UV-vis absorption of BiTA18 (0.1 mM) at 292 nm after adding varied equivalents of *β*-CD. C) UV-vis absorption curves of BiTA18 (0.1 mM) after addition of different equivalents of *γ*-CD (BiTA18: *γ*-CD=1:0 to 1:1.5 equivalent). D) The UV-vis absorption of BiTA18 (0.1 mM) at 292 nm after adding varied equivalents of *γ*-CD.

## 2.3 ^1^H NMR Titration Analysis of Supramolecular Complexes

**
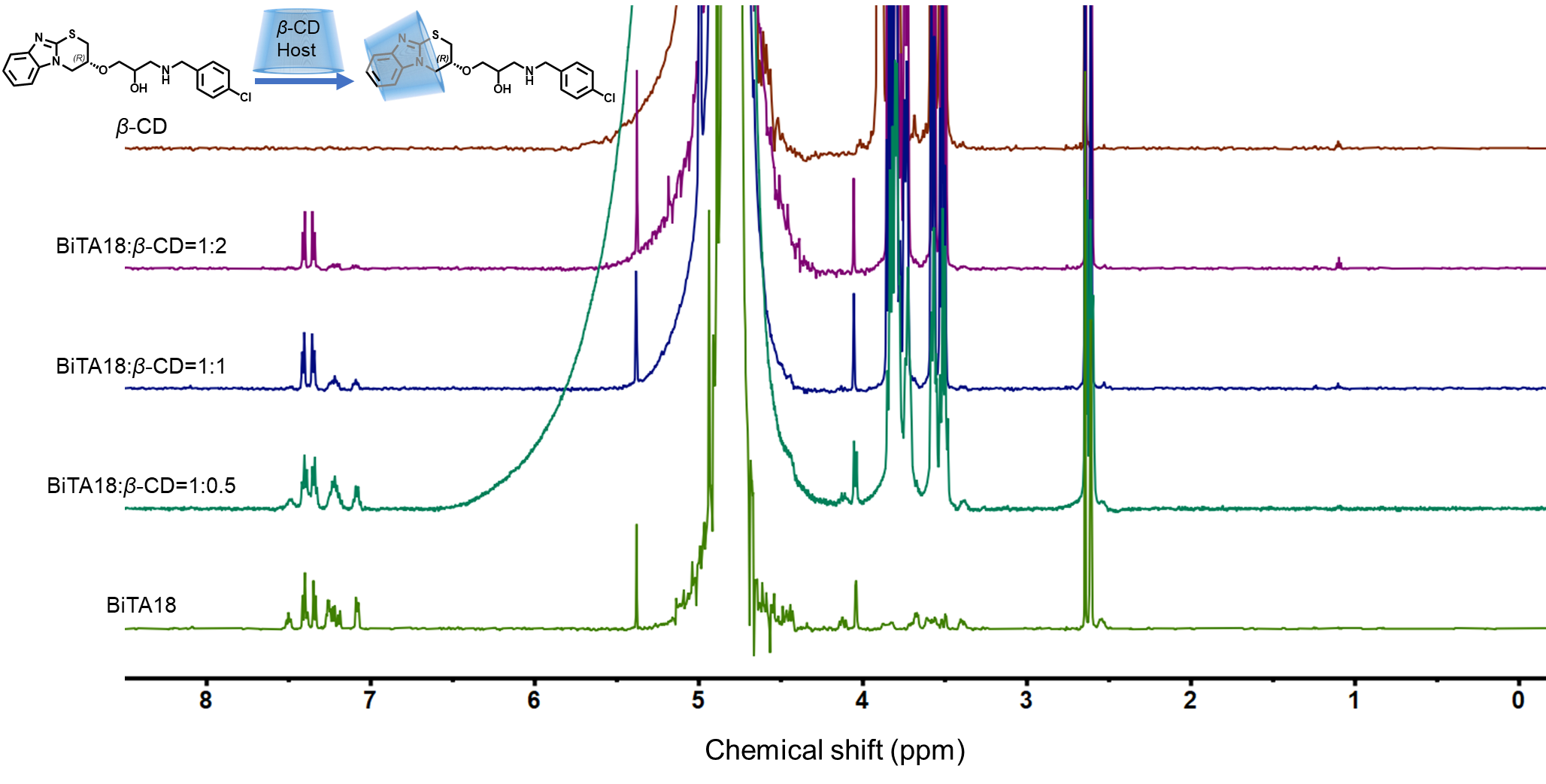
**

**Figure S2.** The ^1^H NMR titration spectrum of BiTA18@*β*-CD.

**Table S2.** The chemical shift signals for BiTA18 after adding *β*-CD.

| No. | Spectrum | H_1_ | H_2_ | H_3_ | H_4_ | H_5_ | H_6_ | H_9_ |
| --- | --- | --- | --- | --- | --- | --- | --- | --- |
| 1 | BiTA18 | 7.50 | 7.40 | 7.34 | 7.25 | 7.21 | 7.08 | 4.04 |
| 2 | *β*-CD | / | / | / | / | / | / | / |
| 3 | BiTA18:*β*-CD=1:1 | 7.48 | 7.40 | 7.35 | 7.22 | 7.23 | 7.10 | 4.06 |


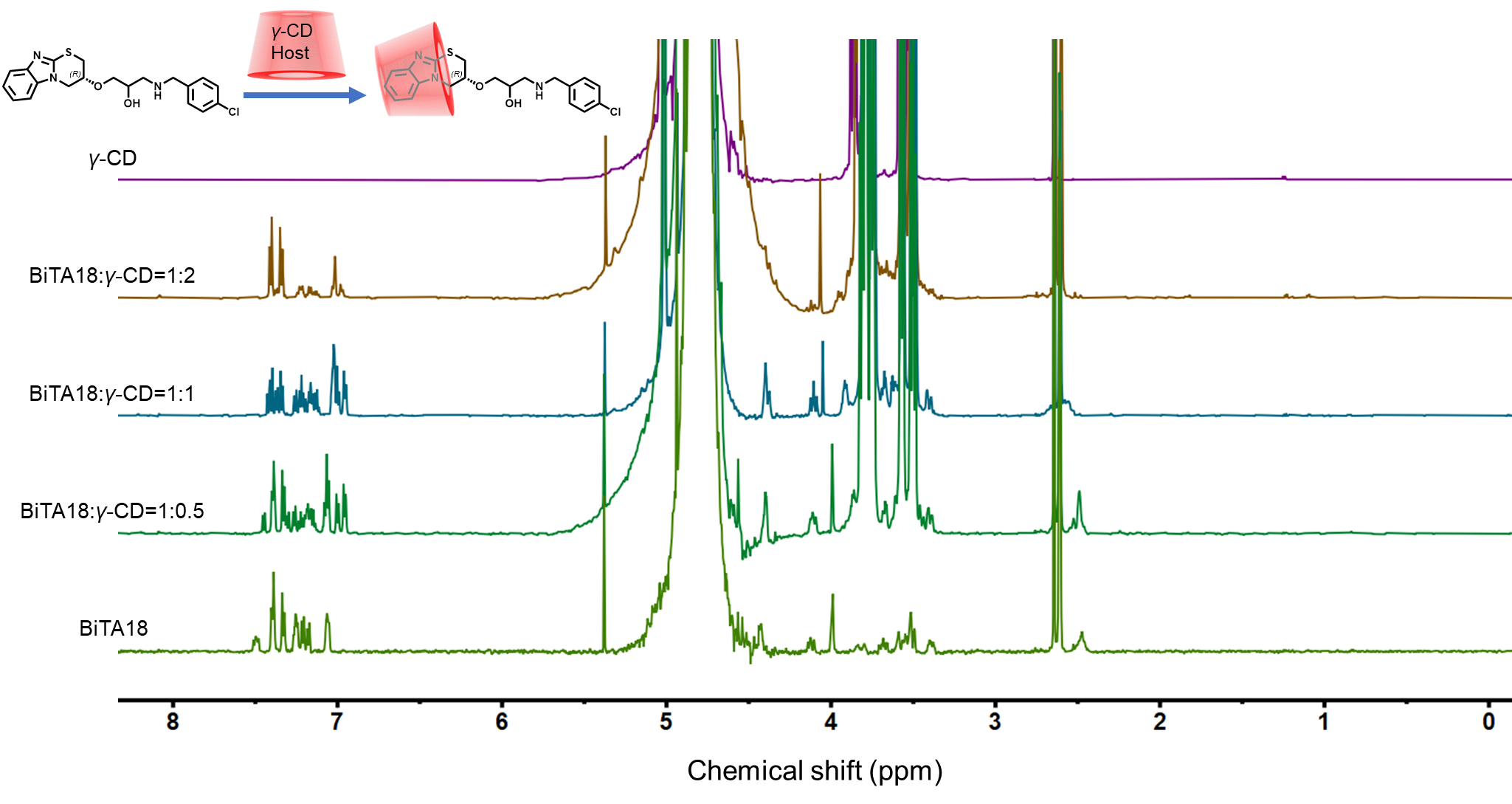


**Figure S3.** The ^1^H NMR titration spectrum of BiTA18@*γ*-CD.

**Table S3.** The chemical shift signals for BiTA18 after adding *γ*-CD.

| No. | Spectrum | H_1_ | H_2_ | H_3_ | H_4_ | H_5_ | H_6_ | H_7_ | H_8_ | H_9_ | H_10_ |
| --- | --- | --- | --- | --- | --- | --- | --- | --- | --- | --- | --- |
| 1 | BiTA18 | 7.49 | 7.40 | 7.33 | 7.25 | 7.19 | 7.06 | 3.82 | 4.11 | 3.99 | 3.38 |
| 2 | *γ*-CD |  |  |  |  |  |  |  |  |  |  |
| 3 | BiTA18:*γ*-CD =1:1 | 7.40/7.23 | 7.41 | 7.34 | 7.15 | 6.98 | 7.03 | 3.92 | 4.11 | 4.05 | 3.41 |

## 2.4 Particle Size Distributions and Zeta Potential Values at Different Storage Periods


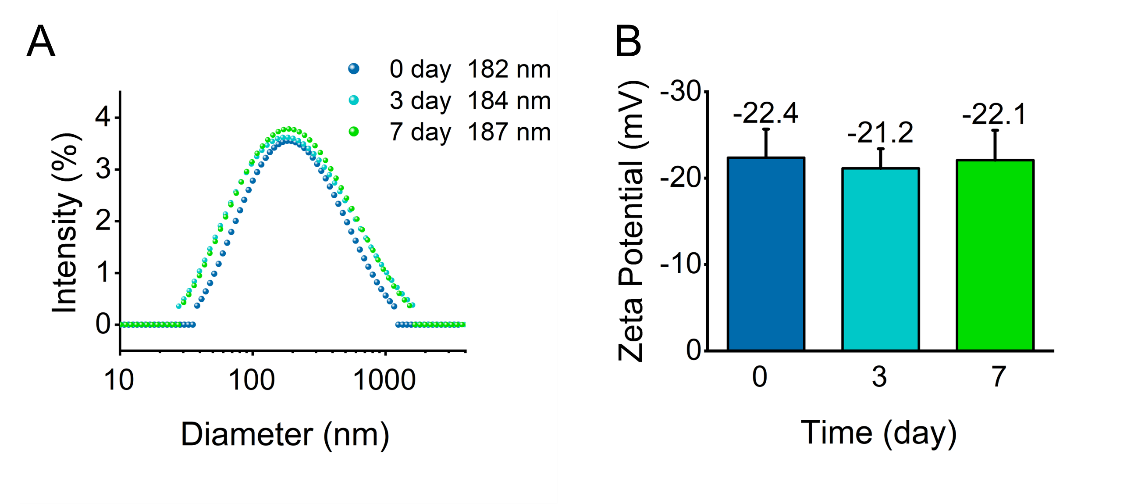


**Figure S4**. A) DLS measurements of BiTA18@*β*-CD at different storage periods (0, 3, 7 days). B) Zeta potential values for BiTA18@*β*-CD at different storage periods (0, 3, 7 days). The effective concentration of BiTA18@*β*-CD was 200 *μ*g mL^-1^ in aqueous solution.

## 2.5 UV-vis Absorption Spectra and Self-assembly Diagram of BiTA18

**
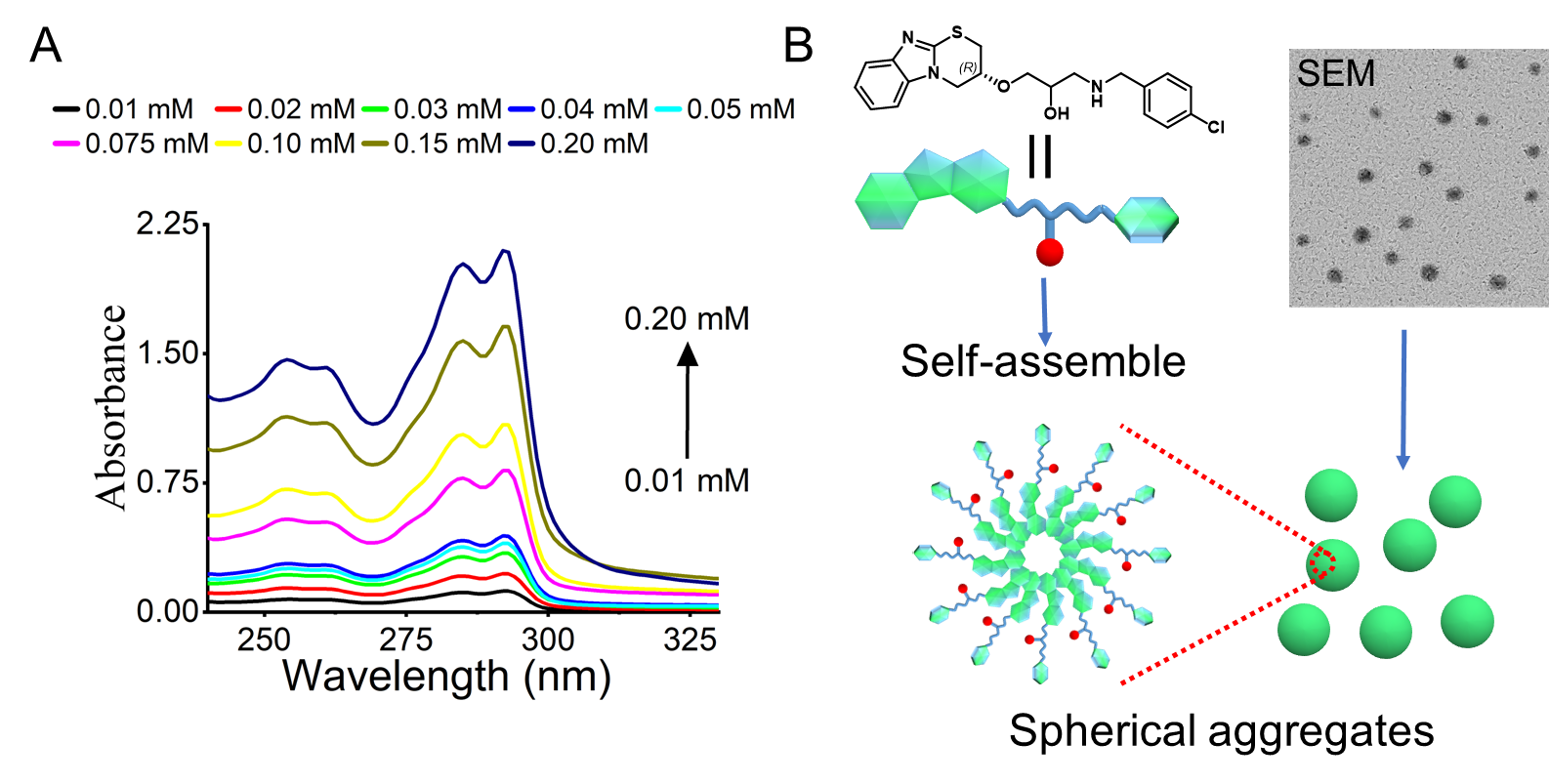
**

**Figure S5.** A) UV–vis absorption spectra of BiTA18 at different concentrations (from 0.01 to 0.20 mM). B) Self-assembly diagram of BiTA18.

## 2.6 Biofilm Inhibition Assays


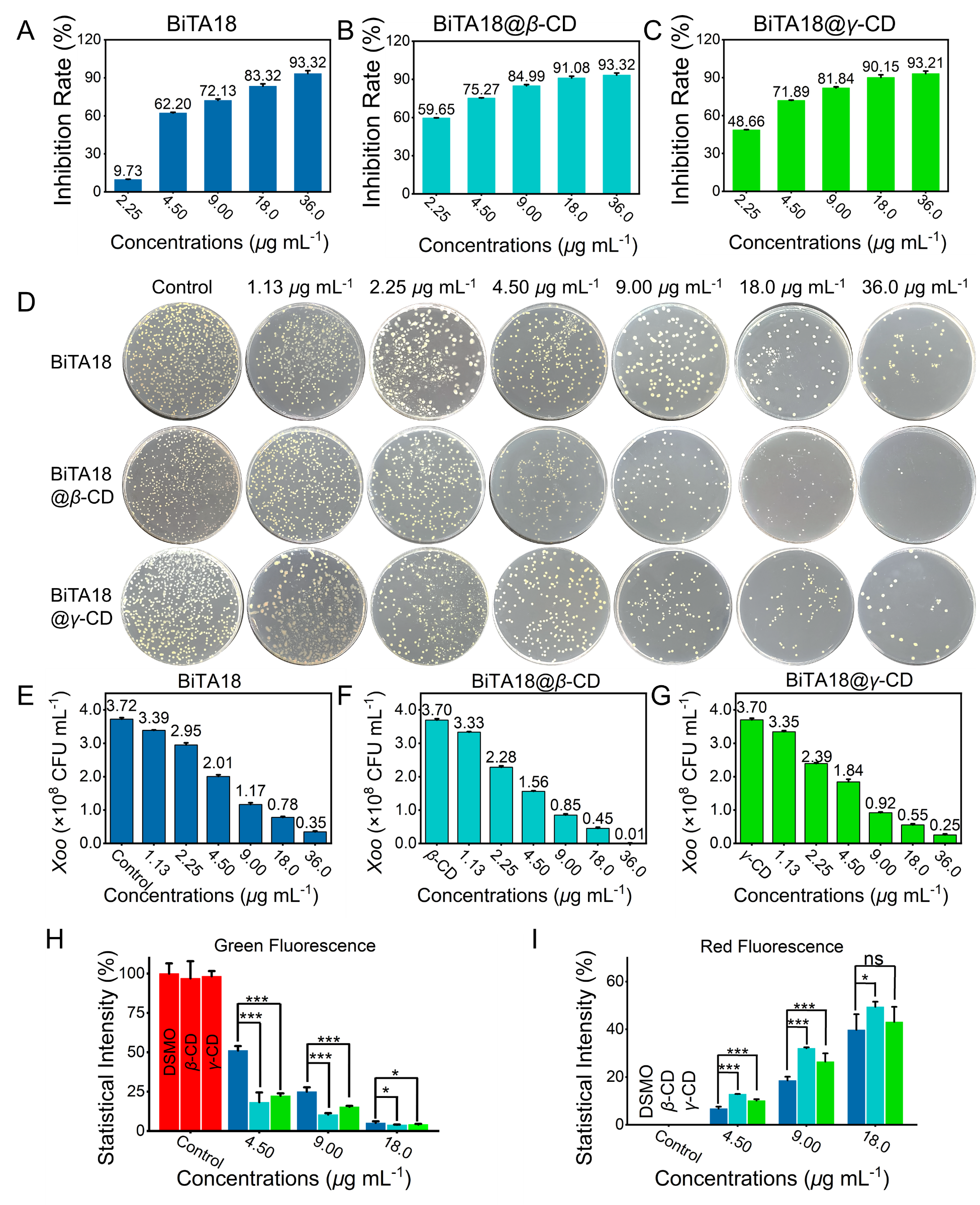


**Figure S6**. A-C) The inhibition rate of A) BiTA18, B) BiTA18@*β*-CD, and C) BiTA18@*γ*-CD on *Xoo*-biofilm. D)The colony-culturing results for *Xoo* within the biofilm after treatment with different concentrations (0, 1.13, 2.25, 4.50, 9.00, 18.0, and 36.0 *μ*g mL^-1^) of BiTA18, BiAT18@*β*-CD, and BiTA18@*γ*-CD, E-G) The number of bacterial clones within the biofilm counted from the agar plates (Condition: treatment with different bactericidal agents for 48 h). H-I) Corresponding statistical green (H) and red (I) fluorescence intensity from CLSM images from the biofilm inhibition experiment by image-J software. For (H-I), use one-way analysis of variance (ANOVA) by least significant difference (LSD) multiple comparison test (For all studies, n ≥ 3, ns: no significant, **p*<0.05, ***p*<0.01, ****p*<0.001) to determine significant differences.

## 2.7 Crystal Violet Staining for Biofilm Eradication

**
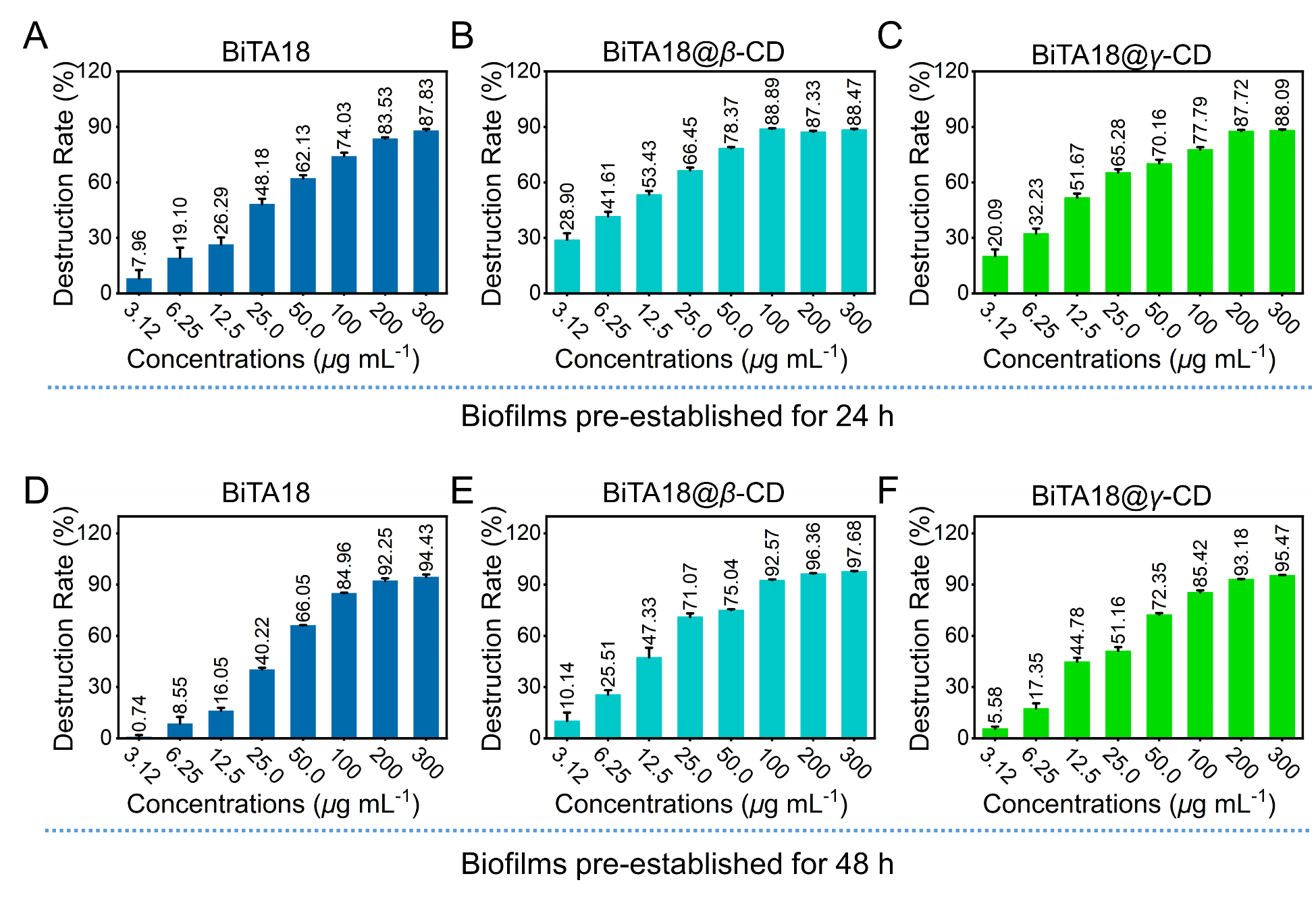
**

**Figure S7.** A-C) Destruction rates of pre-established biofilms as shown by crystal violet staining (Condition: biofilms pre-established for 24 h, and then treatment with BiTA18, BiTA18@*β*-CD, and BiTA18@*γ*-CD for 48 h). D-F) Destruction rates of pre-established biofilms as shown by crystal violet staining (Condition: biofilms pre-established for 48 h, and then treatment with BiTA18, BiTA18@*β*-CD, and BiTA18@*γ*-CD for 48 h).

## 2.8 CLSM Images Show the Eradication of Biofilms


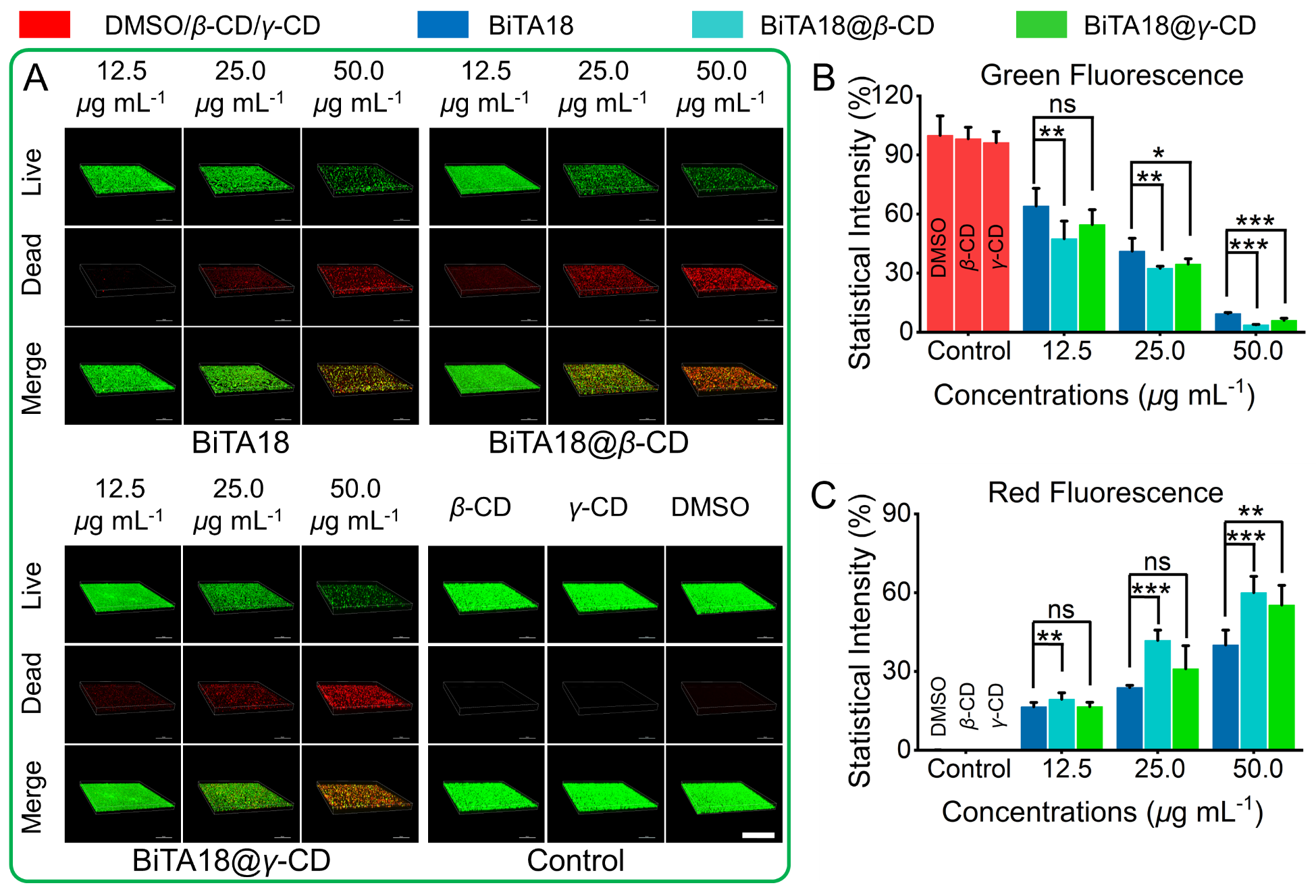


**Figure S8.** A) 3D confocal laser scanning microscopy (CLSM) images of *Xoo* strains treated with DMSO, *β*-CD, *γ*-CD, and various concentrations of BiTA18, BiTA18@*β*-CD, and BiTA18@*γ*-CD (12.5, 25.0, and 50.0 *μ*g mL^-1^), (Condition: biofilms pre-established for 48 h, and then treatment with antibacterial agents for 48 h, scale bars = 100 *μ*m). B-C) Corresponding statistical green (B) and red (C) fluorescence intensity from the left CLSM images by image-J software. For (B-C), use one-way analysis of variance (ANOVA) by least significant difference (LSD) multiple comparison test (For all studies, n ≥ 3, ns: no significant, **p*<0.05, ***p*<0.01, ****p*<0.001) to determine significant differences.

## 2.9 The Plate Monoclonal Experiment from Biofilm Eradication


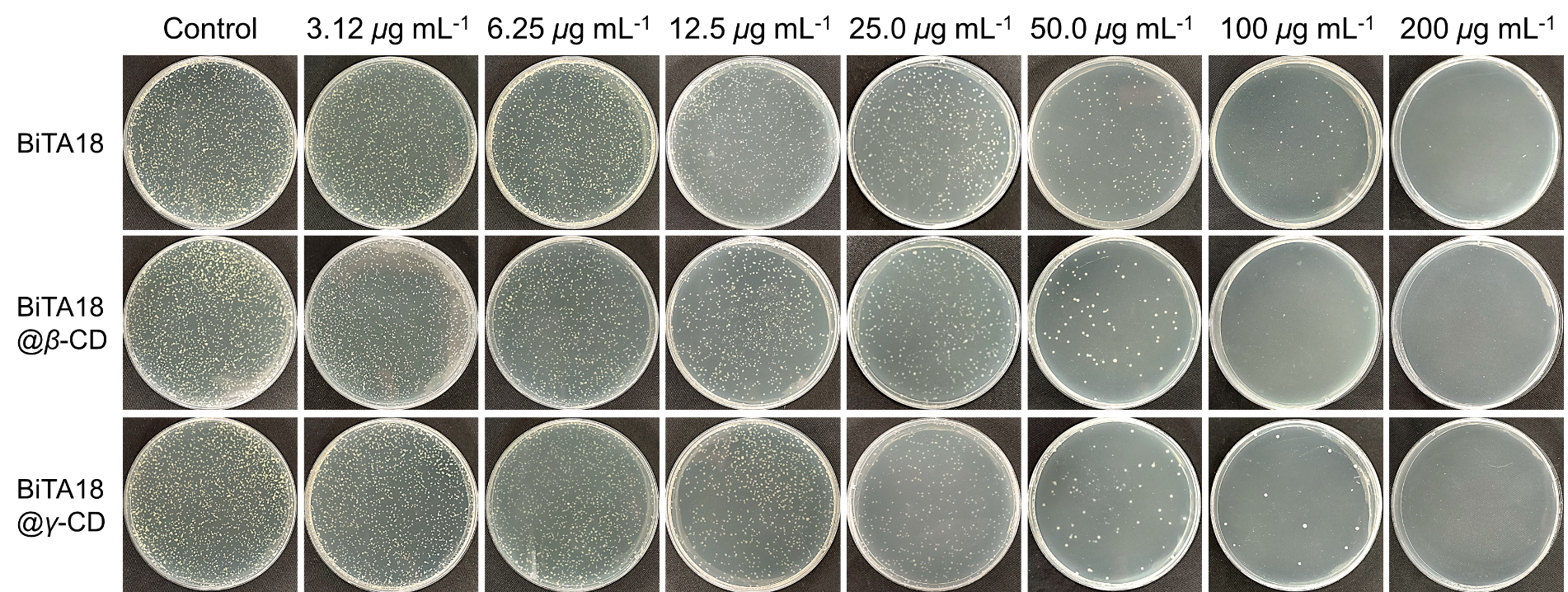


**Figure S9.** The colony-culturing results for *Xoo* cells within the biofilm after treatment with different concentrations (0, 3.12, 6.25, 12.5, 25.0, 50.0, 100, and 200 *μ*g mL^-1^) of BiTA18, BiAT18@*β*-CD, and BiTA18@*γ*-CD (Condition: *Xoo*-biofilms were pre-established for 48 h, and then treatment with bactericidal agents for 48 h).

## 2.10 The Biofilm Penetrability of BiTA18@CDs

**
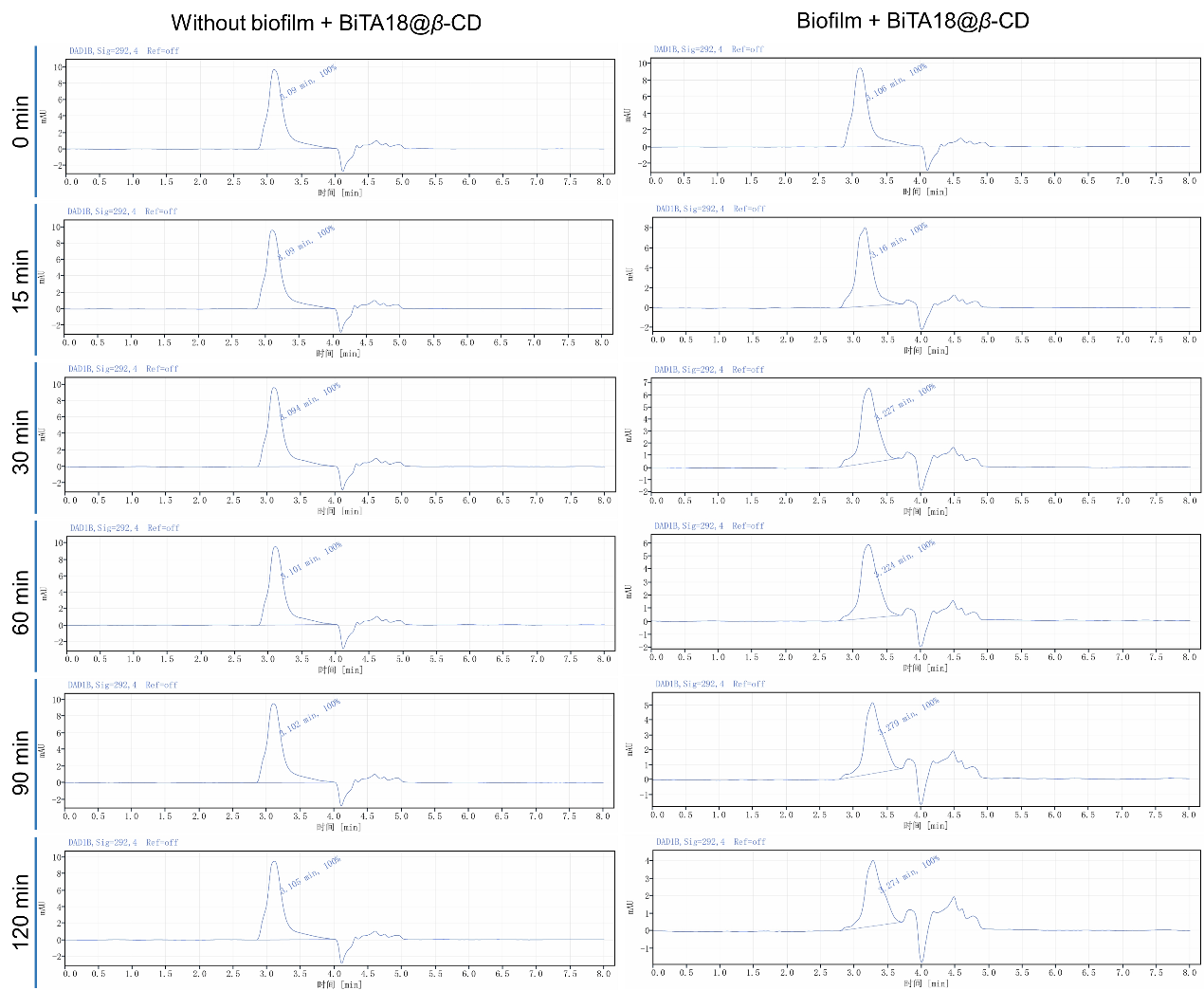
**

**Figure S10**. Representative HPLC chromatograms of BiTA18@*β*-CD at an effective concentration of 9.0 *μ*g mL^-1^ under the condition of without biofilm and with biofilm for 0, 15, 30, 60, 90 and 120 min. Conditions: XDB-C_18_ 4.6×150 mm×5 *µ*m, methanol/water = 8.0/2.0, 1.0 mL/min, injection volume 10.0 *μ*L, λ = 292 nm, Rt = 3.090-3.279 min.


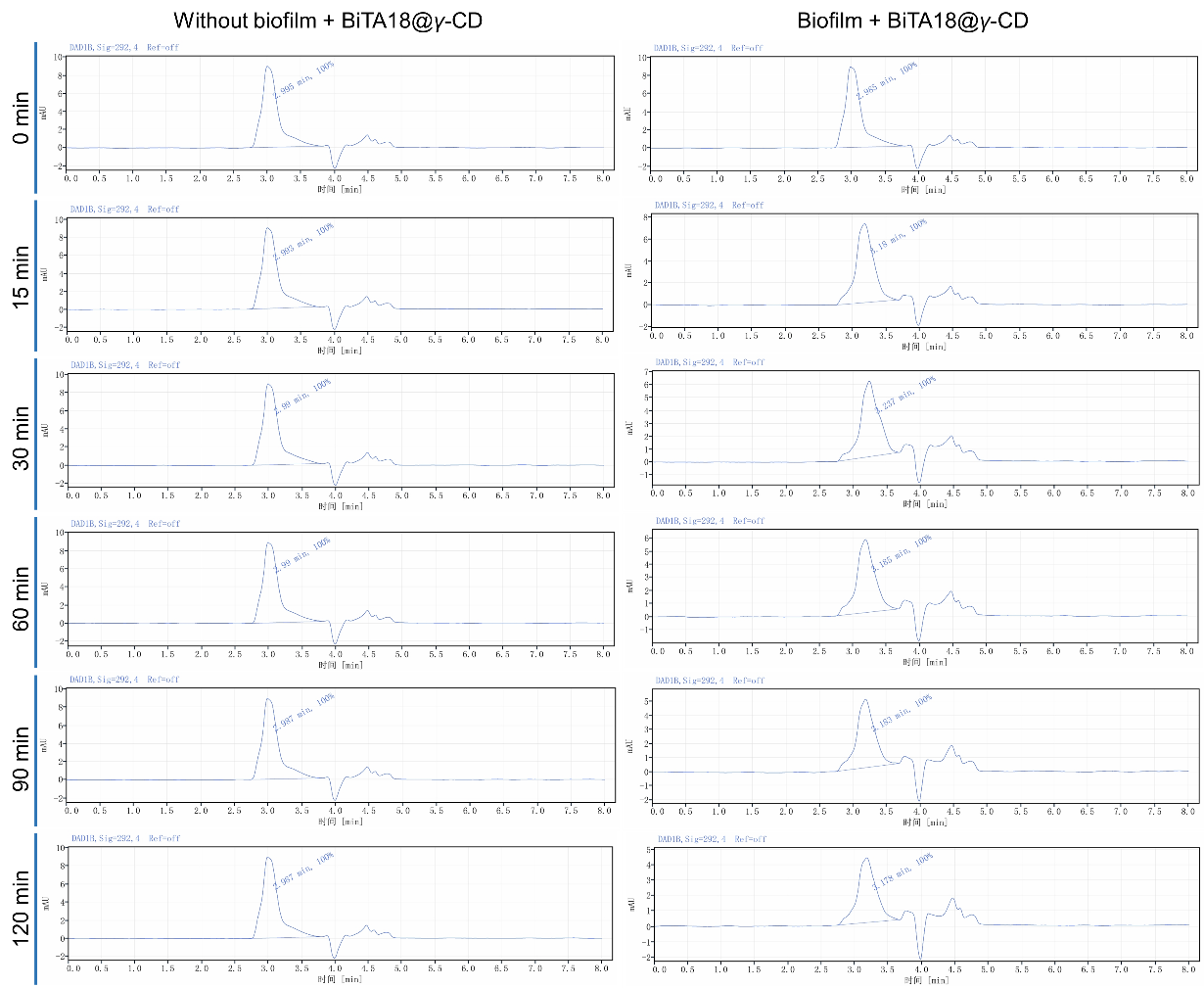


**Figure S11**. Representative HPLC chromatograms of BiTA18@*γ*-CD at an effective concentration of 9.0 *μ*g mL^-1^ under the condition of without biofilm and with biofilm for 0, 15, 30, 60, 90 and 120 min. Conditions: XDB-C_18_ 4.6×150 mm×5 *µ*m, methanol/water = 8.0/2.0, 1.0 mL/min, injection volume 10.0 *μ*L, λ = 292 nm, Rt = 2.985-3.237 min.


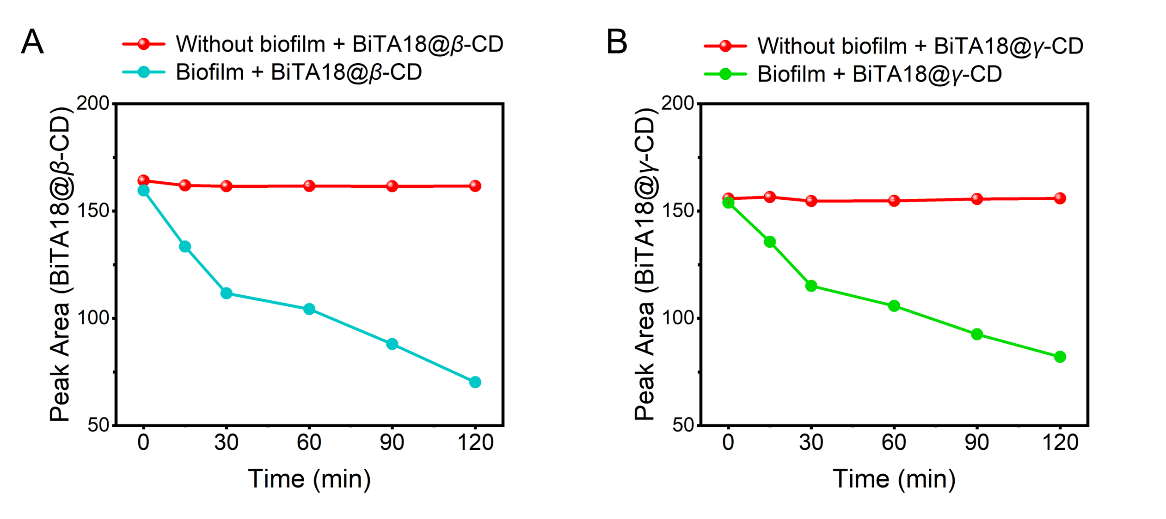


**Figure S12.** The peak area of BiTA18@*β*-CD (A) and BiTA18@*γ*-CD (B) at different time (0, 15, 30, 60, 90 and 120 min) under the condition of without biofilm and with biofilm, the effective concentration of BiTA18@*β*-CD and BiTA18@*γ*-CD was 9.0 *μ*g mL^-1^.

Experimental method: The mature *Xoo* biofilms were firstly preformed in 6-well plates, then 8.0 mL BiTA18@*β*-CD and BiTA18@*γ*-CD aqueous solution with a concentration of 9.0 *µ*g mL^-1^ were added into the above pre-established biofilms to assess the entrance and absorption of BiTA18@*β*-CD and BiTA18@*γ*-CD through HPLC determination (Conditions: XDB-C_18_ 4.6×150 mm×5 *µ*m, methanol/water = 8.0/2.0, 1.0 mL/min, injection volume 10.0 *μ*L, λ = 292 nm, Rt = 2.985-3.279 min.).

## 2.11 SEM Images of *Xoo* Biofilms Triggered by BiTA18@CDs

**
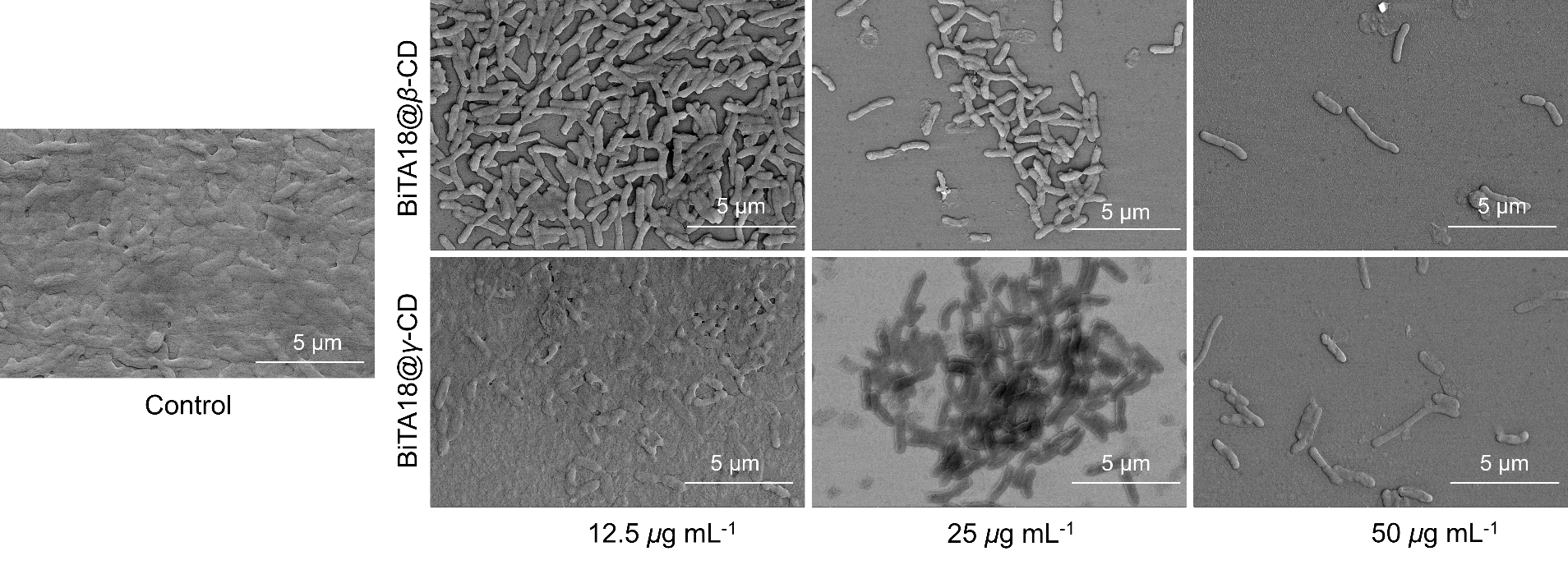
**

**Figure S13.** SEM images of *Xoo* biofilm pre-incubated for 48 h and then treated with BiTA18@*β*-CD or BiTA18@*γ*-CD (at effective concentrations of 12.5, 25.0, and 50.0 *μ*g mL^-1^) for an additional 48 h, scale bar: 5 *μ*m.

Experimental method: First, 5.0 mL of resuspended *Xoo* bacterial solution (overnight culture, OD_595 nm_ = 0.6) was added to 12-well cell culture plates containing sterilized conductive glass. The plates were incubated at 28 °C for 48 h. After incubation, the bacterial solution was removed, and planktonic bacteria were gently washed once with PBS buffer (pH = 7.4). Next, 5.0 mL of fresh medium was added, followed by the introduction of BiTA18@*β*-CD and BiTA18@*γ*-CD at effective concentrations of 12.5, 25.0, and 50.0 *μ*g mL^−1^, respectively. The plates were then incubated for an additional 48 h. After incubation, the bacterial liquid was carefully aspirated, and 5.0 mL of PBS was slowly added along the well wall for a single wash. Subsequently, 5.0 mL of 2.5% glutaraldehyde was added to fix the biofilm for 12 h. The glutaraldehyde was then aspirated, and the samples were dehydrated using a graded ethanol series (30%, 50%, 70%, 90%, and 100%), with each concentration applied for 10 min. After dehydration, the ethanol was removed, and the samples were freeze-dried for 3 h before imaging.

## 2.12 The Transcriptional Level of the Interrelated *Gum* Gene Cluster in *Xoo*


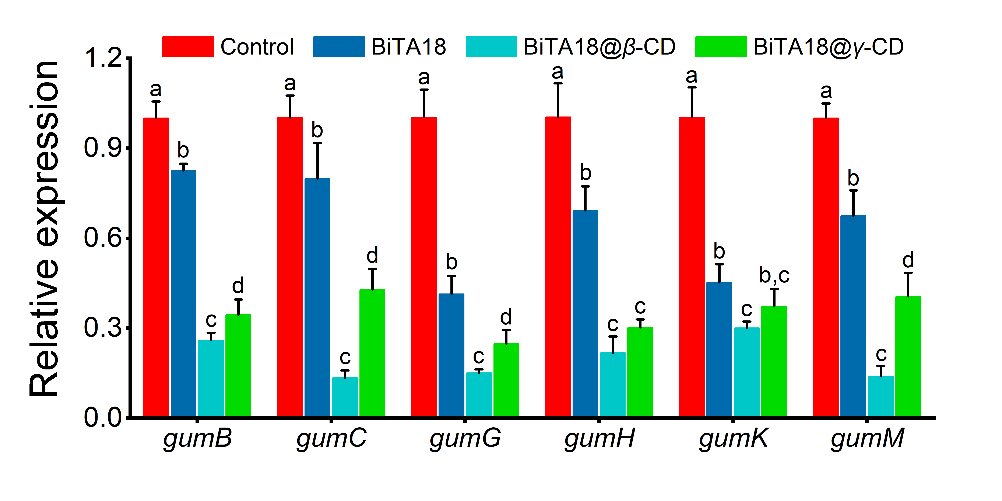


**Figure S14.** The transcriptional levels of *gum* genes in response to BiTA18, BiTA18@*β*-CD and BiTA18@*γ*-CD treatment at an effective concentration of 9.00 *μ*g mL^-1^. The error bars represent the standard error of the means of three independent replicates of qRT-PCR analysis. Different letters indicate significant differences between treatments within the same gene at *P* < 0.05.

**Table S4.** Primer sequences for qRT-PCR.

| Gene | Forward primers | Reverse primers |
| --- | --- | --- |
| *gumB* | GCCATATTTCGTTGCCGCTT | GGAACACGATGACATTGCCG |
| *gumC* | GTTTGCGGAACAACGAGCTT | CGTAGGCACATCTGCGGTAT |
| *gumG* | ACTCTCTCCAACGCATGGTG | GCCCGACAACACGAAAAACA |
| *gumH* | CGGCATGAGGGTTTCGGTAT | CATAAGCCATGGACGCGGTA |
| *gumK* | GAAATGAAGCACGCCGAGAC | CCGCAATAACGGAATCAGCG |
| *gumM* | CTATTCCATGCGTTGGCAGC | ATACGGAATCAGGTCGGTGC |
| *gyrB* | TTCCTCAATTCCGGCGTCAA | CATGGTTTCCTGGTAGGCGT |

Experimental method and quantitative RT-PCR analysis: For the quantitative RT-PCR analysis,^[8]^ total RNA of *Xanthomonas oryzae* pv*. oryzae* (*Xoo*) was extracted using a MolPure Bacterial RNA Kit (Yeasen, China). First-strand cDNA was synthesized cDNA using 100 ng of total RNA in a 20 *μ*L volume with FastKing gDNA Dispelling RT SuperMix (Tiangen, China). The quantitative RT-PCR was performed on a QuantStudio 5 real-time fluorescence quantitative PCR system (Thermo Fisher Scientific, Waltham, MA, USA). The cDNA was amplified using SYBR Green Master Mix (Yeasen, China). The *gyrB* gene was used as internal control, and the gene expression levels in three biological replicates were calculated using the 2^-ΔΔCt^ method.^[9]^ The primers used for real-time PCR are listed in **Table S4.**

## 2.13 Measurement of Extracellular *Xoo*-Proteins Inhibition


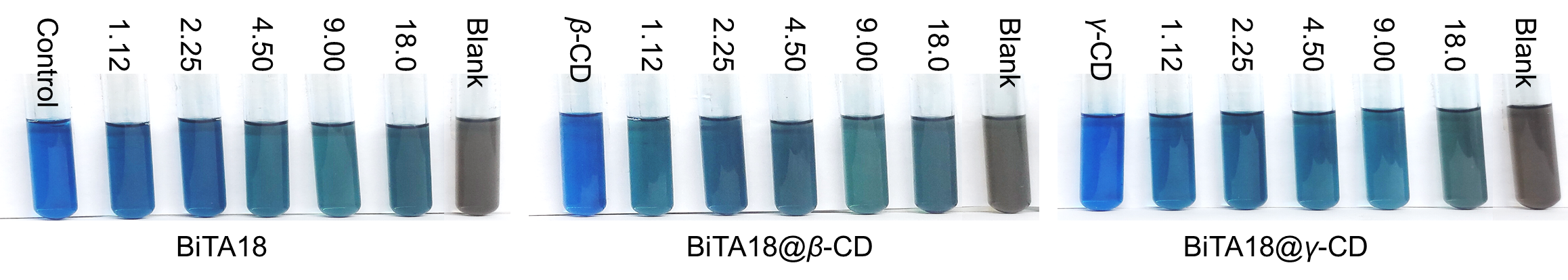


**Figure S15.** The photographs of extracellular *Xoo*-protein content stained with Kamas Brilliant Blue G250

## 2.14 *In vitro* anti-*Xac* Activity of Title Compounds

**Table S5.** *In vitro* preliminary antibacterial activities of compounds BiTA1-BiTA26 against *Xac*.

| Compounds | Inhibition ratio (%) | | Compounds | Inhibition ratio (%) | | |
| --- | --- | --- | --- | --- | --- | --- |
|  | 100 *μ*g mL^-1^ | 50 *μ*g mL^-1^ |  | 100 *μ*g mL^-1^ | | 50 *μ*g mL^-1^ |
| 2-MBI | 28.75±4.31 | 17.09±5.92 | BiTA13 | 62.37±4.63 | 34.07±2.30 | |
| 1 | 11.64±2.13 | 3.59±2.88 | BiTA14 | 66.54±3.91 | 39.08±4.54 | |
| 2 | 42.93±4.29 | 34.25±2.85 | BiTA15 | 67.47±2.22 | 46.43±2.06 | |
| 3 | 10.29±2.98 | 5.70±0.30 | BiTA16 | 28.57±2.38 | 23.53±2.79 | |
| 4 | 40.02±2.39 | 28.98±3.14 | BiTA17 | 100 | 100 | |
| BiTA1 | 33.45±1.66 | 32.32±0.56 | BiTA18 | 100 | 100 | |
| BiTA2 | 36.57±1.08 | 27.15±1.45 | BiTA19 | 0 | 0 | |
| BiTA3 | 64.77±2.80 | 39.13±1.46 | BiTA20 | 60.63±4.92 | 48.59±3.98 | |
| BiTA4 | 62.67±2.56 | 44.59±1.78 | BiTA21 | 40.20±1.76 | 38.81±4.07 | |
| BiTA5 | 100 | 79.23±0.31 | BiTA22 | 71.70±1.23 | 62.92±1.42 | |
| BiTA6 | 76.25±1.41 | 45.57±2.92 | BiTA23 | 38.19±1.87 | 28.70±1.77 | |
| BiTA7 | 76.31±1.97 | 37.70±0.91 | BiTA24 | 100 | 83.78±0.42 | |
| BiTA8 | 43.77±2.04 | 28.43±0.85 | BiTA25 | 89.24±4.33 | 62.40±7.49 | |
| BiTA9 | 59.30±2.15 | 38.84±2.42 | BiTA26 | 100 | 80.86±1.09 | |
| BiTA10 | 79.49±1.64 | 72.96±1.25 | TC | 51.98±1.89 | 20.96±2.19 | |
| BiTA11 | 100 | 100 | KSM | 85.71±2.55 | 64.68±1.60 | |
| BiTA12 | 63.26±1.70 | 32.86±1.56 |  |  |  | |

**Table S6.** The EC_50_ value of Partial Compounds against *Xac*.

| Compounds | Regression equation | R^2^ | EC_50_ ^a^ ( *μ*g mL^-1^） |
| --- | --- | --- | --- |
| BiTA5 | y=1.53x+3.76 | 0.9997 | 6.43±0.21 |
| BiTA10 | y=2.36x+1.62 | 0.9880 | 27.1±0.5 |
| BiTA11 | y=10.39x-1.27 | 0.9984 | 4.01±0.03 |
| BiTA17 | y=2.69x-1.70 | 0.9829 | 16.8±0.5 |
| BiTA18 | y=5.04x+1.60 | 0.9977 | 4.74±0.03 |
| BiTA22 | y=0.82x+3.95 | 0.9919 | 19.0±0.4 |
| BiTA24 | y=1.62x+3.30 | 0.9394 | 9.95±0.17 |
| BiTA25 | y=2.43x+1.94 | 0.9868 | 18.1±0.2 |
| BiTA26 | y=2.02x+2.46 | 0.9596 | 18.2±0.1 |
| TC ^b^ | y=1.55x+1.92 | 0.9771 | 97.1±5.0 |
| KSM^b^ | y=2.07x+1.97 | 0.9609 | 29.1±2.2 |

Note: a) EC_50_ values of antibacterial activities are indicated as means ± SD (standard deviation); b) Commercialized bactericide as the positive control. Abbreviation: TC, thiodiazole copper, KSM, kasugamycin

## 2.15 Interaction of Compounds with the Leaf Surface of Citrus

**
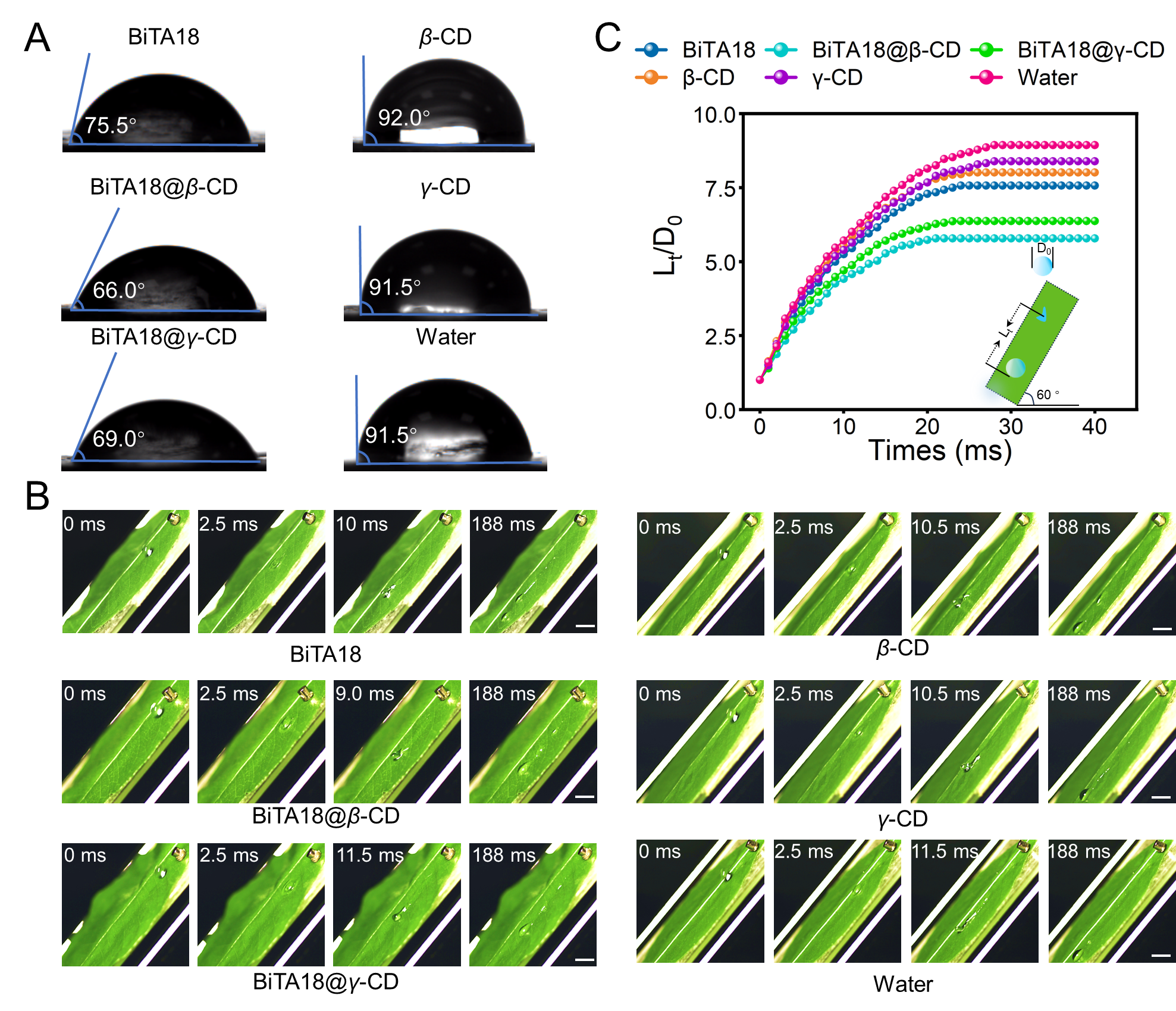
**

**Figure S16**. A) The contact angles of BiTA18, BiTA18@*β*-CD, BiTA18@*γ*-CD, *β*-CD, *γ*-CD, and H_2_O on citrus leaves. B-C) Time-resolved normalized sliding distance (L_t_/D_0_) calculated from **Video S3**, D_0_ and L_t_ represented the initial droplet diameter and the sliding distance, respectively. The angle of the inclined plane is 60°, scale bars=4.0 mm.

## 2.16 Acute Toxicity Experiment on Zebrafish and Earthworms

**
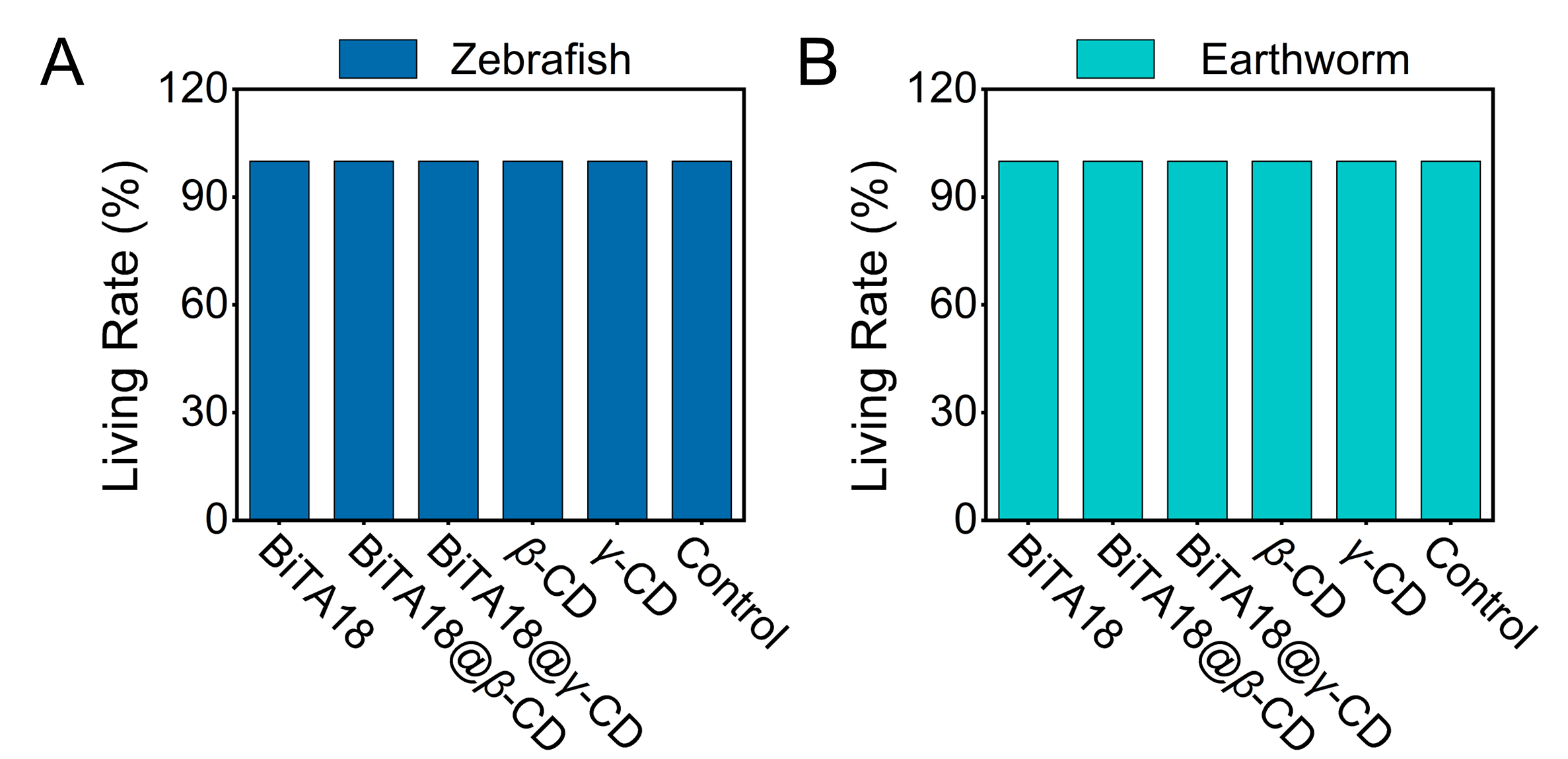
**

**Figure S17.** The final living rate of A) zebrafish and B) earthworms.

## 2.17 Rice Toxicity Experiment

**
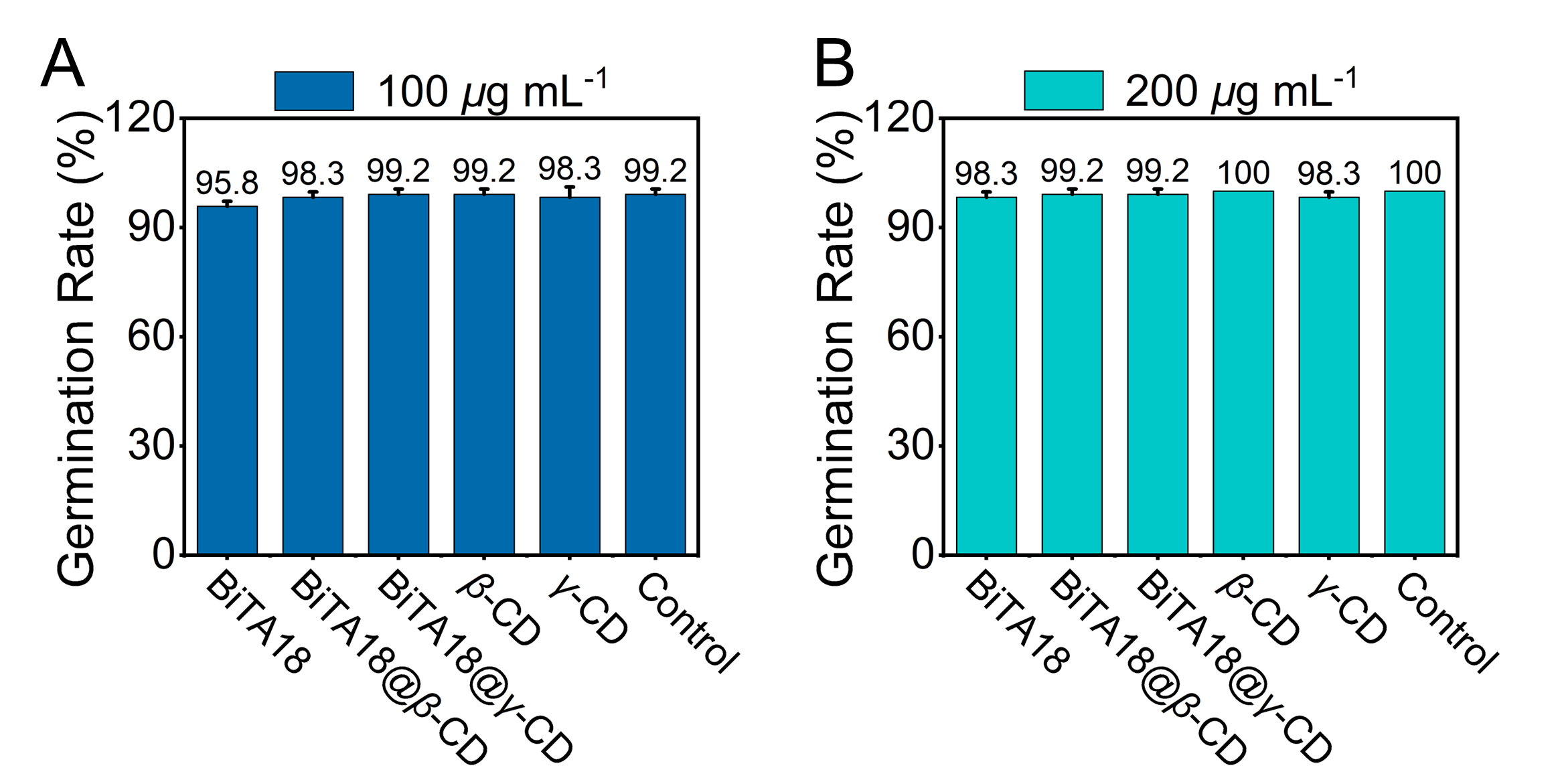
**

**Figure S18**. A-B) Germination rate of rice seeds under the action of 100 or 200 *μ*g mL^-1^ BiTA18, BiTA18@*β*-CD, and BiTA18@*γ*-CD.


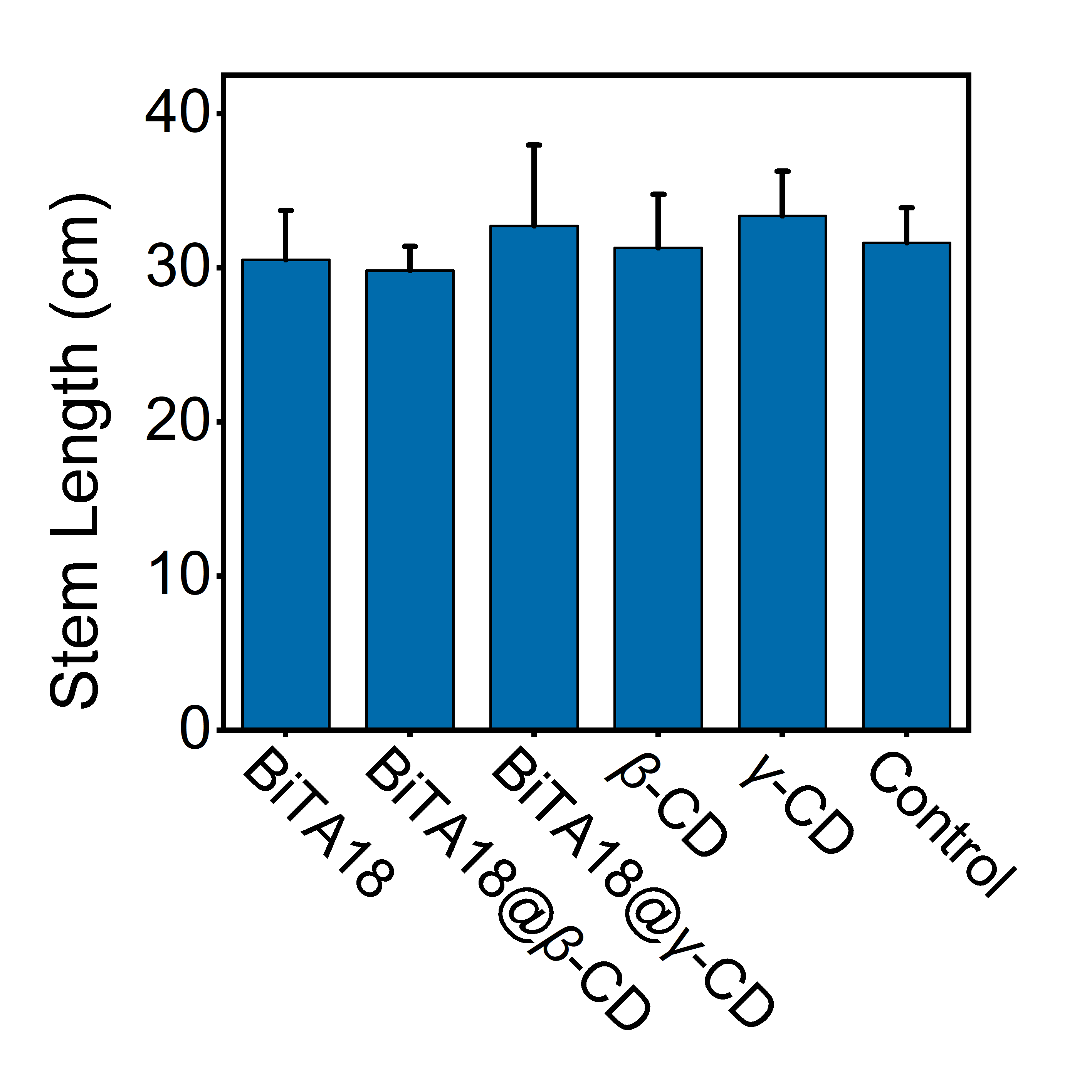


**Figure S19.** The stem length of rice seedlings after being treated with 500 *μ*g mL^-1^ BiTA18, BiTA18@*β*-CD, and BiTA18@*γ*-CD for 14 days.


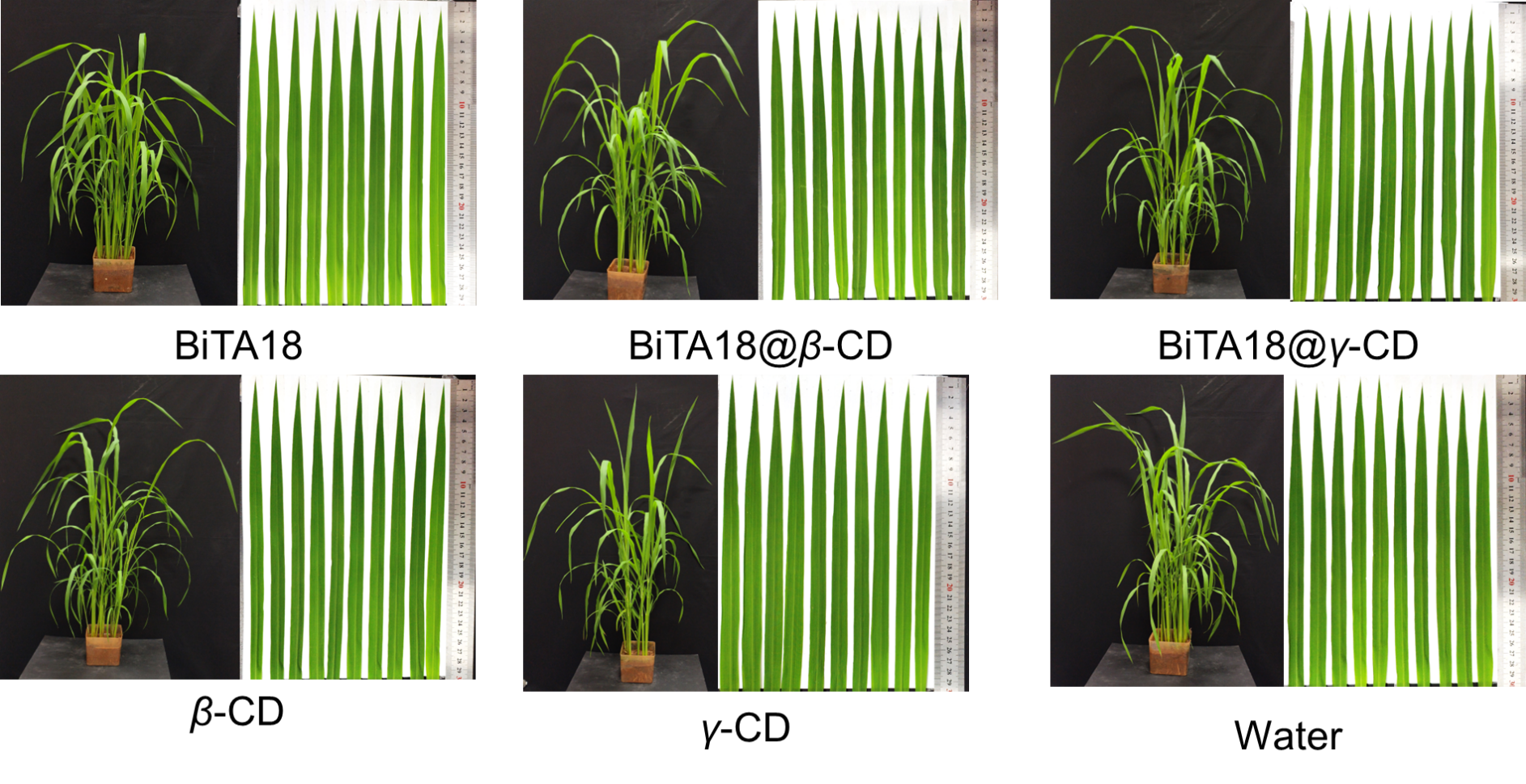


**Figure S20.** Rice leaf toxicity, from the experiment: 500 *μ*g mL^-1^ of the compounds were sprayed on mature rice leaves, and the toxic behavior of the compounds on the leaves was observed after 7 days.

# 3. ^1^H NMR, ^13^C NMR, ^19^F NMR and HRMS Analysis

**Intermediate 3, (*S*)-3,4-dihydro-2H-benzo[4,5]imidazo[2,1-b][1,3]thiazin-3-ol**

Yield, 93.7%, white powder. ^1^H NMR (400 MHz, DMSO-*d*_6_) *δ* 7.48 – 7.43 (m, 1H, Phenyl-H), 7.43 – 7.38 (m, 1H, Phenyl-H), 7.17 – 7.12 (m, 2H, Phenyl-H), 5.80 (s, 1H, -OH), 4.47 (tt, *J =* 6.1, 2.9 Hz, 1H, CH-OH), 4.23 (dd, *J =* 12.7, 3.6 Hz, 1H, N-CH_2_), 4.08 (dd, *J =* 12.7, 5.6 Hz, 1H, , N-CH_2_), 3.41 (d, *J =* 2.5 Hz, 1H, S-CH_2_), 3.21 (dd, *J =* 12.6, 6.9 Hz, 1H S-CH_2_). ^13^C NMR (101 MHz, DMSO-*d*_6_) *δ* 146.3, 142.8, 136.0, 121.8, 120.8, 117.0, 108.8, 60.6, 48.2, 31.4.

**Intermediate 4, (3*S*)-3-(oxiran-2-ylmethoxy)-3,4-dihydro-2H-benzo[4,5]imidazo[2,1-b][1,3]thiazine**

Yield, 88.52%, white powder. ^1^H NMR (500 MHz, Chloroform-*d*) *δ* 7.48 (d, *J =* 7.9 Hz, 1H, Phenyl-H), 7.10 (t, *J =* 7.5 Hz, 1H, Phenyl-H), 7.06 – 6.96 (m, 2H, Phenyl-H), 4.16 – 4.06 (m, 1H, CH_2_-CH**-** O (CH_2_)), 3.96 (d, *J =* 4.8 Hz, 1H, OCH-CH_2_–N), 3.92 (t, *J =* 4.3 Hz, 1H, OCH-CH_2_–N), 3.82 (ddd, *J =* 38.6, 11.8, 2.4 Hz, 1H, O-CH_2_), 3.30 (ddd, *J =* 57.3, 11.8, 6.2 Hz, 1H, O-CH_2_), 3.19 – 3.05 (m, 2H, OCH-CH_2_**-**S), 3.00 (tdt, *J =* 6.6, 4.6, 2.5 Hz, 1H, OCH_2_-CH-O), 2.66 (q, *J =* 4.2 Hz, 1H, OCH-CH_2_-O), 2.47 (dt, *J =* 4.7, 2.2 Hz, 1H, OCH-CH_2_-O). ^13^C NMR (126 MHz, Chloroform-*d*) *δ* 145.9, 145.8, 142.9, 142.8, 135.5, 135.5, 122.1, 121.2, 117.5, 117.5, 107.8, 107.7, 69.8, 69.7, 69.5, 69.1, 50.5, 50.5, 46.5, 46.1, 43.9, 43.8, 28.8, 28.2.

**BiTA1, 1-(benzylamino)-3-(((*R*)-3,4-dihydro-2H-benzo[4,5]imidazo[2,1-b][1,3]thiazin-3-yl)oxy)propan-2-ol**

Yield, 53.42%, light yellow oily liquid. ^1^H NMR (500 MHz, Chloroform-*d*) *δ* 7.59 (d, *J =* 8.1 Hz, 1H, Phenyl-H), 7.33 – 7.28 (m, 3H, Phenyl-H), 7.26 – 7.25 (m, 1H, Phenyl-H), 7.24 (d, *J =* 2.8 Hz, 1H, Phenyl-H), 7.23 – 7.20 (m, 1H, Phenyl-H), 7.18 – 7.17 (m, 1H, Phenyl-H), 7.17 (d, *J =* 1.1 Hz, 1H, Phenyl-H), 4.27 (ddt, *J =* 8.8, 6.6, 3.2 Hz, 1H, CH-OH), 4.19 (ddd, *J =* 12.8, 6.4, 4.0 Hz, 1H, N-CH_2_-CH), 4.12 (dt, *J =* 12.7, 6.4 Hz, 1H, N-CH_2_-CH), 3.88 – 3.84 (m, 1H, CH-O), 3.80 – 3.70 (m, 2H, O-CH_2_-CH), 3.70 – 3.64 (m, 1H, Phenyl-CH_2_), 3.63 – 3.52 (m, 1H, Phenyl-CH_2_), 3.26 (dddd, *J =* 20.1, 13.0, 7.1, 3.1 Hz, 2H, S-CH_2_), 2.73 (dd, *J =* 12.1, 3.9 Hz, 1H, CH_2_-NH), 2.63 (ddd, *J =* 12.2, 7.9, 5.4 Hz, 1H, CH_2_-NH). ^13^C NMR (126 MHz, Chloroform-*d*) *δ* 146.2, 146.1, 143.3, 139.3, 139.2, 135.9, 128.7, 128.6, 128.4, 128.4, 127.5, 127.4, 122.6, 121.7, 118.2, 108.0, 72.0, 70.1, 70.0, 68.6, 68.6, 53.6, 51.0, 51.0, 46.8, 29.0, 29.0. HRMS (ESI) [M+H^+^] calcd for C_20_H_24_O_2_N_3_S^+^: 370.1584, found: 370.1573.

**BiTA2, 1-(benzyl(methyl)amino)-3-(((*R*)-3,4-dihydro-2H-benzo[4,5]imidazo[2,1-b][1,3]thiazin-3-yl)oxy)propan-2-ol**

Yield, 52.40%, light yellow oily liquid. ^1^H NMR (400 MHz, Chloroform-*d*) *δ* 7.63 – 7.56 (d, *J =* 8.0 Hz, 1H, Phenyl-H), 7.35 – 7.26 (m, 4H, Phenyl-H), 7.25 – 7.13 (m, 4H, Phenyl-H), 4.32 – 4.25 (dd, *J =* 10.5, 5.3, 3.1 Hz, 1H, CH-OH), 4.22 – 4.14 (dd, *J =* 12.1, 8.0, 4.3 Hz, 1H, N-CH_2_-CH), 4.08 – 4.01 (dd, *J =* 12.6, 6.6, 2.0 Hz, 1H, N-CH_2_-CH), 3.92 – 3.84 (m, 1H, CH-O), 3.76 – 3.70 (dd, *J =* 10.3, 3.4 Hz, 1H, O-CH_2_-CH), 3.69 – 3.59 (m, 2H, Phenyl-CH_2_), 3.56 – 3.40 (m, 2H, O-CH_2_**-**CHOH), 3.31 – 3.12 (m, 2H, S-CH_2_), 2.56 – 2.48 (dd, *J =* 12.0, 9.9, 1.9 Hz, 1H, CH-CH_2_-N-CH_3_), 2.36 – 2.30 (m, 1H, CH-CH_2_-N-CH_3_), 2.27 – 2.20 (d, *J =* 8.3 Hz, 3H, N-CH_3_).  ^13^C NMR (101 MHz, Chloroform-*d*) *δ* 146.2, 146.2, 143.3, 138.1, 138.1, 135.8, 129.2, 129.1, 128.4, 128.4, 127.4, 127.4, 122.5, 121.6, 118.1, 118.0, 108.0, 71.8, 71.5, 70.4, 70.2, 66.9, 66.8, 62.5, 59.0, 58.9, 46.7, 46.6, 42.3, 42.2, 29.7, 29.0, 28.9. HRMS (ESI) [M+H^+^] calcd for C_20_H_24_O_2_N_3_S^+^: 384.1740, found: 384.1729.

**BiTA3, 1-(benzyl(isopropyl)amino)-3-(((*R*)-3,4-dihydro-2H-benzo[4,5]imidazo[2,1-b][1,3]thiazin-3-yl)oxy)propan-2-ol**

Yield, 60.25%, light yellow oily liquid. ^1^H NMR (500 MHz, Chloroform-*d*) *δ* 7.64 – 7.57 (m, 1H, Phenyl-H), 7.34 – 7.30 (m, 1H, Phenyl-H), 7.30 – 7.27 (m, 2H, Phenyl-H), 7.27 (d, *J =* 2.0 Hz, 1H, Phenyl-H), 7.24 (d, *J =* 7.5 Hz, 1H, Phenyl-H), 7.22 – 7.19 (m, 1H, Phenyl-H), 7.19 – 7.17 (m, 1H, Phenyl-H), 7.17 (s, 1H, Phenyl-H), 4.27 (dp, *J =* 7.0, 3.9, 3.5 Hz, 1H, CH-OH), 4.21 – 4.14 (m, 1H, N-CH_2_-CH), 4.06 – 4.00 (m, 1H, N-CH_2_-CH), 3.71 – 3.63 (m, 3H, CH-O, O-CH_2_-CH), 3.63 – 3.56 (m, 1H, -OH), 3.53 – 3.42 (m, 2H, Phenyl-CH_2_), 3.30 – 3.08 (m, 2H, S-CH_2_), 2.98 – 2.86 (m, 1H, CH-(CH_3_)_2_), 2.52 – 2.40 (m, 2H, CH-CH_2_-N-CH_3_), 1.05 (dd, *J =* 28.1, 6.7 Hz, 3H, CH_3_), 0.99 (dd, *J =* 9.0, 6.5 Hz, 3H, CH_3_). ^13^C NMR (126 MHz, Chloroform-*d*) *δ* 135.9, 128.8, 128.8, 128.5, 128.5, 127.3, 127.2, 122.5, 121.5, 118.2, 118.1, 107.9, 71.8, 71.5, 70.5, 70.4, 66.8, 66.6, 54.5, 54.4, 51.2, 51.1, 50.0, 50.0, 46.8, 46.6, 29.0, 29.0, 20.4, 20.3, 15.7, 15.7. HRMS (ESI) [M+H^+^] calcd for C_23_H_30_O_2_N_3_S^+^: 412.2053, found: 412.2041.

**BiTA4, 1-(((*R*)-3,4-dihydro-2H-benzo[4,5]imidazo[2,1-b][1,3]thiazin-3-yl)oxy)-3-((2-methoxybenzyl)amino)propan-2-ol**

Yield, 43.73%, light yellow oily liquid. ^1^H NMR (400 MHz, Chloroform-*d*) *δ* 7.56 (d, *J =* 7.4 Hz, 1H, Phenyl-H), 7.26 – 7.12 (m, 5H, Phenyl-H), 6.91 – 6.79 (m, 2H, Phenyl-H), 4.25 (dt, *J =* 6.8, 3.3 Hz, 1H, CH-OH), 4.15 – 4.09 (m, 4H, N-CH_2_-CH, O-CH_2_-CH), 3.95 (dq, *J =* 8.7, 4.6 Hz, 1H, CH-O), 3.89 – 3.79 (m, 2H, N-CH_2_-CH), 3.78 (d, *J =* 4.3 Hz, 3H, O-CH_3_), 3.66 – 3.61 (m, 1H, Phenyl-CH_2_), 3.60 – 3.51 (m, 1H, Phenyl-CH_2_), 3.32 – 3.17 (m, 2H, S-CH_2_), 2.74 (dd, *J =* 12.3, 3.6 Hz, 1H, CH-CH_2_-NH), 2.62 (dd, *J =* 12.3, 8.6 Hz, 1H, CH-CH_2_-NH). ^13^C NMR (101 MHz, Chloroform-*d*) *δ* 157.7, 146.2, 143.2, 135.8, 130.6, 129.6, 124.5, 122.6, 121.7, 120.7, 118.1, 110.5, 108.1, 71.7, 71.6, 70.0, 69.9, 67.5, 55.5, 55.5, 50.4, 50.3, 48.5, 46.9, 46.8, 29.0. HRMS (ESI) [M+H^+^] calcd for C_21_H_26_O_3_N_3_S^+^: 400.1689, found: 400.1680.

**BiTA5, 1-(((*R*)-3,4-dihydro-2H-benzo[4,5]imidazo[2,1-b][1,3]thiazin-3-yl)oxy)-3-((3-methoxybenzyl)amino)propan-2-ol**

Yield, 53.01%, light yellow oily liquid. ^1^H NMR (400 MHz, Chloroform-*d*) *δ* 7.58 (d, *J =* 7.5 Hz, 1H, Phenyl-H), 7.23 – 7.15 (m, 4H, Phenyl-H), 6.85 (d, *J =* 6.8 Hz, 1H, Phenyl-H), 6.83 – 6.76 (m, 1H, Phenyl-H), 4.27 (pd, *J =* 4.1, 2.6, 1.9 Hz, 1H, CH-OH), 4.22 – 4.15 (m, 1H, N-CH_2_-CH), 4.14 – 4.08 (m, 1H, N-CH_2_-CH), 3.91 – 3.83 (m, 1H, CH-O), 3.78 (s, 3H, O-CH_3_), 3.76 – 3.70 (m, 2H, O-CH_2_-CH), 3.69 – 3.52 (m, 2H, Phenyl-CH_2_), 3.33 – 3.18 (m, 2H, S-CH_2_), 2.73 (d, *J =* 8.1 Hz, 1H, CH-CH_2_-NH), 2.66 – 2.59 (m, 1H, CH-CH_2_-NH). ^13^C NMR (101 MHz, CDCl_3_) *δ* 159.9, 146.2, 146.2, 143.3, 143.3, 140.5, 140.5, 135.9, 129.7, 129.7, 122.7, 121.7, 121.7, 120.7, 120.7, 118.2, 114.1, 114.0, 112.8, 112.8, 108.0, 72.0, 70.2, 70.0, 68.6, 68.5, 55.4, 53.5, 50.9, 50.9, 46.8, 29.8, 29.0, 29.0. HRMS (ESI) [M+H^+^] calcd for C_21_H_26_O_3_N_3_S^+^: 400.1689, found: 400.1678.

**BiTA6, 1-(((*R*)-3,4-dihydro-2H-benzo[4,5]imidazo[2,1-b][1,3]thiazin-3-yl)oxy)-3-((3-methoxybenzyl)(methyl)amino)propan-2-ol**

Yield, 85.34%, light yellow oily liquid. ^1^H NMR (500 MHz, Chloroform-*d*) *δ* 7.60 (d, *J =* 7.4 Hz, 1H, Phenyl-H), 7.24 – 7.18 (m, 4H, Phenyl-H), 6.86 – 6.78 (m, 3H, Phenyl-H), 4.31 (tdd, *J =* 7.1, 4.2, 2.9 Hz, 1H, CH-OH), 4.21 (dd, *J =* 12.5, 4.2 Hz, 1H, N-CH_2_-CH), 4.07 (dd, *J =* 12.5, 6.7 Hz, 1H, N-CH_2_-CH), 3.93 – 3.87 (m, 1H, CH-O), 3.78 (s, 3H, O-CH_3_), 3.66 (dd, *J =* 8.9, 4.3 Hz, 2H, O-CH_2_), 3.60 (d, *J =* 13.1 Hz, 1H, Phenyl-CH_2_), 3.42 (d, *J =* 13.1 Hz, 1H, Phenyl-CH_2_), 3.33 – 3.23 (m, 2H, S-CH_2_), 2.52 (dd, *J =* 12.3, 10.0 Hz, 1H, CH-CH_2_-N), 2.34 (dd, *J =* 12.3, 4.0 Hz, 1H, CH-CH_2_-N), 2.24 (s, 3H, N-CH_3_). ^13^C NMR (126 MHz, Chloroform-*d*) *δ* 159.7, 146.2, 143.4, 139.8, 135.9, 129.5, 122.5, 121.6, 121.5, 118.2, 114.9, 114.8, 112.7, 108.0, 71.6, 70.4, 66.9, 62.5, 59.1, 55.3, 46.7, 42.3, 29.0. HRMS (ESI) [M+H^+^] calcd for C_22_H_28_O_3_N_3_S^+^: 414.1846, found: 414.1833.

**BiTA7, 1-(((*R*)-3,4-dihydro-2H-benzo[4,5]imidazo[2,1-b][1,3]thiazin-3-yl)oxy)-3-((4-methoxybenzyl)amino)propan-2-ol**

Yield, 59.60%, light yellow oily liquid. ^1^H NMR (400 MHz, Chloroform-*d*) *δ* 7.61 (dd, *J =* 61.3, 8.3 Hz, 1H, Phenyl-H), 7.24 – 7.19 (m, 1H), 7.17 (s, 1H, Phenyl-H), 7.16 – 7.09 (m, 3H, Phenyl-H), 6.92 – 6.81 (m, 1H, Phenyl-H), 6.78 (d, *J =* 8.6 Hz, 1H, Phenyl-H), 4.19 (tt, *J =* 6.8, 2.8 Hz, 1H, CH-OH), 4.05 (d, *J =* 5.8 Hz, 2H, N-CH_2_-CH), 3.89 – 3.85 (m, 1H, CH-O), 3.72 (s, 3H, O-CH_3_), 3.68 (d, *J =* 6.5 Hz, 2H, O-CH_2_), 3.61 (dd, *J =* 9.8, 4.2 Hz, 1H, Phenyl-CH_2_), 3.48 (dd, *J =* 9.9, 5.8 Hz, 1H, Phenyl-CH_2_), 3.25 – 3.10 (m, 2H, S-CH_2_), 2.67 (dd, *J =* 12.2, 3.7 Hz, 1H, CH-CH_2_-N), 2.59 (dd, *J =* 12.2, 8.0 Hz, 1H, CH-CH_2_-N). ^13^C NMR (101 MHz, Chloroform-*d*) *δ* 161.7, 161.2, 159.0, 158.8, 158.7, 146.3, 143.0, 135.8, 130.1, 129.9, 129.2, 128.9, 122.6, 121.7, 117.9, 114.0, 114.0, 108.1, 71.8, 69.7, 68.2, 55.3, 52.6, 50.7, 46.7, 27.5. HRMS (ESI) [M+H^+^] calcd for C_21_H_26_O_3_N_3_S^+^: 400.1689, found: 400.1681.

**BiTA8, 1-(((*R*)-3,4-dihydro-2H-benzo[4,5]imidazo[2,1-b][1,3]thiazin-3-yl)oxy)-3-((3,4-dimethoxybenzyl)amino)propan-2-ol**

Yield, 58.18%, light yellow oily liquid. ^1^H NMR (400 MHz, Chloroform-*d*) *δ* 7.49 (d, *J =* 8.9 Hz, 1H, Phenyl-H), 7.19 – 7.10 (m, 3H, Phenyl-H), 7.09 (d, *J =* 2.2 Hz, 1H, Phenyl-H), 6.82 (t, *J =* 7.0 Hz, 1H, Phenyl-H), 6.66 (d, *J =* 8.3 Hz, 1H, Phenyl-H), 4.18 (p, *J =* 4.4, 3.9 Hz, 1H, CH-OH), 4.14 – 4.06 (m, 2H, N-CH_2_-CH), 4.02 (dt, *J =* 13.0, 4.0 Hz, 1H, CH-O), 3.89 (d, *J =* 7.7 Hz, 2H, O-CH_2_), 3.81 (s, 3H, O-CH_3_), 3.76 (d, *J =* 2.0 Hz, 3H, O-CH_3_), 3.59 – 3.52 (m, 1H, Phenyl-CH_2_), 3.46 (dt, *J =* 9.8, 4.9 Hz, 1H, Phenyl-CH_2_), 3.20 (d, *J =* 3.8 Hz, 2H, S-CH_2_), 2.94 – 2.70 (m, 2H, CH-CH_2_-N). ^13^C NMR (101 MHz, Chloroform-*d*) *δ* 149.5, 149.5, 149.2, 146.4, 142.8, 135.8, 124.8, 124.7, 122.7, 122.6, 122.6, 121.8, 117.8, 113.0, 112.9, 111.1, 108.3, 71.0, 69.3, 69.2, 66.5, 66.5, 56.3, 56.0, 51.9, 49.5, 46.8, 29.8, 28.8. HRMS (ESI) [M+H^+^] calcd for C_22_H_28_O_4_N_3_S^+^: 430.1795, found: 430.1784.

**BiTA9, 1-(((*R*)-3,4-dihydro-2H-benzo[4,5]imidazo[2,1-b][1,3]thiazin-3-yl)oxy)-3-((4-fluorobenzyl)(methyl)amino)propan-2-ol**

Yield, 79.52%, light yellow oily liquid. ^1^H NMR (500 MHz, Chloroform-*d*) *δ* 7.59 (d, *J =* 7.8 Hz, 1H, Phenyl-H), 7.34 – 7.31 (m, 1H, Phenyl-H), 7.30 (d, *J =* 3.2 Hz, 1H, Phenyl-H), 7.29 – 7.27 (m, 1H, Phenyl-H), 7.25 (d, *J =* 7.3 Hz, 1H, Phenyl-H), 7.23 – 7.19 (m, 1H, Phenyl-H), 7.18 – 7.13 (m, 2H, Phenyl-H), 4.27 (dtt, *J =* 9.2, 4.2, 2.1 Hz, 1H, CH-OH), 4.16 (ddd, *J =* 13.5, 9.4, 4.2 Hz, 1H, N-CH_2_-CH), 4.04 (ddd, *J =* 12.5, 6.6, 2.4 Hz, 1H, N-CH_2_-CH), 3.91 – 3.85 (m, 1H, CH-O), 3.68 – 3.59 (m, 2H, O-CH_2_), 3.54 – 3.41 (m, 2H, Phenyl-CH_2_), 3.29 – 3.12 (m, 2H, S-CH_2_), 2.52 (ddd, *J =* 12.2, 9.9, 2.2 Hz, 1H, CH-CH_2_-N), 2.33 (ddd, *J =* 12.3, 4.1, 2.0 Hz, 1H, CH-CH_2_-N), 2.23 (d, *J =* 10.2 Hz, 3H, N-CH_3_). ^13^C NMR (126 MHz, Chloroform-*d*) *δ* 163.3, 163.2, 160.9, 160.8, 146.2, 143.3, 135.8 134.0, 134.0, 130.7, 130.6, 122.5, 121.6, 118.1, 115.4, 115.2, 115.2, 115.0, 107.9, 71.9, 70.3, 66.9, 65.3, 61.9, 61.7, 61.5, 59.0, 46.8, 42.5, 42.1, 28.9. ^19^F NMR (471 MHz, Chloroform-*d*) *δ* -115.2, -115.7. HRMS (ESI) [M+H^+^] calcd for C_21_H_25_O_2_N_3_FS^+^: 402.1646, found: 402.1637.

**BiTA10, 1-(((*R*)-3,4-dihydro-2H-benzo[4,5]imidazo[2,1-b][1,3]thiazin-3-yl)oxy)-3-((3-fluorobenzyl)(methyl)amino)propan-2-ol**

Yield, 81.67%, light yellow oily liquid. ^1^H NMR (500 MHz, Chloroform-*d*) *δ* 7.59 (d, *J =* 7.7 Hz, 1H, Phenyl-H), 7.30 – 7.16 (m, 4H, Phenyl-H), 7.07 – 6.91 (m, 3H, Phenyl-H), 4.34 – 4.29 (m, 1H, CH-OH), 4.22 (dddd, *J =* 12.3, 11.1, 4.1, 1.0 Hz, 1H, N-CH_2_-CH), 4.10 (dt, *J =* 12.6, 6.1 Hz, 1H, N-CH_2_-CH), 3.89 (tdd, *J =* 9.5, 5.3, 3.8 Hz, 1H, CH-O), 3.75 – 3.59 (m, 2H, O-CH_2_), 3.58 – 3.41 (m, 2H, Phenyl-CH_2_), 3.34 – 3.19 (m, 2H, S-CH_2_), 2.51 (ddd, *J =* 12.1, 9.8, 1.9 Hz, 1H, CH-CH_2_-N), 2.35 (dt, *J =* 12.4, 4.3 Hz, 1H, CH-CH_2_-N), 2.24 – 2.21 (m, 3H, N-CH_3_). ^13^C NMR (126 MHz, Chloroform-*d*) *δ* 164.0, 162.0, 146.2, 143.4, 141.1, 141.1, 135.9, 130.0, 130.0, 129.9, 124.6, 124.6, 124.6, 124.6, 122.6, 121.7, 118.3, 115.9, 115.8, 115.7, 115.7, 114.4, 114.4, 114.3, 114.2, 107.9, 71.8, 71.6, 70.5, 70.4, 67.0, 66.9, 62.1, 59.2, 46.9, 46.7, 42.3, 42.3, 42.2, 29.1, 29.0. ^19^F NMR (471 MHz, CDCl_3_) *δ* -113.2, -113.2, -113.3, -113.3, -113.3. HRMS (ESI) [M+H^+^] calcd for C_21_H_25_O_2_N_3_FS^+^: 402.1646, found: 402.1636.

**BiTA11, 1-(((*R*)-3,4-dihydro-2H-benzo[4,5]imidazo[2,1-b][1,3]thiazin-3-yl)oxy)-3-((4-(trifluoromethyl)benzyl)amino)propan-2-ol**

Yield, 72.79%, light yellow oily liquid. ^1^H NMR (400 MHz, Chloroform-*d*) *δ* 7.61 – 7.47 (m, 3H, Phenyl-H), 7.36 (t, *J =* 9.3 Hz, 2H, Phenyl-H), 7.16 (dd, *J =* 17.3, 7.7 Hz, 3H, Phenyl-H), 4.25 (s, 1H, CH-OH), 4.13 (dd, *J =* 9.2, 4.5 Hz, 2H, N-CH_2_-CH), 3.90 (s, 1H, CH-O), 3.80 (dd, *J =* 13.3, 4.0 Hz, 2H, O-CH_2_), 3.68 – 3.51 (m, 2H, Phenyl-CH_2_), 3.34 – 3.16 (m, 2H, S-CH_2_), 2.76 – 2.59 (m, 2H, CH-CH_2_-N). ^13^C NMR (101 MHz, Chloroform-*d*) *δ* 177.2, 146.2, 143.0, 143.0, 142.1, 142.0, 135.8, 128.9, 128.8, 128.8, 128.8, 125.6, 125.6, 125.5, 122.8, 122.7, 121.8, 118.0, 108.0, 71.9, 71.8, 69.6, 69.5, 68.2, 68.2, 52.7, 50.9, 50.9, 46.8, 46.7, 29.8, 29.8, 28.9, 28.9. ^19^F NMR (376 MHz, Chloroform-*d*) *δ* -62.3, -62.3. HRMS (ESI) [M+H^+^] calcd for C_21_H_23_O_2_N_3_F_3_S^+^: 438.1458, found: 438.1446.

**BiTA12, 1-(((*R*)-3,4-dihydro-2H-benzo[4,5]imidazo[2,1-b][1,3]thiazin-3-yl)oxy)-3-((2-fluorobenzyl)amino)propan-2-ol**

Yield, 84.35%, light yellow oily liquid. ^1^H NMR (500 MHz, Chloroform-*d*) *δ* 7.63 – 7.53 (m, 1H, Phenyl-H), 7.26 – 7.11 (m, 5H, Phenyl-H), 7.09 – 6.97 (m, 2H, Phenyl-H), 4.24 (dqd, *J =* 10.2, 4.1, 2.1 Hz, 1H, CH-OH), 4.15 (dddd, *J =* 12.6, 5.0, 4.0, 0.9 Hz, 1H, N-CH_2_-CH), 4.08 (dt, *J =* 12.5, 6.2 Hz, 1H, N-CH_2_-CH), 3.85 (ddd, *J =* 8.0, 5.3, 4.0 Hz, 1H, CH-O), 3.79 (dd, *J =* 9.6, 3.1 Hz, 2H, O-CH_2_), 3.72 – 3.51 (m, 2H, Phenyl-CH_2_), 3.30 – 3.18 (m, 2H, S-CH_2_), 2.70 (dd, *J =* 12.1, 4.0 Hz, 1H, CH-CH_2_-N), 2.61 (ddd, *J =* 12.1, 7.8, 3.3 Hz, 1H, CH-CH_2_-N). ^13^C NMR (126 MHz, Chloroform-*d*) *δ* 162.4, 160.0, 146.2, 146.2, 143.1, 143.1, 135.8, 130.6, 130.6, 130.5, 130.5, 129.1, 129.1, 129.1, 129.0, 126.4, 126.4, 126.3, 126.3, 124.3, 124.2, 122.6, 121.7, 118.0, 118.0, 115.5, 115.5, 115.3, 115.3, 108.0, 72.1, 72.0, 70.1, 69.9, 68.7, 68.6, 51.0, 50.9, 47.1, 47.0, 47.0, 46.7, 28.9, 28.9. ^19^F NMR (471 MHz, Chloroform-*d*) *δ* -119.0, -119.1, -119.1, -119.1, -119.1. HRMS (ESI) [M+H^+^] calcd for C_20_H_23_O_2_N_3_FS^+^: 388.1490, found: 388.1476.

**BiTA13, 1-(((*R*)-3,4-dihydro-2H-benzo[4,5]imidazo[2,1-b][1,3]thiazin-3-yl)oxy)-3-((3-fluorobenzyl)amino)propan-2-ol**

Yield, 74.42%, light yellow oily liquid. ^1^H NMR (500 MHz, Chloroform-*d*) *δ* 7.54 (dd, *J =* 7.5, 1.4 Hz, 1H, Phenyl-H), 7.25 – 7.10 (m, 4H, Phenyl-H), 7.08 – 6.99 (m, 2H, Phenyl-H), 6.95 – 6.87 (m, 1H, Phenyl-H), 4.24 (dtt, *J =* 7.3, 4.7, 2.5 Hz, 1H, CH-OH), 4.11 (d, *J =* 4.8 Hz, 2H, N-CH_2_-CH), 3.92 – 3.87 (m, 1H, CH-O), 3.80 – 3.69 (m, 2H, O-CH_2_), 3.69 – 3.49 (m, 2H, Phenyl-CH_2_), 3.31 – 3.17 (m, 2H, S-CH_2_), 2.71 (dd, *J =* 12.2, 3.8 Hz, 1H, CH-CH_2_-N), 2.66 – 2.59 (m, 1H, CH-CH_2_-N). ^13^C NMR (126 MHz, Chloroform-*d*) *δ* 164.2, 161.7, 146.2, 143.1, 140.6, 135.8, 130.2, 130.2, 124.2, 124.2, 122.7, 121.8, 118.1, 115.6, 115.5, 115.3, 115.3, 114.7, 114.6, 114.5, 114.4, 108.1, 71.8, 69.8, 69.6, 68.3, 68.2, 52.7, 50.8, 50.7, 46.8, 28.9, 28.9. ^19^F NMR (471 MHz, Chloroform-*d*) *δ* -112.8, -112.8, -112.8, -112.8, -112.8, -112.9. HRMS (ESI) [M+H^+^] calcd for C_20_H_23_O_2_N_3_FS^+^: 388.1490, found: 388.1479.

**BiTA14, 1-(((*R*)-3,4-dihydro-2H-benzo[4,5]imidazo[2,1-b][1,3]thiazin-3-yl)oxy)-3-((4-fluorobenzyl)amino)propan-2-ol**

Yield, 74.76%, light yellow oily liquid. ^1^H NMR (500 MHz, Chloroform-*d*) *δ* 7.59 (ddt, *J =* 7.8, 2.0, 0.9 Hz, 1H, Phenyl-H), 7.25 – 7.13 (m, 5H, Phenyl-H), 7.03 – 6.91 (m, 2H, Phenyl-H), 4.27 (dtt, *J =* 7.1, 4.7, 2.5 Hz, 1H, CH-OH), 4.20 – 4.10 (m, 2H, N-CH_2_-CH), 3.85 (ddt, *J =* 7.6, 5.3, 3.8 Hz, 1H, CH-O), 3.74 – 3.52 (m, 4H, O-CH_2_, Phenyl-CH_2_), 3.33 – 3.22 (m, 2H, S-CH_2_), 2.70 (dd, *J =* 12.1, 3.9 Hz, 1H, CH-CH_2_-N), 2.61 (dt, *J =* 12.1, 7.5 Hz, 1H, CH-CH_2_-N). ^13^C NMR (126 MHz, Chloroform-*d*) *δ* 146.2, 143.2, 135.9, 130.8, 130.7, 130.6, 130.3, 122.7, 121.8, 121.8, 118.3, 118.2, 115.8, 115.8, 115.6, 115.6, 108.1, 108.0, 71.7, 69.8, 69.7, 67.9, 57.6, 52.3, 50.5, 46.8, 46.7, 29.8, 29.0. ^19^F NMR (471 MHz, Chloroform-*d*) *δ* -113.7, -113.8, -114.9, -115.2, -115.3. HRMS (ESI) [M+H^+^] calcd for C_20_H_23_O_2_N_3_FS^+^: 388.1490, found: 388.1479.

**BiTA15, 1-(((*R*)-3,4-dihydro-2H-benzo[4,5]imidazo[2,1-b][1,3]thiazin-3-yl)oxy)-3-((2-methylbenzyl)amino)propan-2-ol**

Yield, 77.75%, light yellow oily liquid. ^1^H NMR (500 MHz, DMSO-*d*_6_) *δ* 7.43 (ddd, *J =* 14.5, 5.9, 2.8 Hz, 2H, Phenyl-H), 7.24 (dt, *J =* 7.5, 3.8 Hz, 1H, Phenyl-H), 7.16 – 7.13 (m, 2H, Phenyl-H), 7.10 (d, *J =* 3.5 Hz, 3H, Phenyl-H), 4.35 – 4.30 (m, 1H, CH-OH), 4.30 – 4.20 (m, 2H, N-CH_2_-CH), 3.70 (h, *J =* 5.2 Hz, 1H, CH-O), 3.66 – 3.57 (m, 3H, O-CH_2,_ Phenyl-CH_2_), 3.56 – 3.50 (m, 1H, Phenyl-CH_2_), 3.47 (dd, *J =* 9.9, 5.5 Hz, 2H, S-CH_2_), 2.61 – 2.54 (m, 1H, CH-CH_2_-N), 2.47 (dd, *J =* 11.8, 6.9 Hz, 1H, CH-CH_2_-N), 2.25 (d, *J =* 6.9 Hz, 3H, Phenyl-CH_3_). ^13^C NMR (126 MHz, DMSO-*d*_6_) *δ* 146.3, 142.7, 138.5, 136.1, 135.9, 129.9, 128.3, 128.3, 126.6, 125.6, 121.9, 120.9, 117.1, 108.8, 71.5, 71.4, 68.7, 68.6, 68.2, 52.3, 52.3, 50.8, 46.4, 46.2, 28.2, 28.1, 18.7. HRMS (ESI) [M+H^+^] calcd for C_21_H_26_O_2_N_3_S^+^: 384.1740, found: 384.1732.

**BiTA16, 1-(((*R*)-3,4-dihydro-2H-benzo[4,5]imidazo[2,1-b][1,3]thiazin-3-yl)oxy)-3-(4-(pyridin-4-yl)piperazin-1-yl)propan-2-ol**

Yield, 57.02%, light yellow oily liquid. ^1^H NMR (500 MHz, Chloroform-*d*) *δ* 8.21 (d, *J =* 4.9 Hz, 2H, pyridinyl-H), 7.58 (d, *J =* 7.6 Hz, 1H, Phenyl-H), 7.23 – 7.12 (m, 3H, Phenyl-H), 6.60 (dd, *J =* 6.6, 4.9 Hz, 2H, pyridinly-H), 4.32 (td, *J =* 5.6, 4.8, 2.6 Hz, 1H, CH-OH), 4.24 – 4.13 (m, 2H, N-CH_2_-CH), 3.90 (dq, *J =* 8.9, 4.3 Hz, 1H, CH-O), 3.77 – 3.73 (m, 1H, O-CH_2_), 3.68 (d, *J =* 4.2 Hz, 1H, O-CH_2_), 3.58 (dd, *J =* 9.9, 5.6 Hz, 1H, -OH)**,** 3.37 – 3.20 (m, 6H, S-CH_2_, piperazine-H), 2.64 (dt, *J =* 10.7, 4.5 Hz, 2H, CH-CH_2_-N), 2.53 – 2.34 (m, 4H, piperazine-H). ^13^C NMR (126 MHz, Chloroform-*d*) *δ* 154.8, 149.3, 149.3, 146.0, 135.7, 122.4, 121.5, 118.0, 108.2, 107.8, 107.8, 71.6, 71.4, 69.9, 69.8, 66.3, 66.2, 60.2, 59.9, 52.6, 52.6, 46.5, 46.5, 45.8, 28.9, 28.9. HRMS (ESI) [M+H^+^] calcd for C_22_H_28_O_2_N_5_S^+^: 426.1958, found: 426.1947.

**BiTA17, 1-((3-chlorobenzyl)amino)-3-(((*R*)-3,4-dihydro-2H-benzo[4,5]imidazo[2,1-b][1,3]thiazin-3-yl)oxy)propan-2-ol**

Yield, 79.51%, light yellow oily liquid. ^1^H NMR (400 MHz, Chloroform-*d*) *δ* 7.57 (d, *J =* 7.7 Hz, 1H, Phenyl-H), 7.27 (s, 1H, Phenyl-H), 7.22 – 7.17 (m, 3H, Phenyl-H), 7.16 – 7.08 (m, 3H, Phenyl-H), 4.24 (dh, *J =* 7.0, 2.7 Hz, 1H, CH-OH), 4.12 (d, *J =* 4.4 Hz, 2H, N-CH_2_-CH), 3.86 (dddd, *J =* 10.4, 8.3, 5.3, 2.8 Hz, 1H, CH-O), 3.74 – 3.67 (m, 2H, O-CH_2_), 3.67 – 3.52 (m, 2H, Phenyl-CH_2_), 3.30 – 3.18 (m, 2H, S-CH_2_), 2.68 (dd, *J =* 12.2, 4.0 Hz, 1H, CH-CH_2_-N), 2.64 – 2.56 (m, 1H, CH-CH_2_-N). ^13^C NMR (101 MHz, Chloroform-*d*) *δ* 146.2, 146.2, 143.1, 143.1, 141.5, 141.5, 135.8, 134.3, 134.3, 129.9, 129.9, 128.4, 128.3, 127.5, 127.4, 126.5, 126.4, 122.7, 121.7, 118.1, 108.0, 72.0, 70.0, 69.8, 68.7, 68.7, 53.0, 51.1, 51.0, 46.7, 46.7, 29.0, 28.9. HRMS (ESI) [M+H^+^] calcd for C_20_H_23_O_2_N_3_ClS^+^: 404.1194, found: 404.1185.

**BiTA18, 1-((4-chlorobenzyl)amino)-3-(((*R*)-3,4-dihydro-2H-benzo[4,5]imidazo[2,1-b][1,3]thiazin-3-yl)oxy)propan-2-ol**

Yield, 83.18%, light yellow oily liquid. ^1^H NMR (400 MHz, Chloroform-*d*) *δ* 7.60 (dd, *J =* 7.1, 1.7 Hz, 1H, Phenyl-H), 7.32 – 7.27 (m, 1H, Phenyl-H), 7.25 – 7.22 (m, 2H, Phenyl-H), 7.21 (d, *J =* 3.9 Hz, 1H, Phenyl-H), 7.19 (s, 1H, Phenyl-H), 7.18 (d, *J =* 1.3 Hz, 1H, Phenyl-H), 7.16 (d, *J =* 8.4 Hz, 1H, Phenyl-H), 4.30 (dtd, *J =* 6.8, 4.7, 4.1, 2.6 Hz, 1H, CH-OH), 4.25 – 4.14 (m, 2H, N-CH_2_-CH), 3.83 (d, *J =* 5.0 Hz, 1H, CH-O), 3.74 – 3.68 (m, 2H, O-CH_2_), 3.68 – 3.56 (m, 2H, Phenyl-CH_2_), 3.37 – 3.23 (m, 2H, S-CH_2_), 2.71 (dd, *J =* 12.1, 4.1 Hz, 1H, CH-CH_2_-N), 2.62 (dd, *J =* 12.1, 7.2 Hz, 1H, CH-CH_2_-N). ^13^C NMR (101 MHz, Chloroform-*d*) *δ* 146.0, 143.4, 138.3, 135.9, 133.0, 129.6, 129.6, 128.8, 128.7, 128.7, 128.6, 122.7, 121.8, 118.3, 107.9, 72.1, 70.1, 69.9, 68.9, 68.8, 53.1, 51.0, 46.9, 45.9, 29.9, 29.0. HRMS (ESI) [M+H^+^] calcd for C_20_H_23_O_2_N_3_ClS^+^: 404.1194, found: 404.1183.

**BiTA19, 1-(((*R*)-3,4-dihydro-2H-benzo[4,5]imidazo[2,1-b][1,3]thiazin-3-yl)oxy)-3-(dimethylamino)propan-2-ol**

Yield, 69.84%, light yellow oily liquid. ^1^H NMR (400 MHz, Chloroform-*d*) *δ* 7.59 (d, *J =* 7.3 Hz, 1H, Phenyl-H), 7.24 – 7.15 (m, 3H, Phenyl-H), 4.39 – 4.30 (m, 1H, CH-OH), 4.26 (dddd, *J =* 12.5, 8.4, 4.0, 0.9 Hz, 1H, N-CH_2_-CH), 4.15 (ddd, *J =* 12.5, 9.3, 6.3 Hz, 1H, N-CH_2_-CH), 3.87 (ddt, *J =* 12.3, 9.3, 3.9 Hz, 1H, CH-O), 3.75 – 3.51 (m, 2H, O-CH_2_), 3.40 – 3.24 (m, 2H, S-CH_2_), 2.43 (ddd, *J =* 12.3, 10.1, 6.3 Hz, 1H, CH-CH_2_-N), 2.28 (d, *J =* 6.2 Hz, 6H, N-(CH_3_)_2_), 2.27 – 2.21 (m, 1H, CH-CH_2_-N). ^13^C NMR (101 MHz, Chloroform-*d*) *δ* 143.8, 135.9, 122.6, 121.7, 119.5, 107.1, 72.0, 71.8, 70.4, 66.8, 66.7, 61.6, 61.5, 46.9, 46.7, 45.5, 29.1, 29.0. HRMS (ESI) [M+H^+^] calcd for C_15_H_22_O_2_N_3_S^+^: 308.1427, found: 308.1418.

**BiTA20, 1-(benzyl(isopropyl)amino)-3-(((*S*)-3,4-dihydro-2H-benzo[4,5]imidazo[2,1-b][1,3]thiazin-3-yl)oxy)propan-2-ol**

Yield, 80.25%, light yellow oily liquid. ^1^H NMR (400 MHz, Chloroform-*d*) *δ* 7.59 (d, *J =* 8.1 Hz, 1H, Phenyl-H), 7.34 – 7.26 (m, 4H, Phenyl-H), 7.25 – 7.14 (m, 4H, Phenyl-H), 4.26 (dt, *J =* 9.0, 2.6 Hz, 1H, CH-OH), 4.18 (ddd, *J =* 12.5, 8.1, 4.2 Hz, 1H, N-CH_2_-CH), 4.08 – 4.00 (m, 1H, N-CH_2_-CH), 3.69 (d, *J =* 2.9 Hz, 1H, CH-O), 3.68 – 3.58 (m, 2H, O-CH_2_), 3.54 – 3.45 (m, 2H, Phenyl-CH_2_), 3.31 – 3.20 (m, 1H, S-CH_2_), 3.19 – 3.08 (m, 1H, S-CH_2_), 3.00 – 2.89 (m, 1H, N-CH), 2.54 – 2.41 (m, 2H, CH-CH_2_-N), 1.10 – 0.97 (m, 6H, CH_3_). ^13^C NMR (101 MHz, Chloroform-*d*) *δ* 146.3, 146.2, 143.5, 143.5, 139.6, 135.9, 128.9, 128.8, 128.6, 128.6, 127.4, 127.3, 122.5, 121.6, 118.2, 118.2, 107.9, 71.9, 70.6, 70.5, 66.8, 66.7, 54.7, 54.5, 51.3, 51.3, 46.8, 46.7, 29.8, 29.1, 29.1, 20.3, 20.2, 15.8, 15.8. HRMS (ESI) [M+H^+^] calcd for C_23_H_30_O_2_N_3_S^+^: 412.2053, found: 412.2043.

**BiTA21, 1-(benzylamino)-3-(((*S*)-3,4-dihydro-2H-benzo[4,5]imidazo[2,1-b][1,3]thiazin-3-yl)oxy)propan-2-ol**

Yield, 68.77%, light yellow oily liquid. ^1^H NMR (400 MHz, Chloroform-*d*) *δ* 7.58 (d, *J =* 7.5 Hz, 1H, Phenyl-H), 7.34 – 7.26 (m, 5H, Phenyl-H), 7.19 (q, *J =* 6.2, 5.0 Hz, 3H, Phenyl-H), 4.26 (dt, *J =* 6.4, 3.0 Hz, 1H, CH-OH), 4.15 (d, *J =* 7.4 Hz, 2H, N-CH_2_-CH), 3.97 – 3.87 (m, 1H, CH-O), 3.86 – 3.73 (m, 2H, O-CH_2_), 3.70 – 3.50 (m, 2H, Phenyl-CH_2_), 3.30 – 3.20 (m, 2H, S-CH_2_), 2.79 – 2.59 (m, 2H, CH-CH_2_-N). ^13^C NMR (126 MHz, Chloroform-*d*) *δ* 146.2, 143.1, 137.7, 135.8, 128.7, 127.8, 122.7, 121.7, 118.1, 108.1, 71.8, 69.9, 69.8, 68.1, 53.2, 50.7, 46.8, 28.9. HRMS (ESI) [M+H^+^] calcd for C_20_H_24_O_2_N_3_S^+^: 370.1584, found: 370.1574.

**BiTA22: 1-(((*S*)-3,4-dihydro-2H-benzo[4,5]imidazo[2,1-b][1,3]thiazin-3-yl)oxy)-3-((3-methoxybenzyl)amino)propan-2-ol**

Yield, 61.35%, light yellow oily liquid. ^1^H NMR (400 MHz, Chloroform-*d*) *δ* 7.54 (d, *J =* 7.8 Hz, 1H, Phenyl-H), 7.21 – 7.09 (m, 4H, Phenyl-H), 6.93 – 6.71 (m, 4H, Phenyl-H), 4.20 (dq, *J =* 6.6, 3.3 Hz, 1H, CH-OH), 4.07 (d, *J =* 2.8 Hz, 2H, N-CH_2_-CH), 3.87 (h, *J =* 4.4 Hz, 1H, CH-O), 3.74 (s, 3H, O-CH_3_), 3.54 (ddq, *J =* 13.6, 9.6, 4.6, 3.7 Hz, 4H, O-CH_2_, Phenyl-CH_2_), 3.25 – 3.14 (m, 2H, S-CH_2_), 2.73 – 2.56 (m, 2H, CH-CH_2_-N). ^13^C NMR (101 MHz, Chloroform-*d*) *δ* 159.9, 159.8, 146.3, 146.2, 143.0, 140.0, 140.0, 135.8, 129.7, 129.6, 122.6, 121.7, 120.8, 120.7, 118.0, 114.1, 114.1, 112.9, 108.1, 71.9, 71.8, 69.9, 69.7, 68.4, 68.3, 55.5, 55.3, 53.3, 50.9, 50.8, 46.7, 45.9, 29.8, 28.9, 28.8. HRMS (ESI) [M+H^+^] calcd for C_21_H_24_O_3_N_3_S^+^: 400.1689, found: 400.1682.

**BiTA23, 1-(((*S*)-3,4-dihydro-2H-benzo[4,5]imidazo[2,1-b][1,3]thiazin-3-yl)oxy)-3-((3-fluorobenzyl)(methyl)amino)propan-2-ol**

Yield, 94.49%, light yellow oily liquid. ^1^H NMR (500 MHz, Chloroform-*d*) *δ* 7.54 (ddd, *J =* 12.7, 6.6, 3.3 Hz, 2H, Phenyl-H), 7.45 – 7.38 (m, 1H, Phenyl-H), 7.30 – 7.19 (m, 4H, Phenyl-H), 7.15 (td, *J =* 8.7, 2.6 Hz, 1H, Phenyl-H), 4.47 – 4.42 (m, 1H, CH-OH), 4.41 – 4.31 (m, 2H, N-CH_2_-CH), 3.87 (t, *J =* 7.1 Hz, 1H, CH-O), 3.66 (d, *J =* 5.1 Hz, 2H, O-CH_2_), 3.64 – 3.54 (m, 4H, Phenyl-CH_2_, S-CH_2_), 2.47 (td, *J =* 13.0, 5.6 Hz, 1H, CH-CH_2_-N), 2.42 – 2.34 (m, 1H CH-CH_2_-N), 2.19 (s, 3H, CH_3_). ^13^C NMR (101 MHz, DMSO-*d*_6_) *δ* 163.5, 161.1, 146.3, 142.7, 135.9, 130.1, 130.0, 124.7, 121.9, 120.9, 117.1, 115.4, 115.1, 113.8, 113.5, 108.8, 71.6, 71.5, 68.3, 68.3, 67.7, 67.6, 61.5, 60.1, 60.1, 46.4, 46.2, 42.7, 42.7, 28.3, 28.1. ^19^F NMR (376 MHz, DMSO-*d*_6_) *δ* -113.8, -113.8 HRMS (ESI) [M+H^+^] calcd for C_21_H_25_O_2_N_3_FS^+^: 402.1646, found: 402.1636.

**BiTA24, 1-(((*S*)-3,4-dihydro-2H-benzo[4,5]imidazo[2,1-b][1,3]thiazin-3-yl)oxy)-3-((4-(trifluoromethyl)benzyl)amino)propan-2-ol**

Yield, 62.02%, light yellow oily liquid. ^1^H NMR (500 MHz, Chloroform-*d*) *δ* 7.57 (dd, *J =* 8.0, 3.9 Hz, 1H, Phenyl-H), 7.54 – 7.49 (m, 2H, Phenyl-H), 7.34 (dd, *J =* 15.3, 8.0 Hz, 2H, Phenyl-H), 7.23 – 7.10 (m, 3H, Phenyl-H), 4.26 (dh, *J =* 6.9, 3.4, 2.9 Hz, 1H, CH-OH), 4.17 – 4.08 (m, 2H, N-CH_2_-CH), 3.88 – 3.83 (m, 1H, CH-O), 3.80 – 3.71 (m, 2H, O-CH_2_), 3.70 – 3.53 (m, 2H, Phenyl-CH_2_), 3.34 – 3.17 (m, 2H, S-CH_2_), 2.72 – 2.57 (m, 2H, CH-CH_2_-NH). ^13^C NMR (126 MHz, Chloroform-*d*) *δ* 146.1, 143.6, 143.0, 135.8, 128.5, 128.4, 125.4, 122.7, 121.7, 118.0, 108.0, 72.0, 69.7, 69.4, 68.7, 68.6, 53.1, 51.2, 51.2, 46.7, 46.6, 28.9, 28.8. ^19^F NMR (471 MHz, Chloroform-*d*) *δ* -62.2. HRMS (ESI) [M+H^+^] calcd for C_21_H_23_O_2_N_3_F_3_S^+^: 438.1458, found: 438.1445.

**BiTA25, 1-((3-chlorobenzyl)amino)-3-(((*S*)-3,4-dihydro-2H-benzo[4,5]imidazo[2,1-b][1,3]thiazin-3-yl)oxy)propan-2-ol**

Yield, 83.65%, light yellow oily liquid. ^1^H NMR (500 MHz, Chloroform-*d*) *δ* 7.57 (d, *J =* 7.9 Hz, 1H, Phenyl-H), 7.25 (d, *J =* 6.4 Hz, 1H, Phenyl-H), 7.19 (dd, *J =* 5.4, 2.4 Hz, 3H, Phenyl-H), 7.13 (dq, *J =* 11.4, 6.1, 4.6 Hz, 3H, Phenyl-H), 4.23 (dp, *J =* 9.9, 3.2 Hz, 1H, CH-OH), 4.17 – 4.03 (m, 2H, N-CH_2_-CH), 3.86 – 3.81 (m, 1H, CH-O), 3.73 – 3.51 (m, 4H, O-CH_2_, Phenyl-CH_2_), 3.28 – 3.18 (m, 2H, S-CH_2_), 2.67 (dd, *J =* 12.1, 4.0 Hz, 1H, CH-CH_2_-NH), 2.58 (ddd, *J =* 11.9, 7.6, 4.1 Hz, 1H, CH-CH_2_-NH). ^13^C NMR (101 MHz, Chloroform-*d*) *δ* 146.2, 143.0, 141.9, 135.7, 134.2, 129.8, 128.2, 127.3, 126.3, 122.6, 121.7, 118.0, 108.0, 72.0, 69.9, 69.7, 68.8, 68.7, 53.1, 51.1, 46.6, 28.8. HRMS (ESI) [M+H^+^] calcd for C_20_H_23_O_2_N_3_ClS^+^: 404.1194, found: 404.1184.

**BiTA26, 1-((4-chlorobenzyl)amino)-3-(((*S*)-3,4-dihydro-2H-benzo[4,5]imidazo[2,1-b][1,3]thiazin-3-yl)oxy)propan-2-ol**

Yield, 72.61%, light yellow oily liquid. ^1^H NMR (500 MHz, Chloroform-*d*) *δ* 7.55 (d, *J =* 7.8 Hz, 1H, Phenyl-H), 7.25 – 7.18 (m, 3H, Phenyl-H), 7.18 – 7.09 (m, 4H, Phenyl-H), 4.21 (h, *J =* 4.7, 4.3 Hz, 1H, CH-OH), 4.12 – 4.05 (m, 2H, N-CH_2_-CH), 3.84 (tq, *J =* 7.6, 4.3, 3.7 Hz, 1H, CH-O), 3.67 (dd, *J =* 11.6, 7.1 Hz, 2H, O-CH_2_), 3.64 – 3.59 (m, 1H, Phenyl-CH_2_), 3.53 (ddd, *J =* 27.7, 9.8, 4.9 Hz, 1H, Phenyl-CH_2_), 3.25 – 3.15 (m, 2H, S-CH_2_), 2.65 (dd, *J =* 12.1, 3.9 Hz, 1H, CH-CH_2_-NH), 2.61 – 2.54 (m, 1H, CH-CH_2_-NH). ^13^C NMR (126 MHz, Chloroform-*d*) *δ* 146.1, 142.9, 137.7, 135.7, 132.9, 132.8, 129.7, 129.7, 128.6, 122.6, 121.7, 117.9, 108.0, 71.9, 69.6, 69.4, 68.5, 68.4, 52.7, 51.0, 46.6, 28.7. HRMS (ESI) [M+H^+^] calcd for C_20_H_23_O_2_N_3_ClS^+^: 404.1194, found: 404.1185.

4. ^1^H NMR, ^13^C NMR, and HRMS Spectra


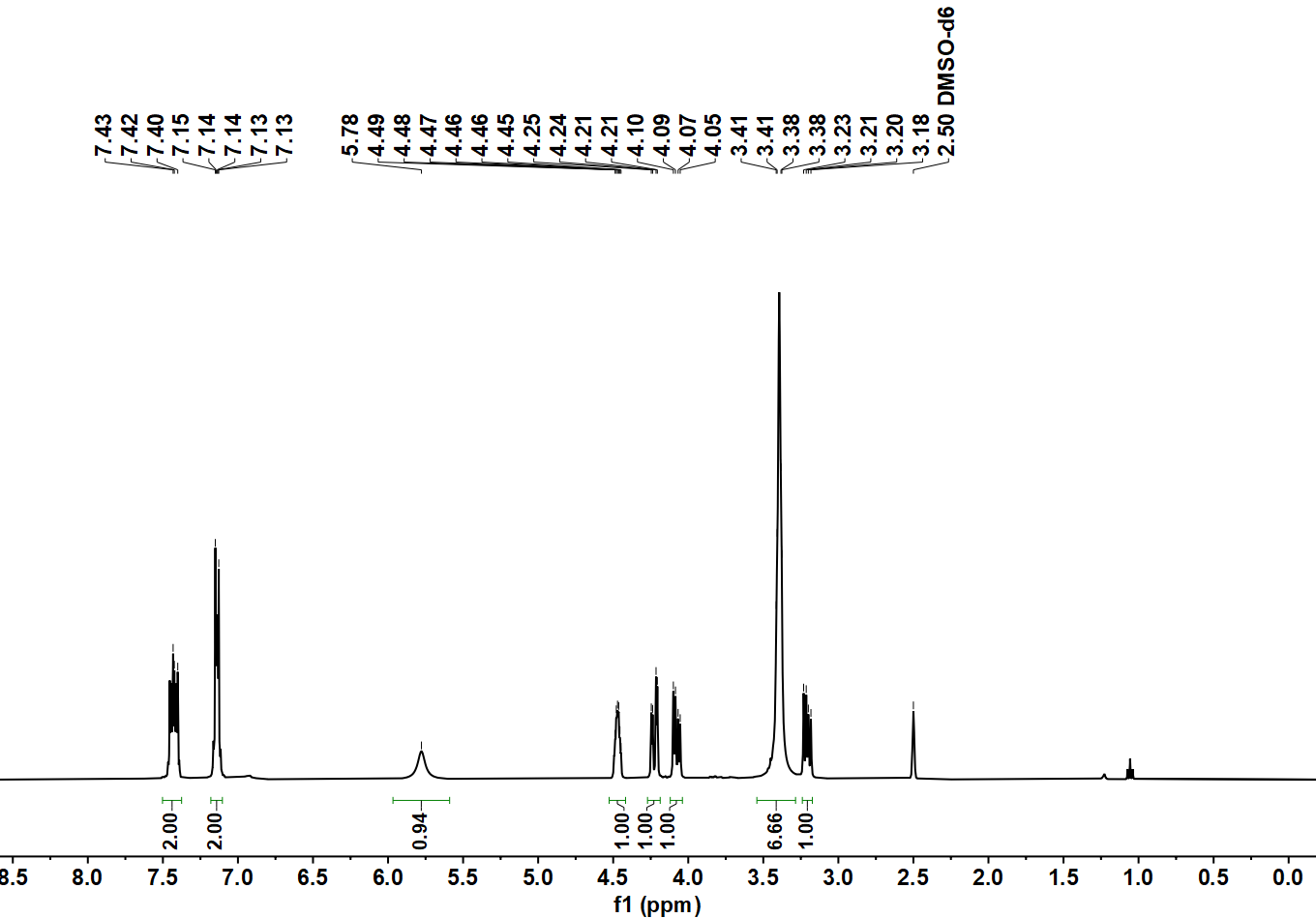


**Figure S21.** ^1^H NMR spectrum (400 MHz, DMSO-*d*_6_) of Intermediate 1


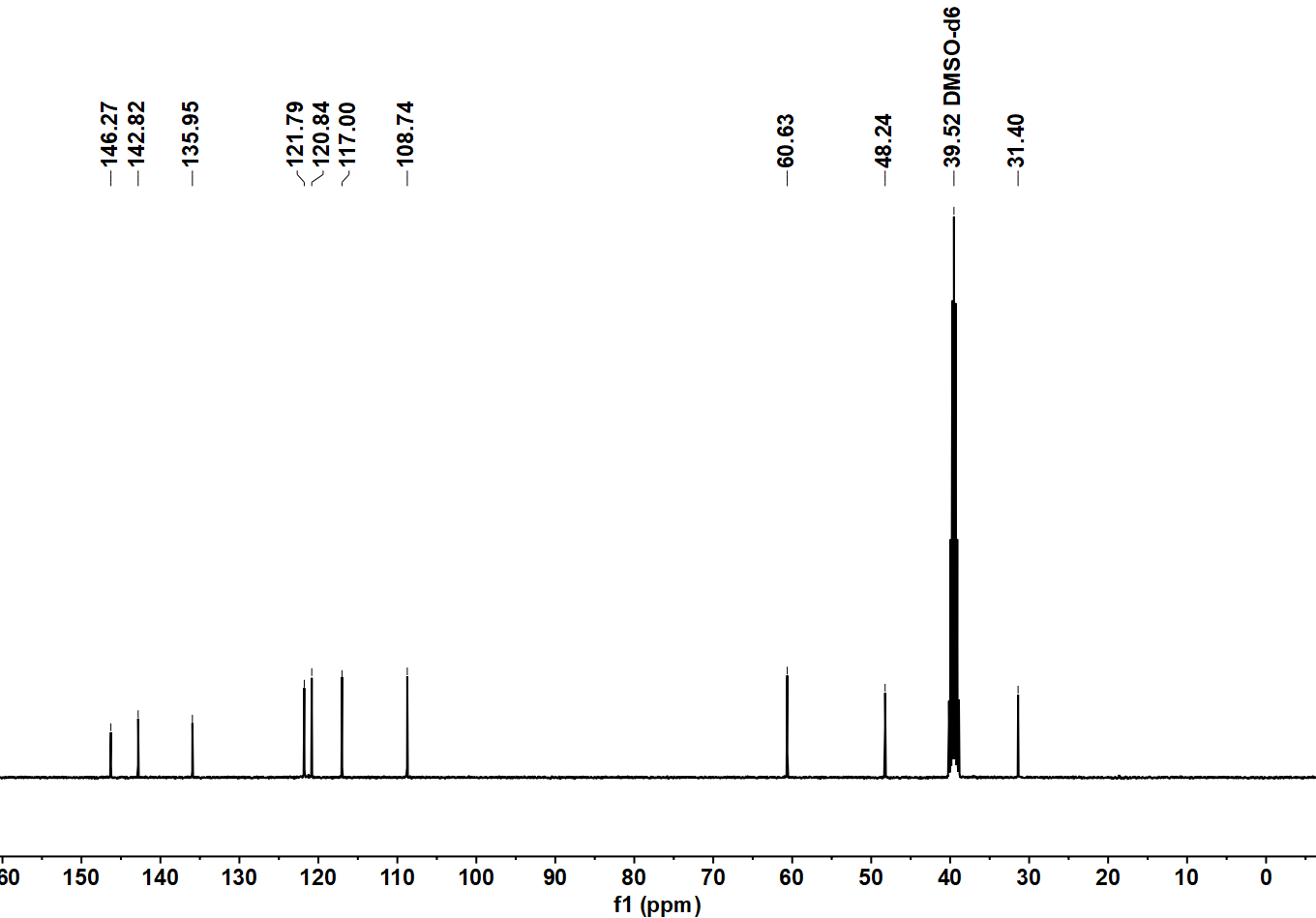


**Figure S22.** ^13^C NMR spectrum (101 MHz, DMSO-*d*_6_) of Intermediate 1


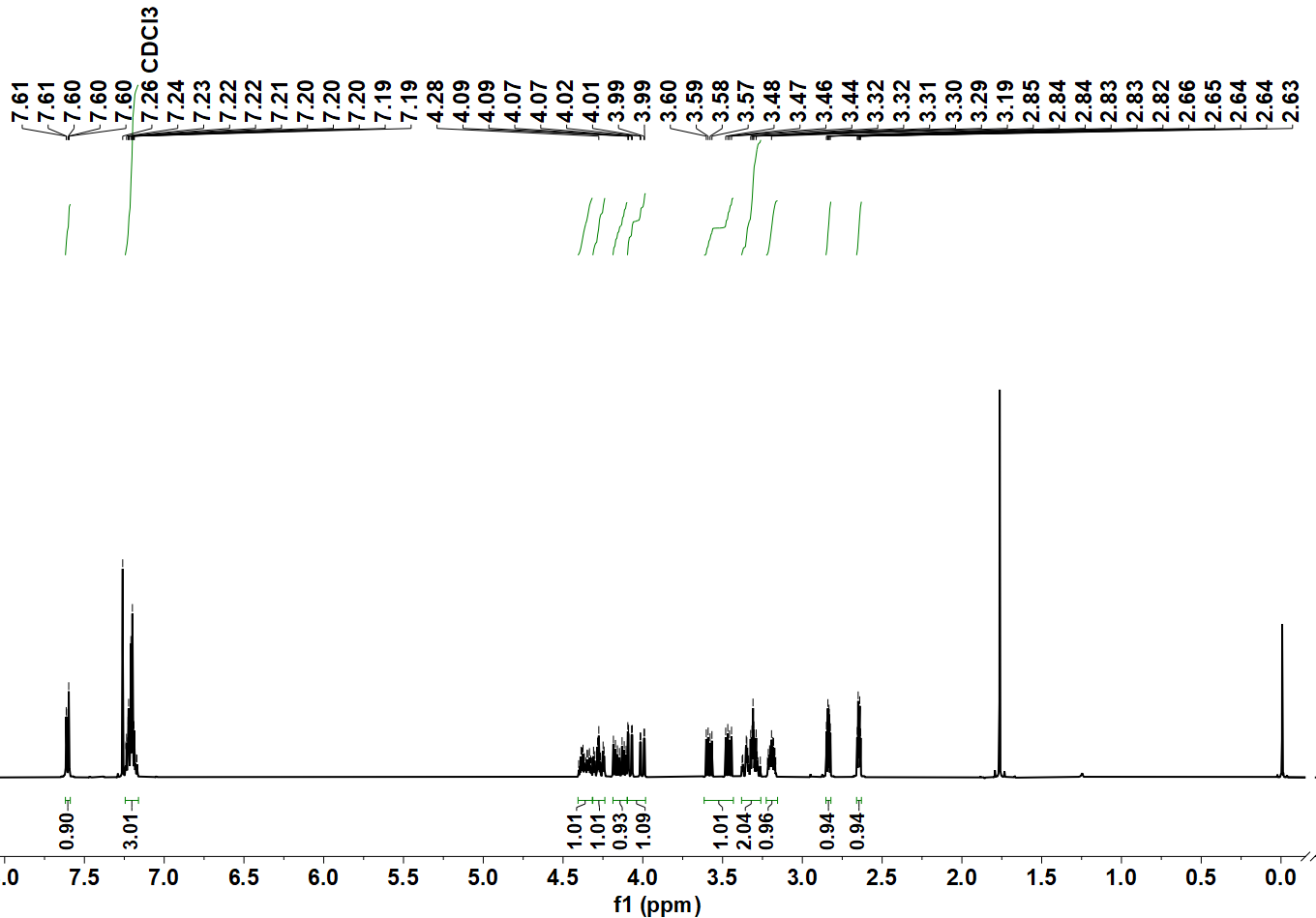


**Figure S23.** ^1^H NMR spectrum (500 MHz, Chloroform-*d*) of Intermediate 2


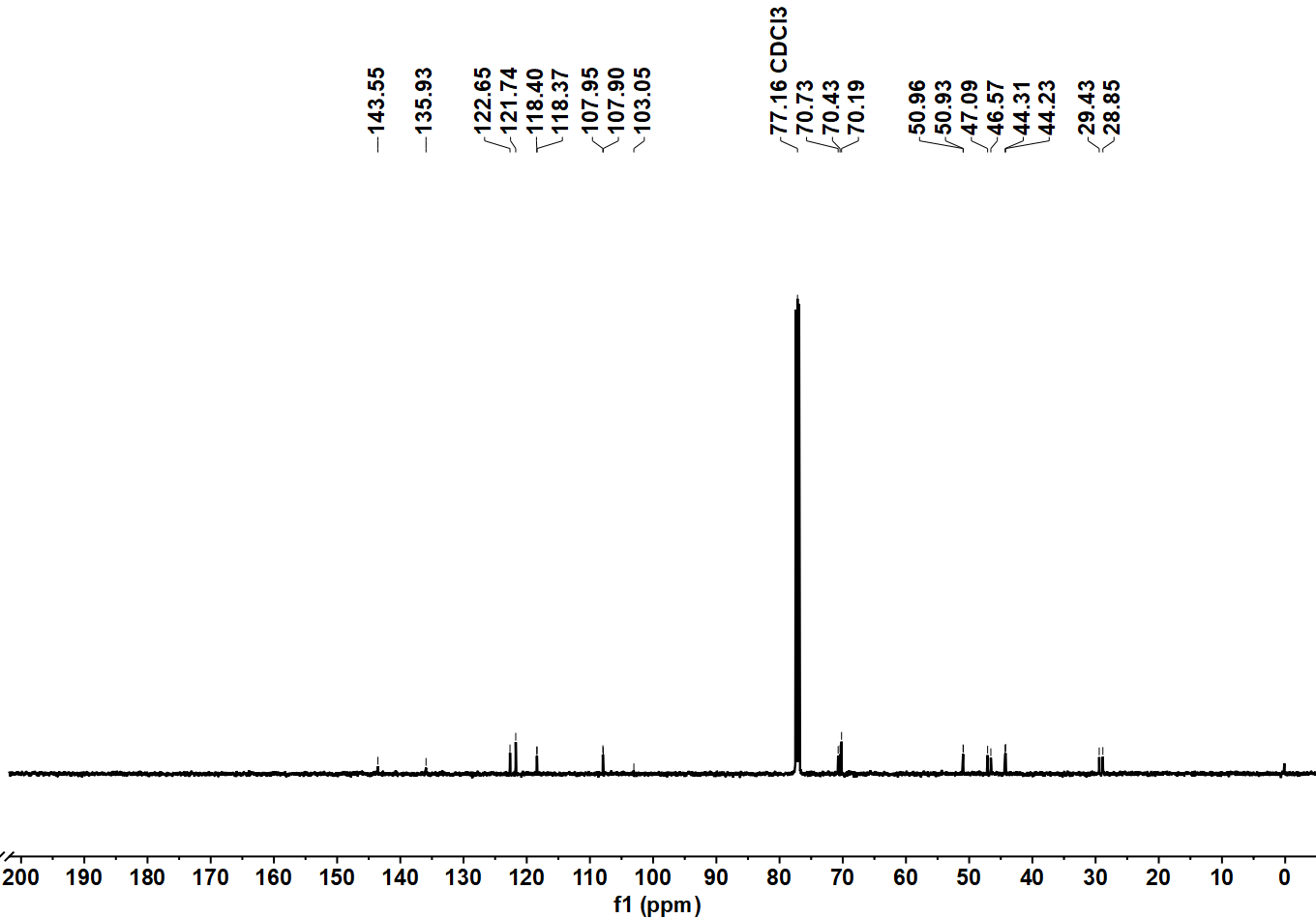


**Figure S24.** ^13^C NMR spectrum (126 MHz, Chloroform-*d*) of Intermediate 2

**
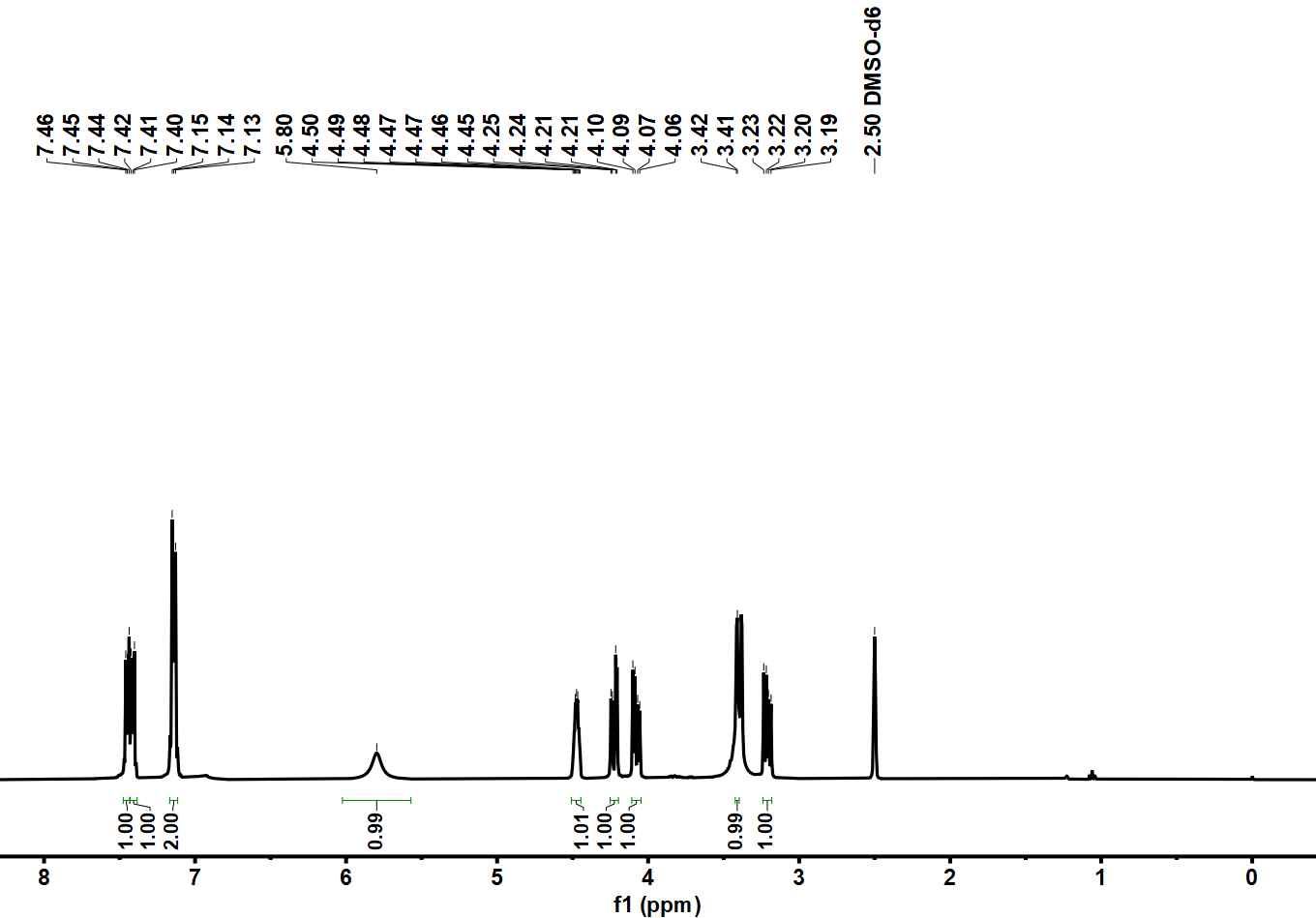
**

**Figure S25.** ^1^H NMR spectrum (400 MHz, DMSO-*d*_6_) of Intermediate 3

**
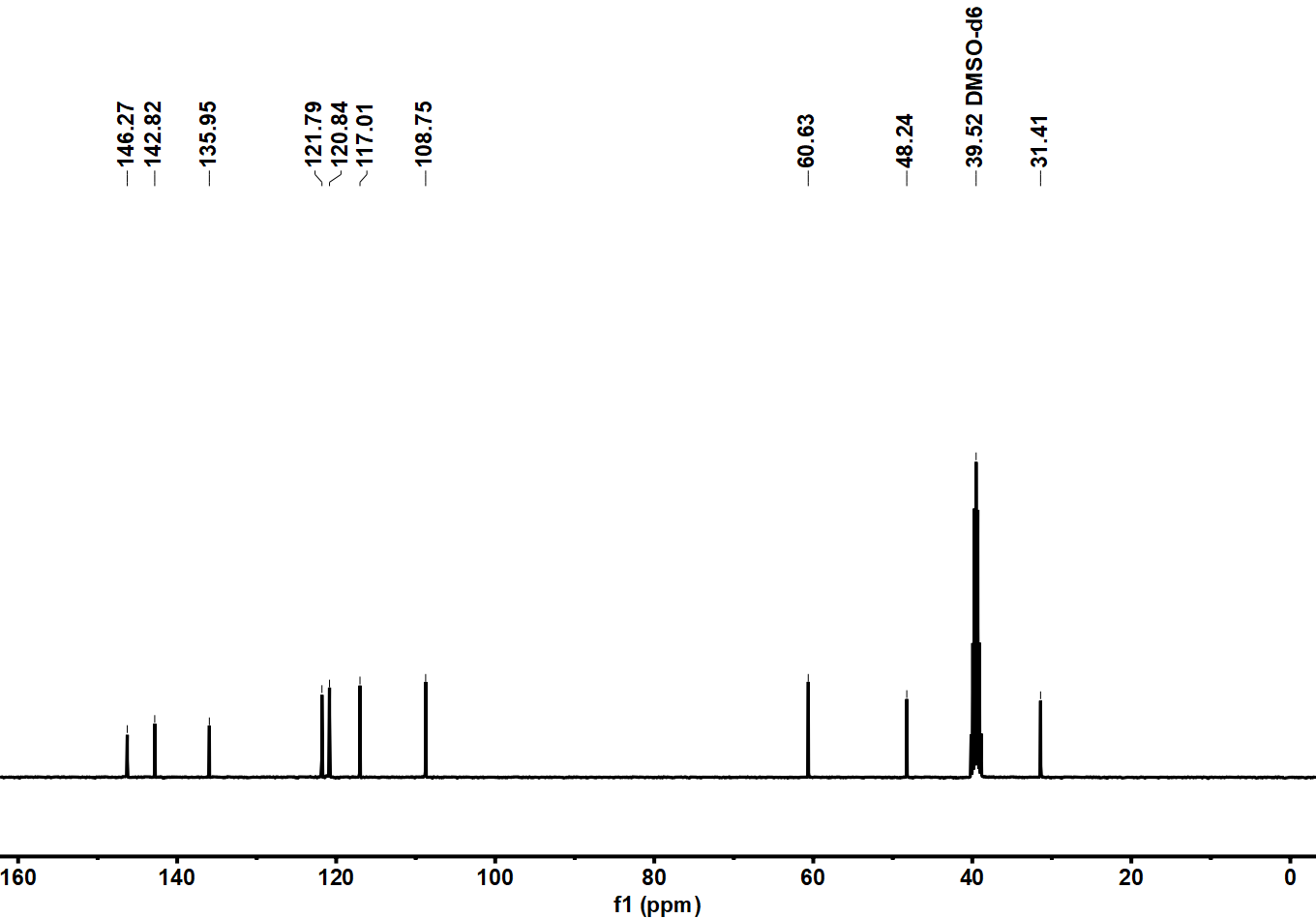
**

**Figure S26.** ^13^C NMR spectrum (101 MHz, DMSO-*d*_6_) of Intermediate 3


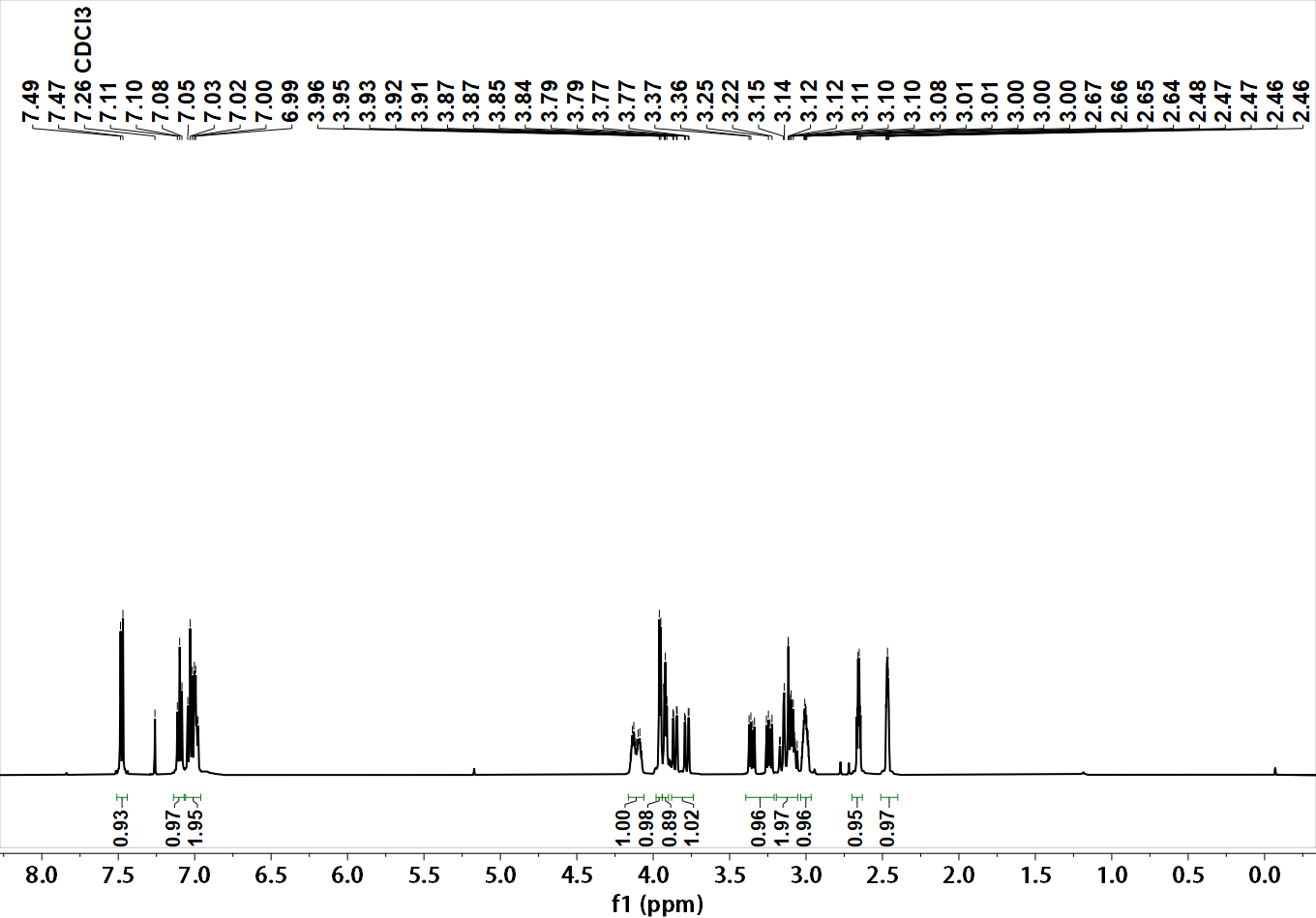


**Figure S27.** ^1^H NMR spectrum (500 MHz, Chloroform-*d*) of Intermediate 4


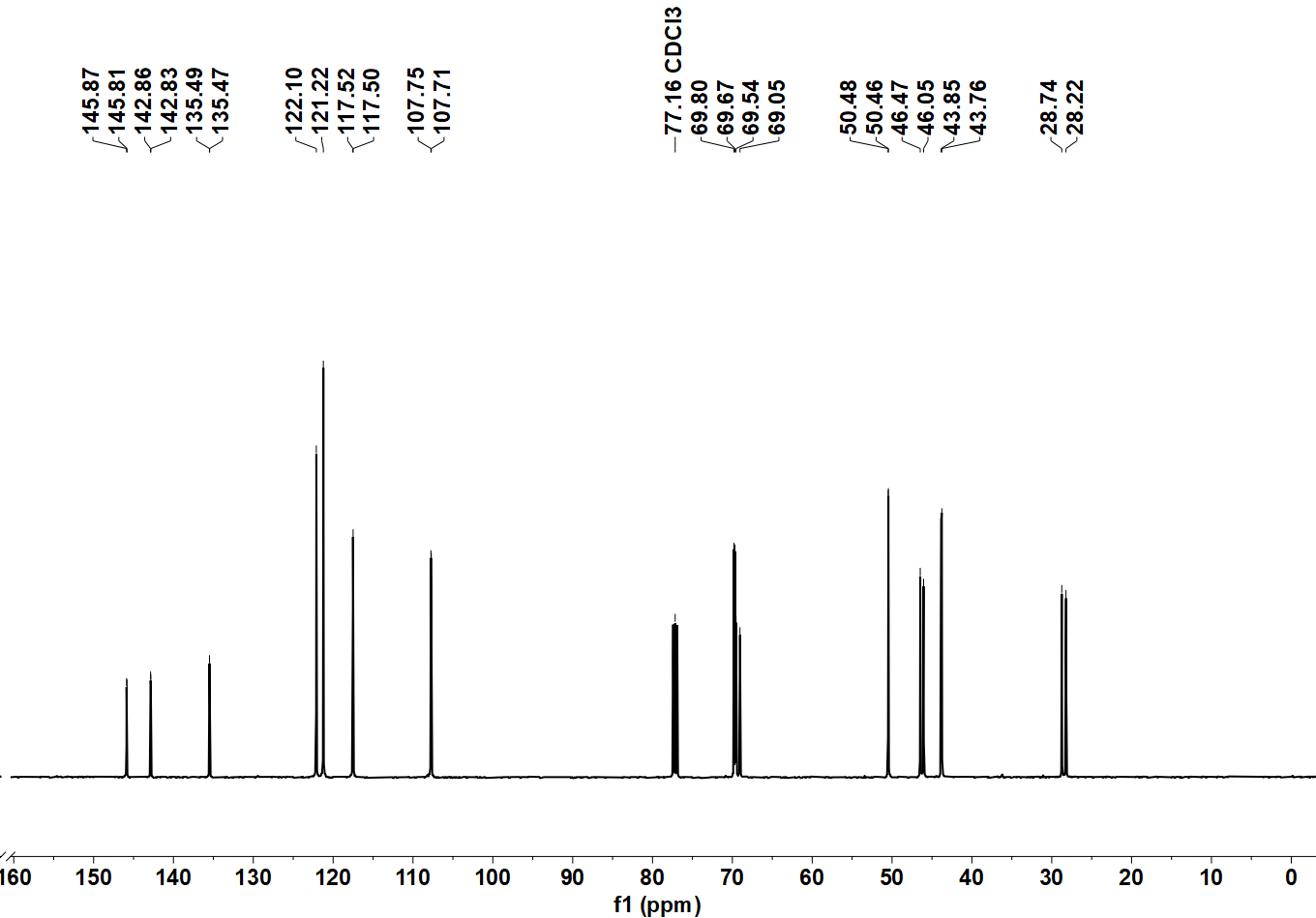


**Figure S28.** ^13^C NMR spectrum (126 MHz, Chloroform-*d*) of Intermediate 4


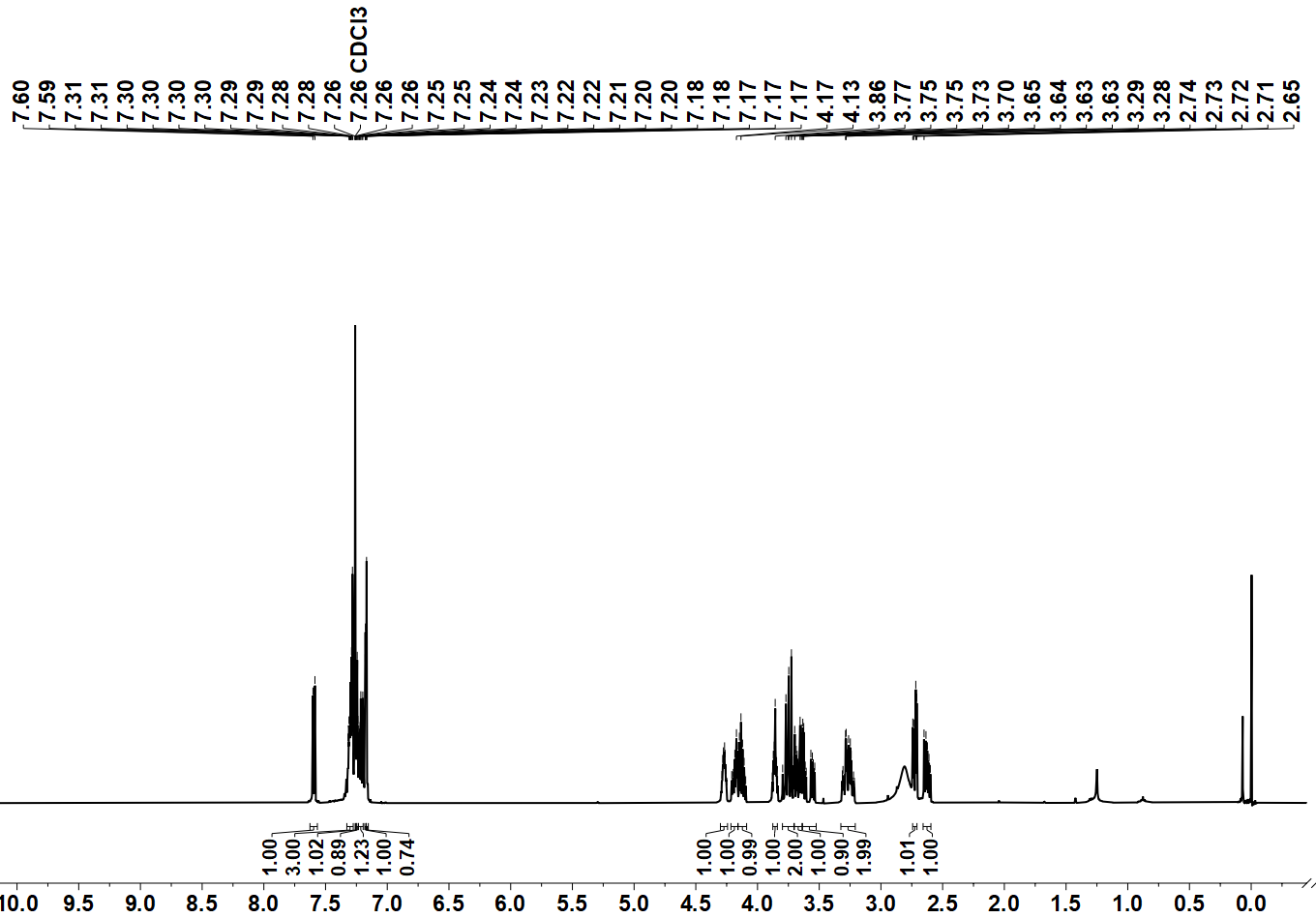


**Figure S29.** ^1^H NMR spectrum (500 MHz, Chloroform-*d*) of BiTA1


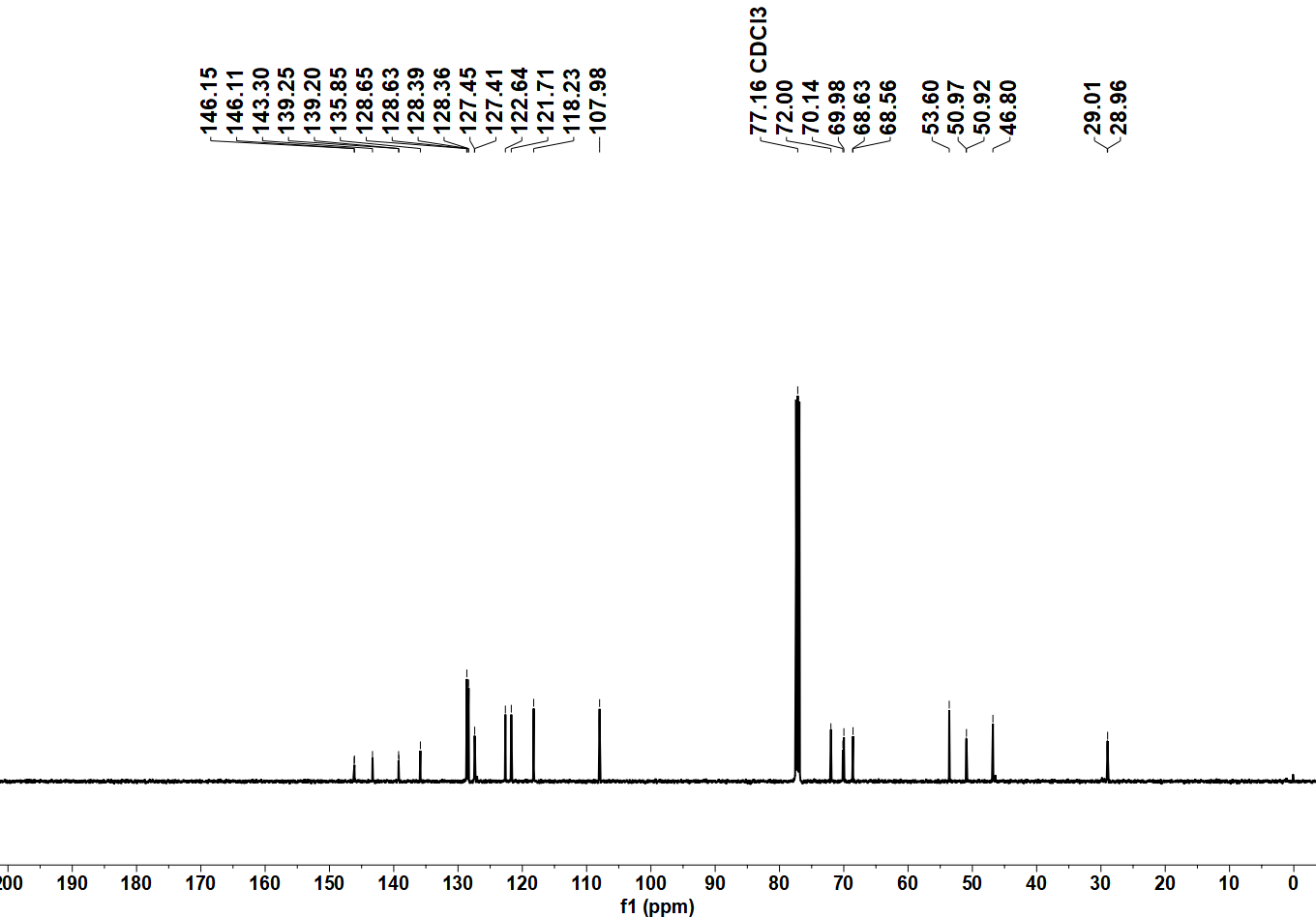


**Figure S30.** ^13^C NMR spectrum (126 MHz, Chloroform-*d*) of BiTA1

**Figure S31.** HRMS spectrum of BiTA1

**
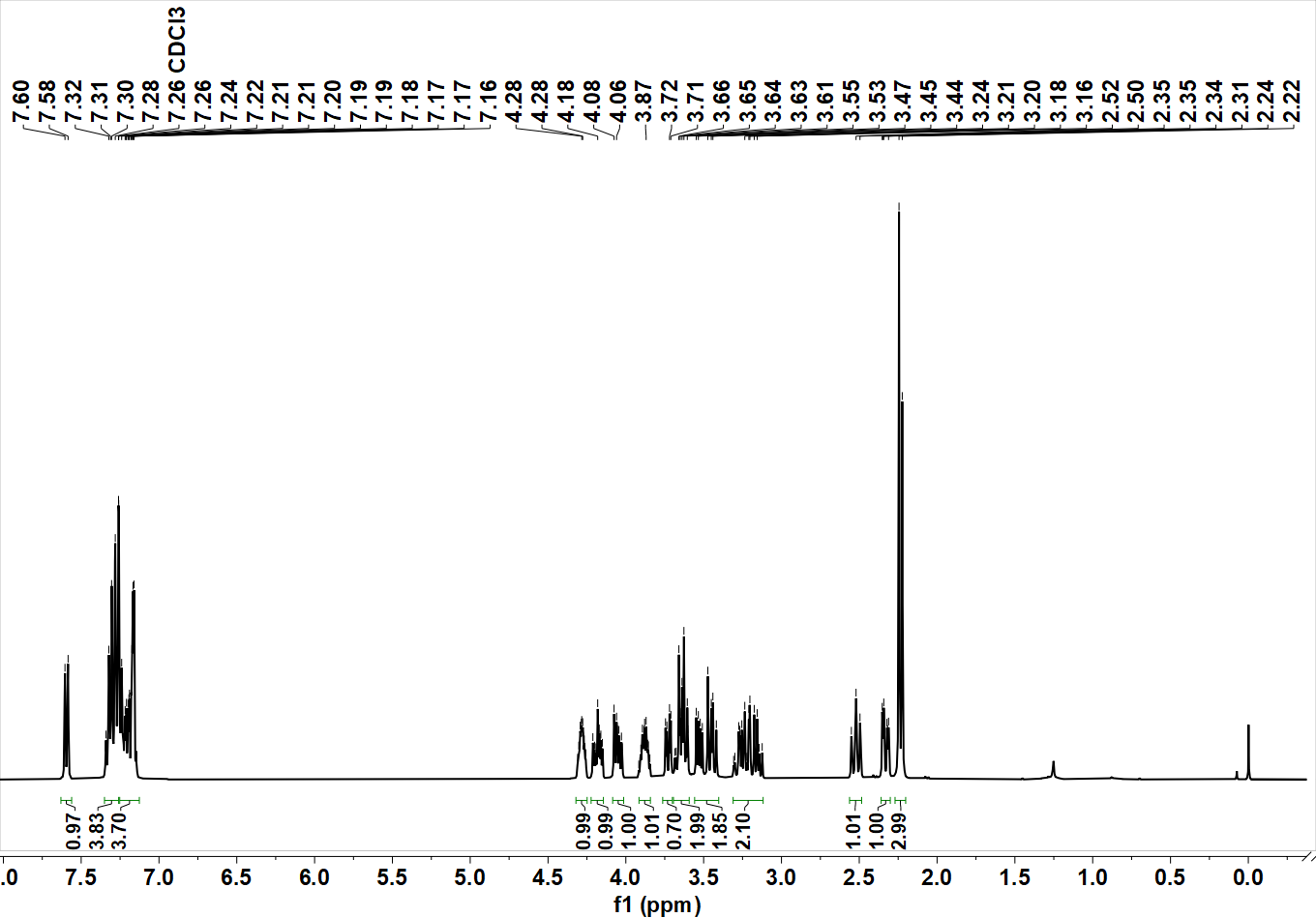
**

**Figure S32.** ^1^H NMR spectrum (400 MHz, Chloroform-*d*) of BiTA2

**
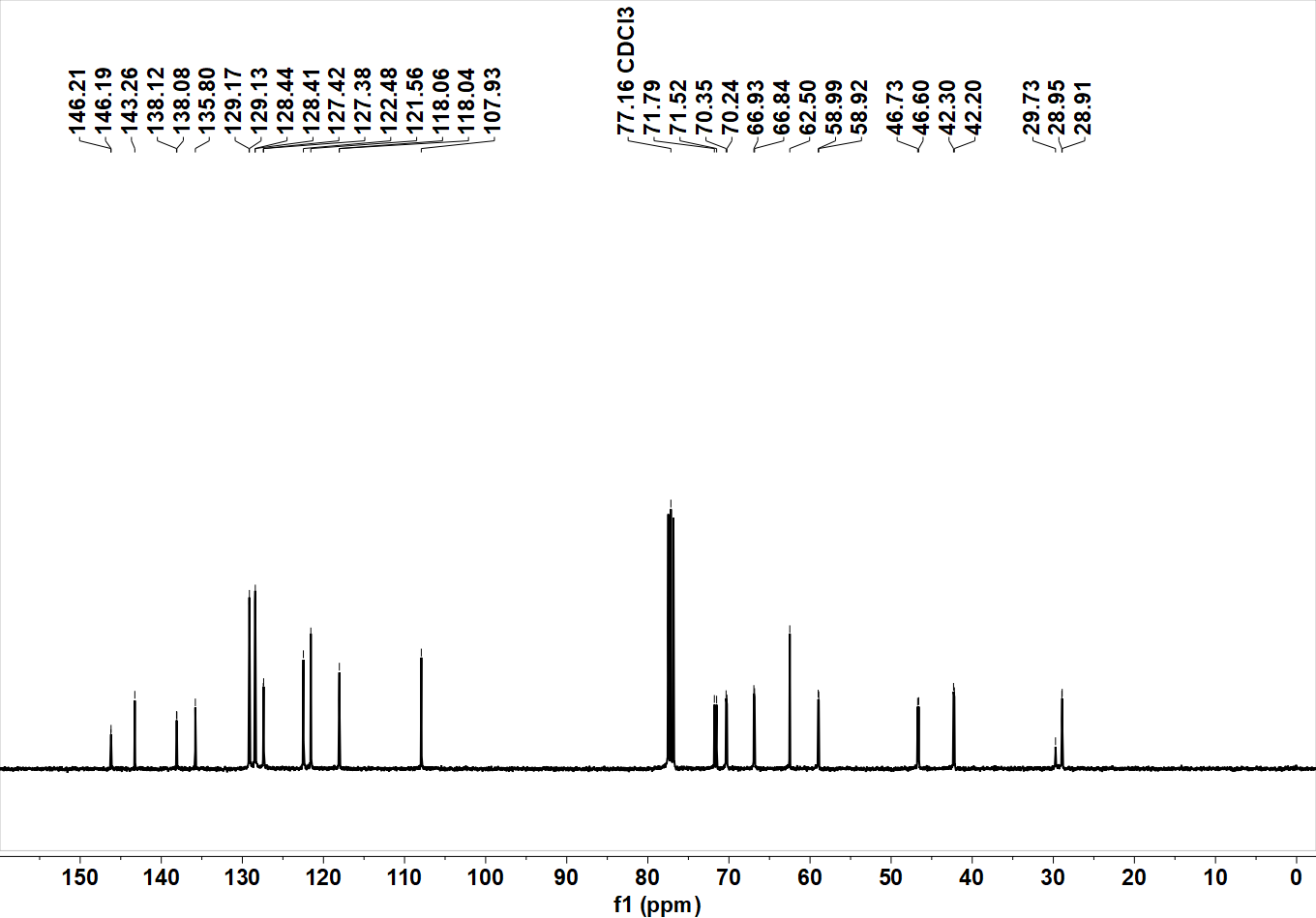
**

**Figure S33.** ^13^C NMR spectrum (101 MHz, Chloroform-*d*) of BiTA2

**Figure S34.** HRMS spectrum of BiTA2

**
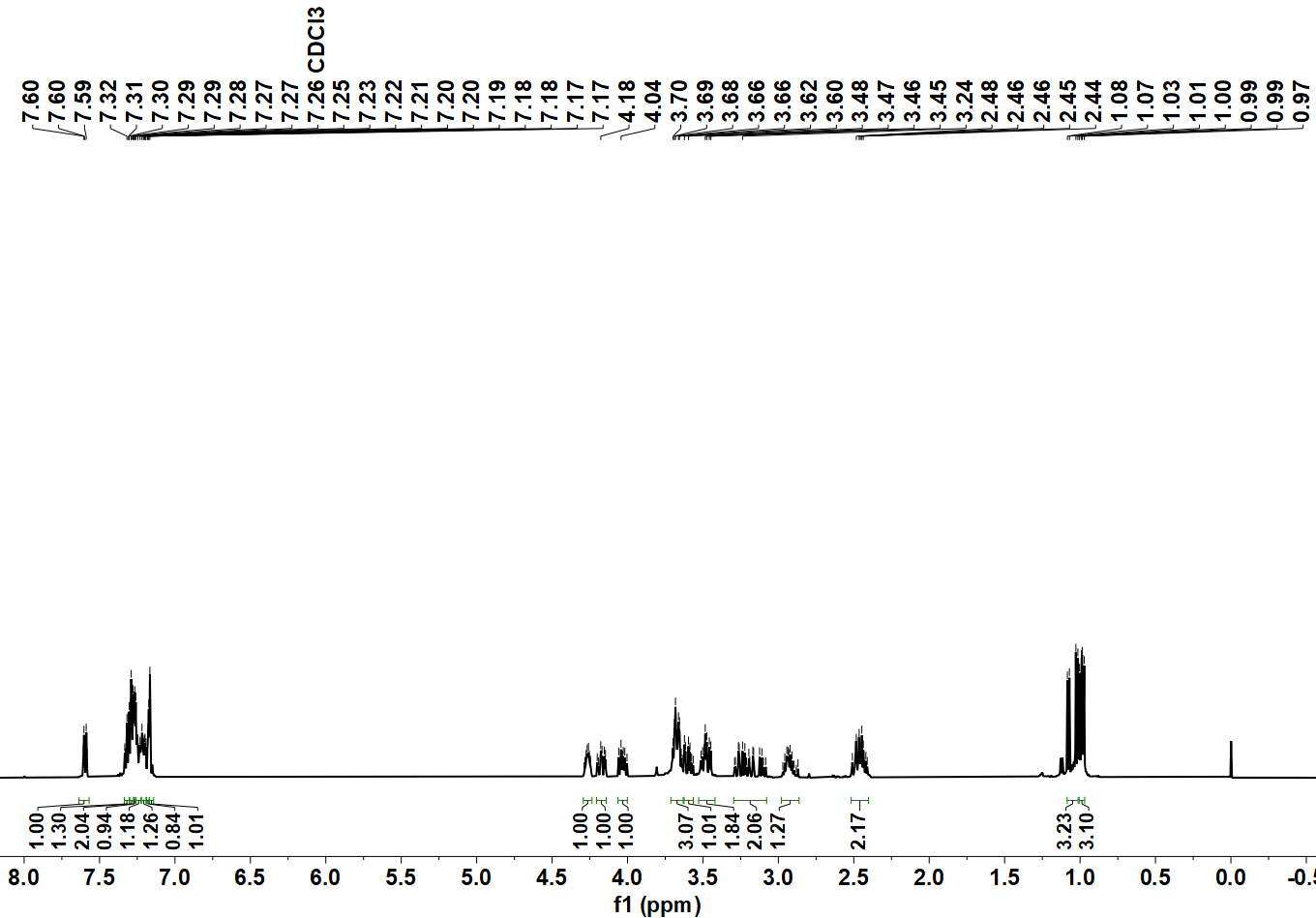
**

**Figure S35.** ^1^H NMR spectrum (500 MHz, Chloroform-*d*) of BiTA3

**
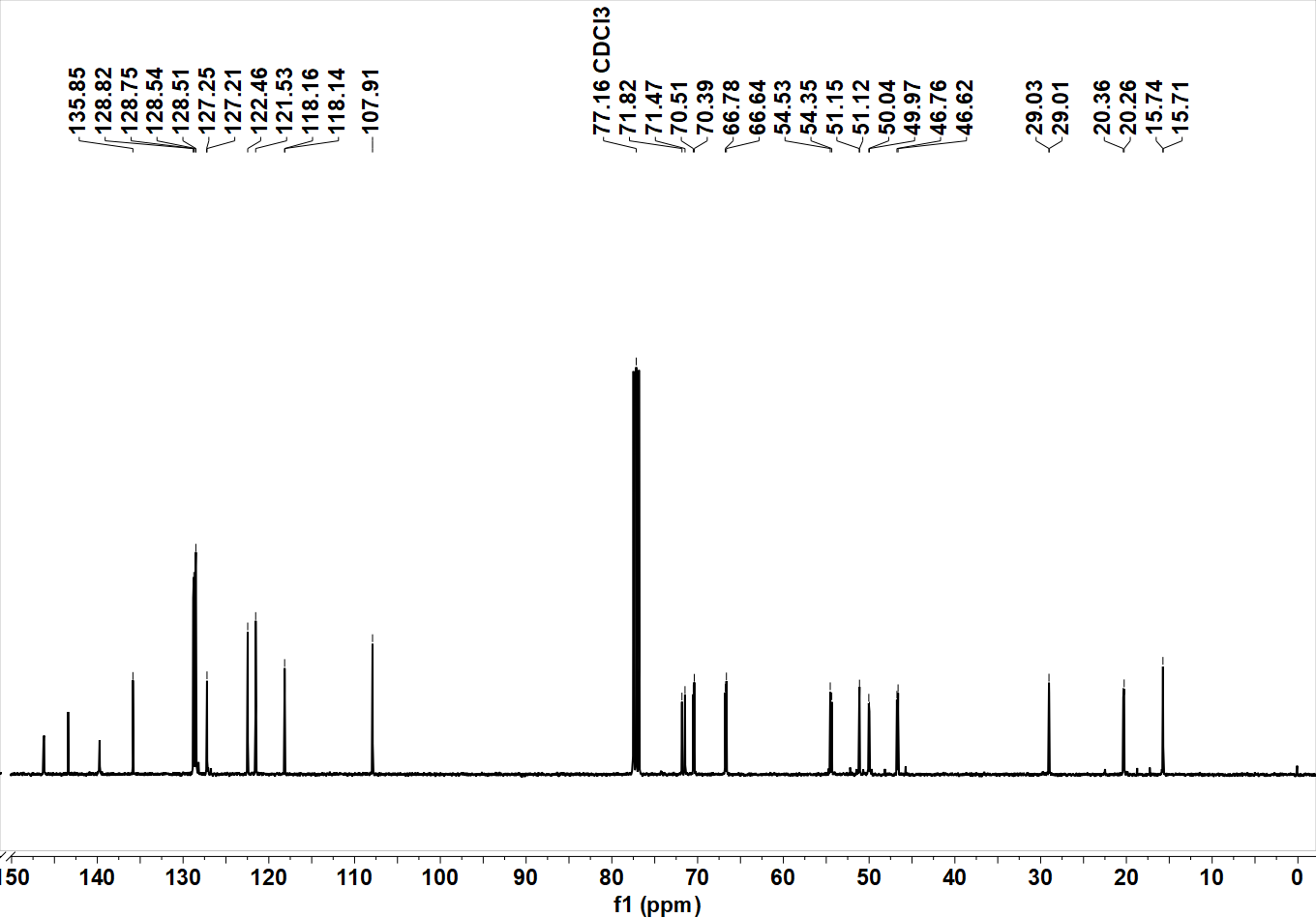
**

**Figure S36.** ^13^C NMR spectrum (126 MHz, Chloroform-*d*) of BiTA3

**Figure S37.** HRMS spectrum of BiTA3

**
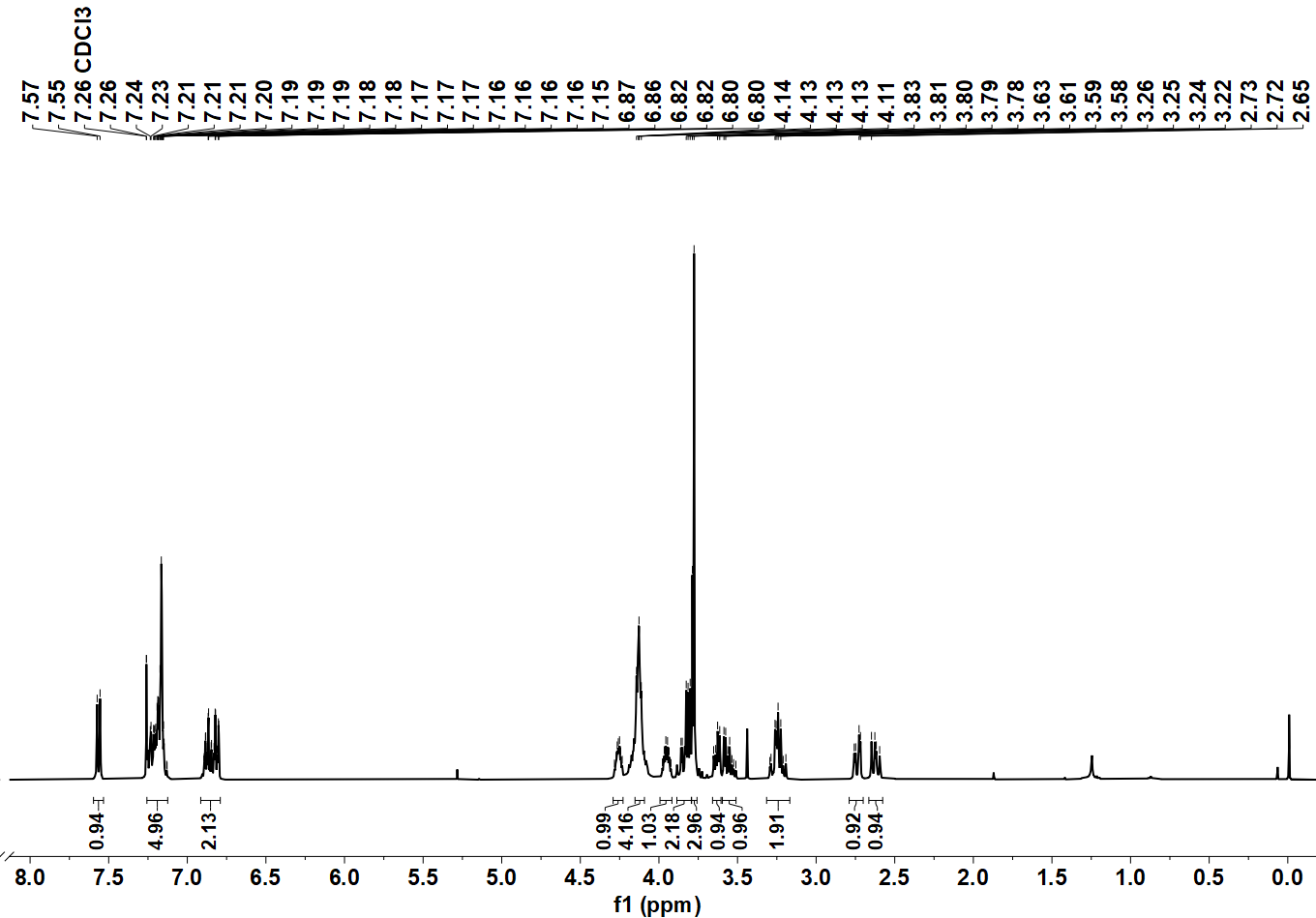
**

**Figure S38.** ^1^H NMR spectrum (400 MHz, Chloroform-*d*) of BiTA4

**
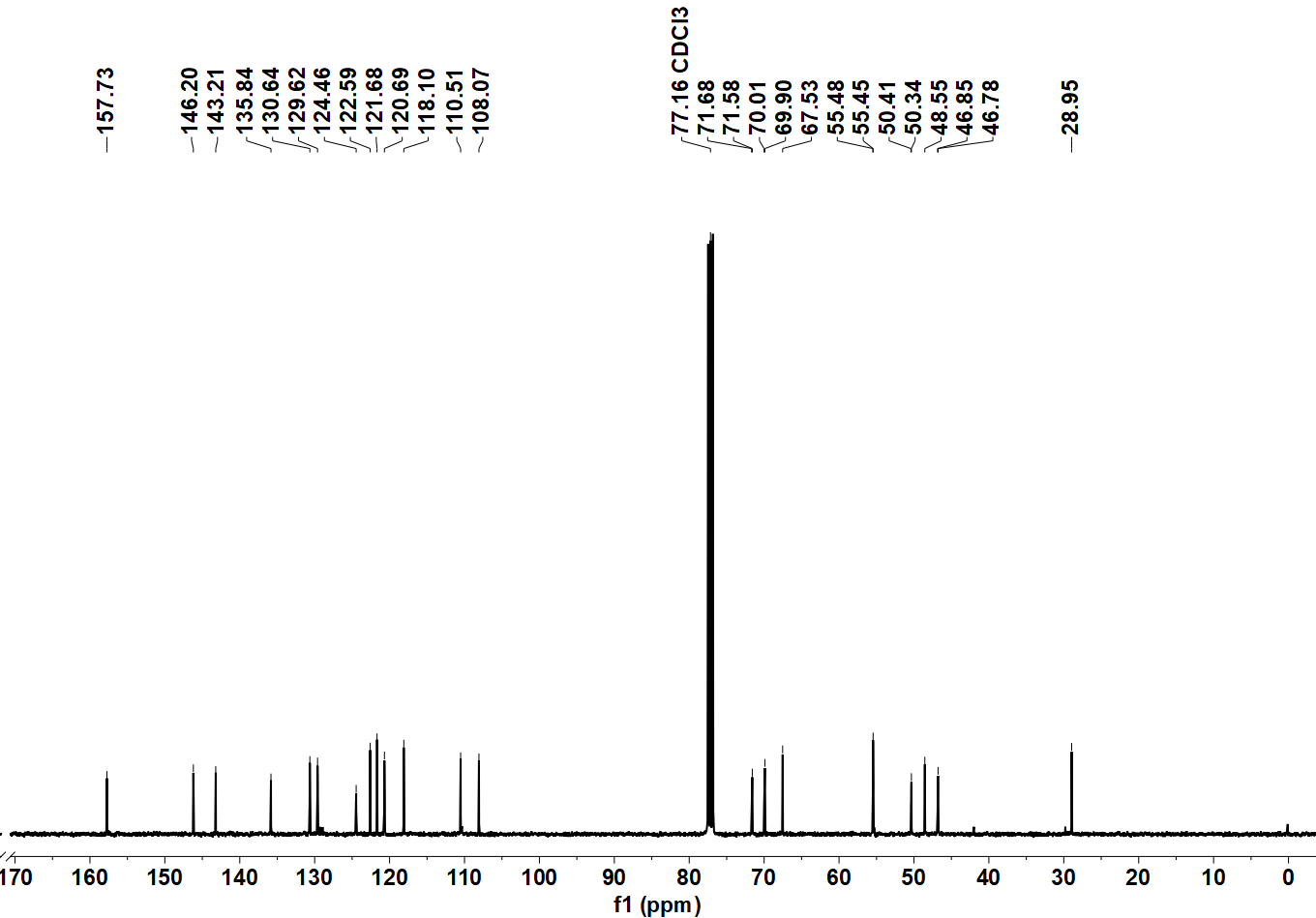
**

**Figure S39.** ^13^C NMR spectrum (101 MHz, Chloroform-*d*) of BiTA4

**Figure S40.** HRMS spectrum of BiTA4

**
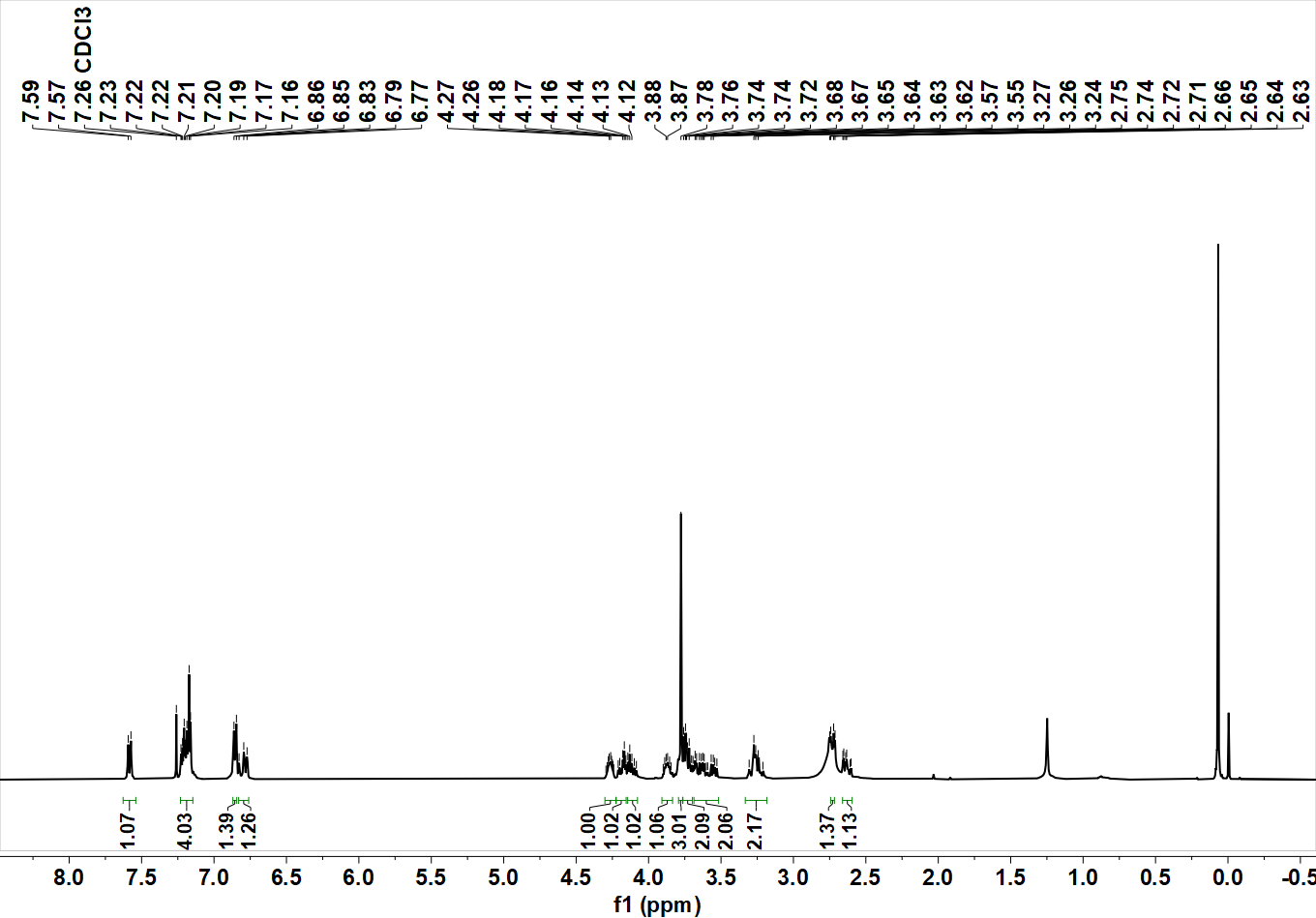
**

**Figure S41.** ^1^H NMR spectrum (400 MHz, Chloroform-*d*) of BiTA5

**
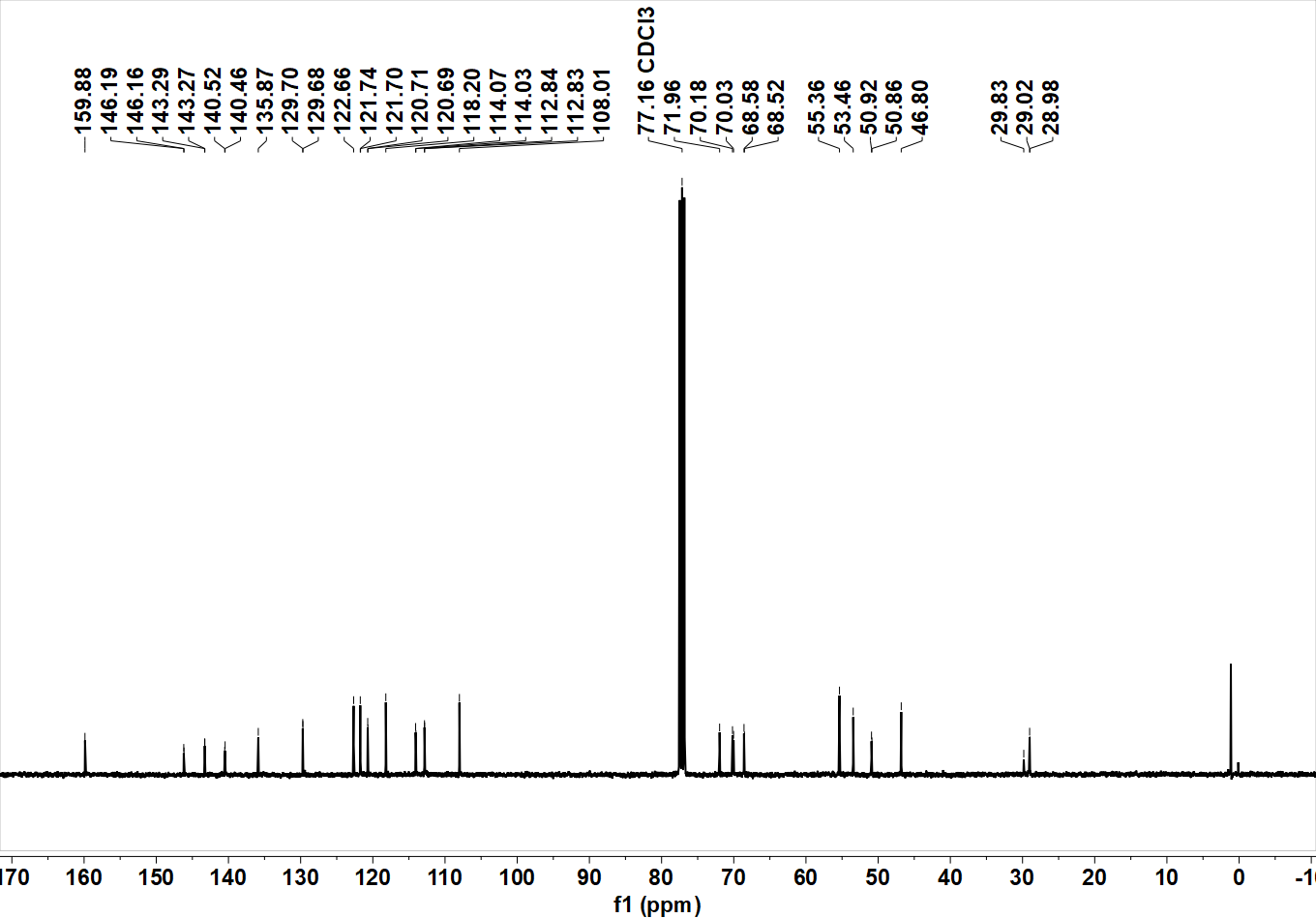
**

**Figure S42.** ^13^C NMR spectrum (101 MHz, Chloroform-*d*) of BiTA5

**Figure S43.** HRMS spectrum of BiTA5

**
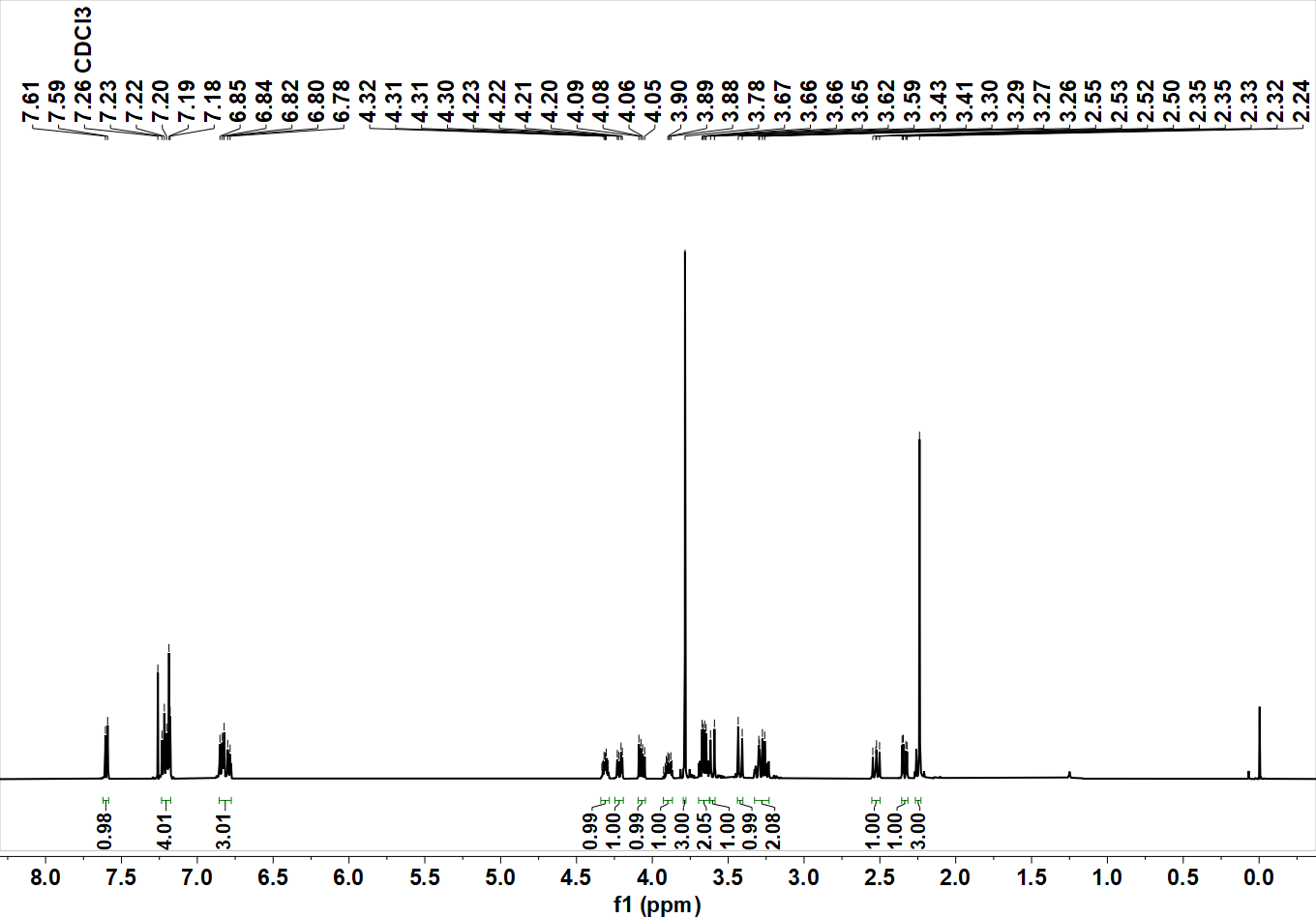
**

**Figure S44.** ^1^H NMR spectrum (500 MHz, Chloroform-*d*) of BiTA6

**
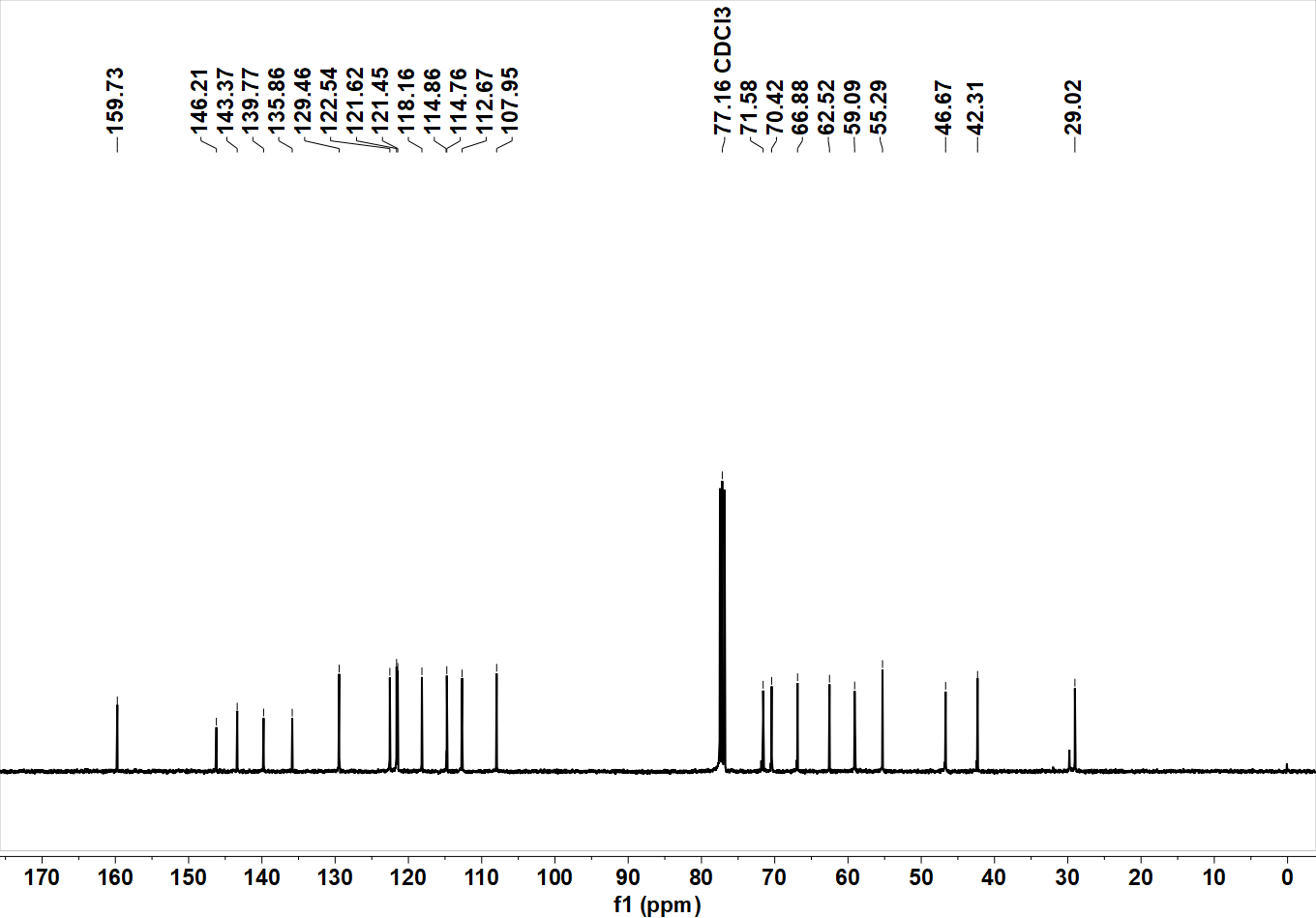
**

**Figure S45.** ^13^C NMR spectrum (126 MHz, Chloroform-*d*) of BiTA6

**Figure S46.** HRMS spectrum of BiTA6

**
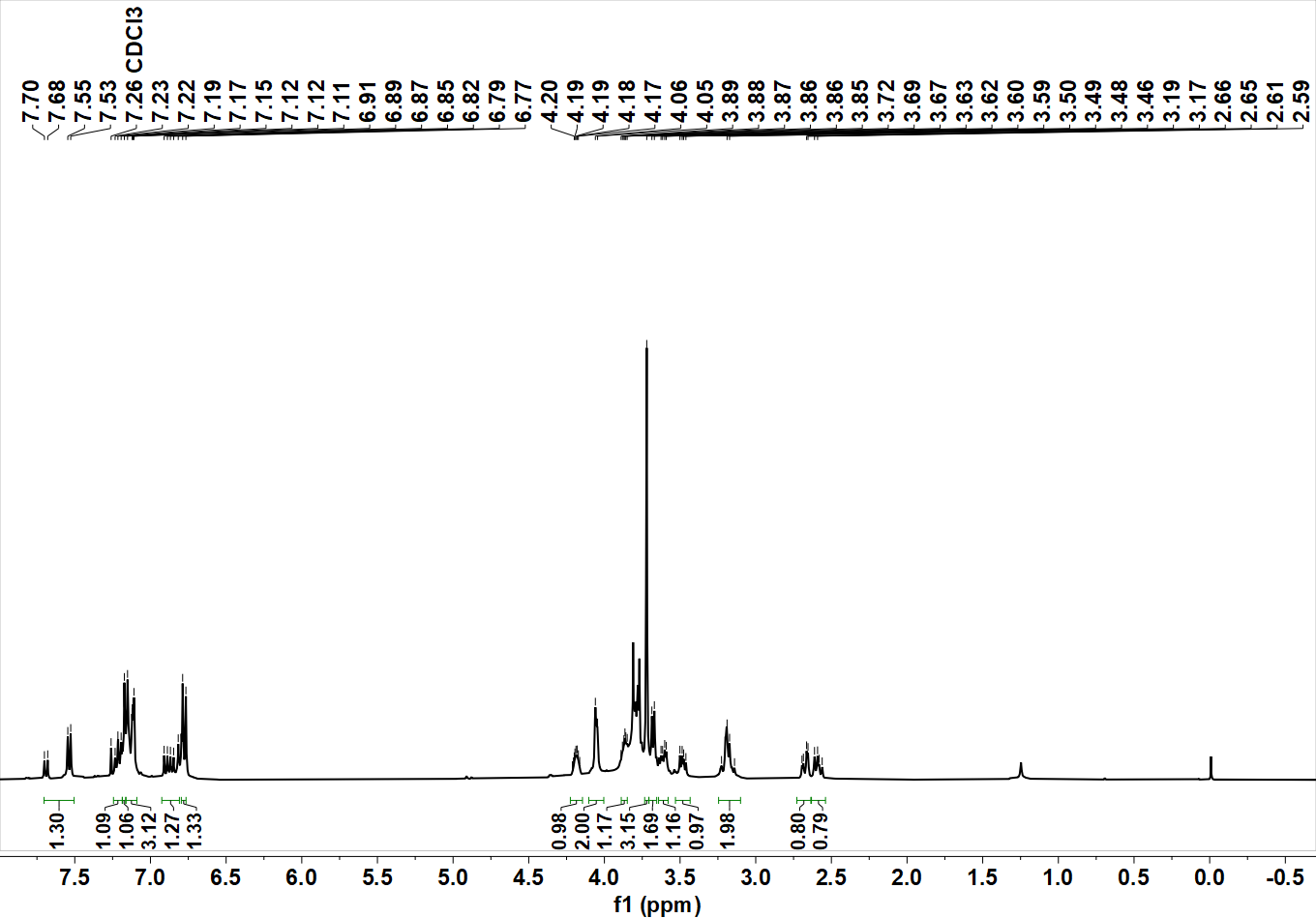
**

**Figure S47.** ^1^H NMR spectrum (400 MHz, Chloroform-*d*) of BiTA7

**
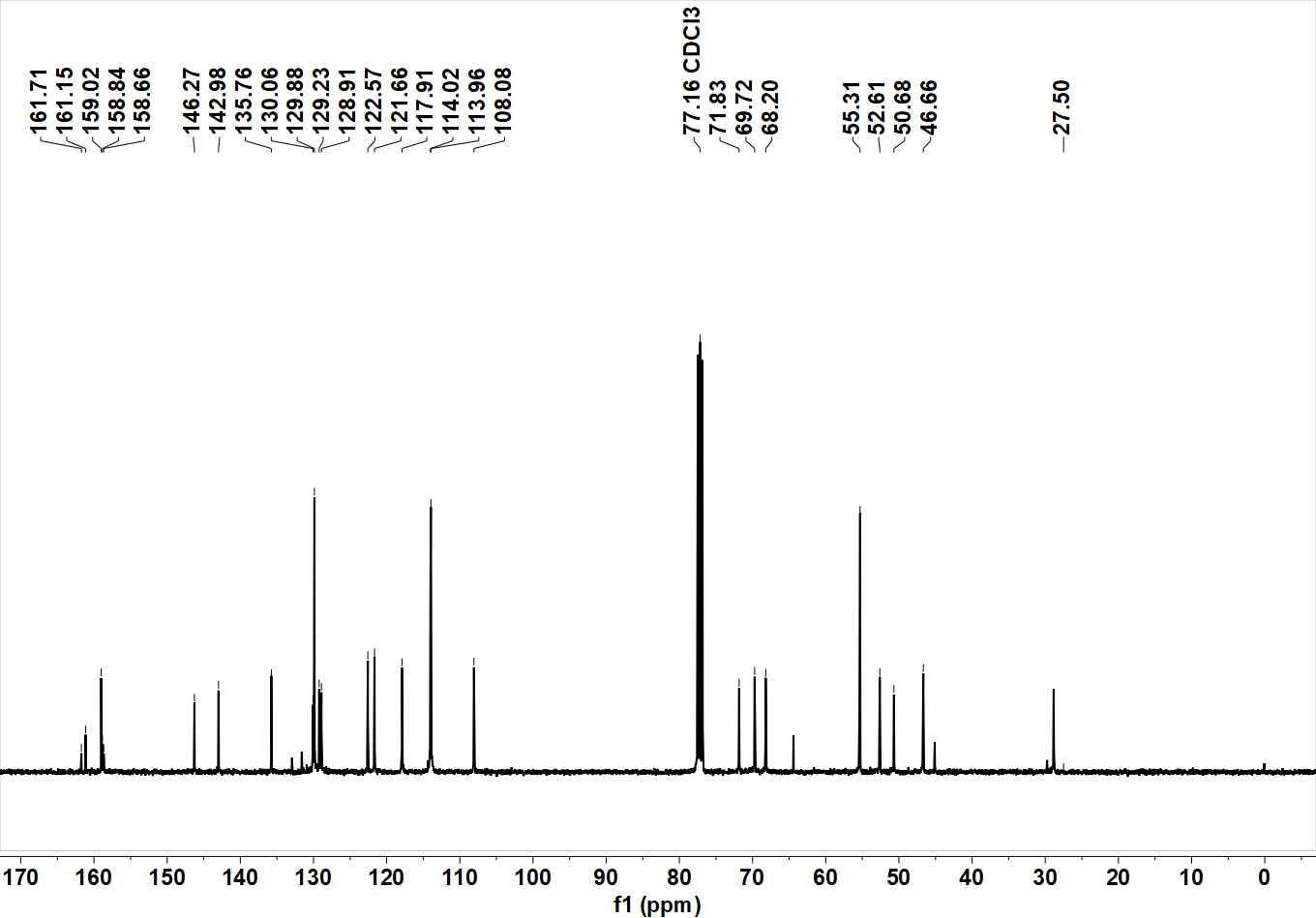
**

**Figure S48.** ^13^C NMR spectrum (101 MHz, Chloroform-*d*) of BiTA7

**Figure S49.** HRMS spectrum of BiTA7

**
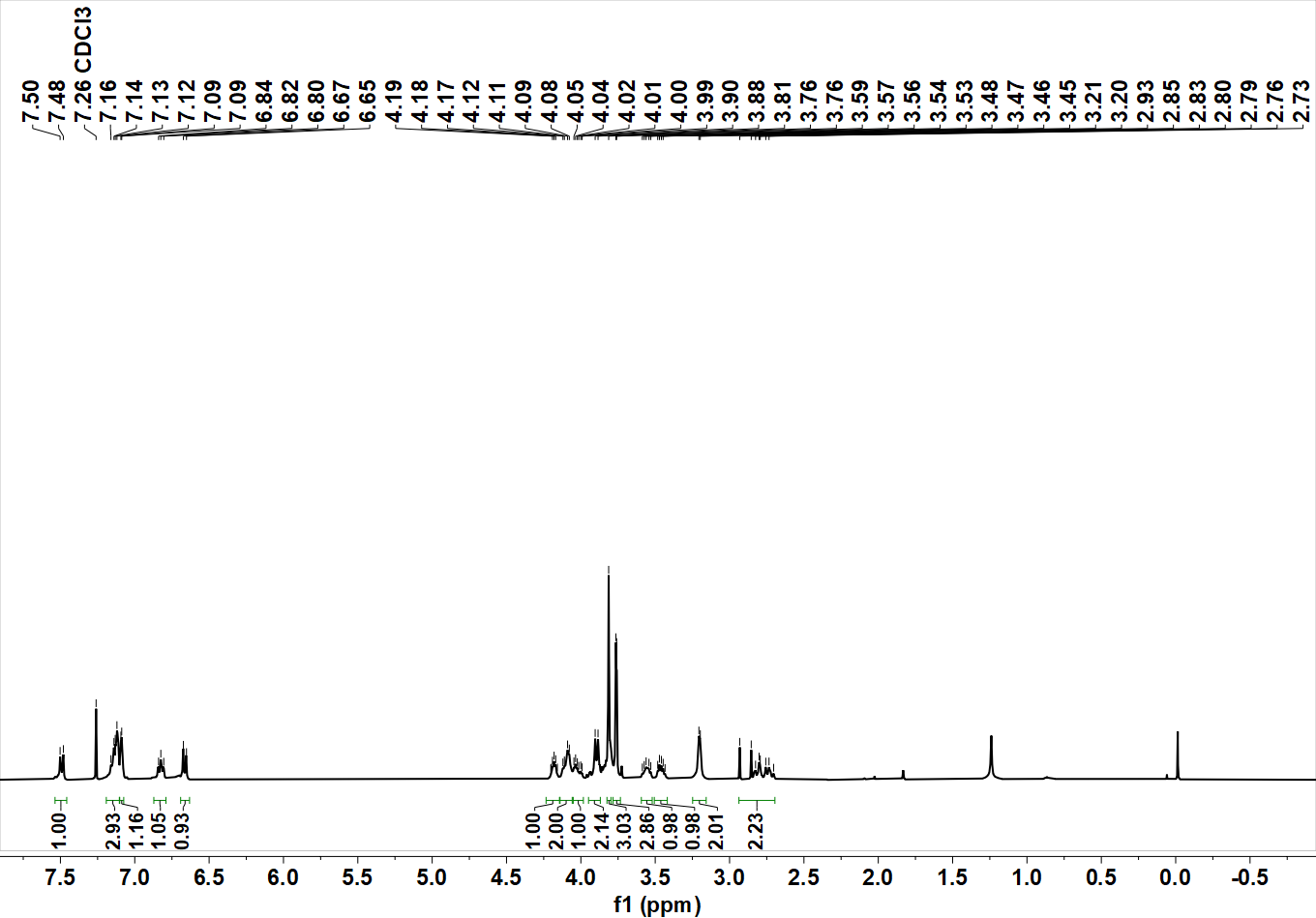
**

**Figure S50.** ^1^H NMR spectrum (400 MHz, Chloroform-*d*) of BiTA8

**
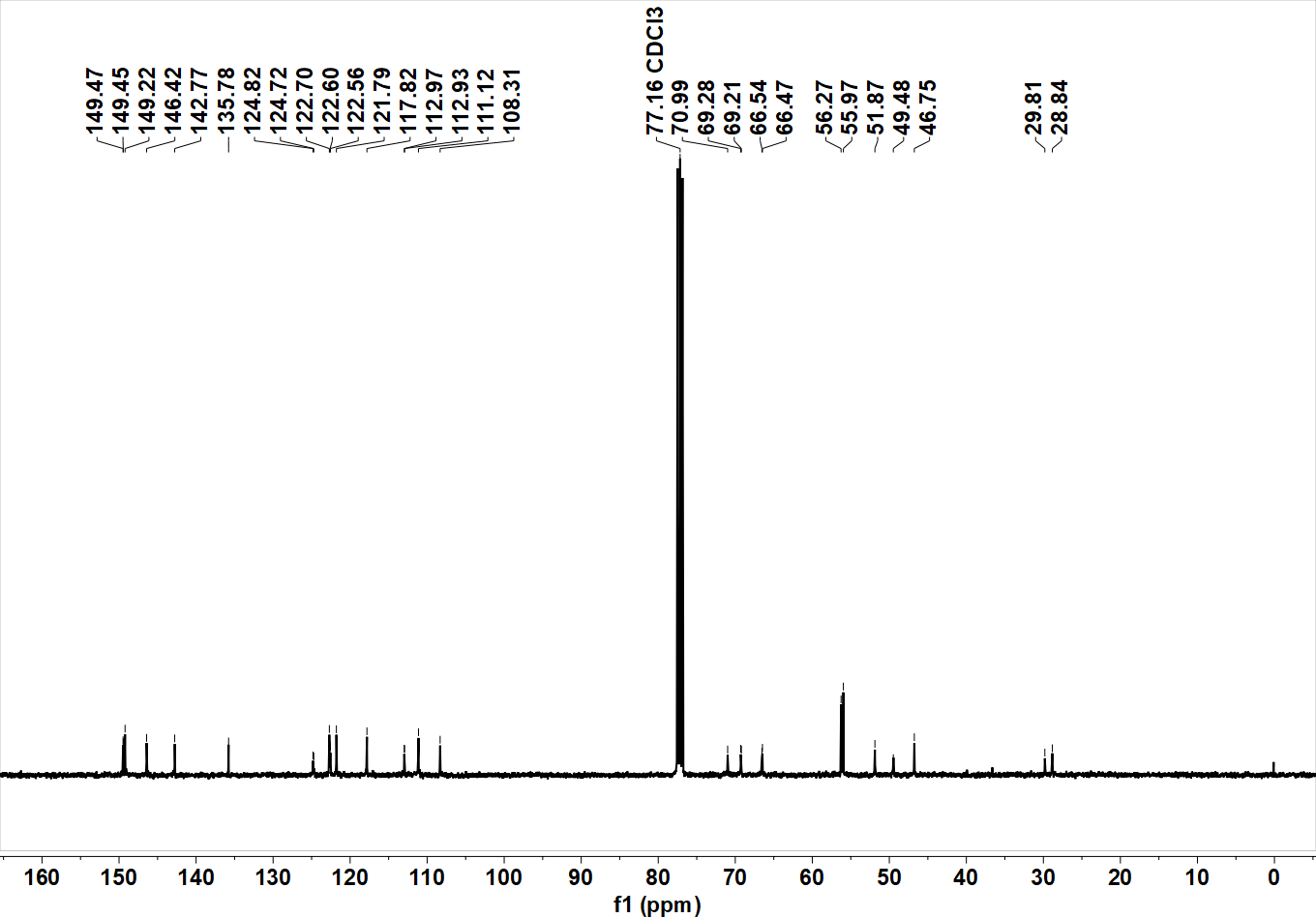
**

**Figure S51.** ^13^C NMR spectrum (101 MHz, Chloroform-*d*) of BiTA8

**Figure S52.** HRMS spectrum of BiTA8

**
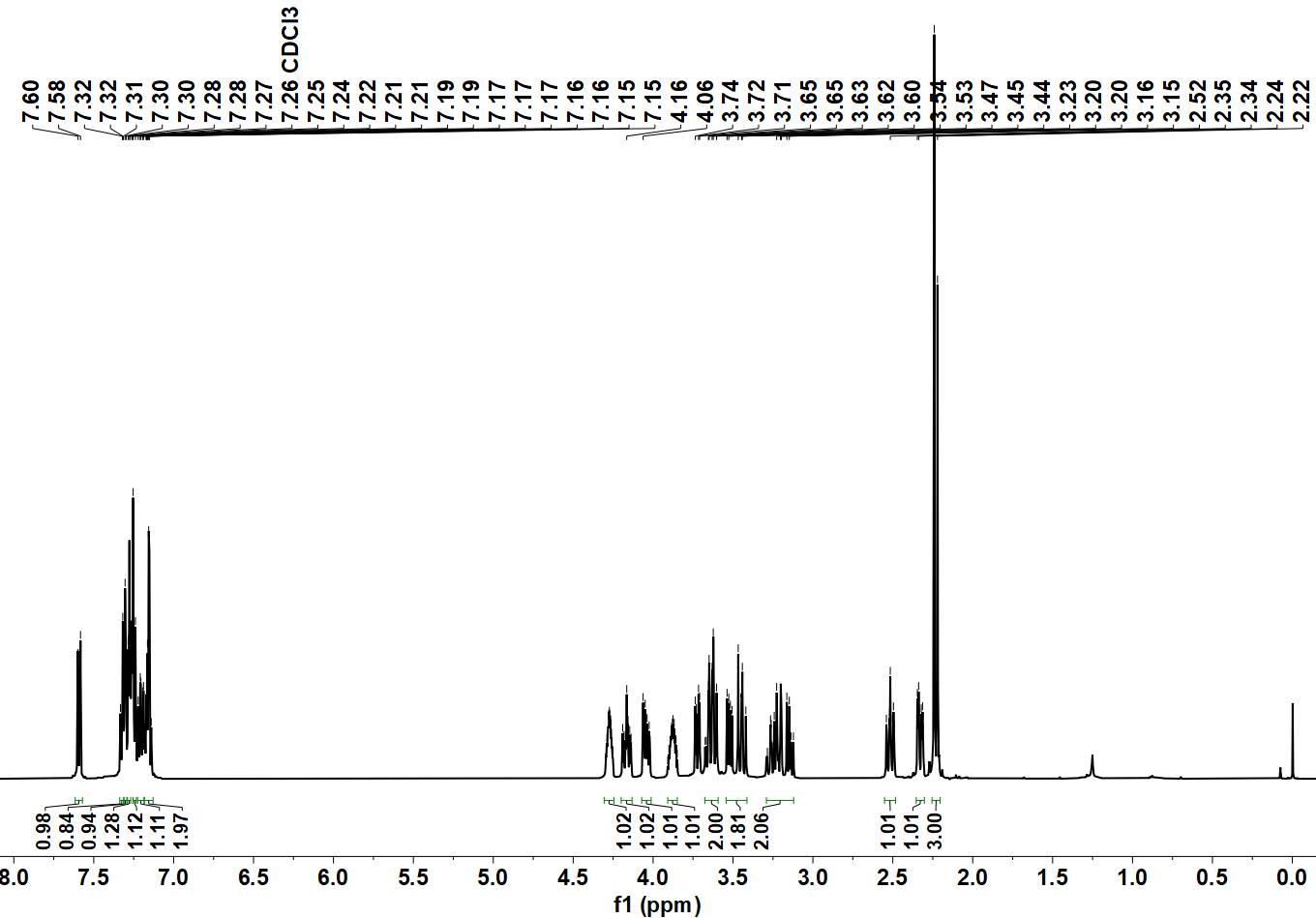
**

**Figure S53.** ^1^H NMR spectrum (500 MHz, Chloroform-*d*) of BiTA9

**
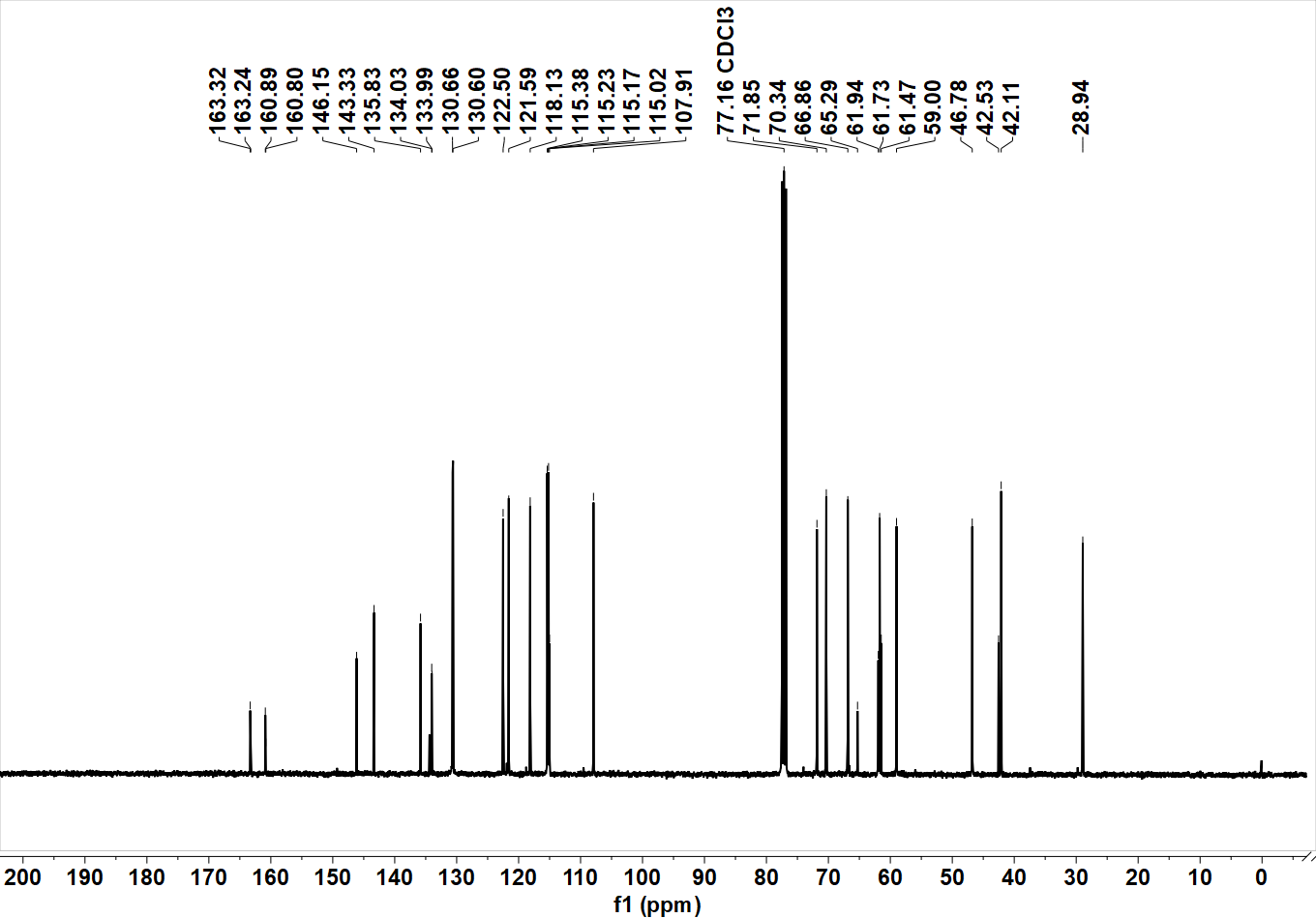
**

**Figure S54.** ^13^C NMR spectrum (126 MHz, Chloroform-*d*) of BiTA9

**
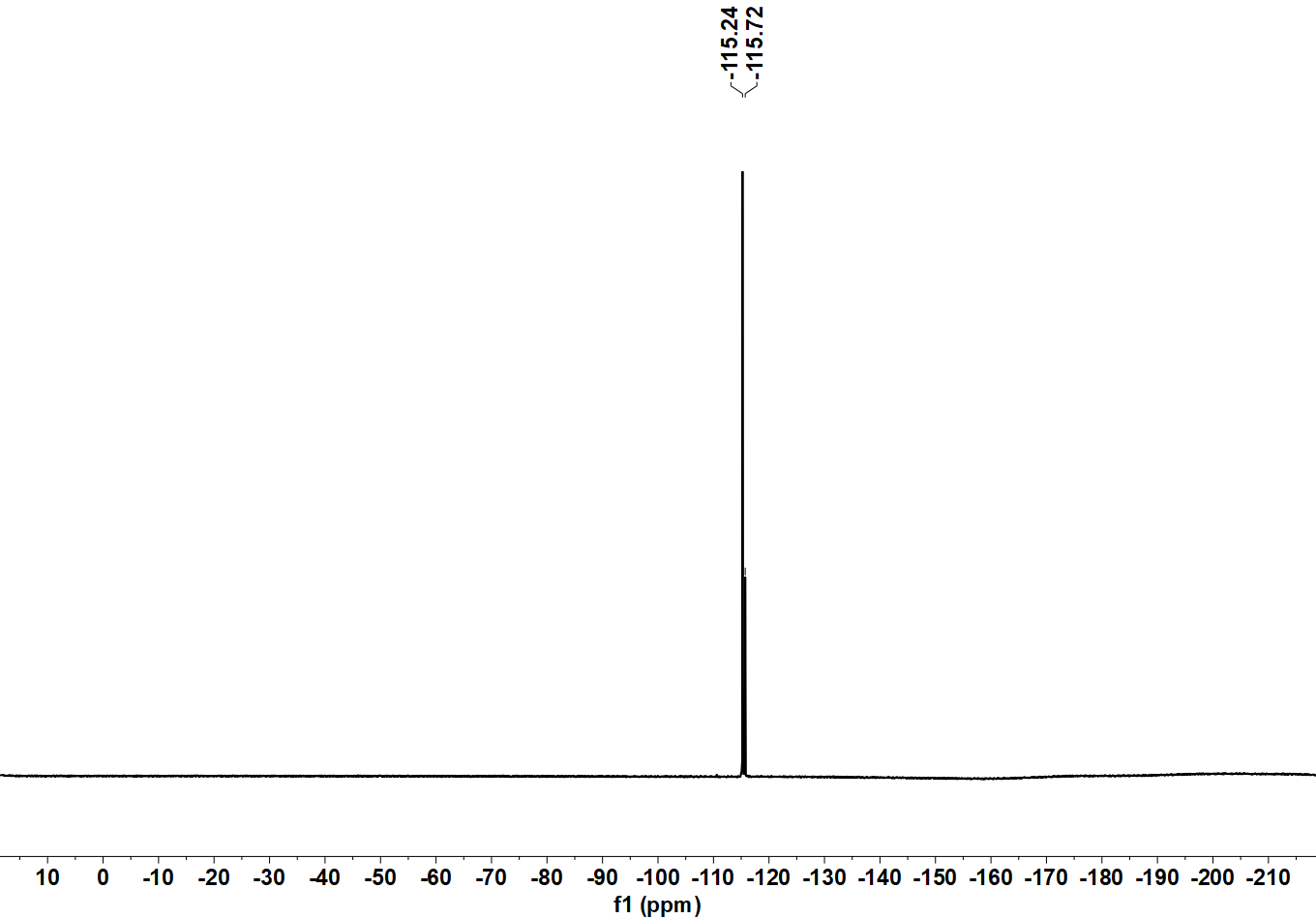
**

**Figure S55.** ^19^F NMR spectrum (471 MHz, Chloroform-*d*) of BiTA9

**Figure S56.** HRMS spectrum of BiTA9

**
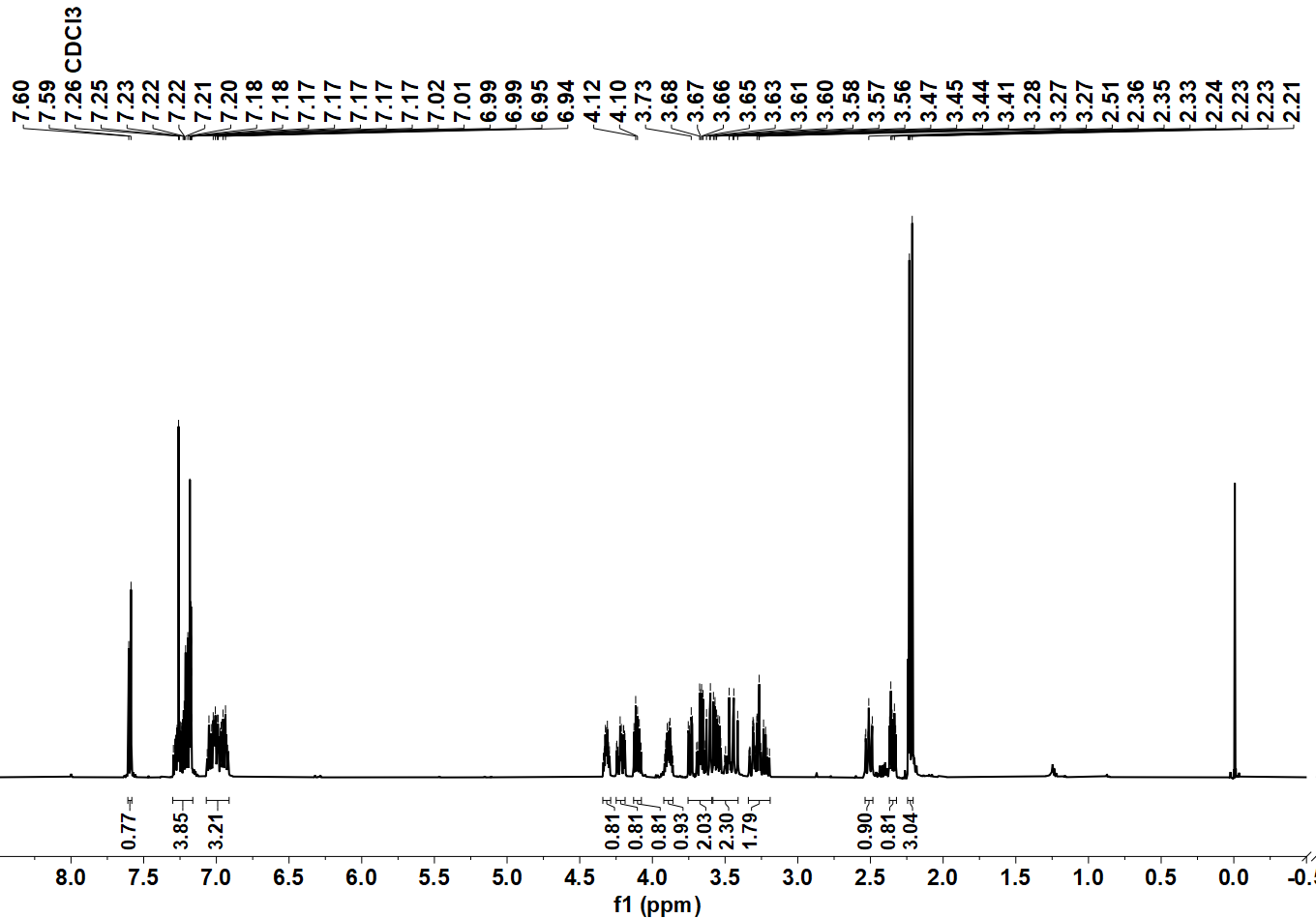
**

**Figure S57.** ^1^H NMR spectrum (500 MHz, Chloroform-*d*) of BiTA10

**
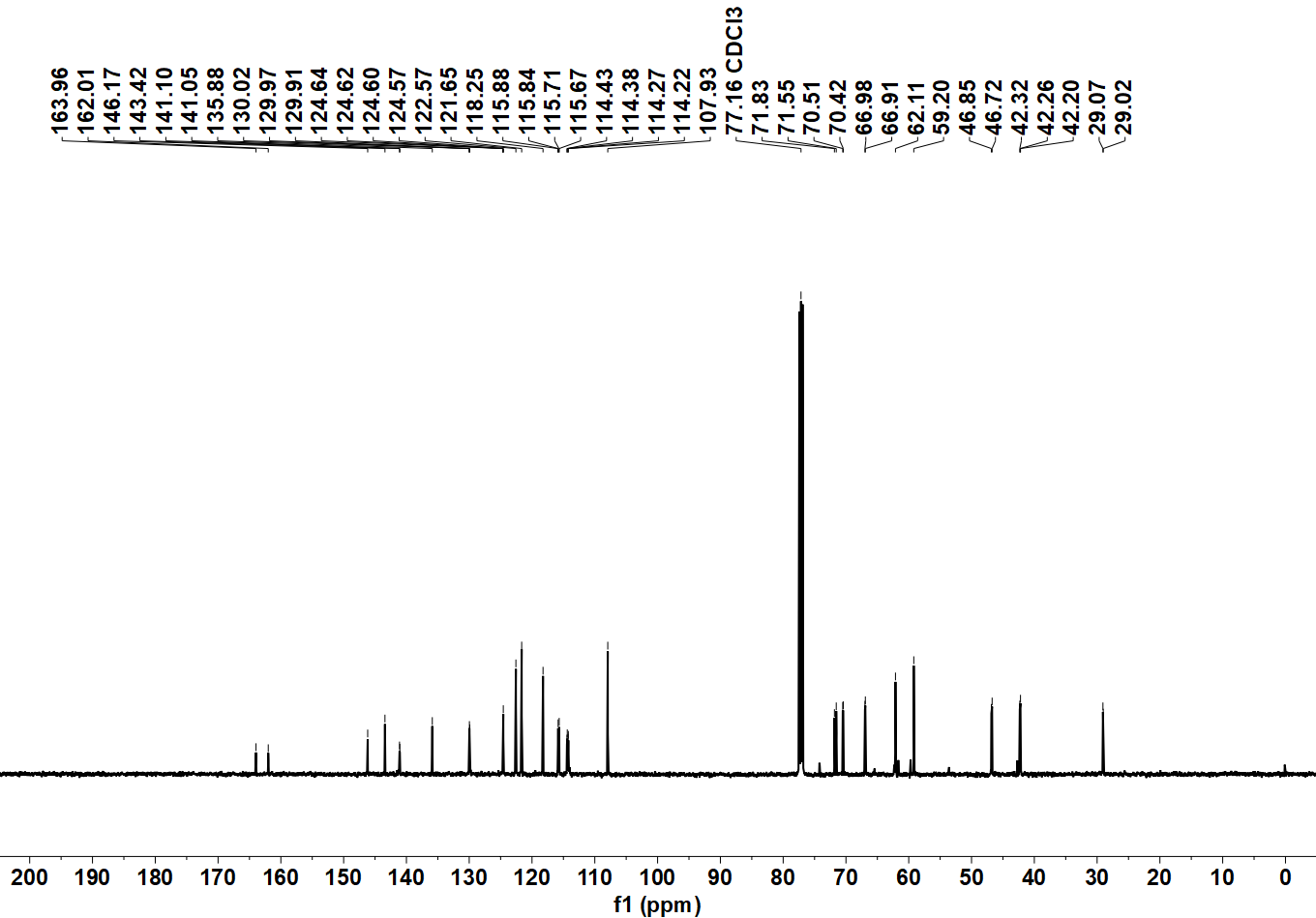
**

**Figure S58.** ^13^C NMR spectrum (126 MHz, Chloroform-*d*) of BiTA10

**
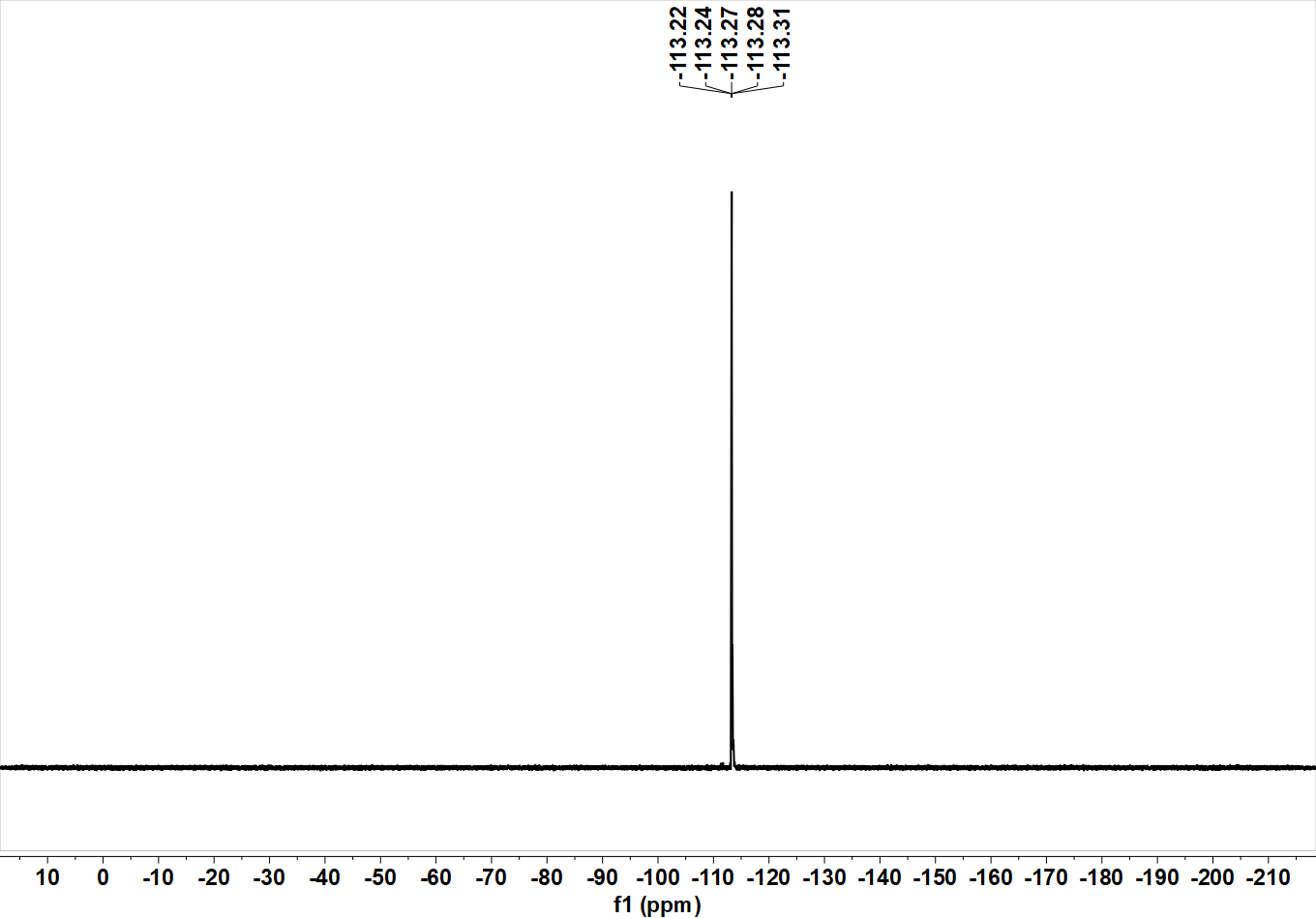
**

**Figure S59.** ^19^F NMR spectrum (471 MHz, Chloroform-*d*) of BiTA10

**Figure S60.** HRMS spectrum of BiTA10

**
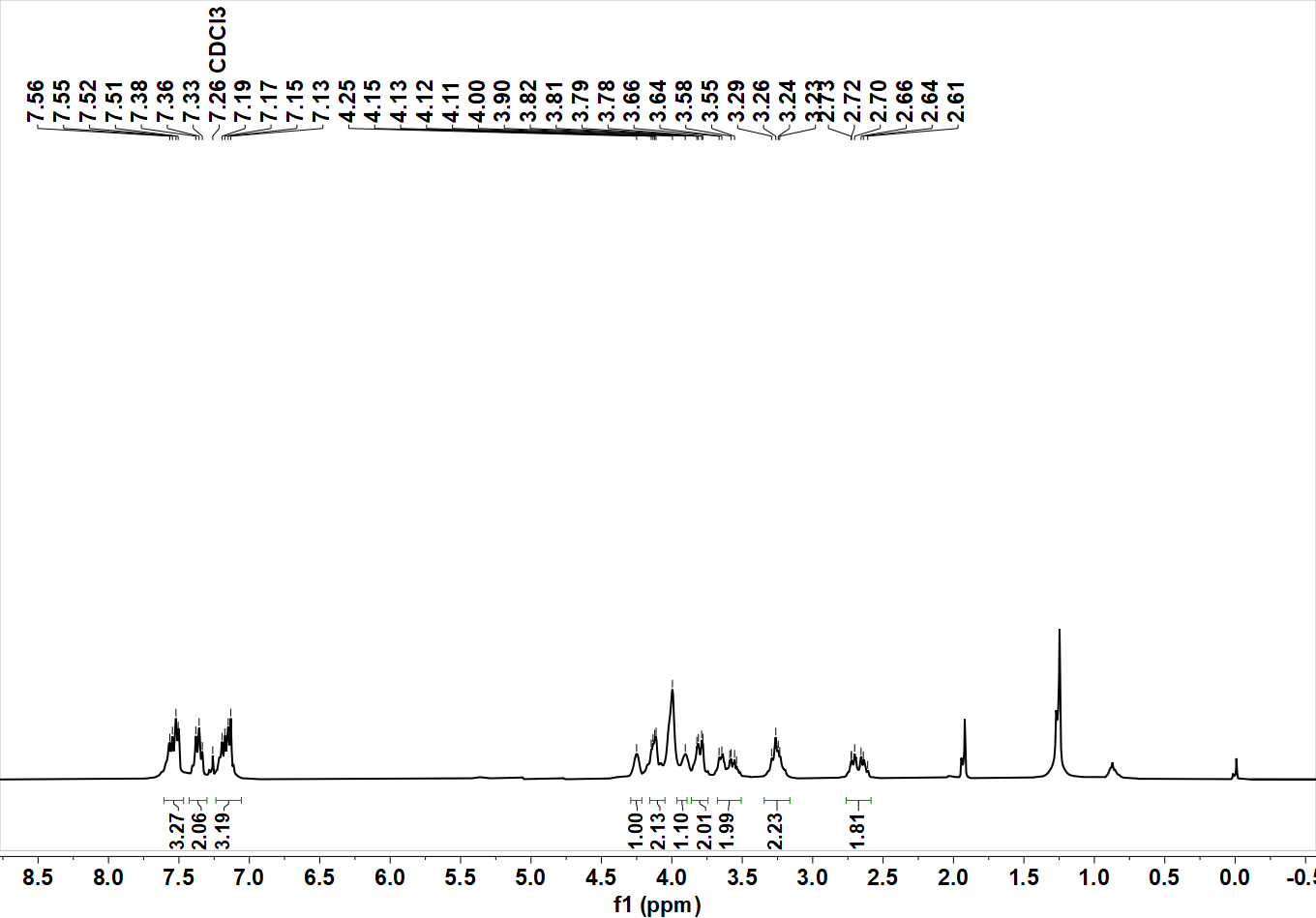
**

**Figure S61.** ^1^H NMR spectrum (400 MHz, Chloroform-*d*) of BiTA11

**
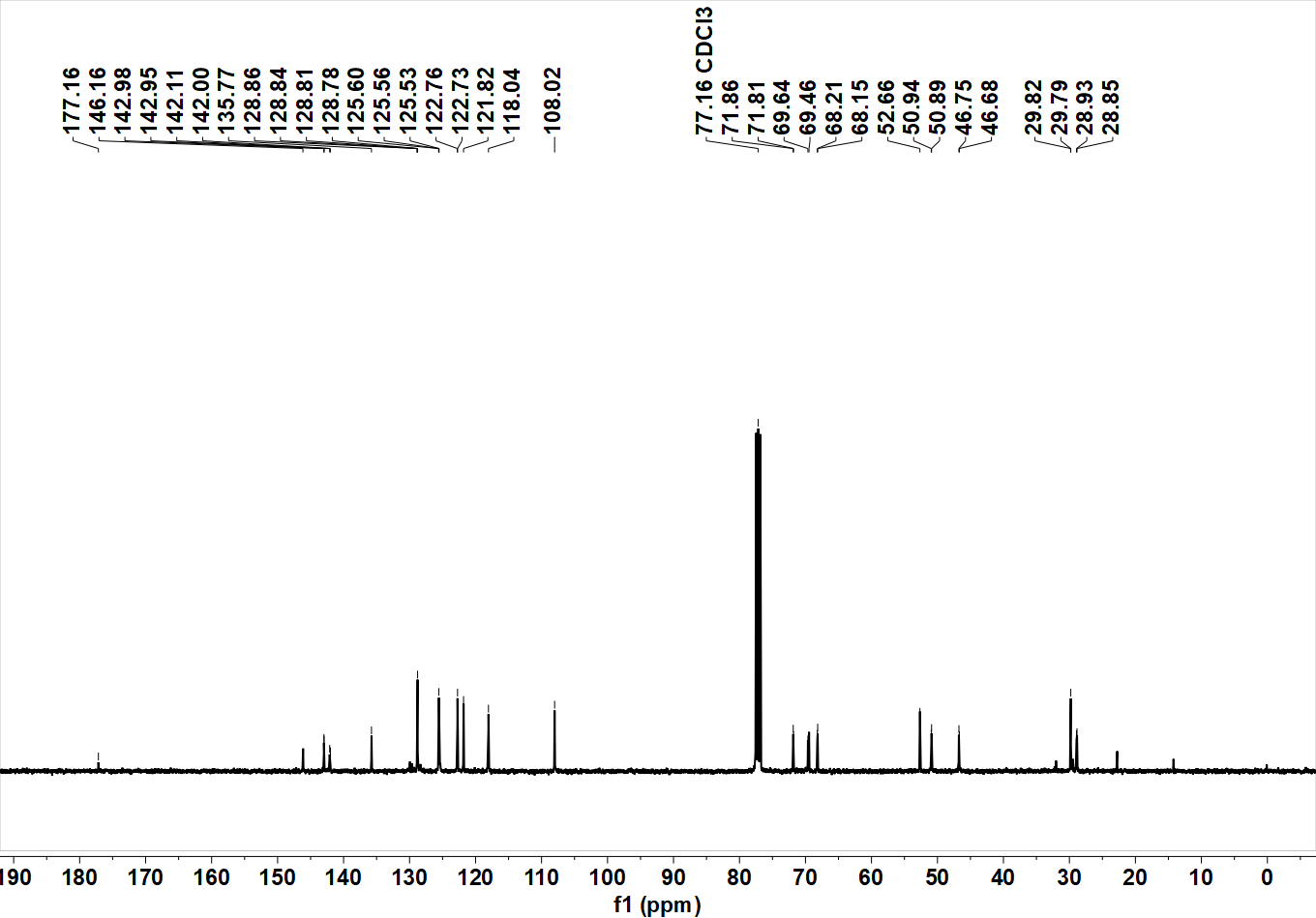
**

**Figure S62.** ^13^C NMR spectrum (101 MHz, Chloroform-*d*) of BiTA11

**
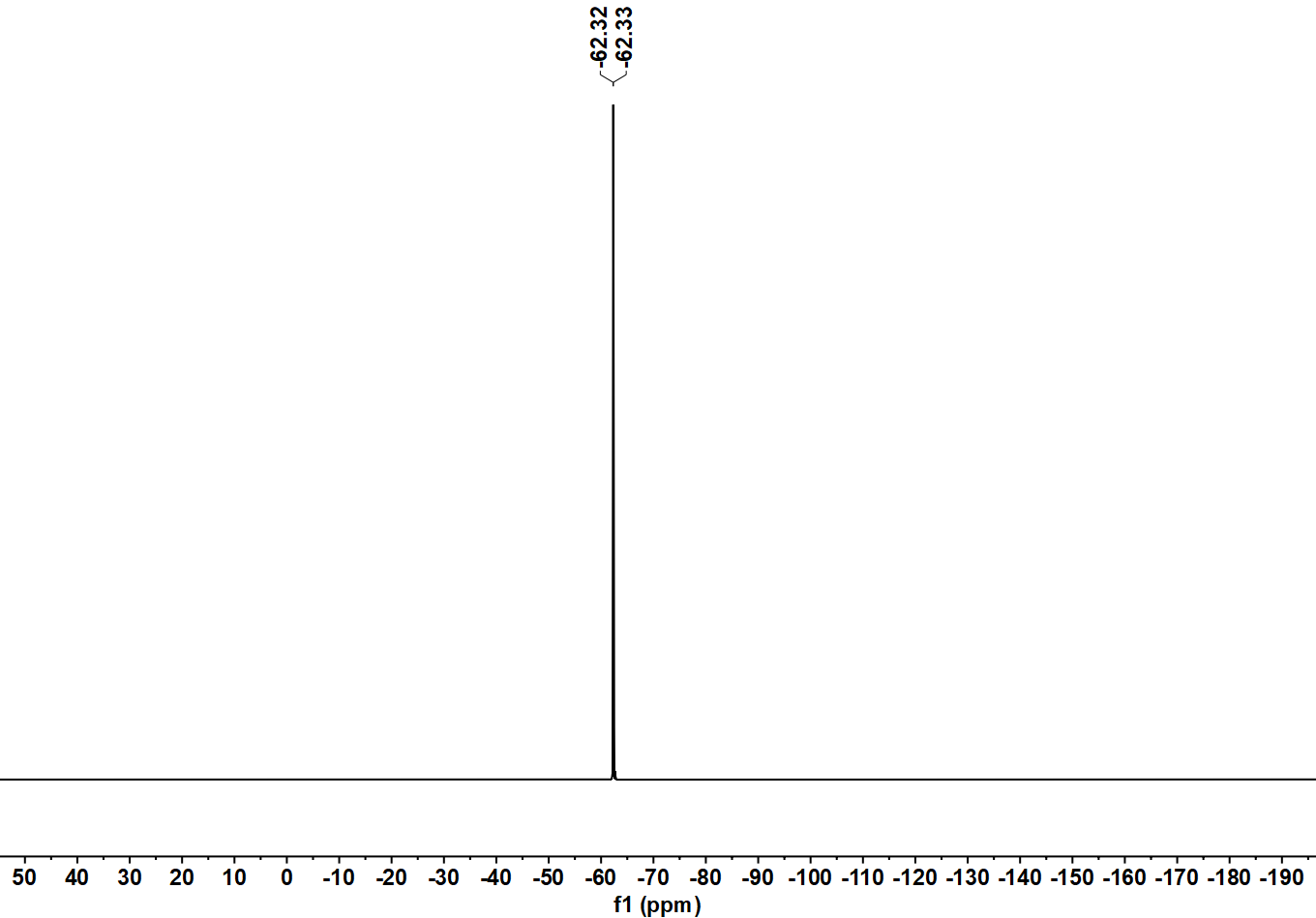
**

**Figure S63.** ^19^F NMR spectrum (376 MHz, Chloroform-*d*) of BiTA11

**Figure S64.** HRMS spectrum of BiTA11

**
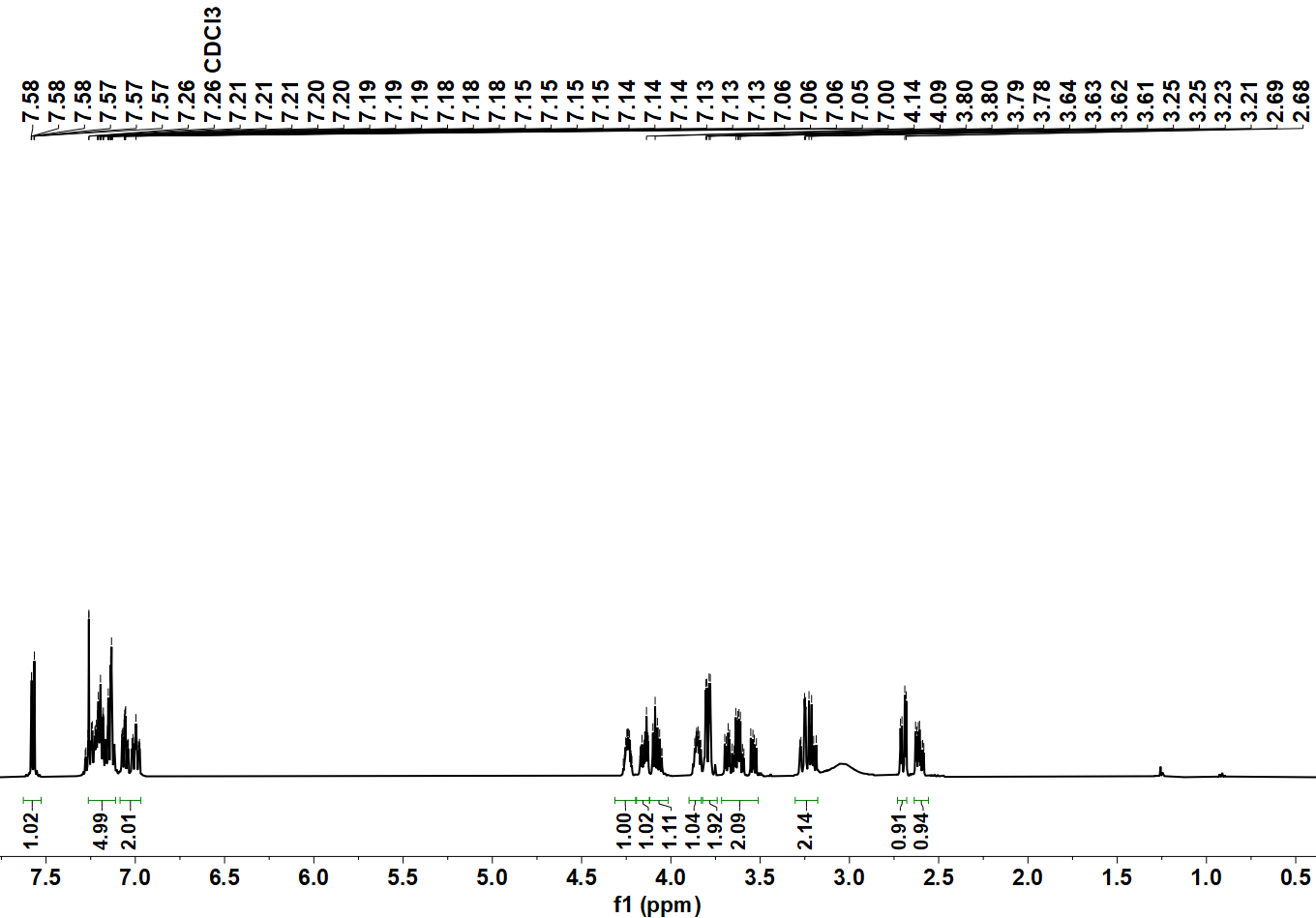
**

**Figure S65.** ^1^H NMR spectrum (500 MHz, Chloroform-*d*) of BiTA12

**
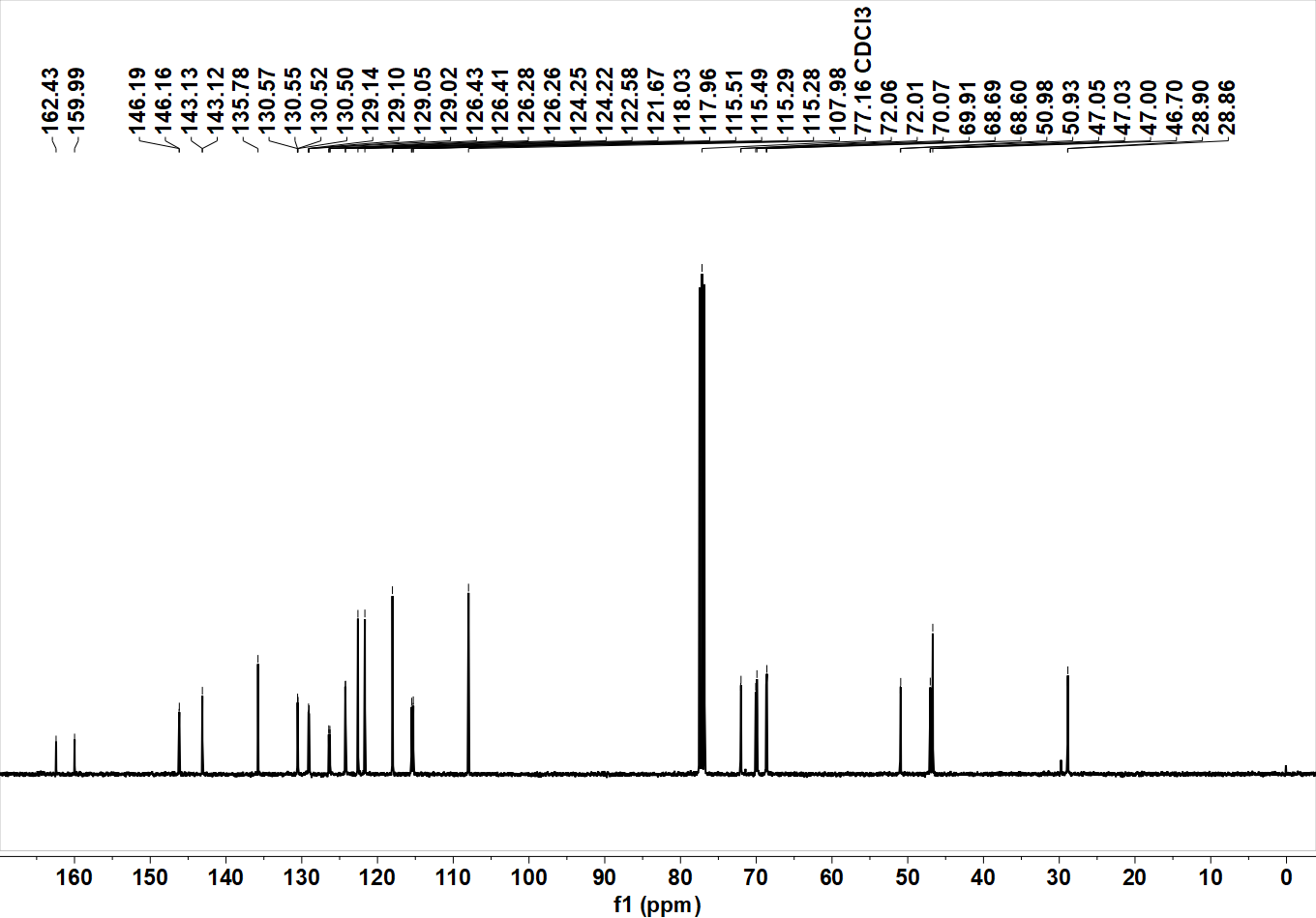
**

**Figure S66.** ^13^C NMR spectrum (126 MHz, Chloroform-*d*) of BiTA12

**
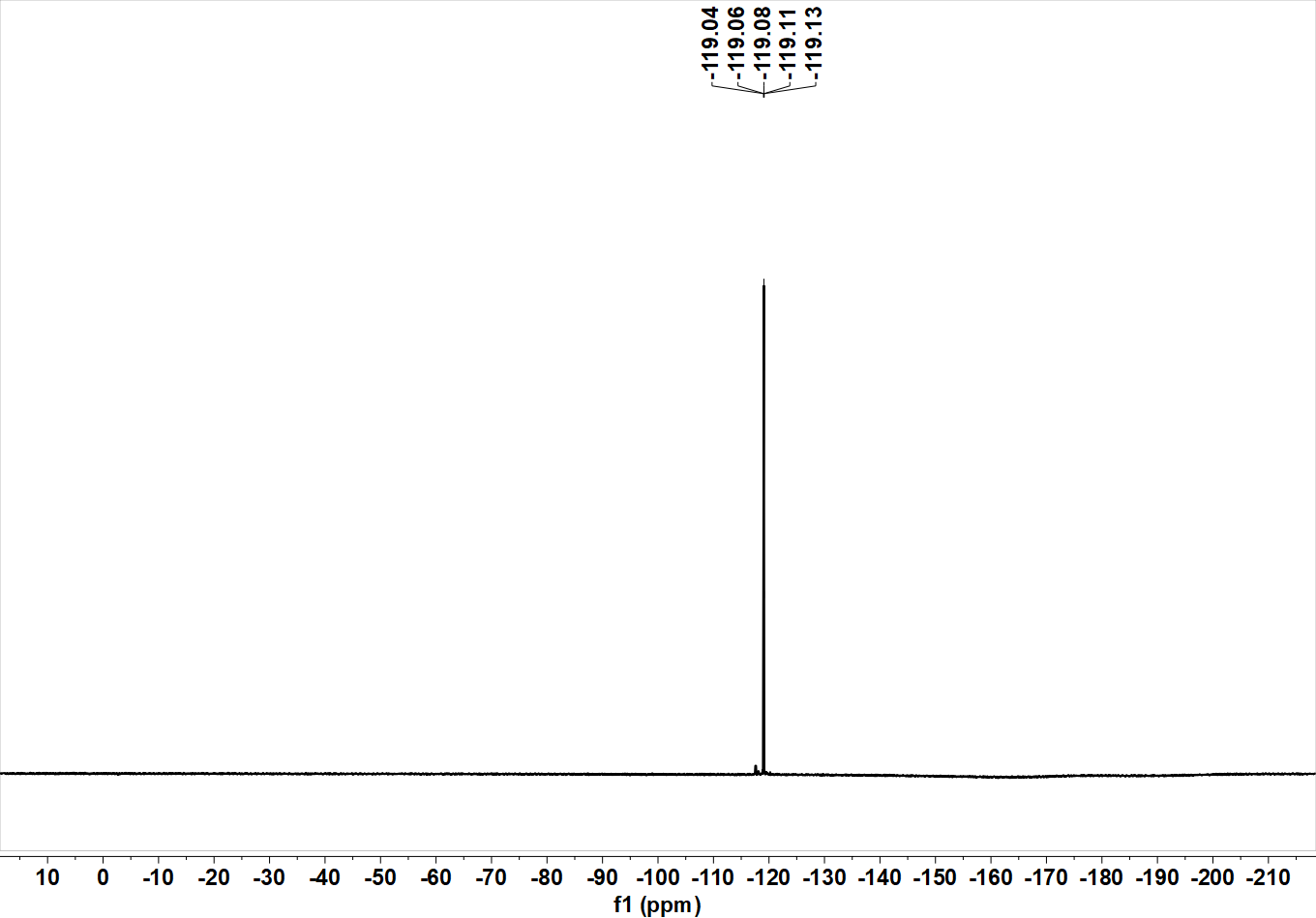
**

**Figure S67.** ^19^F NMR spectrum (471 MHz, Chloroform-*d*) of BiTA12

**Figure S68.** HRMS spectrum of BiTA12

**
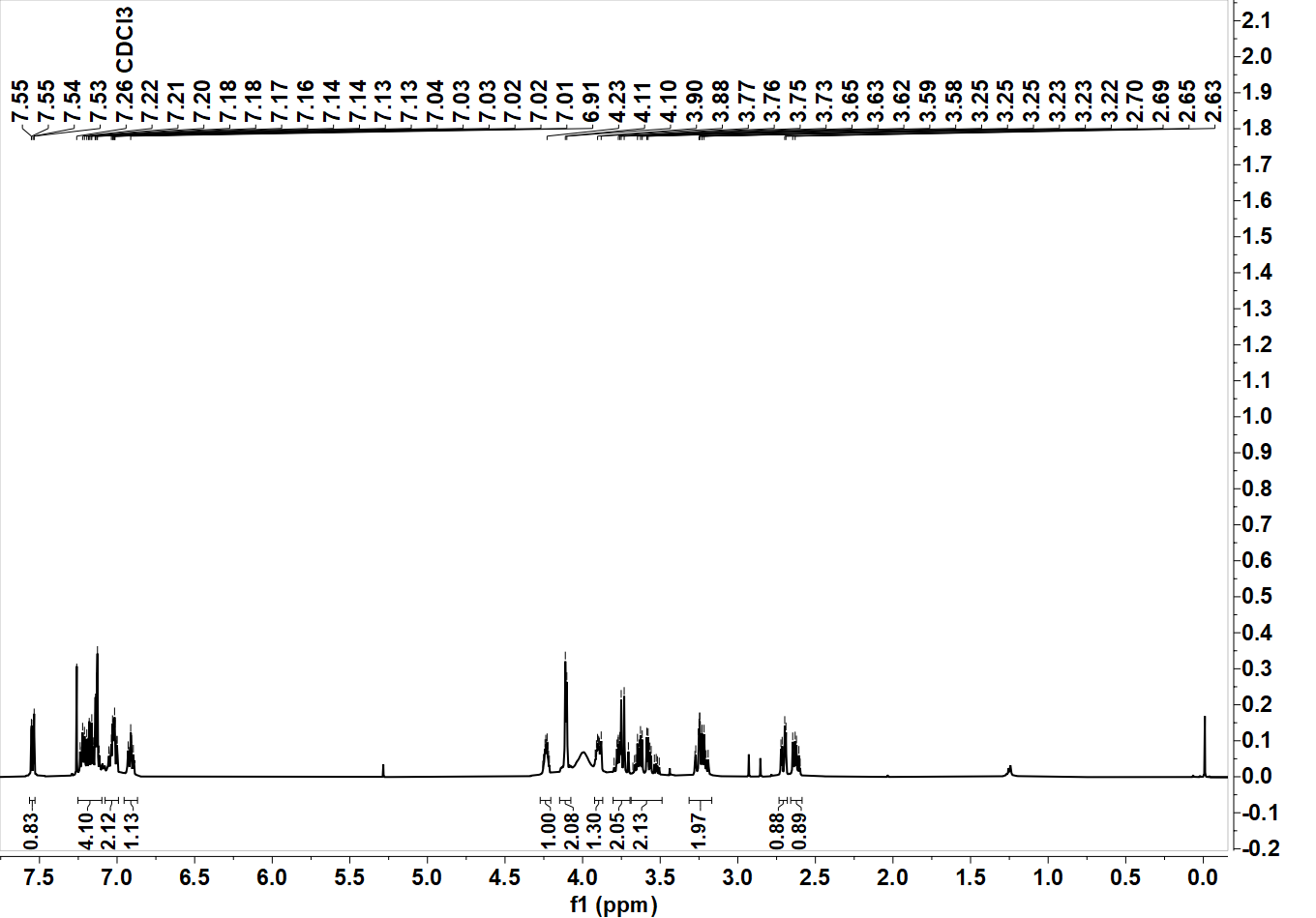
**

**Figure S69.** ^1^H NMR spectrum (500 MHz, Chloroform-*d*) of BiTA13

**
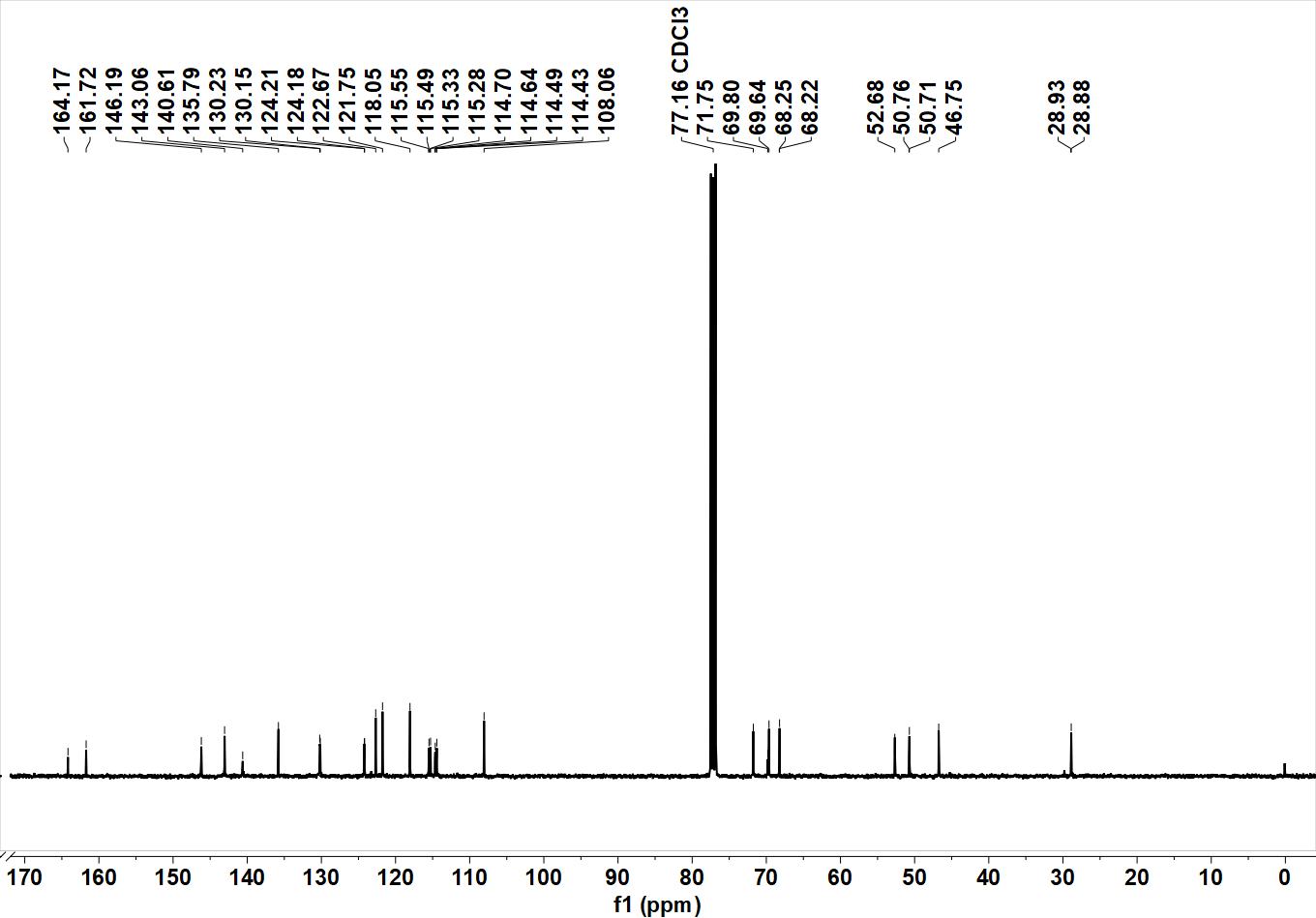
**

**Figure S70.** ^13^C NMR spectrum (126 MHz, Chloroform-*d*) of BiTA13

**
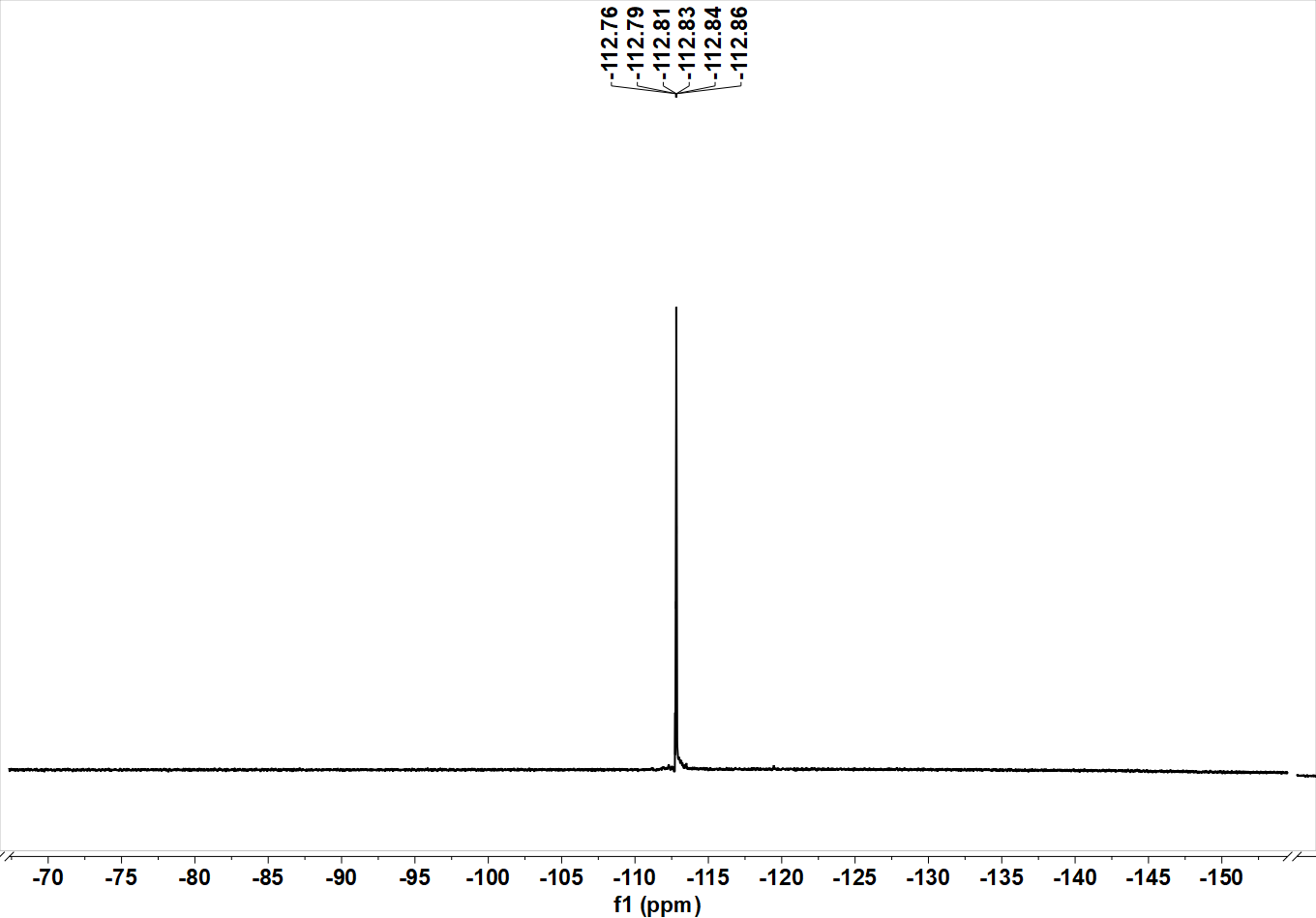
**

**Figure S71.** ^19^F NMR spectrum (471 MHz, Chloroform-*d*) of BiTA13

**Figure S72.** HRMS spectrum of BiTA13

**
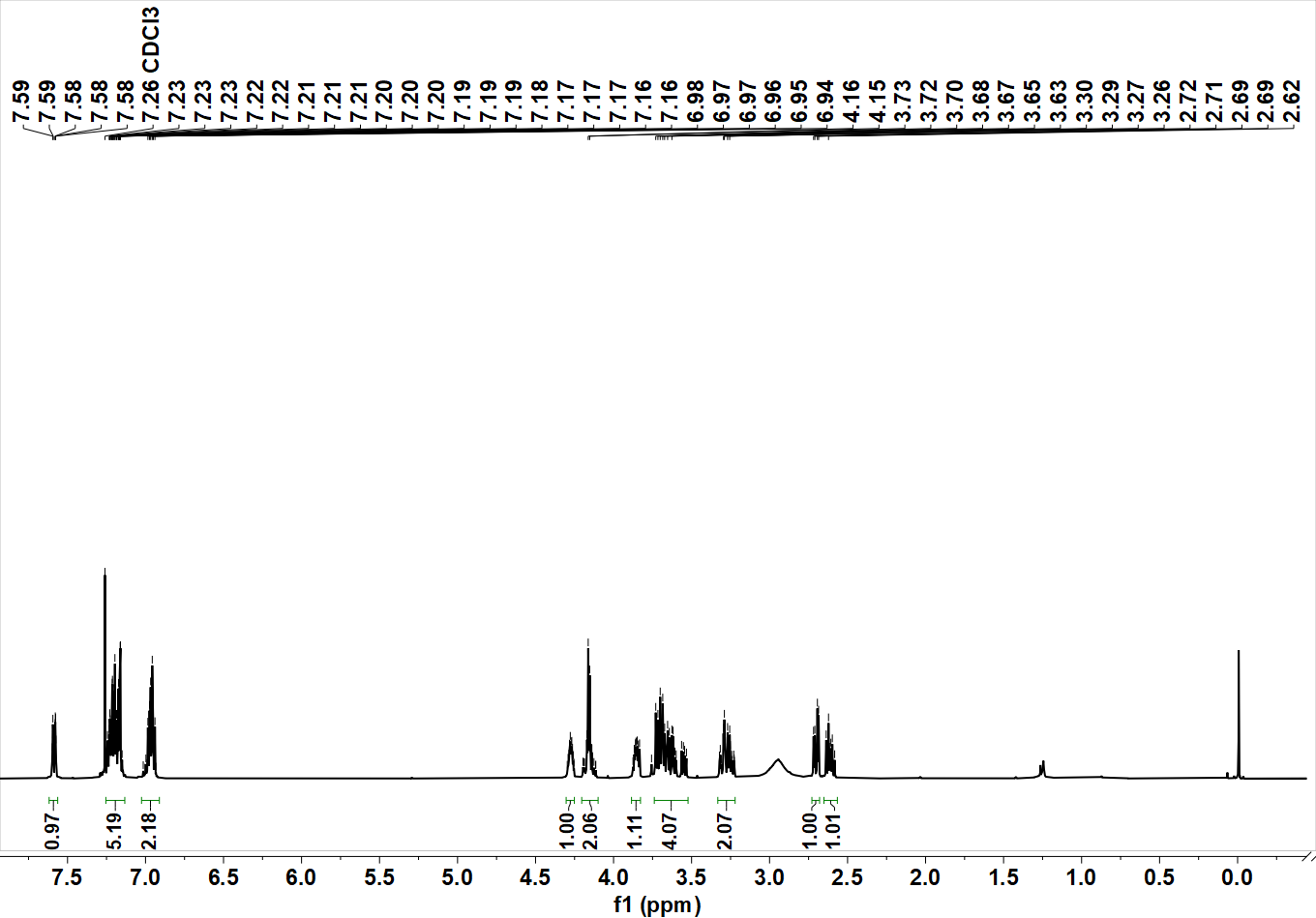
**

**Figure S73.** ^1^H NMR spectrum (500 MHz, Chloroform-*d*) of BiTA14

**
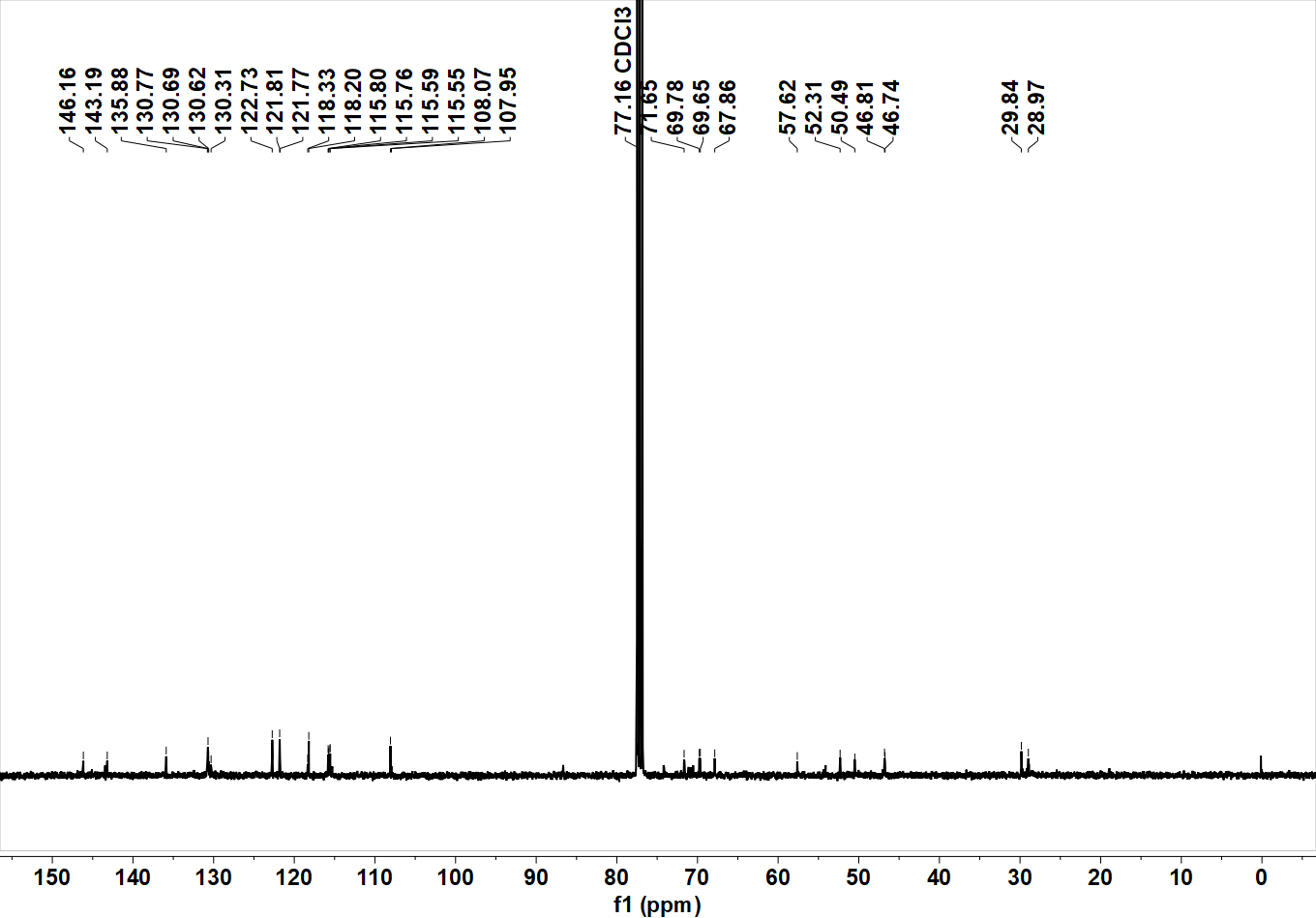
**

**Figure S74.** ^13^C NMR spectrum (126 MHz, Chloroform-*d*) of BiTA14

**
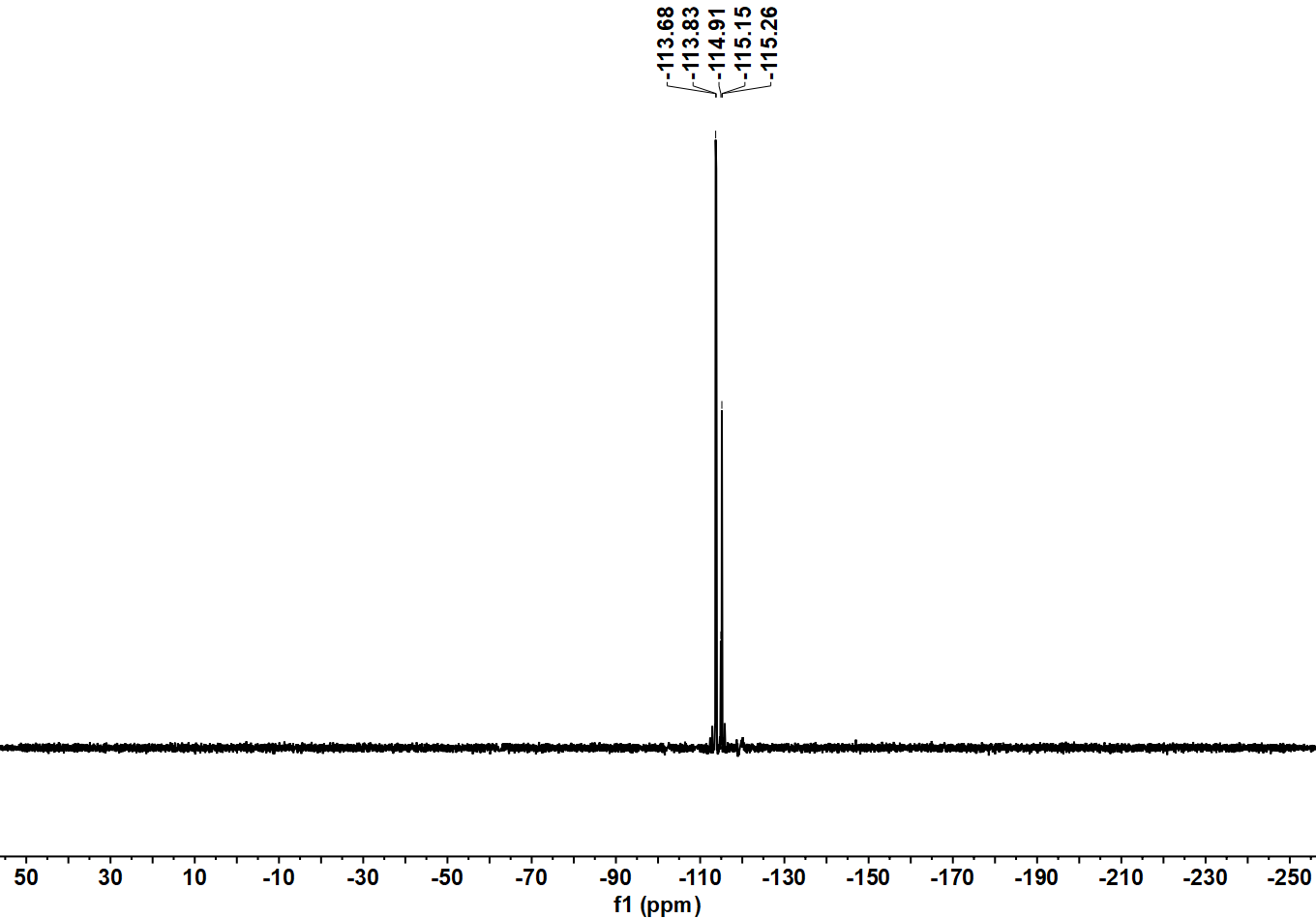
**

**Figure S75.** ^19^F NMR spectrum (471 MHz, Chloroform-*d*) of BiTA14

**Figure S76.** HRMS spectrum of BiTA14

**
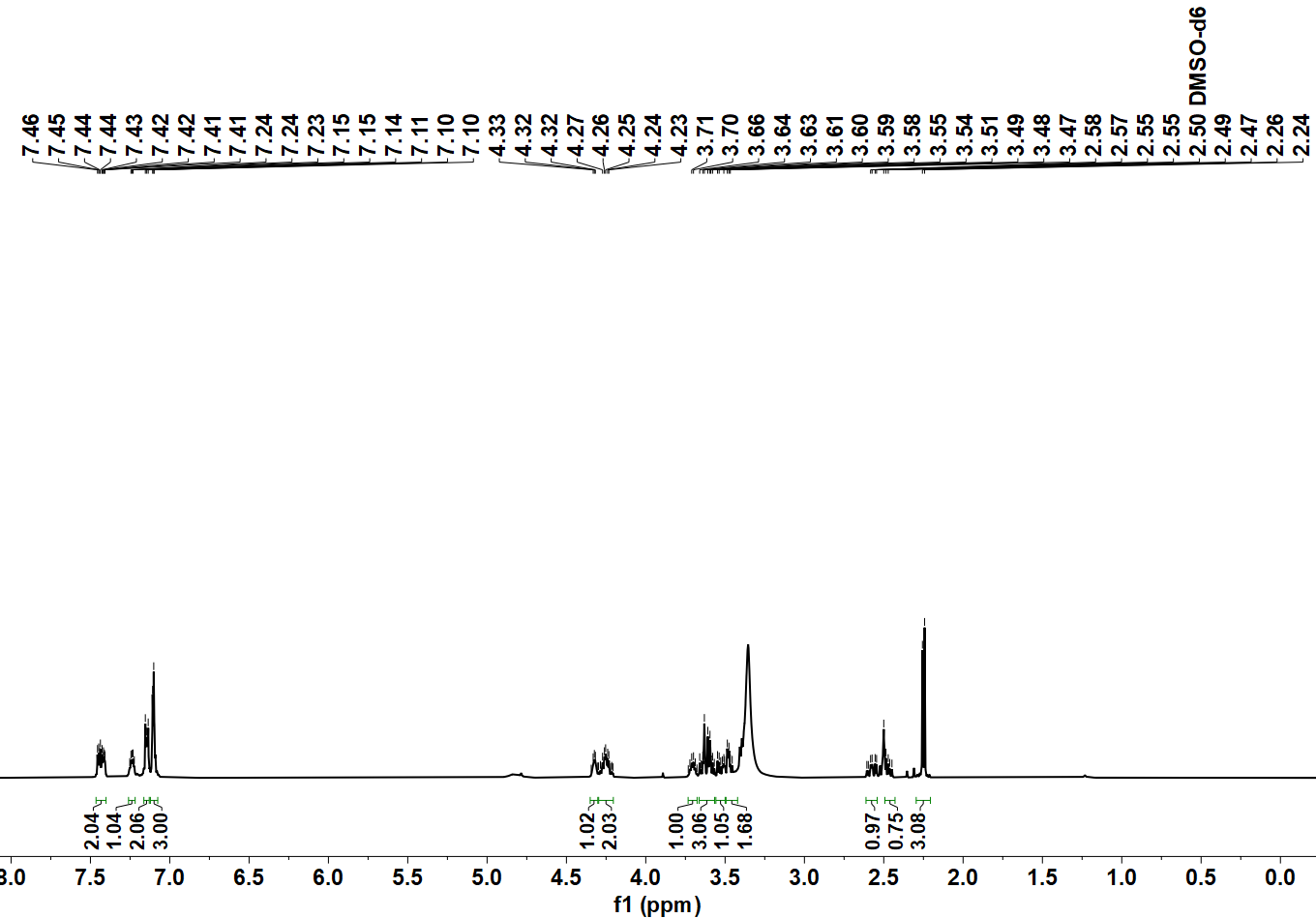
**

**Figure S77.** ^1^H NMR spectrum (500 MHz, DMSO-*d*_6_) of BiTA15

**
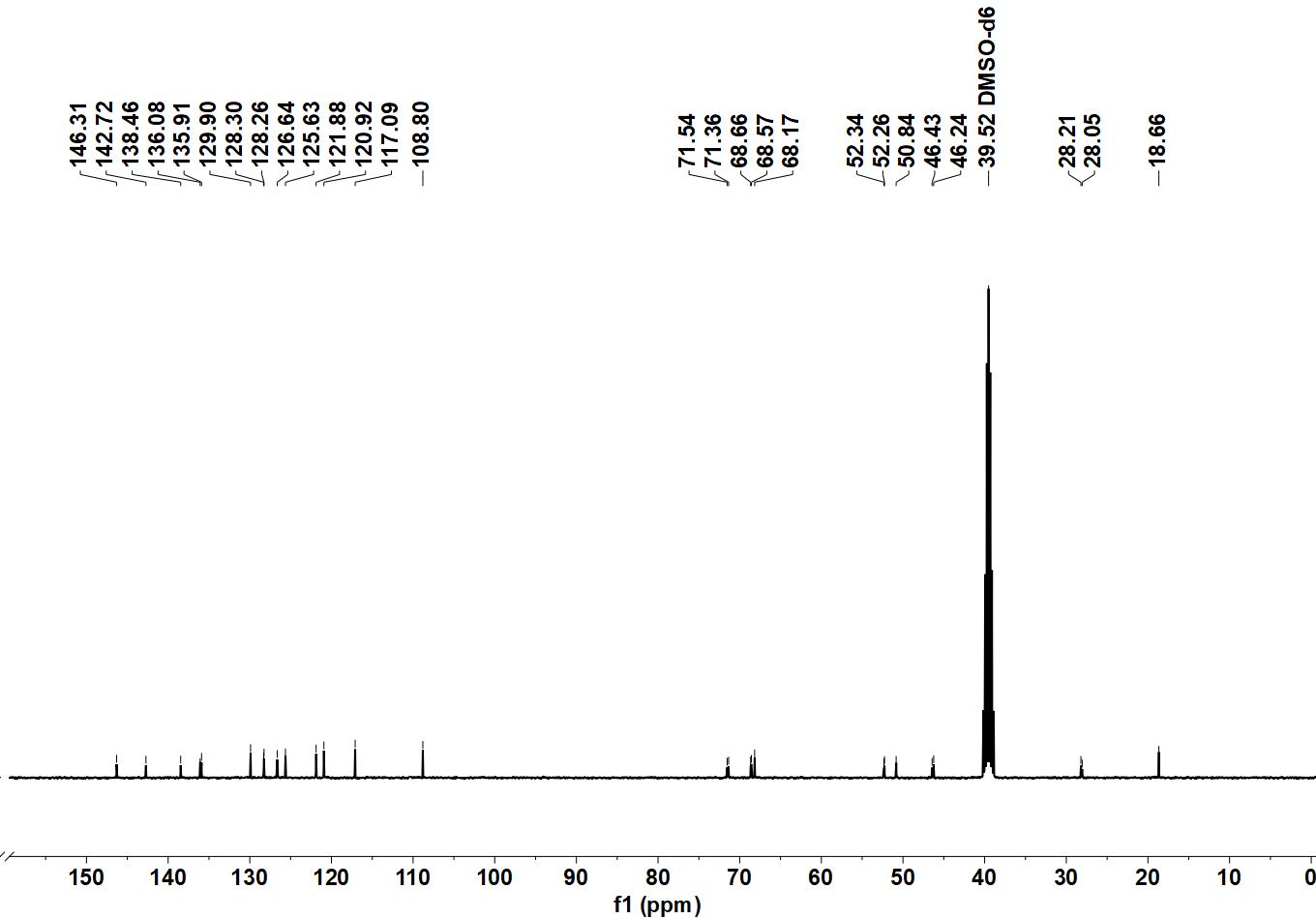
**

**Figure S78.** ^13^C NMR spectrum (126 MHz, DMSO-*d*_6_) of BiTA15

**Figure S79.** HRMS spectrum of BiTA15

**
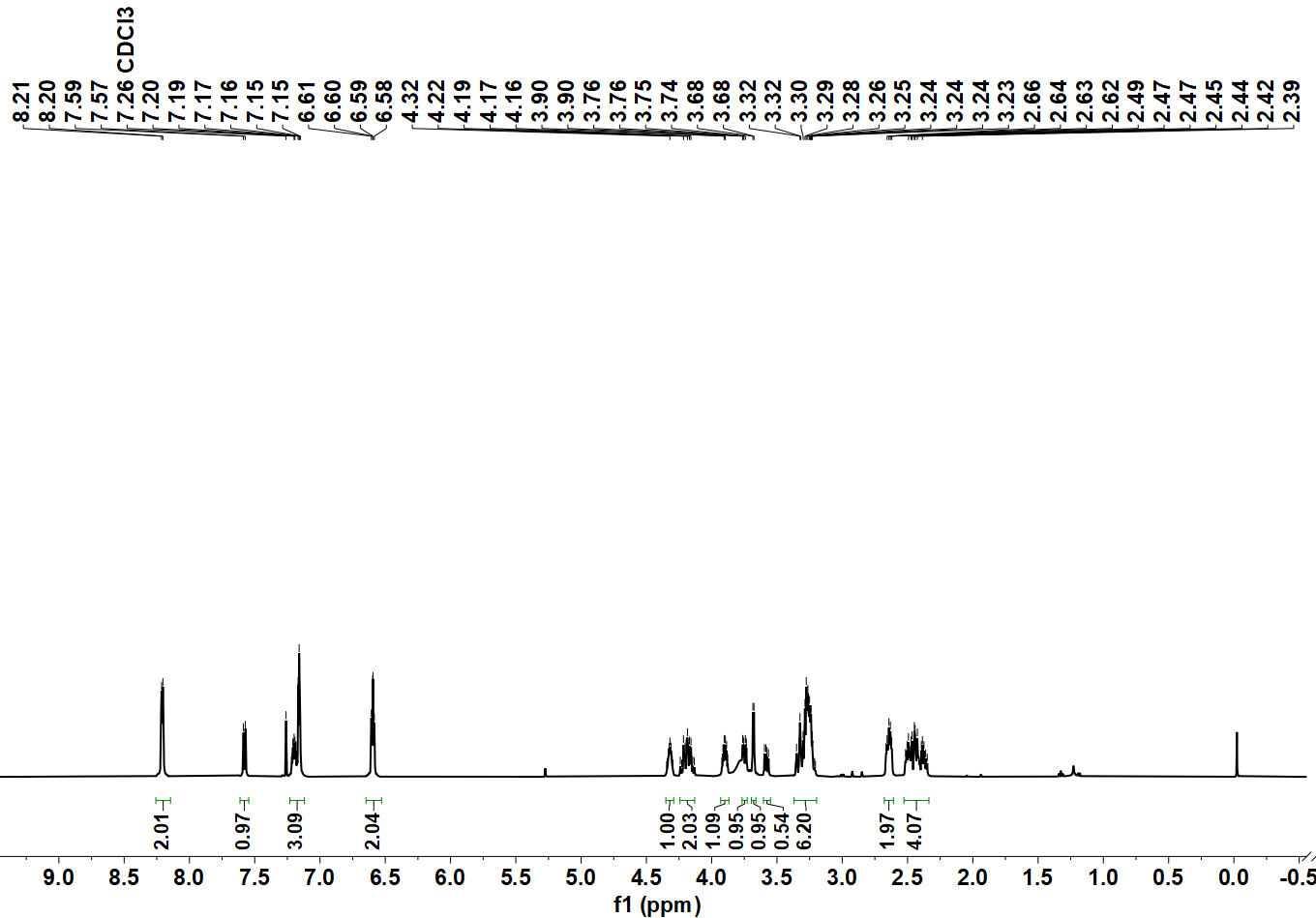
**

**Figure S80.** ^1^H NMR spectrum (500 MHz, Chloroform-*d*) of BiTA16

**
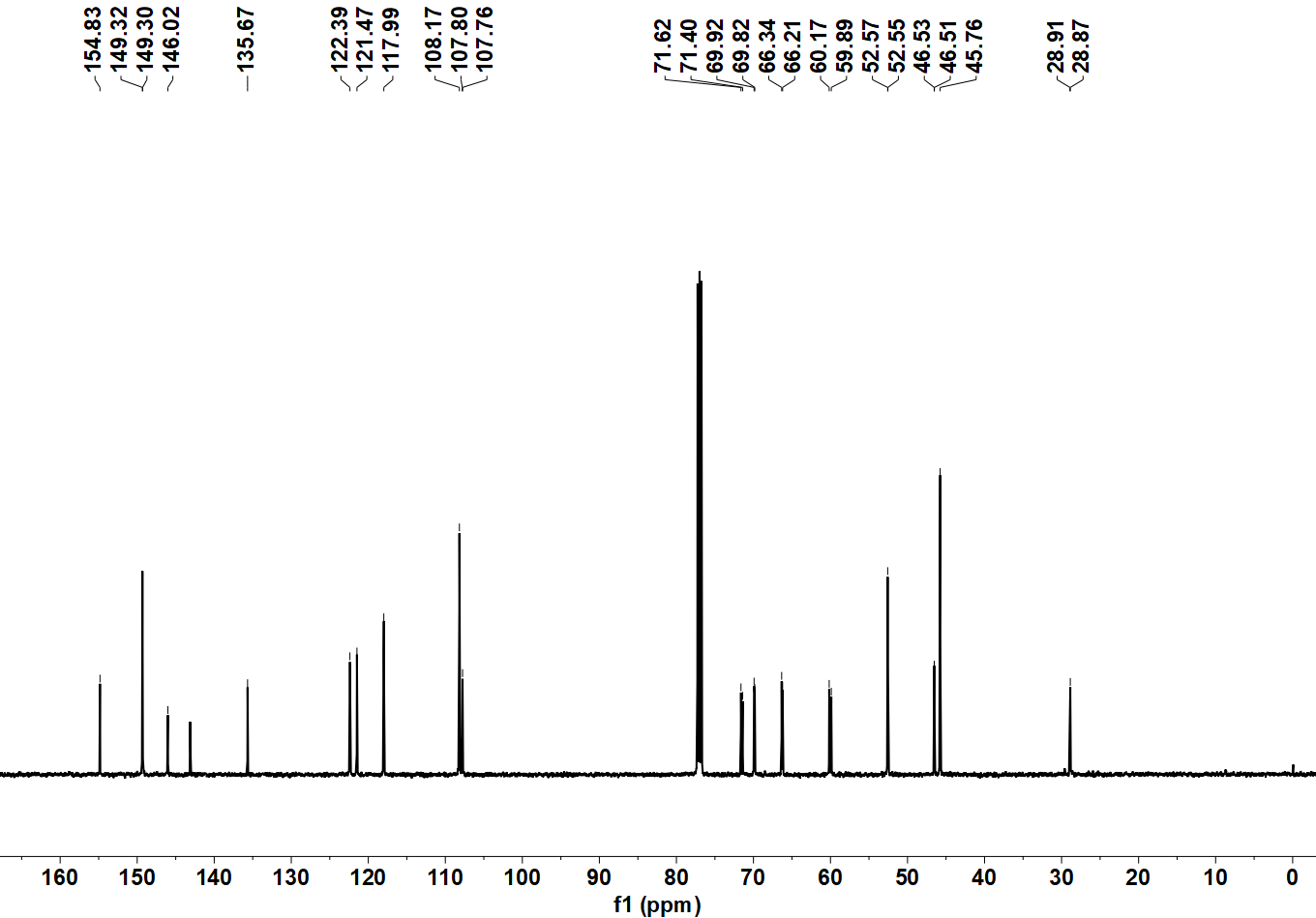
**

**Figure S81.** ^13^C NMR spectrum (126 MHz, Chloroform-*d*) of BiTA16

**Figure S82.** HRMS spectrum of BiTA16

**
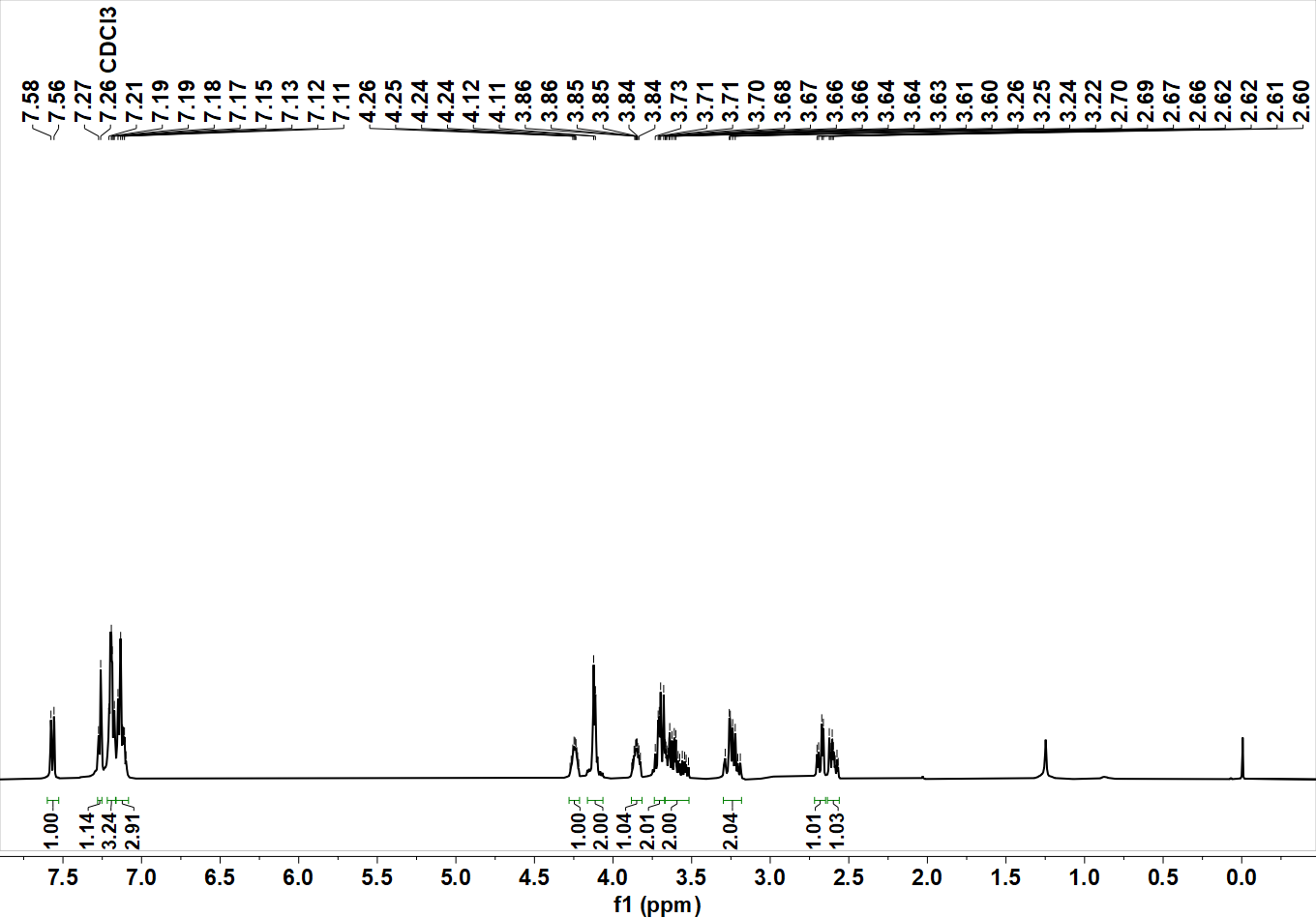
**

**Figure S83.** ^1^H NMR spectrum (400 MHz, Chloroform-*d*) of BiTA17

**
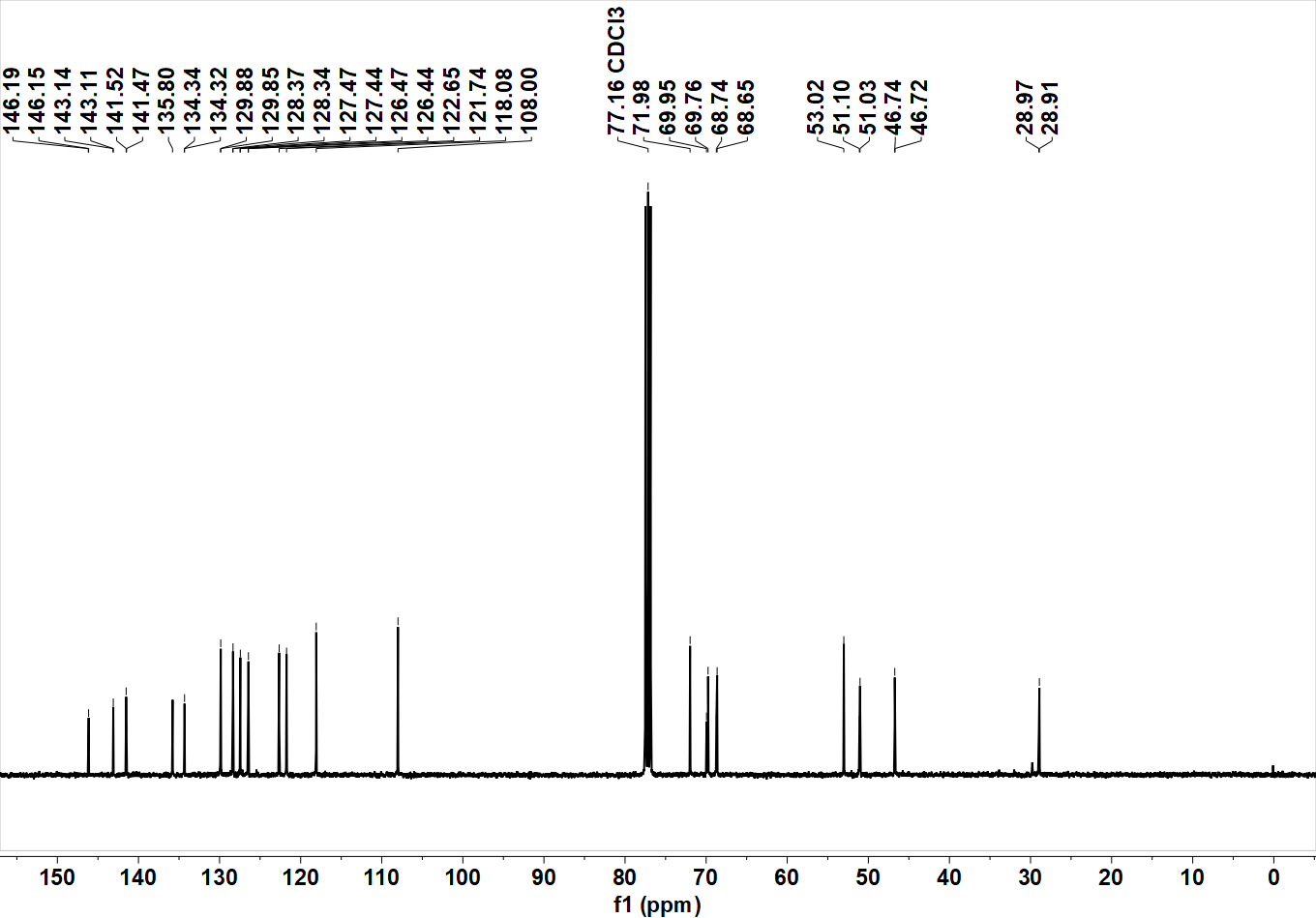
**

**Figure S84.** ^13^C NMR spectrum (101 MHz, Chloroform-*d*) of BiTA17

**Figure S85.** HRMS spectrum of BiTA17

**
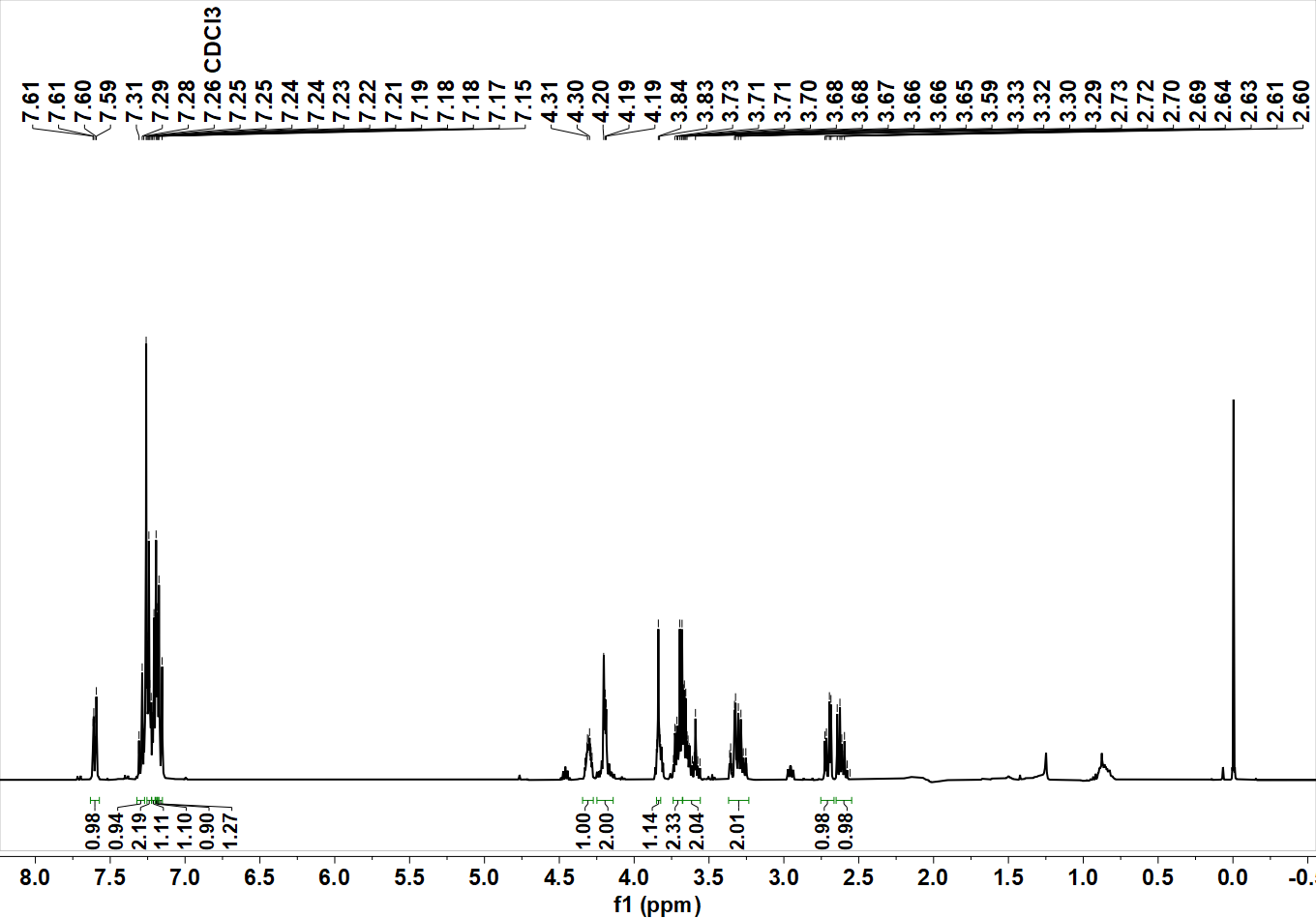
**

**Figure S86.** ^1^H NMR spectrum (400 MHz, Chloroform-*d*) of BiTA18

**
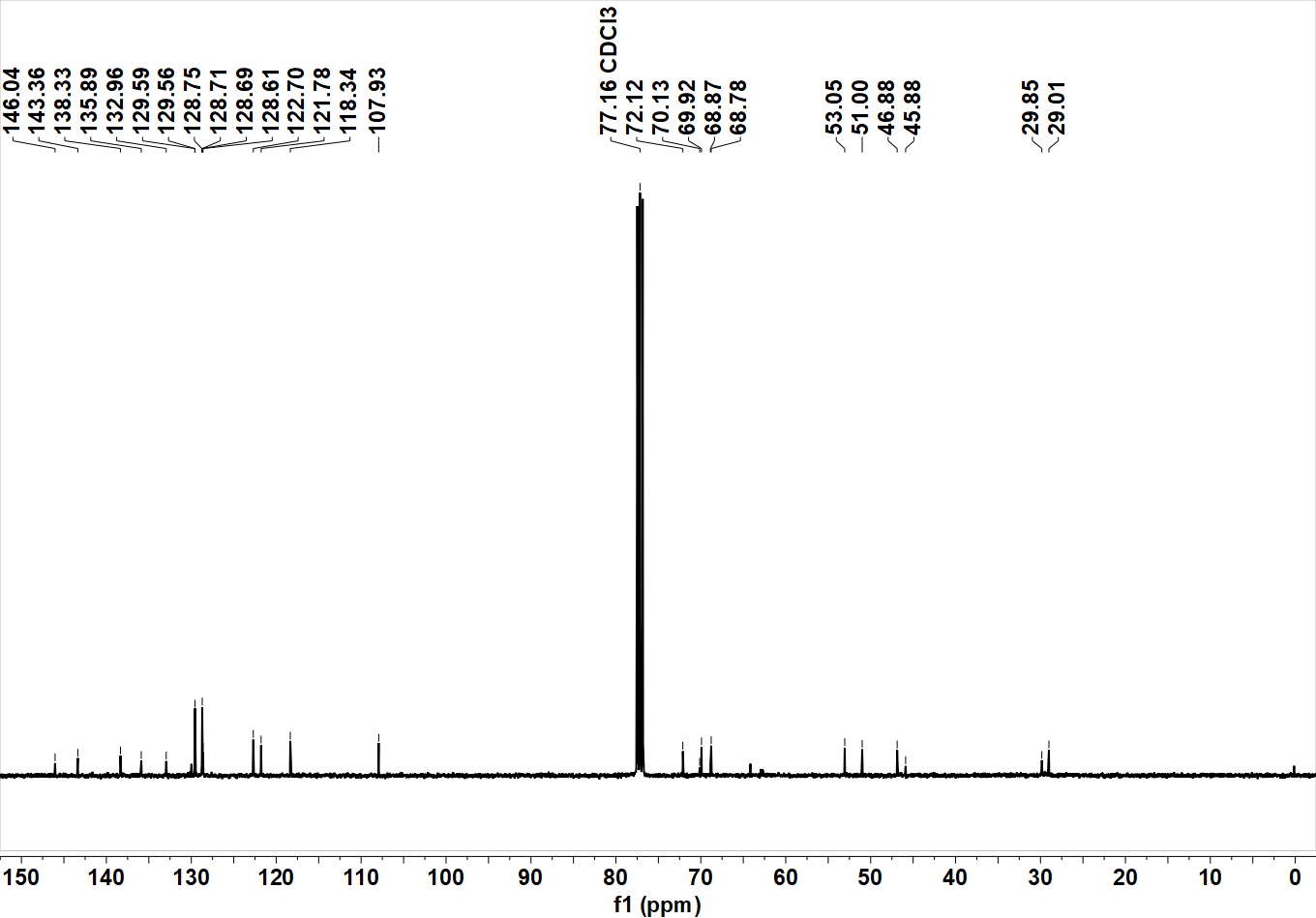
**

**Figure S87.** ^13^C NMR spectrum (101 MHz, Chloroform-*d*) of BiTA18

**Figure S88.** HRMS spectrum of BiTA18

**
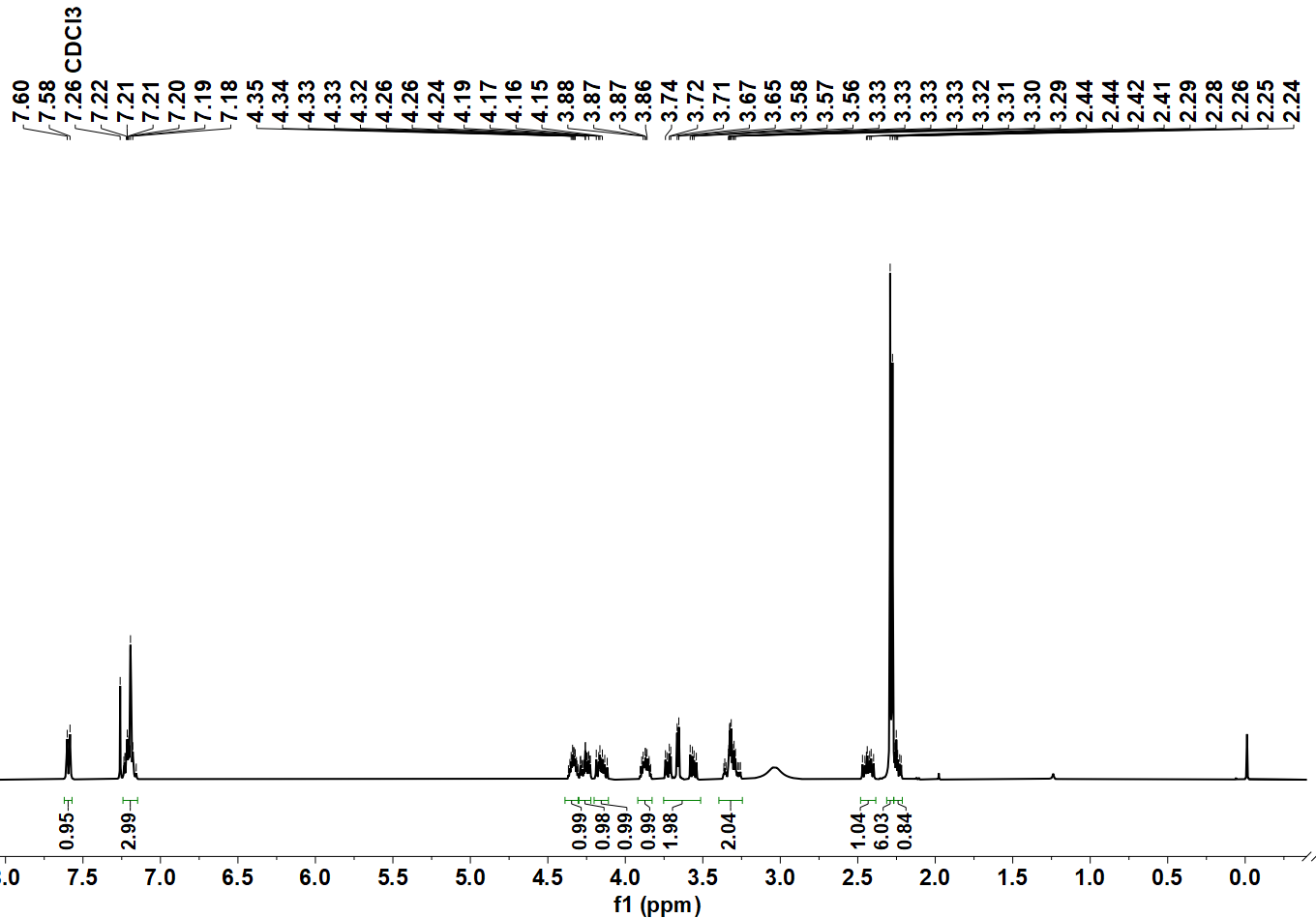
**

**Figure S89.** ^1^H NMR spectrum (400 MHz, Chloroform-*d*) of BiTA19

**
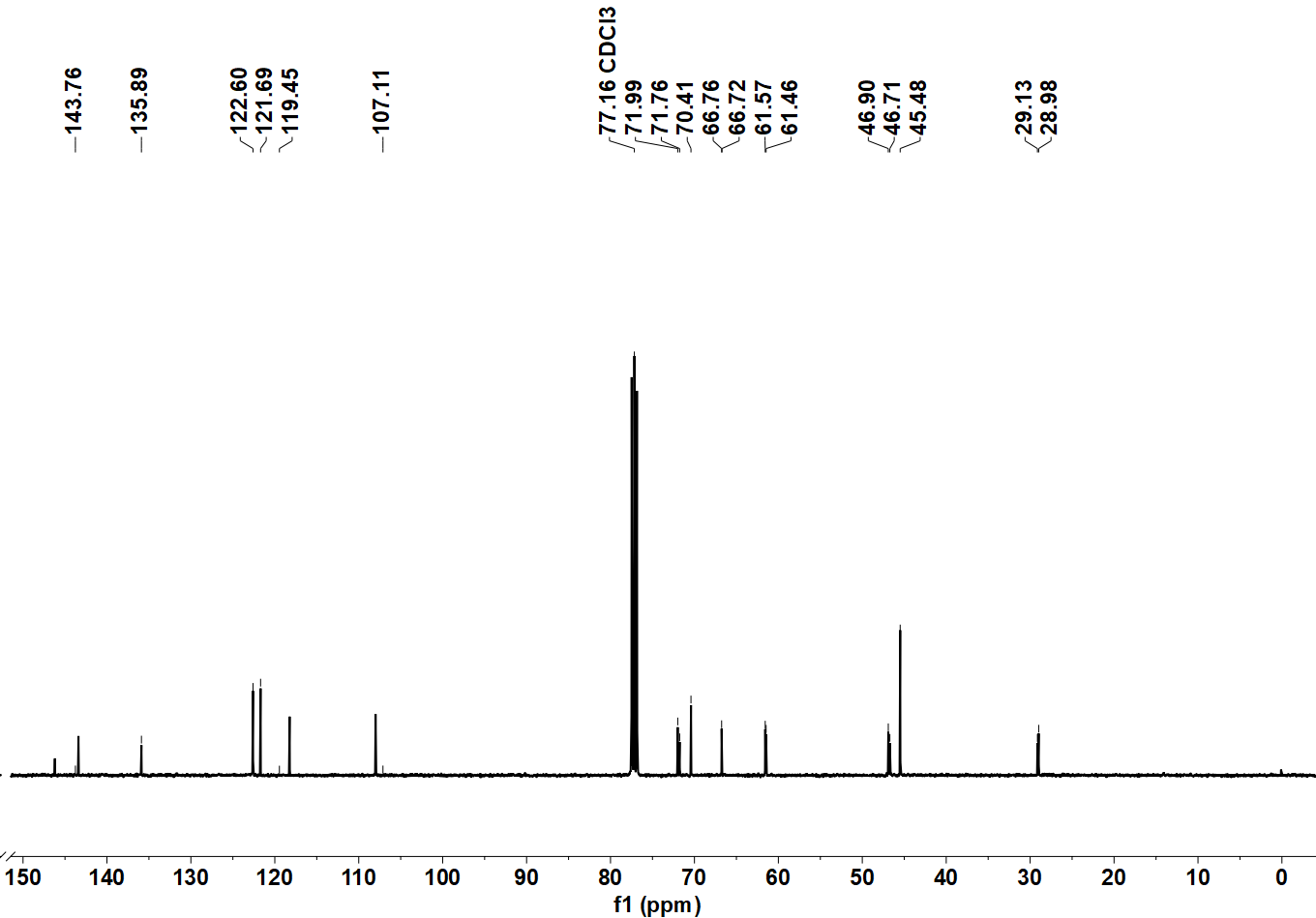
**

**Figure S90.** ^13^C NMR spectrum (101 MHz, Chloroform-*d*) of BiTA19

**Figure S91.** HRMS spectrum of BiTA19

**
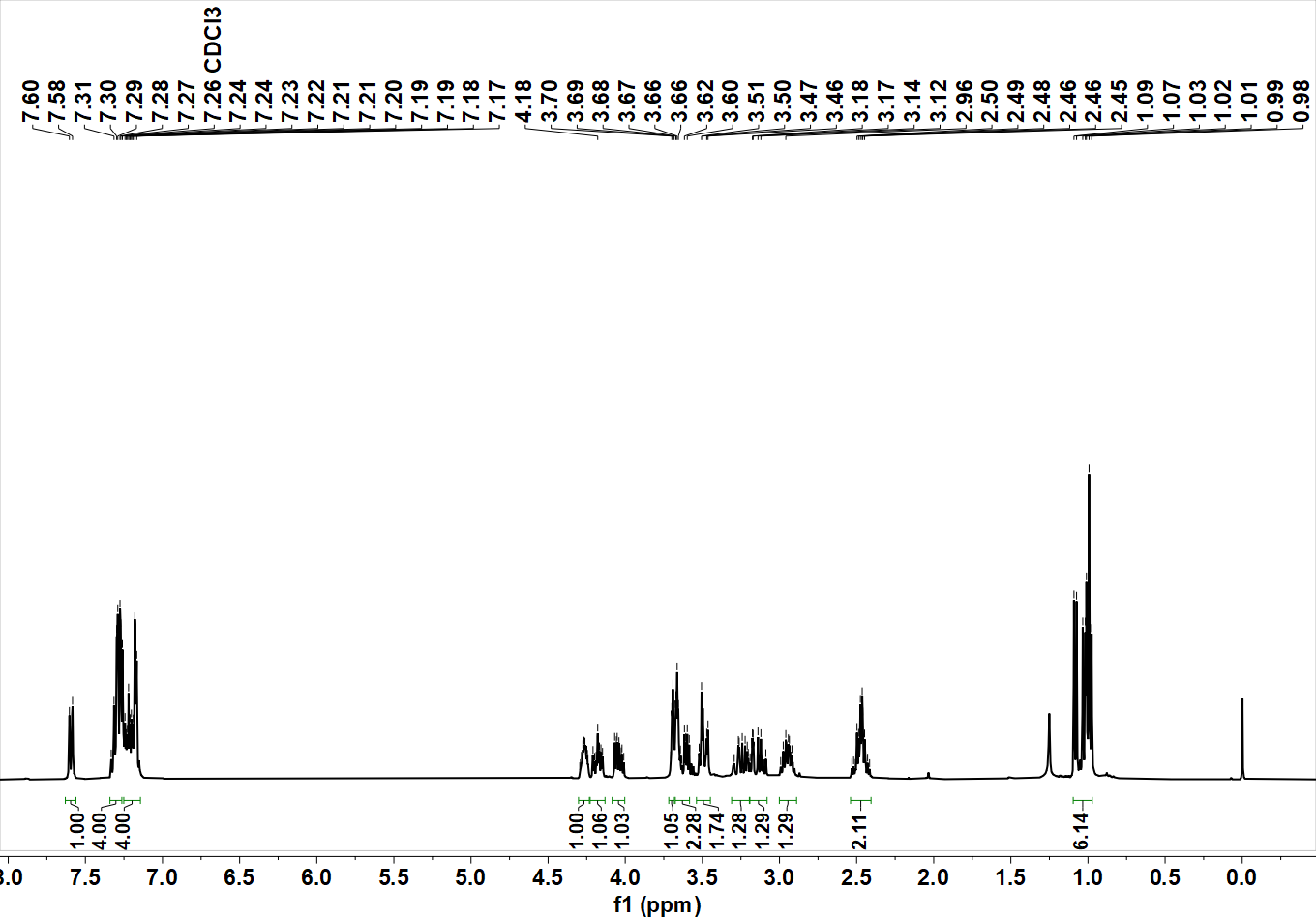
**

**Figure S92.** ^1^H NMR spectrum (400 MHz, Chloroform-*d*) of BiTA20

**
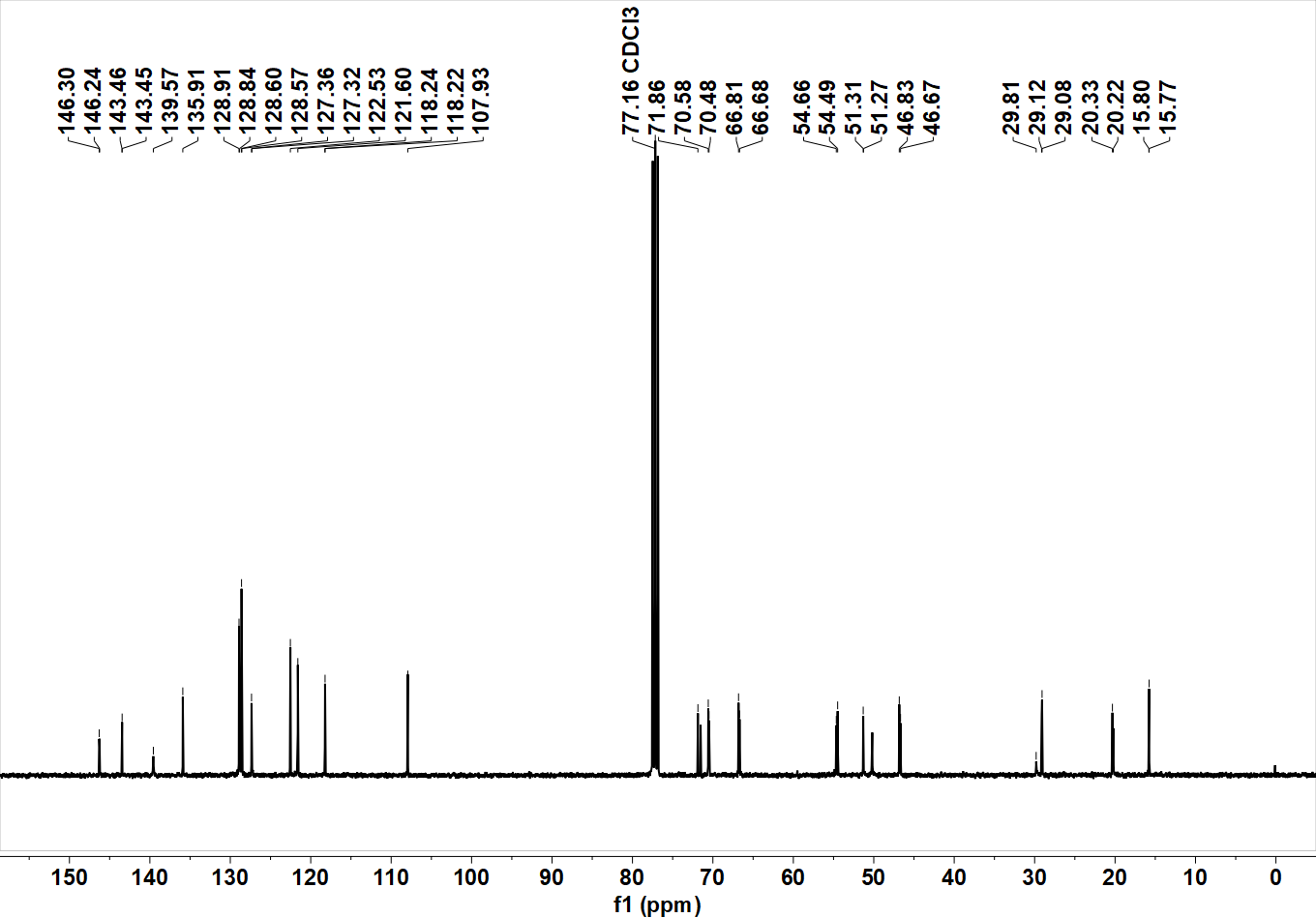
**

**Figure S93.** ^13^C NMR spectrum (101 MHz, Chloroform-*d*) of BiTA20

**Figure S94.** HRMS spectrum of BiTA20

**
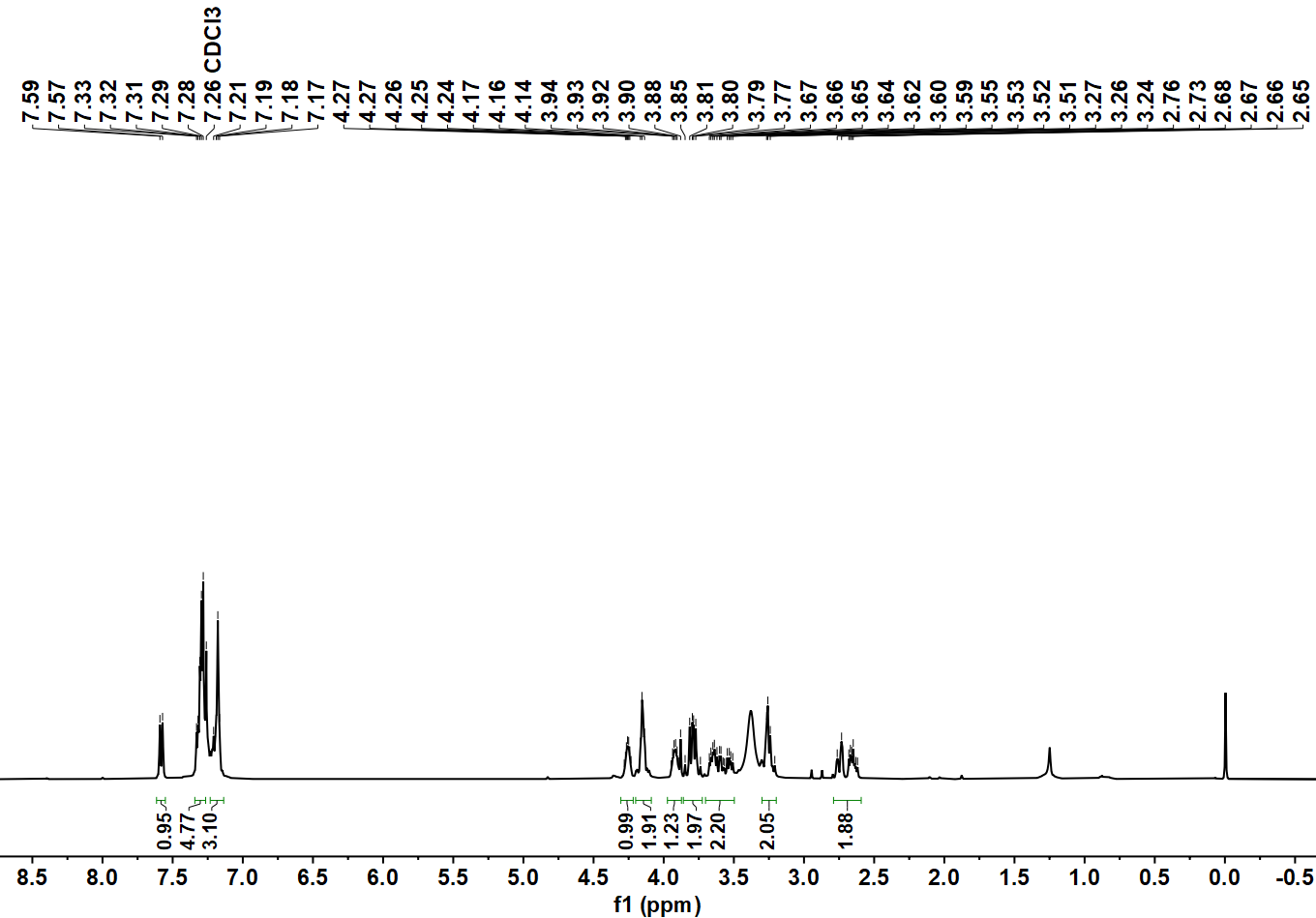
**

**Figure S95.** ^1^H NMR spectrum (400 MHz, Chloroform-*d*) of BiTA21

**
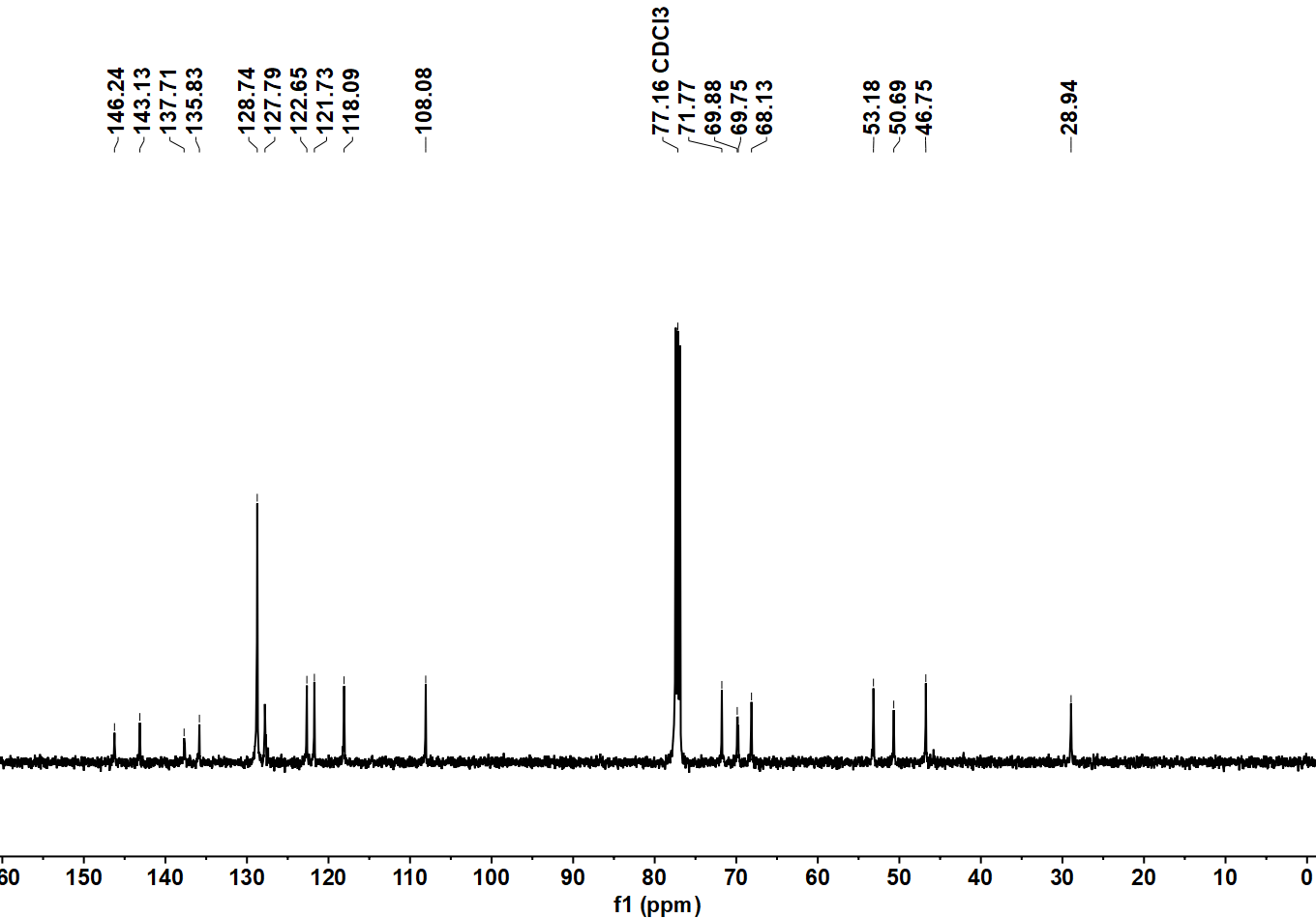
**

**Figure S96.** ^13^C NMR spectrum (101 MHz, Chloroform-*d*) of BiTA21

**Figure S97.** HRMS spectrum of BiTA21

**
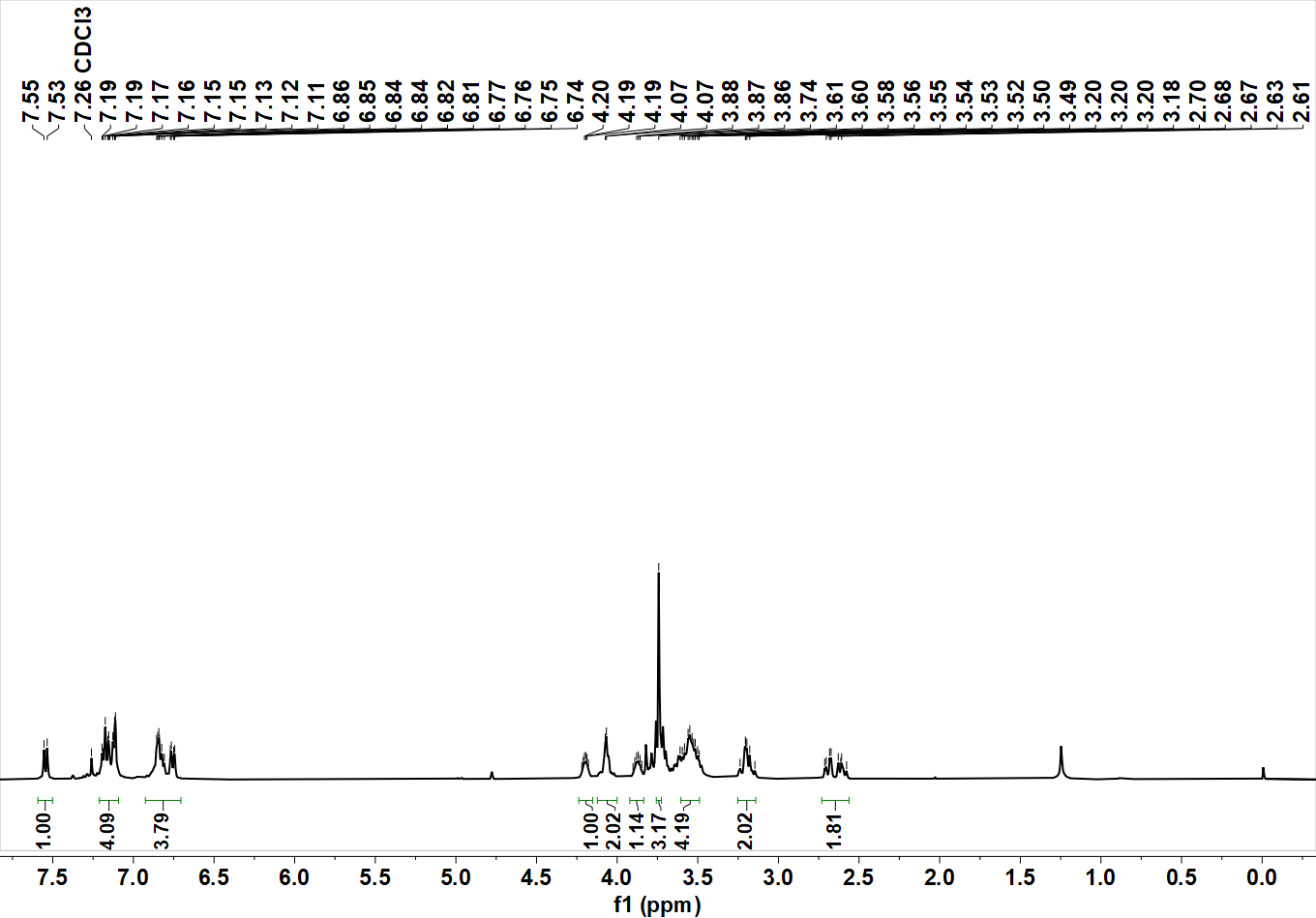
**

**Figure S98.** ^1^H NMR spectrum (400 MHz, Chloroform-*d*) of BiTA22

**
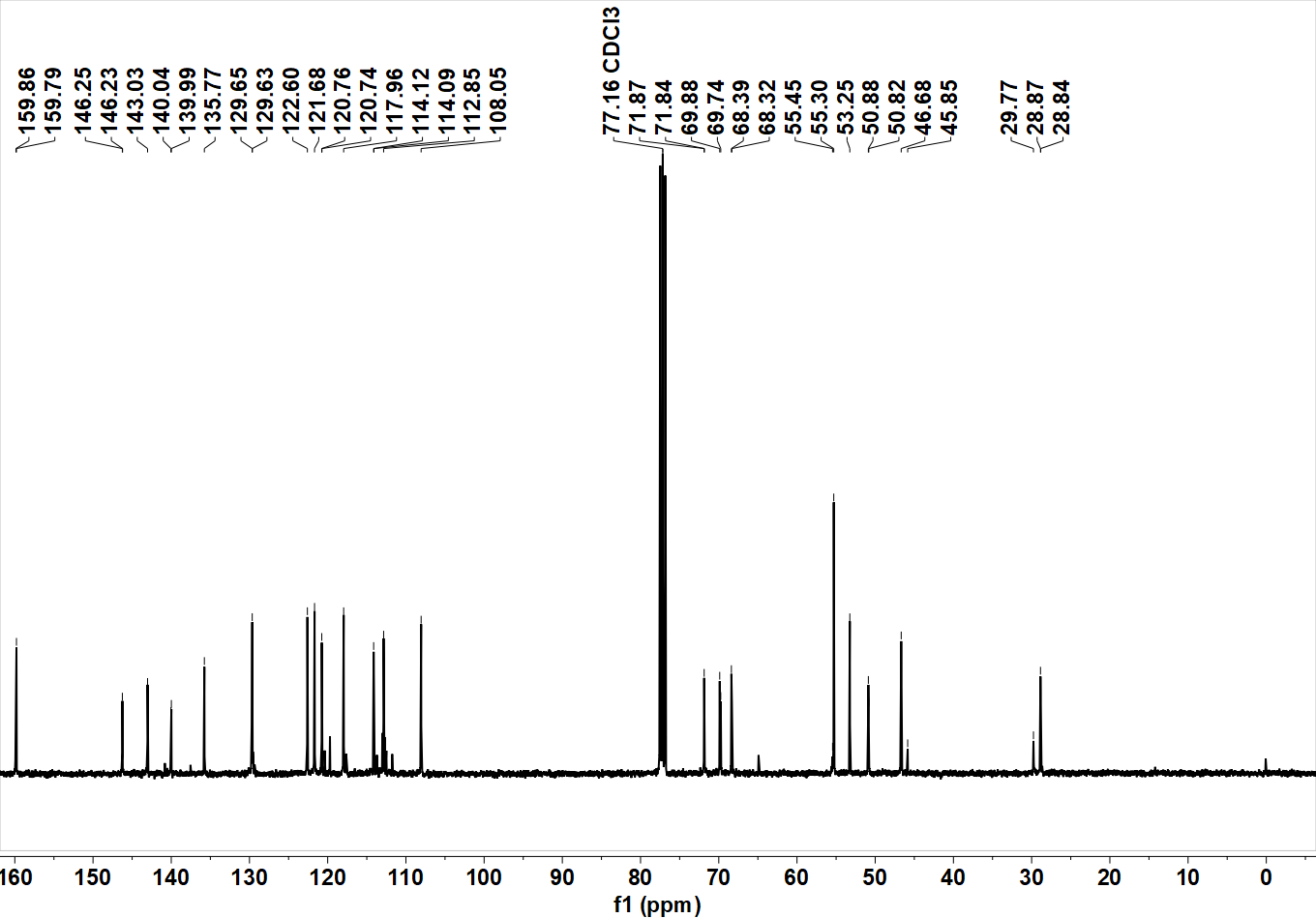
**

**Figure S99.** ^13^C NMR spectrum (101 MHz, Chloroform-*d*) of BiTA22

**Figure S100.** HRMS spectrum of BiTA22

**
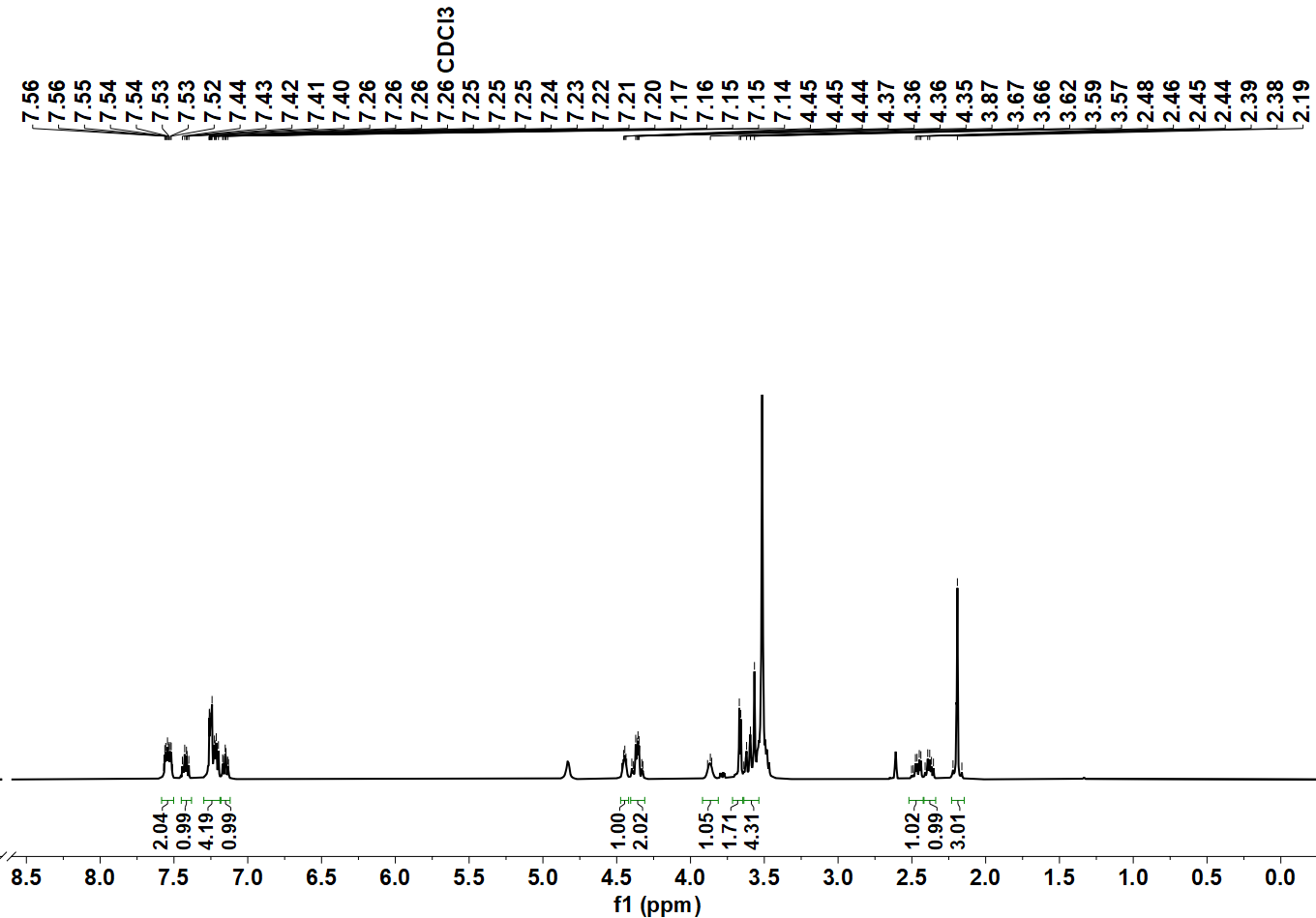
**

**Figure S101.** ^1^H NMR spectrum (500 MHz, Chloroform-*d*) of BiTA23

**
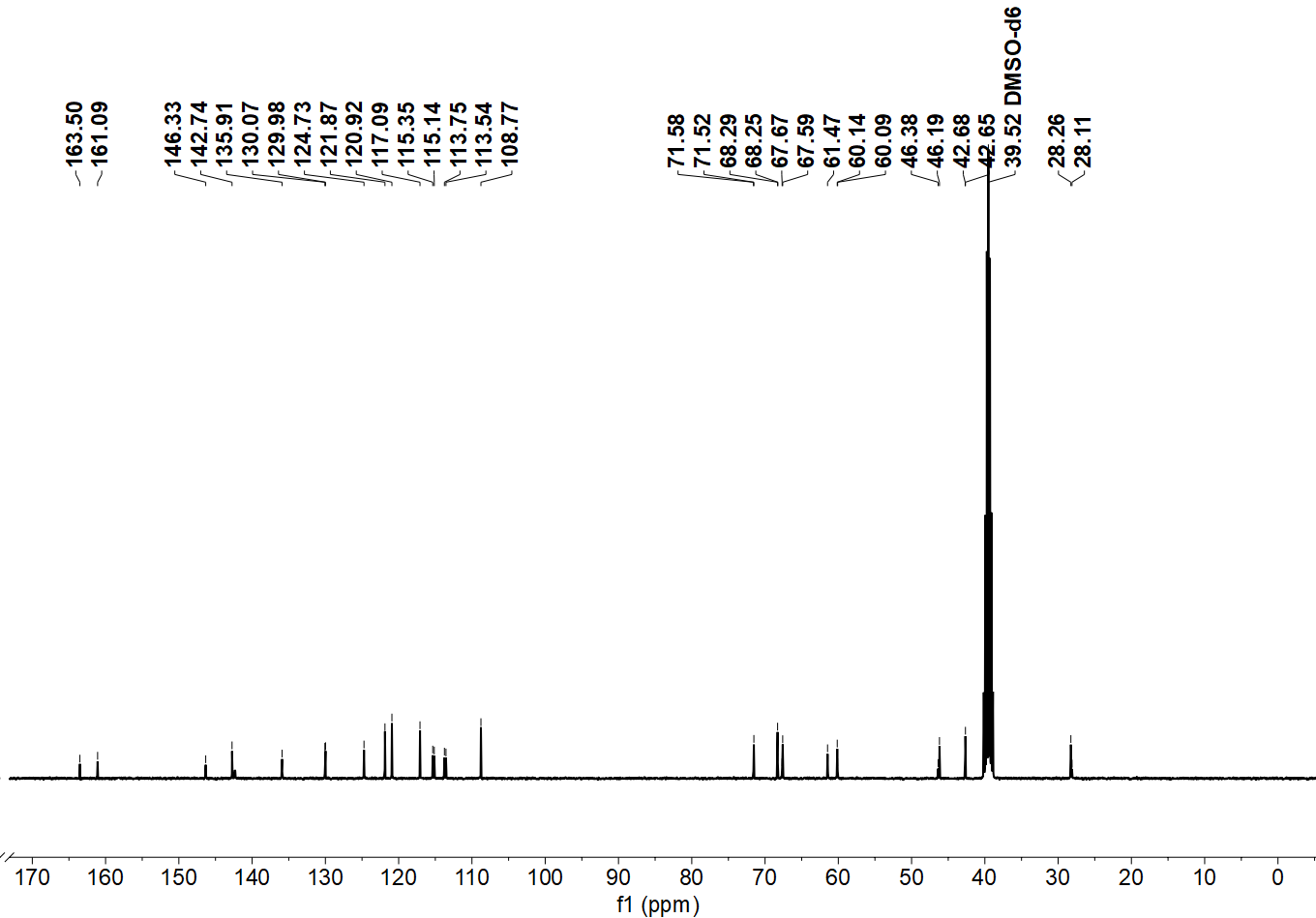
**

**Figure S102.** ^13^C NMR spectrum (126 MHz, DMSO-*d*_6_) of BiTA23

**
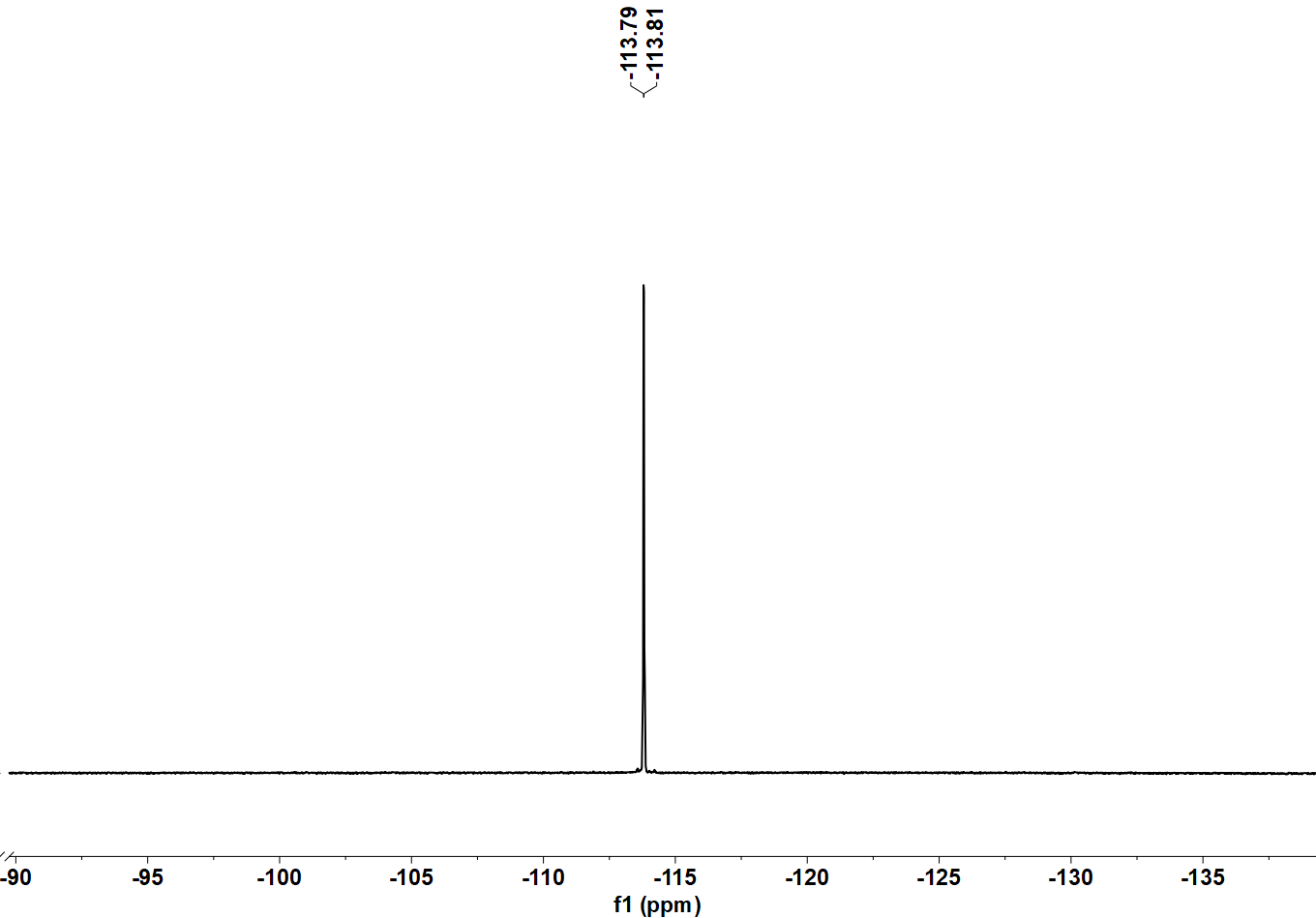
**

**Figure S103.** ^19^F NMR spectrum (471 MHz, DMSO-*d*_6_) of BiTA23

**Figure S104.** HRMS spectrum of BiTA23

**
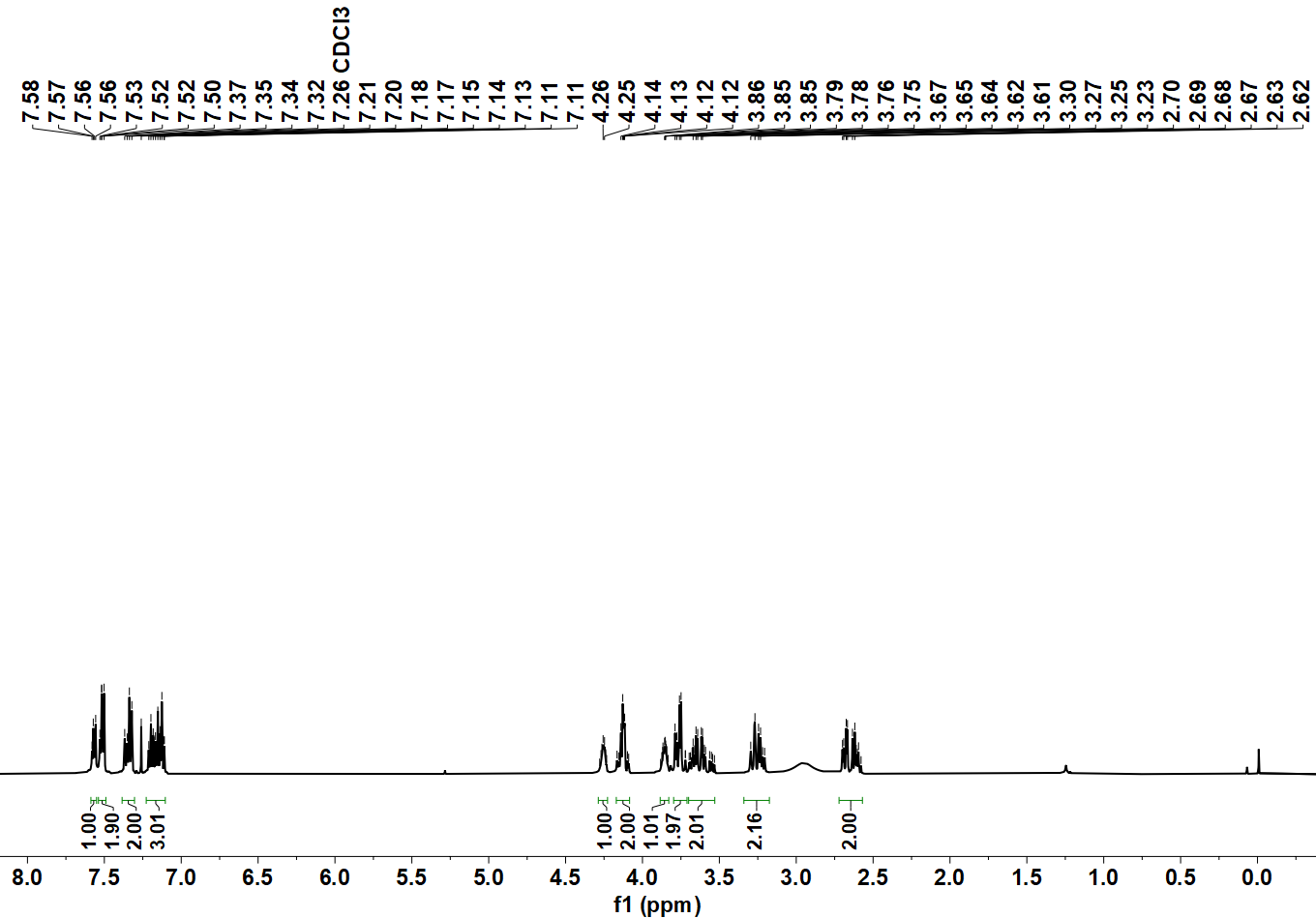
**

**Figure S105.** ^1^H NMR spectrum (500 MHz, Chloroform-*d*) of BiTA24

**
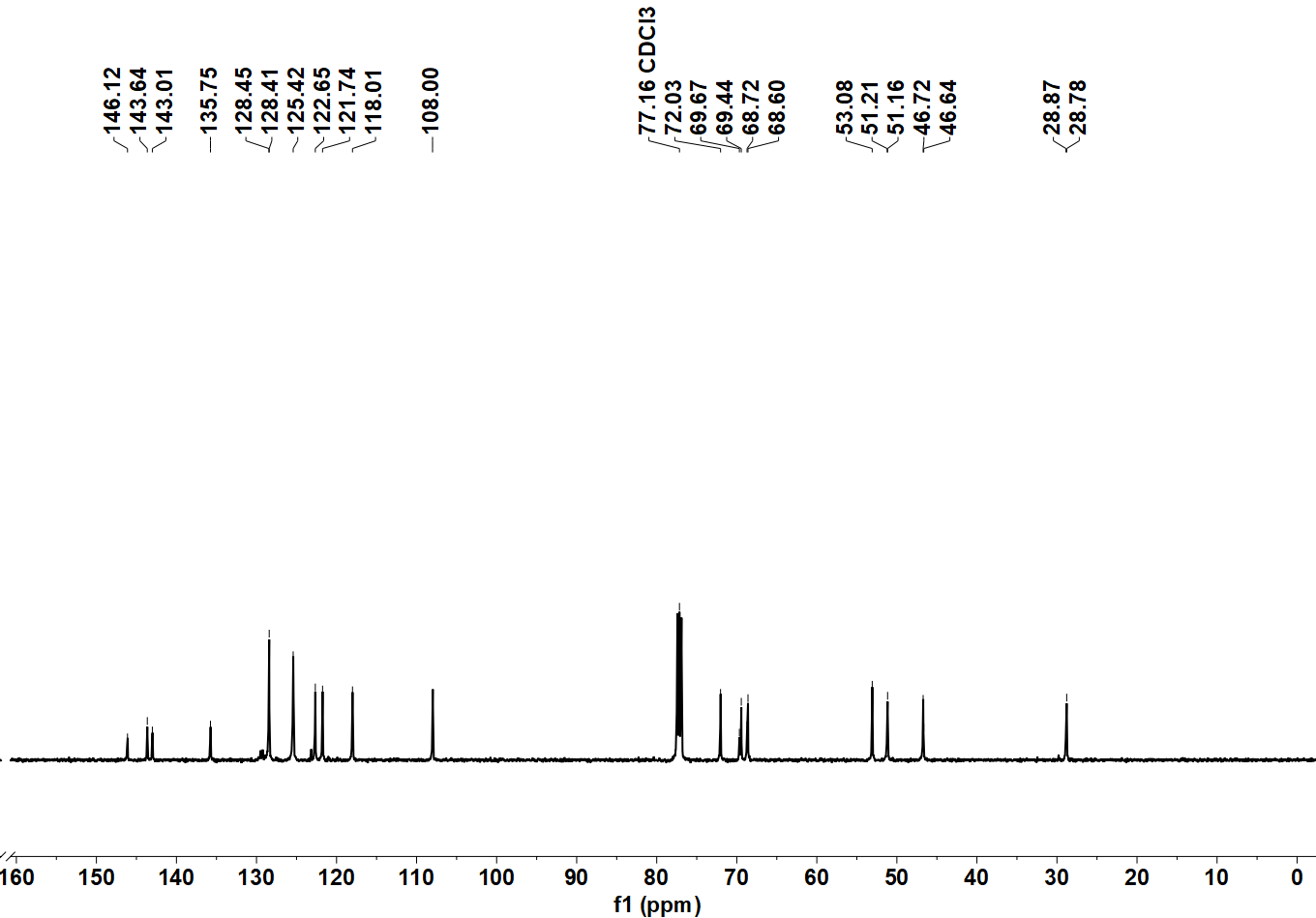
**

**Figure S106.** ^13^C NMR spectrum (126 MHz, DMSO-*d*_6_) of BiTA24

**
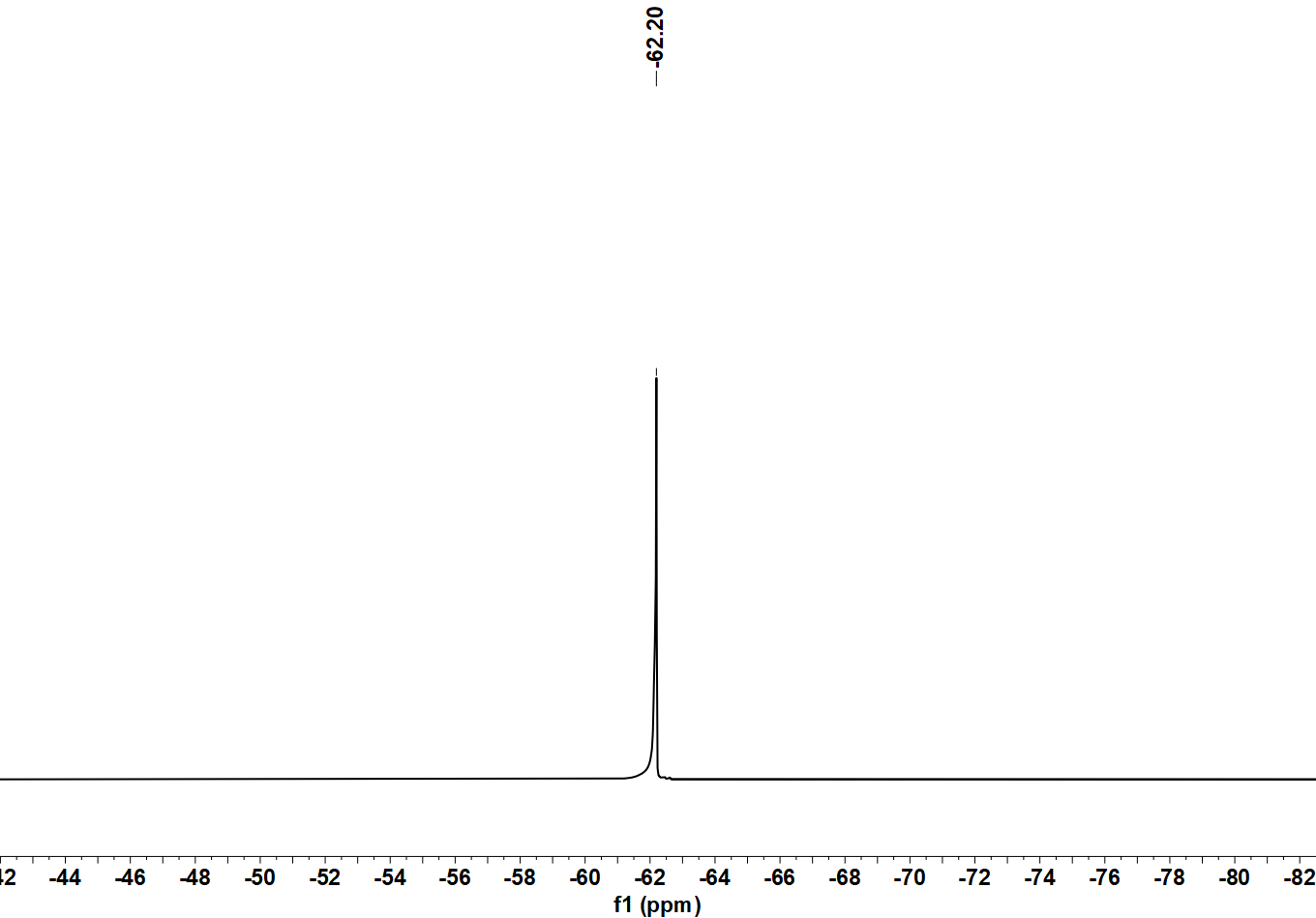
**

**Figure S107.** ^19^F NMR spectrum (471 MHz, DMSO-*d*_6_) of BiTA24

**Figure S108.** HRMS spectrum of BiTA24

**
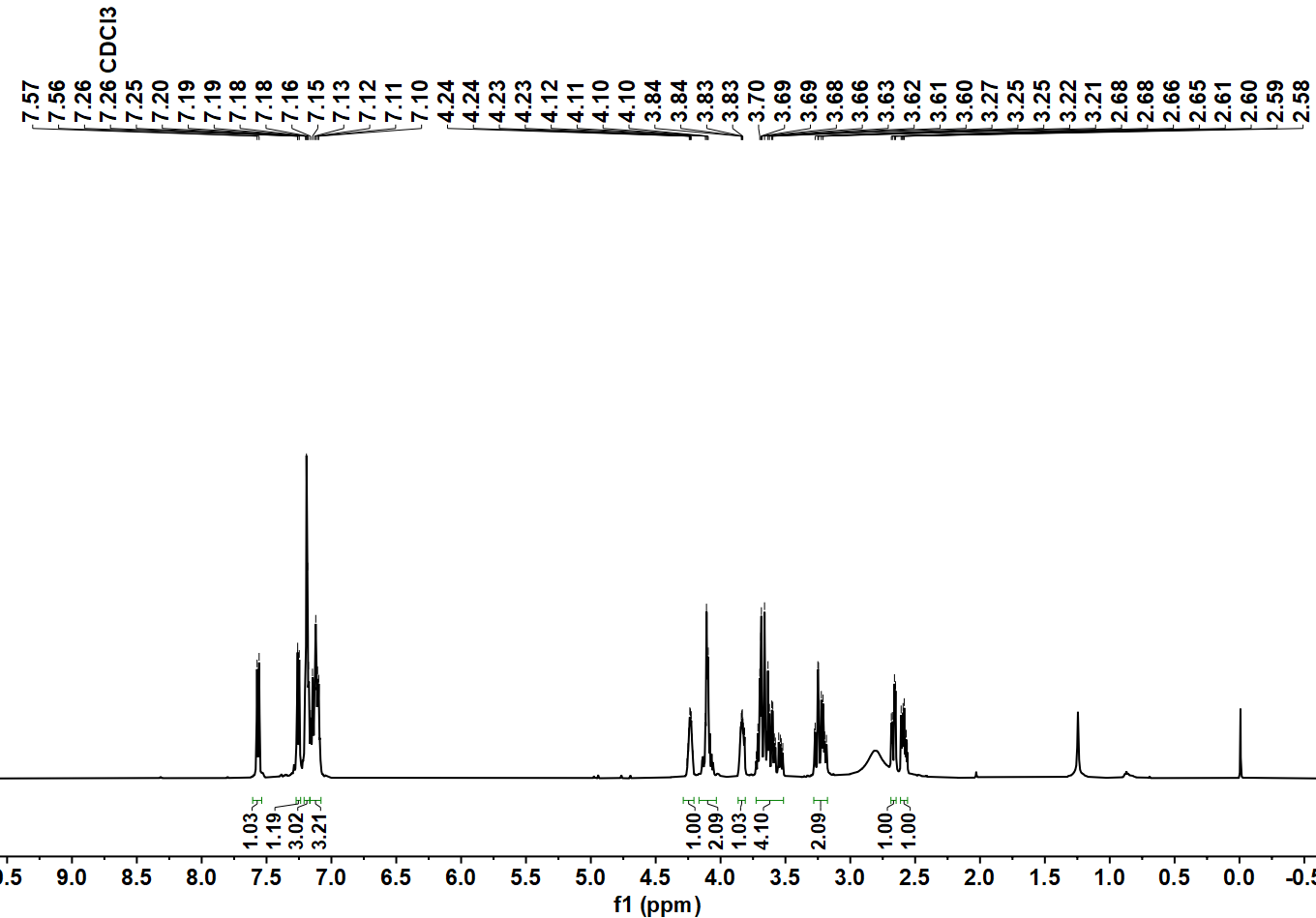
**

**Figure S109.** ^1^H NMR spectrum (500 MHz, Chloroform-*d*) of BiTA25

**
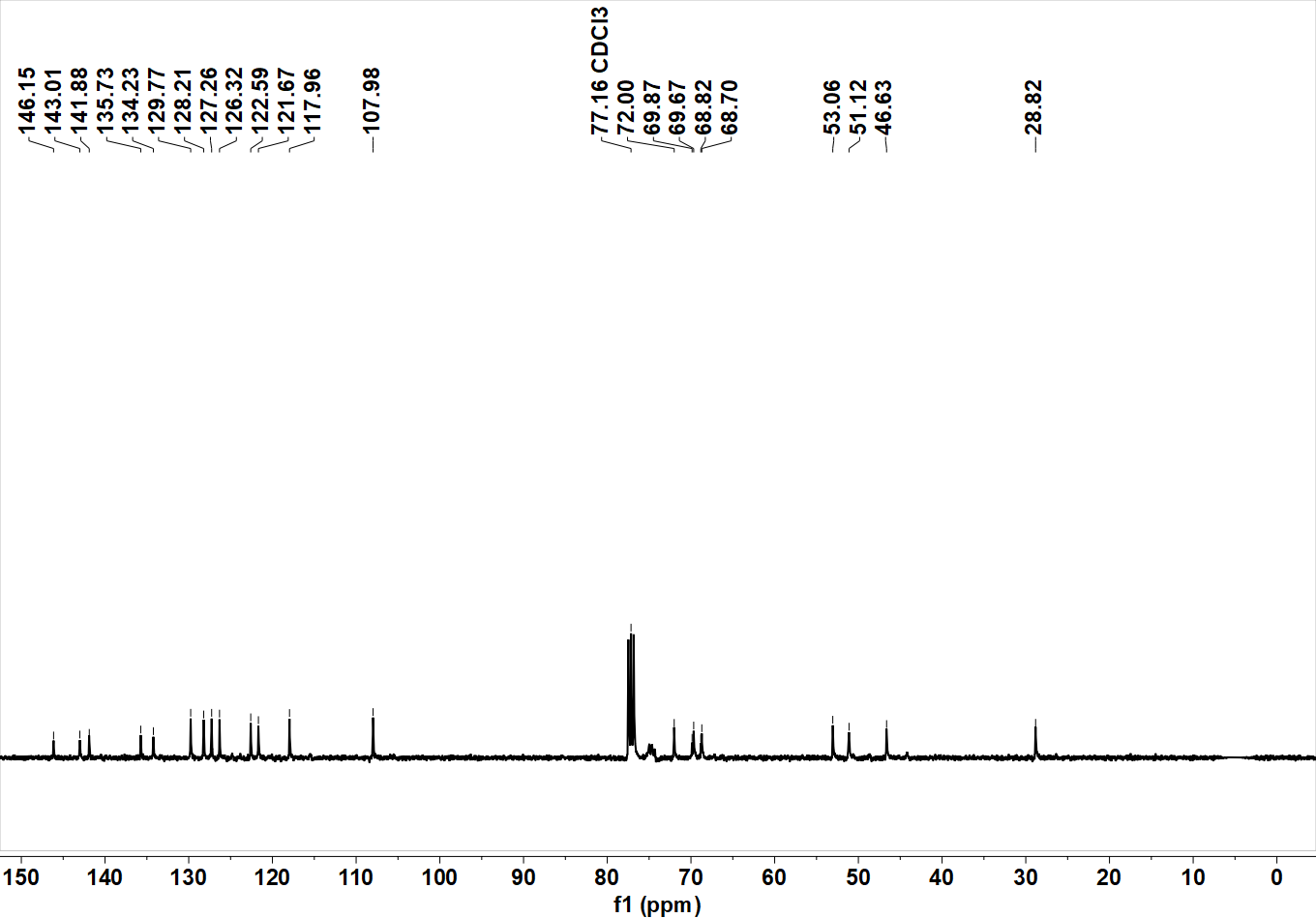
**

**Figure S110.** ^13^C NMR spectrum (126 MHz, DMSO-*d*_6_) of BiTA25

**Figure S111.** HRMS spectrum of BiTA25

**
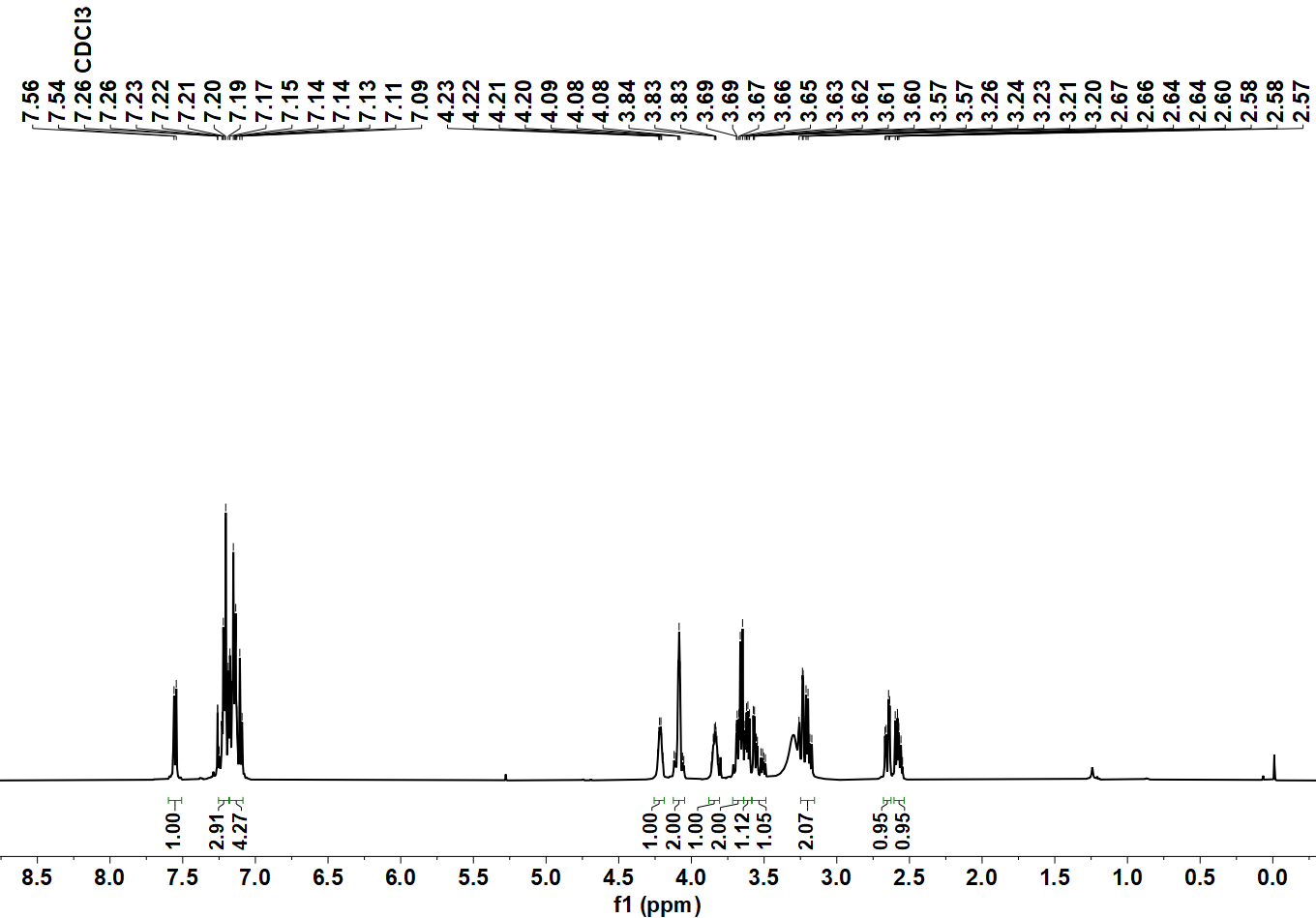
**

**Figure S112.** ^1^H NMR spectrum (500 MHz, Chloroform-*d*) of BiTA26

**
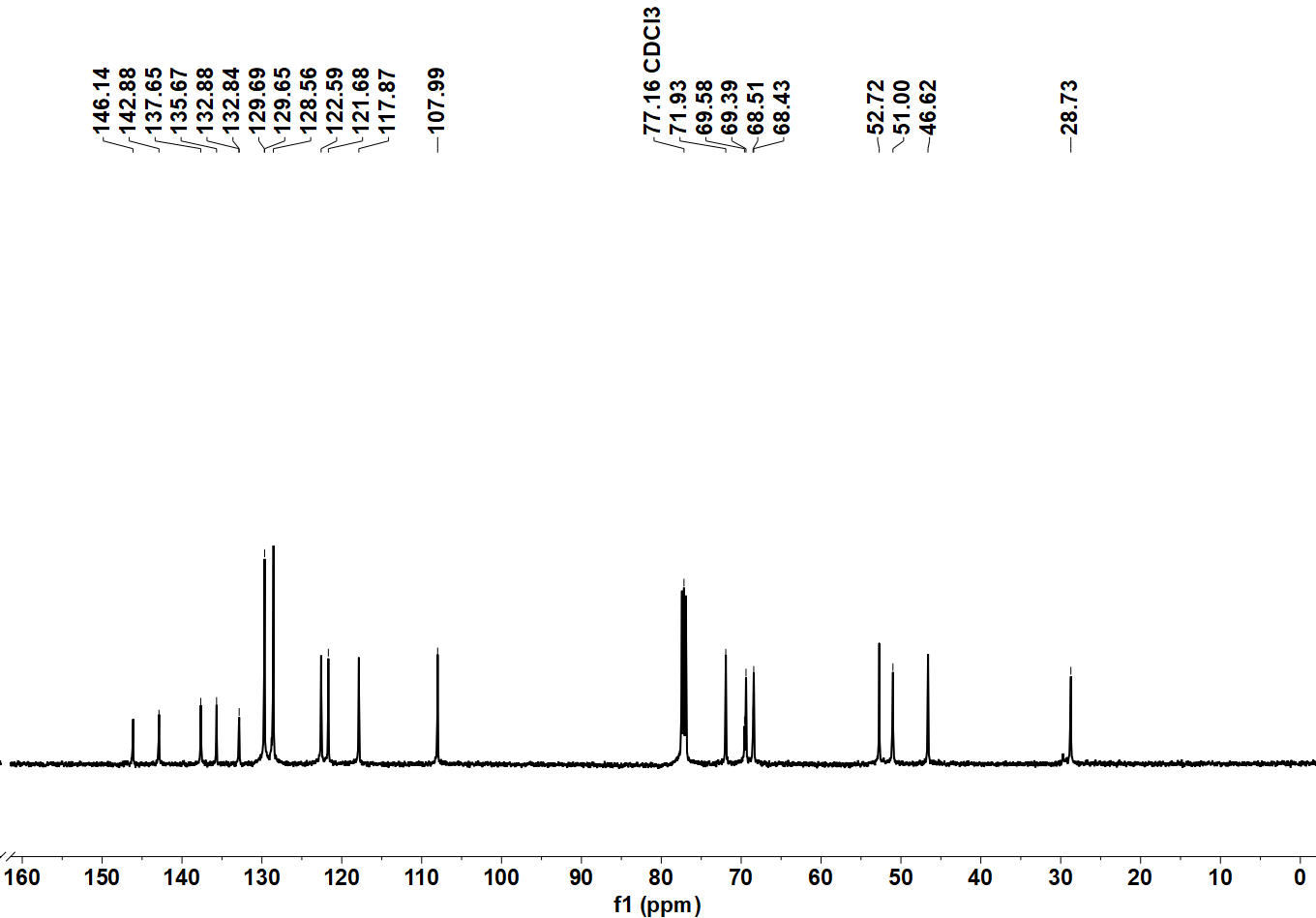
**

**Figure S113.** ^13^C NMR spectrum (126 MHz, Chloroform-*d*) of BiTA26

**Figure S114.** HRMS spectrum of BiTA26

# References

[1] E. Katharopoulos, K. Touloupi, M. Touraki, Monitoring of multiple bacteriocins through a developed dual extraction protocol and comparison of HPLC-DAD with turbidometry as their quantification system, *J. Microbiol. Methods*, **2016**, *127*, 123, https://doi.org/10.1016/j.mimet.2016.06.003.

[2] a) G. Li, W. Xu, H. Qu, D. Tian, H. Zhong, H. Li, Selective wetting and transport of systemic pesticides on bionic stomatal surface regulated by host–guest interaction, *Chem. Eng. J.*, **2024**, *488*, 150878, https://doi.org/10.1016/j.cej.2024.150878; b) Y. Wu, Z. Bao, S. Zhang, et al., Salinity-Driven Interface Self-Assembly of a Biological Amphiphilic Emulsifier to Form Stable Janus Core–Shell Emulsion for Enhancing Agrichemical Delivery, *ACS Nano*, **2024**, *18* (13), 9486, https://doi.org/10.1021/acsnano.3c11919.

[3] J. Tang, X. Tong, Y. Chen, et al., Deposition and water repelling of temperature-responsive nanopesticides on leaves, *Nat. Commun*, **2023**, *14* (1), 6401, https://doi.org/10.1038/s41467-023-41878-3.

[4] K. Ivanova, E. Ramon, U. Wnorowska, et al., Integrated Biofilm Dispersion and Virulence Responsiveness for Targeted Treatment of Pseudomonas aeruginosa Infection in Lungs, *Adv. Funct. Mater.*, **2024**, *34* (46), 2402868 https://doi.org/10.1002/adfm.202402868.

[5] a) Y. Chen, Y. Gao, Y. Huang, Q. Jin, J. Ji, Inhibiting Quorum Sensing by Active Targeted pH-Sensitive Nanoparticles for Enhanced Antibiotic Therapy of Biofilm-Associated Bacterial Infections, *ACS Nano*, **2023**, *17* (11), 10019, https://doi.org/10.1021/acsnano.2c12151; b) C. Jonkergouw, N. K. Beyeh, E. Osmekhina, et al., Repurposing host-guest chemistry to sequester virulence and eradicate biofilms in multidrug resistant Pseudomonas aeruginosa and Acinetobacter baumannii, *Nat. Commun*, **2023**, *14* (1), 2141, https://doi.org/10.1038/s41467-023-37749-6; c) W. Feng, M. Chittò, W. Xie, et al., Poly(d-amino acid) Nanoparticles Target Staphylococcal Growth and Biofilm Disassembly by Interfering with Peptidoglycan Synthesis, *ACS Nano*, **2024**, *18* (11), 8017, https://doi.org/10.1021/acsnano.3c10983; d) S. Xiao, L. Xie, Y. Gao, et al., Artificial Phages with Biocatalytic Spikes for Synergistically Eradicating Antibiotic‐Resistant Biofilms, *Adv. Mater*, **2024**, *36* (32), 2404411, https://doi.org/10.1002/adma.202404411.

[6] A. Vishwakarma, F. Dang, A. Ferrell, H. A. Barton, A. Joy, Peptidomimetic Polyurethanes Inhibit Bacterial Biofilm Formation and Disrupt Surface Established Biofilms, *J. Am. Chem. Soc.*, **2021**, *143* (25), 9440, https://doi.org/10.1021/jacs.1c02324.

[7] a) C. A. Prauchner, G. V. Kozloski, R. Farenzena, Evaluation of sonication treatment and buffer composition on rumen bacteria protein extraction and carboxymethylcellulase activity, *J. Sci. Food Agric.*, **2012**, *93* (7), 1733, https://doi.org/10.1002/jsfa.5959; b) M. Yang, D. Shi, Y. Wang, A. G. Ebadi, M. Toughani, Study on Interaction of Coomassie Brilliant Blue G-250 with Bovine Serum Albumin by Multispectroscopic, *Int. J. Pept. Res. Ther.*, **2020**, *27* (1), 421, https://doi.org/10.1007/s10989-020-10096-6.

[8] S.-M. Kim, J.-P. Suh, Y. Qin, T.-H. Noh, R. F. Reinke, K. K. Jena, Identification and fine-mapping of a new resistance gene, Xa40, conferring resistance to bacterial blight races in rice (Oryza sativa L.), *Theoretical and Applied Genetics*, **2015**, *128* (10), 1933, https://doi.org/10.1007/s00122-015-2557-2.

[9] M. S. Shafique, L. Yapei, L. Man, W. Hongjie, S. Ruyi, W. Chunlian, J. Zhiyuan, Coevolution unveiled: Sulfate transporters mediate rice resistance and susceptibility to Xanthomonas oryzae pv. oryzicola, *Plant Biotechnology Journal*, **2024**, *22* (9), 2632, https://doi.org/10.1111/pbi.14377.
